# Comparative genomics reveals high biological diversity and specific adaptations in the industrially and medically important fungal genus *Aspergillus*

**DOI:** 10.1186/s13059-017-1151-0

**Published:** 2017-02-14

**Authors:** Ronald P. de Vries, Robert Riley, Ad Wiebenga, Guillermo Aguilar-Osorio, Sotiris Amillis, Cristiane Akemi Uchima, Gregor Anderluh, Mojtaba Asadollahi, Marion Askin, Kerrie Barry, Evy Battaglia, Özgür Bayram, Tiziano Benocci, Susanna A. Braus-Stromeyer, Camila Caldana, David Cánovas, Gustavo C. Cerqueira, Fusheng Chen, Wanping Chen, Cindy Choi, Alicia Clum, Renato Augusto Corrêa dos Santos, André Ricardo de Lima Damásio, George Diallinas, Tamás Emri, Erzsébet Fekete, Michel Flipphi, Susanne Freyberg, Antonia Gallo, Christos Gournas, Rob Habgood, Matthieu Hainaut, María Laura Harispe, Bernard Henrissat, Kristiina S. Hildén, Ryan Hope, Abeer Hossain, Eugenia Karabika, Levente Karaffa, Zsolt Karányi, Nada Kraševec, Alan Kuo, Harald Kusch, Kurt LaButti, Ellen L. Lagendijk, Alla Lapidus, Anthony Levasseur, Erika Lindquist, Anna Lipzen, Antonio F. Logrieco, Andrew MacCabe, Miia R. Mäkelä, Iran Malavazi, Petter Melin, Vera Meyer, Natalia Mielnichuk, Márton Miskei, Ákos P. Molnár, Giuseppina Mulé, Chew Yee Ngan, Margarita Orejas, Erzsébet Orosz, Jean Paul Ouedraogo, Karin M. Overkamp, Hee-Soo Park, Giancarlo Perrone, Francois Piumi, Peter J. Punt, Arthur F. J. Ram, Ana Ramón, Stefan Rauscher, Eric Record, Diego Mauricio Riaño-Pachón, Vincent Robert, Julian Röhrig, Roberto Ruller, Asaf Salamov, Nadhira S. Salih, Rob A. Samson, Erzsébet Sándor, Manuel Sanguinetti, Tabea Schütze, Kristina Sepčić, Ekaterina Shelest, Gavin Sherlock, Vicky Sophianopoulou, Fabio M. Squina, Hui Sun, Antonia Susca, Richard B. Todd, Adrian Tsang, Shiela E. Unkles, Nathalie van de Wiele, Diana van Rossen-Uffink, Juliana Velasco de Castro Oliveira, Tammi C. Vesth, Jaap Visser, Jae-Hyuk Yu, Miaomiao Zhou, Mikael R. Andersen, David B. Archer, Scott E. Baker, Isabelle Benoit, Axel A. Brakhage, Gerhard H. Braus, Reinhard Fischer, Jens C. Frisvad, Gustavo H. Goldman, Jos Houbraken, Berl Oakley, István Pócsi, Claudio Scazzocchio, Bernhard Seiboth, Patricia A. vanKuyk, Jennifer Wortman, Paul S. Dyer, Igor V. Grigoriev

**Affiliations:** 1 Westerdijk Fungal Biodiversity Institute, Uppsalalaan 8, 3584 CT Utrecht, The Netherlands; 2 Fungal Molecular Physiology, Utrecht University, Uppsalalaan 8, 3584 CT Utrecht, The Netherlands; 3 US Department of Energy Joint Genome Institute, 2800 Mitchell Drive, Walnut Creek, CA 94598 USA; 4 Department of Food Science and Biotechnology, Faculty of Chemistry, National University of Mexico, Ciudad Universitaria, D.F. C.P. 04510 Mexico; 5 Department of Biology, National and Kapodistrian University of Athens, Panepistimioupolis, 15781 Athens, Greece; 6 Laboratório Nacional de Ciência e Tecnologia do Bioetanol (CTBE), Centro Nacional de Pesquisa em Energia e Materiais (CNPEM), Caixa Postal 6192 CEP 13083-970, Campinas, São Paulo Brasil; 7 Laboratory for Molecular Biology and Nanobiotechnology, National Institute of Chemistry, Hajdrihova 19, 1000 Ljubljana, Slovenia; 8 Department of Biochemical Engineering, Faculty of Science and Technology, University of Debrecen, 4032 Debrecen, Hungary; 9 Institute of Biology Leiden, Molecular Microbiology and Biotechnology, Leiden University, Sylviusweg 72, 2333 BE Leiden, The Netherlands; 10 Department of Molecular Microbiology and Genetics, Institute for Microbiology and Genetics, Georg August University Göttingen, Grisebachstr. 8, 37077 Göttingen, Germany; 11 Department of Biology, Maynooth University, Maynooth, Co. Kildare Ireland; 12 Max Planck Partner Group, Brazilian Bioethanol Science and Technology Laboratory, CEP 13083-100 Campinas, Sao Paulo Brazil; 13 Department of Genetics, Faculty of Biology, University of Seville, Avda de Reina Mercedes 6, 41012 Sevilla, Spain; 14 Fungal Genetics and Genomics Unit, Department of Applied Genetics and Cell Biology, University of Natural Resources and Life Sciences (BOKU) Vienna, Vienna, Austria; 15 Broad Institute of Harvard and MIT, 75 Ames St, Cambridge, MA 02142 USA; 16 College of Food Science and Technology, Huazhong Agricultural University, Wuhan, 430070 China; 17 Department of Biochemistry and Tissue Biology, Institute of Biology, University of Campinas, CEP 13083-862 Campinas, SP Brazil; 18 Department of Biotechnology and Microbiology, Faculty of Science and Technology, University of Debrecen, Egyetem tér 1, 4032 Debrecen, Hungary; 19 Institute of Sciences of Food Production (ISPA), National Research Council (CNR), via Provinciale Lecce-Monteroni, 73100 Lecce, Italy; 20 Institute of Biosciences and Applications, Microbial Molecular Genetics Laboratory, National Center for Scientific Research, Demokritos (NCSRD), Athens, Greece; 21 School of Life Sciences, University of Nottingham, University Park, Nottingham, NG7 2RD UK; 22 CNRS, Aix-Marseille Université, Marseille, France; 23 Institut Pasteur de Montevideo, Unidad Mixta INIA-IPMont, Mataojo 2020, CP11400 Montevideo, Uruguay; 24 INRA, USC 1408 AFMB, 13288 Marseille, France; 25 Department of Biological Sciences, King Abdulaziz University, Jeddah, Saudi Arabia; 26 Department of Food and Environmental Sciences, University of Helsinki, Viikinkaari 9, Helsinki, Finland; 27 Dutch DNA Biotech BV, Utrechtseweg 48, 3703AJ Zeist, The Netherlands; 28 Swammerdam Institute for Life Sciences, University of Amsterdam, Amsterdam, The Netherlands; 29 School of Biology, University of St Andrews, St Andrews, Fife KY16 9TH UK; 30 Department of Medicine, Faculty of Medicine, University of Debrecen, Nagyerdei krt. 98, 4032 Debrecen, Hungary; 31 Department of Medical Informatics, University Medical Centre, Robert-Koch-Str.40, 37075 Göttingen, Germany; 32 Department of Molecular Biology, Universitätsmedizin Göttingen, Humboldtallee 23, Göttingen, 37073 Germany; 33 INRA, Aix-Marseille Univ, BBF, Biodiversité et Biotechnologie Fongiques, Marseille, France; 34 Institute of Sciences of Food Production (ISPA), National Research Council (CNR), Via Amendola 122/O, 70126 Bari, Italy; 35 Departamento de Biotecnología, Instituto de Agroquímica y Tecnología de Alimentos, Consejo Superior de Investigaciones Científicas (CSIC), Paterna, Valencia, Spain; 36 Departamento de Genética e Evolução, Centro de Ciências Biológicas e da Saúde, Universidade Federal de São Carlos, São Carlos, São Paulo Brazil; 37 Uppsala BioCenter, Department of Microbiology, Swedish University of Agricultural Sciences, P.O. Box 7025, 750 07 Uppsala, Sweden; 38 Institute of Biotechnology, Department Applied and Molecular Microbiology, Berlin University of Technology, Gustav-Meyer-Allee 25, 13355 Berlin, Germany; 39 MTA-DE Momentum, Laboratory of Protein Dynamics, Department of Biochemistry and Molecular Biology, University of Debrecen, Nagyerdei krt.98., 4032 Debrecen, Hungary; 40 School of Food Science and Biotechnology, Kyungpook National University, Daegu, 702-701 Republic of Korea; 41 Sección Bioquímica, Departamento de Biología Celular y Molecular, Facultad de Ciencias, Universidad de la República, Montevideo, Uruguay; 42 Department of Microbiology, Karlsruhe Institute of Technology, Institute for Applied Biosciences, Hertzstrasse 16,, 76187 Karlsruhe, Germany; 43 Department of Biology, School of Science, University of Sulaimani, Al Sulaymaneyah, Iraq; 44 Institute of Food Science, Faculty of Agricultural and Food Sciences and Environmental Management, University of Debrecen, 4032 Debrecen, Hungary; 45 Department of Biology, Biotechnical Faculty, University of Ljubljana, Jamnikarjeva 101, 1000 Ljubljana, Slovenia; 46 Systems Biology/Bioinformatics group, Leibniz Institute for Natural Product Research and Infection Biology, Hans Knoell Institute, (HKI), Beutenbergstr. 11a, 07745 Jena, Germany; 47 Department of Genetics, Stanford University, Stanford, CA 94305-5120 USA; 48 Department of Plant Pathology, Kansas State University, Manhattan, KS 66506 USA; 49 Centre for Structural and Functional Genomics, Concordia University, 7141 Sherbrooke Street West, Montreal, QC H4B 1R6 Canada; 50 Department of Biotechnology and Biomedicine, Technical University of Denmark, Søltofts Plads 223, 2800 Kongens Lyngby, Denmark; 51 Departments of Bacteriology and Genetics, University of Wisconsin-Madison, 1550 Linden Drive, Madison, WI 53706 USA; 52 Fungal Biotechnology Team, Pacific Northwest National Laboratory, Richland, Washington, 99352 USA; 53 Department of Molecular and Applied Microbiology, Leibniz-Institute for Natural Product Research and Infection Biology - Hans Knoell Institute (HKI) and Institute for Microbiology, Friedrich Schiller University Jena, Beutenbergstr. 11a, 07745 Jena, Germany; 54 Faculdade de Ciências Farmacêuticas de Ribeirão Preto, Universidade de São Paulo, Av. do Café S/N, CEP 14040-903 Ribeirão Preto, São Paulo Brazil; 55 Department of Molecular Biosciences, University of Kansas, Lawrence, Kansas 66045 USA; 56 Department of Microbiology, Imperial College, London, SW7 2AZ UK; 57 Institute for Integrative Biology of the Cell (I2BC), CEA, CNRS, University Paris‐Sud, Université Paris‐Saclay, 91198 Gif‐sur‐Yvette cedex, France; 58 Research Division Biochemical Technology, Institute of Chemical Engineering, TU Wien, Gumpendorferstraße 1a, 1060 Vienna, Austria; 59 Broad Institute, 415 Main St, Cambridge, MA 02142 USA; 60 Present address: VTT Brasil, Alameda Inajá, 123, CEP 06460-055 Barueri, São Paulo Brazil; 61 Present address: CSIRO Publishing, Unipark, Building 1 Level 1, 195 Wellington Road, Clayton, VIC 3168 Australia; 62 Present address: Université Libre de Bruxelles Institute of Molecular Biology and Medicine (IBMM), Brussels, Belgium; 63 Present address: Instituto de Profesores Artigas, Consejo de Formación en Educación, ANEP, CP 11800, Av. del Libertador 2025, Montevideo, Uruguay; 64 Present Address: Department of Chemistry, University of Ioannina, Ioannina, 45110 Greece; 65 Present address: Center for Algorithmic Biotechnology, St.Petersburg State University, St. Petersburg, Russia; 66 Present address: Aix-Marseille Université, Unité de Recherche sur les Maladies Infectieuses et Tropicales Emergentes (URMITE), UM63, CNRS 7278, IRD 198, INSERM U1095, IHU Méditerranée Infection, Pôle des Maladies Infectieuses, Assistance Publique-Hôpitaux de Marseille, Faculté de Médecine, 27 Bd Jean Moulin, 13005 Marseille, France; 67 Present address: Swedish Chemicals Agency, Box 2, 172 13 Sundbyberg, Sweden; 68 Present address: Instituto de Ciencia y Tecnología Dr. César Milstein, Fundación Pablo Cassará, CONICET, Saladillo 2468 C1440FFX, Ciudad de Buenos Aires, Argentina; 69 Present address: Centre for Structural and Functional Genomics, Concordia University, 7141 Sherbrooke Street West, Montreal, QC H4B 1R6 Canada; 70 Present address: INRA UMR1198 Biologie du Développement et de la Reproduction - Domaine de Vilvert, Jouy en Josas, 78352 Cedex France; 71 Present address: Department Applied and Molecular Microbiology, Institute of Biotechnology, Berlin University of Technology, Gustav-Meyer-Allee 25, 13355 Berlin, Germany; 72 Present address: BaseClear B.V., Einsteinweg 5, 2333 CC Leiden, The Netherlands; 73 Present address: Seres Therapeutics, 200 Sidney St, Cambridge, MA 02139 USA; 74 Present address: Centre of Functional and Structure Genomics Biology Department Concordia University, 7141 Sherbrooke St. W., Montreal, QC H4B 1R6 Canada

**Keywords:** *Aspergillus*, Genome sequencing, Comparative genomics, Fungal biology

## Abstract

**Background:**

The fungal genus *Aspergillus* is of critical importance to humankind. Species include those with industrial applications, important pathogens of humans, animals and crops, a source of potent carcinogenic contaminants of food, and an important genetic model. The genome sequences of eight aspergilli have already been explored to investigate aspects of fungal biology, raising questions about evolution and specialization within this genus.

**Results:**

We have generated genome sequences for ten novel, highly diverse *Aspergillus* species and compared these in detail to sister and more distant genera. Comparative studies of key aspects of fungal biology, including primary and secondary metabolism, stress response, biomass degradation, and signal transduction, revealed both conservation and diversity among the species. Observed genomic differences were validated with experimental studies. This revealed several highlights, such as the potential for sex in asexual species, organic acid production genes being a key feature of black aspergilli, alternative approaches for degrading plant biomass, and indications for the genetic basis of stress response. A genome-wide phylogenetic analysis demonstrated in detail the relationship of the newly genome sequenced species with other aspergilli.

**Conclusions:**

Many aspects of biological differences between fungal species cannot be explained by current knowledge obtained from genome sequences. The comparative genomics and experimental study, presented here, allows for the first time a genus-wide view of the biological diversity of the aspergilli and in many, but not all, cases linked genome differences to phenotype. Insights gained could be exploited for biotechnological and medical applications of fungi.

**Electronic supplementary material:**

The online version of this article (doi:10.1186/s13059-017-1151-0) contains supplementary material, which is available to authorized users.

## Background

The genus *Aspergillus* is one of the best studied genera of filamentous fungi, largely because of the medical (*A. fumigatus*, *A. terreus*), food spoilage (*A. flavus*, *A. parasiticus*), and industrial (*A. niger*, *A. aculeatus*, *A. oryzae*) relevance of some of its species, in addition to the fundamental studies in the model fungus *A. nidulans* that have contributed broadly to our understanding of eukaryotic cell biology and molecular processes. Aspergilli can grow in a wide range of niches, mainly in soils and on dead matter, and some are also capable of colonizing living animal or plant hosts and, in total, approximately 350 species have been identified in this genus [1]. The broad relevance and economic importance of the genus has pushed it to the forefront of fungal research, with one of the largest academic and industrial research communities [2].

*Aspergillus* species are characterized by the unifying feature of the “aspergillum,” an asexual reproductive structure. The aspergilli form a broad monophyletic group, but show large taxonomic divergence with respect to morphology [3] and phylogenetic distance [4]. Genome sequences for three aspergilli [4–6] were among the first to be reported from filamentous fungi and were soon followed by an additional five genomes [7–10]. This has resulted in many genomic, comparative genomic, and post-genomic studies covering a wide variety of topics [11, 12], largely due to the size of the *Aspergillus* research community. These studies were facilitated by genome resources for this genus, such as CADRE [13] and AspGD [14], in which gene curation and functional annotation of reference species were combined with synteny and orthology analysis. The inclusion of these genomes in MycoCosm [15, 16] enabled comparison to sister and more distant genera. These studies also revealed substantial genomic variations between these species and raised questions about the evolution of various aspects of fungal biology within the genus.

In this study, ten novel genome sequences of the genus *Aspergillus* were generated, namely *A. luchuensis*, *A. aculeatus*, *A. brasiliensis*, *A. carbonarius*, *A. glaucus*, *A. sydowii*, *A. tubingensis*, *A. versicolor*, *A. wentii*, and *A. zonatus*. These species were chosen primarily to provide better coverage of the whole genus, to complement the already available genome sequences of *A. clavatus*, *A. fischeri*, *A. flavus*, *A. fumigatus*, *A. nidulans*, *A. niger*, *A. oryzae*, and *A. terreus*, and to allow more detailed data mining of the industrially relevant section *Nigri* (*A. luchuensis*, *A. aculeatus*, *A. brasiliensis*, *A. carbonarius*, *A. niger*, *A. tubingensis*). Additional species from the section *Nidulantes* were included because of the high divergence of the genome sequence of *A. nidulans* from the other *Aspergillus* genomes, and *A. sydowii* because of its marine life-style and being a pathogen of Gorgonian corals [17]. We demonstrate that this combined set of genomes provides a highly valuable dataset for comparative and functional genomics. This study was performed as a global consortium effort with different researchers addressing different topics as subgroups of the consortium. Where possible, experimental data were generated to examine inferences from the genomic differences and to provide an unprecedented comparative analysis of variation and functional specialization within a fungal genus. The paper is organized in topic based sections, covering:

- General comparison of the genomes and phylogenomics
- Asexual and sexual reproduction
- Primary carbon catabolism
- Plant biomass degradation
- Secondary metabolism
- Stress response
- Transporters
- Flavohemoglobins and nitric oxide sensitivity
- Signal transduction pathway genes
- DNA protein organisation and methylation

## Results and discussion

### General comparison of the genomes and phylogenomics

**Fig. 1:**
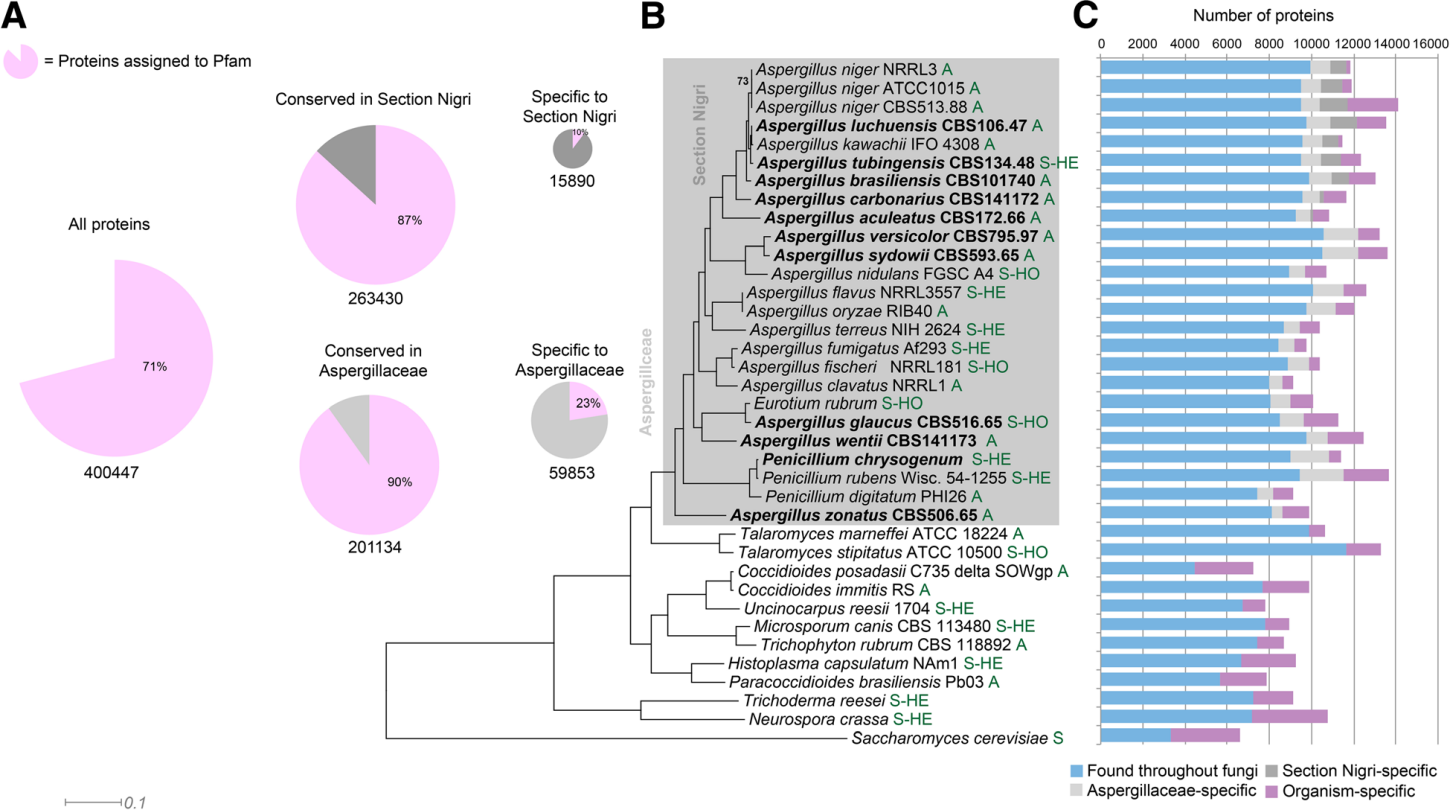
Genome overview of *Aspergillus* and comparative species. **a** Core genes, based on MCL clustering of protein sequences, and cluster membership in section *Nigri* and the Aspergillaceae. *Pink* area of *pie charts* indicates proteins assignable to one or more Pfam domain; *white* or *gray* areas indicate proteins with no Pfam domain. The majority of proteins conserved in Section Nigri, Aspergillaceae, or the full set of comparative fungi could be assigned to a Pfam. Contrastingly, most clade-specific proteins (occurring only in Section Nigri or Aspergillaceae) were not assignable to any Pfam. **b** Maximum likelihood phylogeny inferred from 149 conserved protein sequences. Organisms newly sequenced for this study are indicated in *bold*. All bootstrap values are 100 except where indicated. Section *Nidulantes* is inferred to be a sister group to section *Nigri*, in contrast to previous studies. Letters in *green* behind the strain numbers indicate the reproductive state: *A* asexual, *S-HO* sexual homothallic, *S-HE* sexual heterothallic. **c** Protein conservation, inferred from MCL clustering of proteins, indicates that the majority of proteins in Aspergillaceae have homologs in other fungi, *bars* showing number of proteins being aligned to individual species to the *left-hand side* in (**b**). Some 21% of proteins are specific to the Aspergillaceae, while 14% of proteins in section *Nigri* are specific to that clade. Organism-specific proteins make up 9% for the Aspergillaceae as a whole and 8% for section *Nigri*

The genome sequences were generated at the Joint Genome Institute (JGI) and compared to other fungal genomes in JGI’s MycoCosm database [15] with respect to genome size, structure, and gene content (Table 1, Fig. 1, Additional file 1). All *Aspergillus* genomes are similar in size (29–36 Mb) and GC content (48–53%), while smaller genomes are present in the Onygenales (Table 1, Additional file 1A). The number of predicted genes in the aspergilli is in the range of 9113–13,553, which is generally higher than that observed for the Onygenales (<10,000; Additional file 1B). Larger differences were found for the repetitive portion of the genome, which is in the range of 100 kb to 1.3 Mb for the aspergilli. Genomes of the Aspergillaceae have an average repeat content of 2–3% (Additional file 1C).

**Table 1:** Comparison of genome features of 34 ascomycete genomes

Species	Strain	Class	Order	Section	Genome size (Mb)	GC %	Contigs (*n*)	Reference
*Aspergillus niger*	ATCC1015	Eurotiomycetes	Eurotiales	*Nigri*	35	50.3	24	[7]
*Aspergillus niger*	CBS513.88	Eurotiomycetes	Eurotiales	*Nigri*	34	50.5	470	[10]
***Aspergillus luchuensis***	**CBS106.47**	**Eurotiomycetes**	**Eurotiales**	***Nigri***	**37**	**49.1**	**318**	**This study**
***Aspergillus tubingensis***	**CBS134.48**	**Eurotiomycetes**	**Eurotiales**	***Nigri***	**35**	**49.2**	**87**	**This study**
***Aspergillus brasiliensis***	**CBS101740**	**Eurotiomycetes**	**Eurotiales**	***Nigri***	**36**	**50.5**	**290**	**This study**
***Aspergillus carbonarius***	**CBS141172**	**Eurotiomycetes**	**Eurotiales**	***Nigri***	**36**	**51.7**	**1346**	**This study**
***Aspergillus aculeatus***	**CBS172.66**	**Eurotiomycetes**	**Eurotiales**	***Nigri***	**35**	**50.9**	**851**	**This study**
***Aspergillus versicolor***	**CBS795.97**	**Eurotiomycetes**	**Eurotiales**	***Nidulantes***	**33**	**50.1**	**58**	**This study**
***Aspergillus sydowii***	**CBS593.65**	**Eurotiomycetes**	**Eurotiales**	***Nidulantes***	**34**	**50.0**	**133**	**This study**
*Aspergillus nidulans*	FGSC A4	Eurotiomycetes	Eurotiales	*Nidulantes*	30	50.4	82	[4]
*Aspergillus flavus*	NRRL3557	Eurotiomycetes	Eurotiales	*Flavi*	37	48.4	765	[9]
*Aspergillus oryzae*	RIB40	Eurotiomycetes	Eurotiales	*Flavi*	38	48.3	28	[5]
*Aspergillus terreus*	NIH2624	Eurotiomycetes	Eurotiales	*Terrei*	29	52.9	267	[14]
*Aspergillus fumigatus*	Af293	Eurotiomycetes	Eurotiales	*Fumigati*	29	49.8	19	[6]
*Aspergillus fischeri*	NRRL181	Eurotiomycetes	Eurotiales	*Fumigati*	33	49.4	976	[8]
*Aspergillus clavatus*	NRRL1	Eurotiomycetes	Eurotiales	*Clavati*	28	49.2	143	[8]
***Aspergillus glaucus***	**CBS516.65**	**Eurotiomycetes**	**Eurotiales**	***Aspergillus***	**30**	**49.4**	**433**	**This study**
***Aspergillus wentii***	**CBS141173**	**Eurotiomycetes**	**Eurotiales**	***Cremei***	**31**	**48.0**	**118**	**This study**
***Penicillium chrysogenum***	**unknown**	**Eurotiomycetes**	**Eurotiales**	***Chrysogena***	**31**	**48.8**	**299**	**This study**
*Penicillium rubens*	Wisconsin 54-1255	Eurotiomycetes	Eurotiales	*Chrysogena*	32	49.0	242	[18]
*Penicillium digitatum*	PHI26	Eurotiomycetes	Eurotiales	*Penicillium*	26	48.9	304	[19]
***Aspergillus zonatus***	**CBS506.65**	**Eurotiomycetes**	**Eurotiales**	**Unclassified**	**29**	**48.3**	**460**	**This study**
*Talaromyces marneffei*	ATCC18224	Eurotiomycetes	Eurotiales	*Talaromyces*	29	46.7	524	Unpublished
*Talaromyces stipitatus*	ATCC10500	Eurotiomycetes	Eurotiales	*Talaromyces*	36	46.1	896	Unpublished
*Coccidioides immitis*	RS	Eurotiomycetes	Onygenales		29	46.0	10	[20]
*Coccidioides posadasii*	C735 delta SOWgp	Eurotiomycetes	Onygenales		27	46.6	55	[20]
*Uncinocarpus reesii*	1704	Eurotiomycetes	Onygenales		22	48.7	582	[20]
*Microsporum canis*	CBS113480	Eurotiomycetes	Onygenales		23	47.5	309	[21]
*Trichophyton rubrum*	CBS118892	Eurotiomycetes	Onygenales		23	48.3	622	[21]
*Histoplasma capsulatum*	NAm1	Eurotiomycetes	Onygenales		33	46.2	2867	[20]
*Paracoccidioides brasiliensis*	Pb03	Eurotiomycetes	Onygenales		29	44.5	552	[22]
*Trichoderma reesei*	QM6a	Sordariomycetes	Hypocreales		33	52.8	131	[23]
*Neurospora crassa*	OR74A	Sordariomycetes	Sordariales		41	48.3	411	[24]
*Saccharomyces cerevisiae*	S288C	Saccharomycetes	Saccharomycetales		12	38.3	16	[25]

The novel genomes generated in this study are in bold. The order of species follows the taxonomic organization (Fig. 1a)

A substantial proportion (80% on average) of the identified genes in Aspergillaceae genomes have homologs in other lineages of fungi, indicating that around 20% are specific to Aspergillaceae. Many such genes are specific to a clade, e.g. the genomes of section *Nigri* contain an average of ~1800 *Nigri*-specific genes (Fig. 1a). The number of unique genes per species differs significantly, even between the three *A. niger* genomes included in the analysis (Fig. 1b and c). However, different approaches to genome annotation and gene calling may have contributed to these differences, given that not all genomes were sequenced at the same time or by the same technology, nor analyzed with the same software. Therefore, the true number of unique genes is likely to be lower. There is also the possibility that some apparent differences are caused by genes missing due to gaps in the different genome sequences. Protein sequence identity within the Aspergillaceae ranges from 64% (e.g. between *A. zonatus* and *A. versicolor*) to 99% in *A. niger* strains NRRL3 and ATCC 1015, which compares to the average 68% amino acid identity reported in previous studies, and is similar to the divergence between mammals and fish [3].

A genome-wide phylogenetic analysis was conducted to determine the relationship between the newly sequenced genomes and other related species present in MycoCosm. This resulted in a highly supported phylogram providing insights into taxonomic relationships within *Aspergillus* and between *Aspergillus* and related genera. With the introduction of the single name nomenclature for fungal anamorph and teleomorph forms, there is discussion over whether to split *Aspergillus* or to maintain the genus as one. One argument for splitting is the indication that the genus is paraphyletic. In previous taxonomic studies on *Aspergillaceae*, up to five genes were studied and deeper nodes in these phylograms were either poorly or not supported [26, 27]. An important finding is that our analysis using 149 conserved protein sequences (Fig. 1b) places almost all *Aspergillus* species in a monophyletic clade that is sister to a clade containing *Penicillium* species. The only exception was *A. zonatus*, which is consistent with studies suggesting that *A. zonatus* is incorrectly classified in *Aspergillus* and is instead most closely related to *Penicilliopsis clavariiformis* [26]. The genus *Aspergillus* has traditionally been classified into a number of subgenera and sections based on morphological and phylogenetic analysis, although precise relationships for many groups have remained uncertain [19]. Our analysis indicates that the subgenus *Circumdati* appears to be polyphyletic, with *A. flavus*, *A. oryzae*, and *A. terreus* forming one clade and the black aspergilli (sect. *Nigri*) forming a separate clade. *Aspergillus versicolor*, *A. sydowii*, and *A. nidulans*, all members of the section *Nidulantes*, were positioned between these two clades. *Aspergillus wentii* occupied a basal position in the subgenus *Aspergillus* near to *A. glaucus* and *A. ruber*, consistent with the xerophilic nature of other species in this subgenus. Within the section *Nigri*, *A. luchuensis* (previously referred to as *A. foetidus* or *A. acidus* [28]) and *A. tubingensis* were sister species related to *A. niger*, while *A. brasiliensis* occupied a more basal position in agreement with other phylogenetic analyses [1, 29].

### Asexual and sexual reproduction

One reason for the widespread occurrence of *Aspergillus* species is their ability to produce prolific numbers of mitotically derived conidia, which are dispersed by wind and colonize new substrates. These asexual spores are produced from conidiophores, which have a characteristic structure in most aspergilli consisting of a stalk with a vesicle bearing phialides from which chains of conidia radiate outwards [30, 31]. About 64% of *Aspergillus* species are known to reproduce only by asexual means, but the remainder can also reproduce sexually, forming structures known as cleistothecia within which meiotically derived ascospores are produced [32]. Where sexual reproduction occurs, it involves either homothallic (self-fertility) or heterothallic (obligate out-crossing) breeding systems, with different types of cleistothecia produced according to taxonomic grouping [33, 34]. The diversity of reproductive modes and their associated morphologies in the aspergilli provide excellent opportunities to study the evolutionary genetics of asexual and sexual reproduction in fungi.

#### Asexual reproduction

**Fig. 2:**
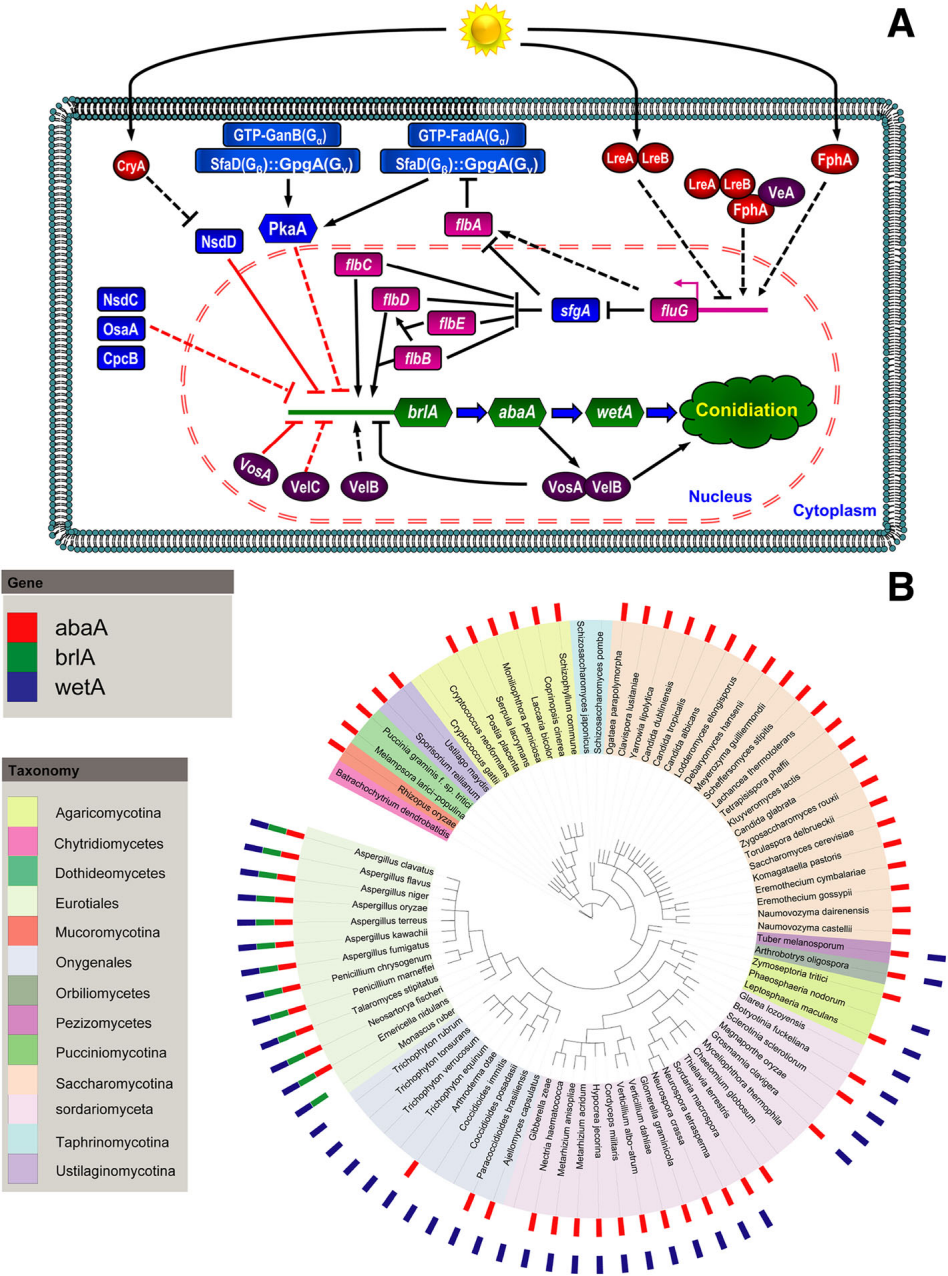
Regulatory pathway of asexual sporulation in *A. nidulans*. **a** Central regulators, upstream activators, negative regulators, velvet regulators, and light-responsive regulators are illustrated by *green*, *light purple*, *blue*, *dark purple*, and *red icons*, respectively. **b** Distribution of central regulators for asexual sporulation in 85 fungi. These fungi are representatives from the phyla Ascomycota, Basidiomycota, Chytridiomycota, and Zygomycota. Their genome protein sequences were searched for homologs of AbaA, BrlA, and WetA by BlastP using sequences of *A. nidulans* AbaA, BrlA, and WetA as queries. Details of these fungi are presented in Central Regulator Strain Information. As shown, BrlA seems to be limited to the Eurotiales group, suggesting a specific role for conidiation in Eurotiales fungi. By contrast, WetA is widely distributed in Pezizomycotina fungi, which suggests a general function for the synthesis of cell wall layers to make conidia mature and impermeable. Surprisingly, AbaA is widespread being found in the phyla Ascomycota, Basidiomycota, and Zygomycota, suggesting that AbaA is involved not only in conidial development, but also has other general functions in fungal development

The regulation of asexual sporulation (conidiation) has been extensively studied in the model fungus *A. nidulans*. Many regulatory genes have been identified and can be divided into central regulators, upstream activators, negative regulators, light-responsive, and *velvet* regulators (Fig. 2a) [35]. A central regulatory pathway (*brlA*, *abaA*, and *wetA*) controls conidiation-specific gene expression and asexual developmental processes [36]. Proper activation of *brlA* requires upstream developmental activators (encoded by *fluG*, *flbA*-*E*) [30] and removal of repression by several negative regulators (including SfgA, VosA, NsdD, and two G-protein signaling pathways; see “Signal transduction pathway” section below) [35, 37]. Light also regulates expression of asexual developmental genes, including *brlA* and upstream activators, with several photoreceptors identified such as one red-light (FphA) and three blue-light receptors [38]. Moreover, *velvet* regulators can interact with each other and with non-*velvet* proteins to control development and sporogenesis [39]. Comparison of the conidiation system of *A. nidulans* to other aspergilli revealed that the regulatory genes are highly conserved, suggesting similar triggers and signaling of the conidiation regulatory pathways in this genus (Additional file 2). The central regulatory pathway (*brlA* → *abaA* → *wetA*) is present in all *Aspergillus* and *Penicillium* genomes, whereas only one or two elements of this pathway were found in the Ascomycotina in general, so making this a central conserved feature of the aspergilli and penicillia (Fig. 2b). Normally, these and other regulatory genes were only present in single copy. However, some absences and occurrences of gene duplication were observed (Additional file 2). Notably, *Monascus ruber* lacks an *abaA* homolog, correlating with a change in conidiation form [40], whereas *A. oryzae* and *A. carbonarius* have two copies of *abaA* and *wetA*. Regarding upstream developmental activators, almost half of the species, including *A. flavus*, *A. fumigatus*, and *A. terreus*, possess two copies of the *fluG* gene, suggesting more differentiated regulation of development in these species. Two copies of the blue light responsive *lreA* and *lreB* transcription factor genes were found in *A. carbonarius* and related experimental work showed this species had the highest levels of asexual sporulation in blue light (relative to white light) of all tested aspergilli (Additional files 2 and 3). Finally, multiple copies of the *fphA* red light receptor were found in some species, although in this case there was no clear correlation with the extent of asexual sporulation under different light treatments (Additional file 4).

#### Sexual reproduction

The apparently asexual nature of the majority of *Aspergillus* species has precluded the use of the sexual cycle as a tool for strain improvement and genetic analysis in them. However, there is accumulating evidence that many such supposedly asexual species have the potential for sexual reproduction if the correct mating partners and environmental conditions to induce sexuality can be identified [32, 33]. The *Aspergillus* species included in this study were typical in that the majority of them have no known sexual cycle (Additional file 5). To investigate whether the asexual species may have a cryptic sexual cycle, the presence and expression of both mating-type (*MAT*) genes conferring sexual identity [41] and a pheromone signaling pathway involved with mating [42] were explored. All of the presumed asexual species were found to contain either a *MAT1-1* alpha gene or *MAT1-2* gene encoding a high-mobility group domain protein, with adjacent gene synteny conserved across all species. This is consistent with heterothallism, involving the presence of either *MAT1-1* or *MAT1-2* idiomorphs [41]. All of the presumed asexual species also contain *ppgA* homologs encoding a pheromone precursor, and *preA* and *preB* homologs encoding pheromone receptors (Additional file 5). These species were grown under conditions conducive to sexual reproduction in the aspergilli and gene expression was monitored by reverse transcriptase polymerase chain reaction (RT)-PCR. It was found that the *MAT1-1* or *MAT1-2*, *ppgA*, *preA*, and *preB* genes were expressed in all of the asexual species in the same way as known sexual species, as shown by the detection of spliced transcripts (although exact expression levels were not determined) (Additional file 6). This suggests that key genes for sexual reproduction may be active in the asexual species. These results are of importance as they suggest the possibility of inducing the sexual cycle in species of biotechnological importance such as *A. niger*, as has recently been achieved for the penicillin producer *P. chrysogenum* [43]. This could accelerate cell-engineering efforts, because sexual crosses would become possible. These results also bring into question the supposed predominance of asexuality in the aspergilli. However, it has to be considered that at least 75 other genes are required for completion of the sexual cycle in the aspergilli [33]. Mutation or lack of expression in any of these genes may be responsible for asexuality if fixed within populations, although previous genome studies found no evidence for gene loss or mutation in over 200 genes implicated in fungal mating in *A. oryzae* and *A. niger* [4, 10].

One other outstanding question concerning the evolution of sex in *Aspergillus* species is whether the ancestral state was homothallism or heterothallism. It has been argued that homothallic fungi are derived from heterothallic ancestors [44]. However, given the predominance of homothallic species among the aspergilli and initial analysis of *MAT* gene organization, it has been suggested that homothallism was ancestral in the aspergilli with subsequent conversion to heterothallism [4, 45]. One key piece of supporting evidence was that heterothallism was presumed to be rare in the aspergilli. However, our findings indicate that many supposedly asexual *Aspergillus* species are in fact heterothallic in nature, and this is true across the wide taxonomic diversity of aspergilli under study (Fig. 1b). This indicates that heterothallism is widespread in the aspergilli, with a conserved organization and synteny of the mating-type (*MAT*) locus observed in the current study. This also contrasts with the variation in organization of *MAT* loci observed in homothallic *Aspergillus* species [4, 46–48]. Overall, our results strongly support the hypothesis that the aspergilli are evolved from a heterothallic ancestor, with homothallic species arising due to divergent forms of *MAT* gene arrangement as previously reported for *Cochliobolus* species [44]. This conclusion is of significance for future attempts to induce sexual reproduction in asexual species and possible genetic manipulation of *MAT* genes to induce sexuality [41].

### Primary carbon metabolism

Fungi are well-known for their ability to exploit a wide range of simple and complex carbon substrates for growth [49, 50]. Differences in their primary metabolism may partly help to explain the diversity of fungal phenotypes. We experimentally and genetically analyzed three traits of fungal primary metabolism in the aspergilli to examine possible links to genome diversity, namely carbon source utilization, presence of alternative oxidases, and organic acid production.

#### Carbon source utilization

**Fig. 3:**
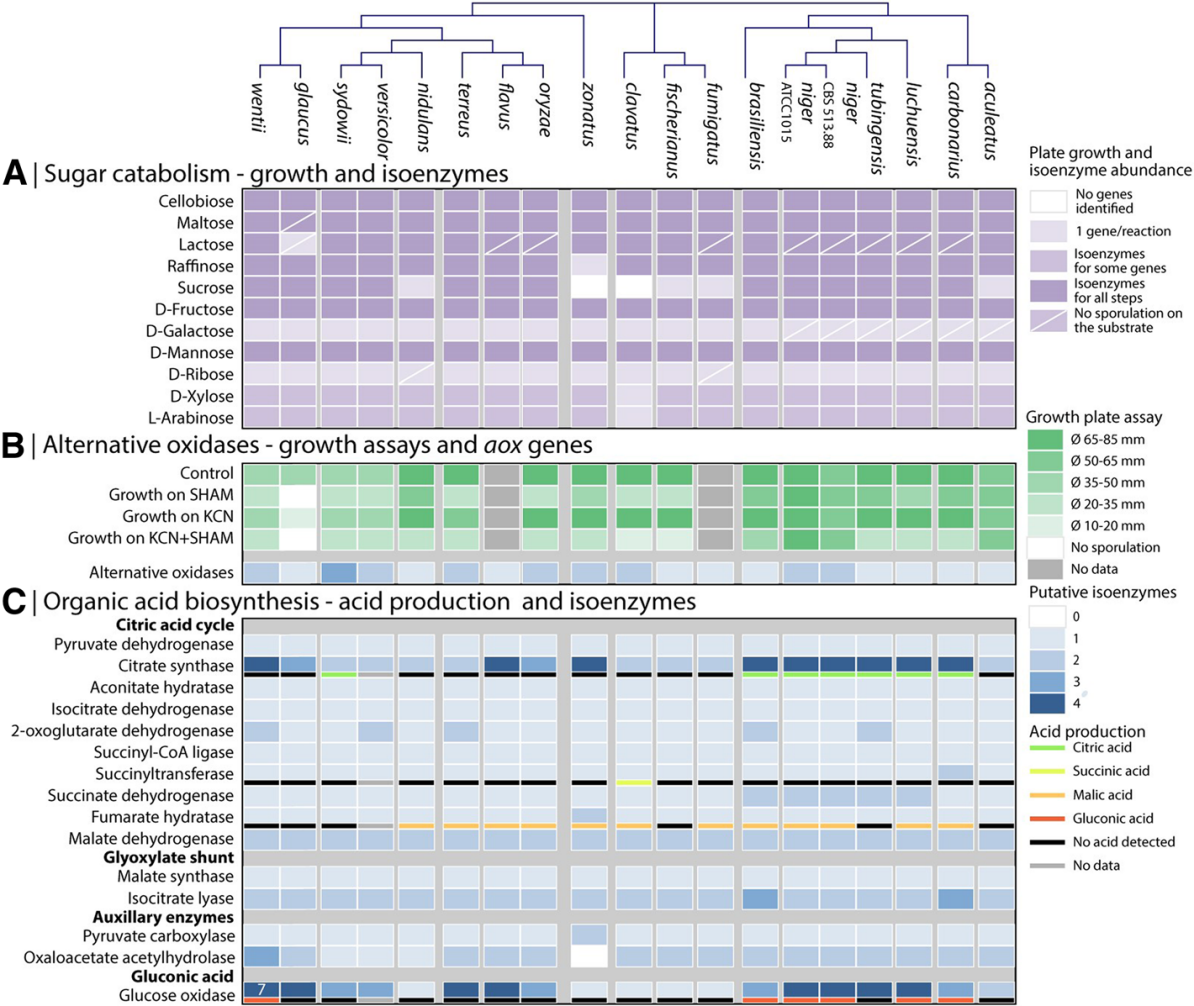
Experimental and in silico analysis of central metabolism. Clustering of the species was performed based on *gpdA* and orthologs from all genomes. **a** Catabolism of 11 sugars correlated to the number of isoenzymes investigated for each of the 11 catabolic pathways. A cross line marks all conditions where not growth was observed. **b** Copy numbers of (putative) alternative oxidases (*aox*) compared to growth assays on plates with agents inhibiting either the standard oxidative phosphorylation (KCN) or Aox (SHAM). **c** Correlation of putative isoenzymes for activities involved in organic acid formation and produced organic acids

The genomes of 19 aspergilli were analyzed for copy numbers of carbon catabolic genes and compared for growth on 11 different monomeric and dimeric carbon sources (Fig. 3a, Additional files 6 and 7). A nearly complete correlation was found between the presence of genes predicted to encode catabolic enzymes and the ability to grow on a specific carbon source. Exceptions to this were the inability of *A. niger* to grow on D-galactose and lactose, but it is known that the relevant transport and germination triggers for these carbon sources are not active in *A. niger* spores [51–53] (Fig. 3a). Furthermore, *A. clavatus* and *A. zonatus* can grow on sucrose despite having no apparent invertases in their genomes. This could be due to genes missing in the assembly, the activity of α-glucosidases [54], non-specific enzymes, or a so far unknown type of invertase possibly found outside the standard GH32 Carbohydrate Active enZyme (CAZy) invertase family.

In general, extra copies of a catabolic pathway, or steps thereof, did not confer more efficient carbon utilization under the conditions tested, possibly due to differential expression or altered substrate specificity of the paralogs. It was further observed that gene copy number of specific enzymes was generally correlated with phylogenetic relationships, in contrast to the increased levels of genetic diversity and horizontal gene transfer observed for secondary metabolism among closely related species (see below).

#### Alternative oxidase

A characteristic feature of fungal mitochondria is the presence of an additional terminal oxidase, the alternative oxidase (Aox), alongside the cytochrome-dependent one. Aox catalyzes the reduction of oxygen to water, bypassing respiratory complexes III and IV of the cytochrome-mediated electron transport chain. All examined species showed the presence of at least one alternative oxidase (*aox*) gene (Fig. 3b). Growth experiments confirmed that all *Aspergillus* species grew well in the presence of potassium cyanide (KCN), which inhibits the cytochrome pathway (Additional file 8). Addition of salicylhydroxamic acid (SHAM), which inhibits Aox, as well as KCN stunted or eliminated growth of nearly all species except *A. niger* and *A. aculeatus*. In general, the section *Nigri* exhibited higher tolerance to SHAM and KCN and grew faster than other aspergilli. As with the primary metabolism above, this increased growth could not be explained by changes in gene copy numbers as the *Nigri* had similar or lower *aox* copy numbers than other species (Fig. 3b).

#### Organic acid production

Organic acid production by *A. niger* is a process of major industrial importance. We examined the number of putative isoenzymes associated with citric acid and gluconic acid production in each of the 19 genomes and measured the concentration of relevant organic acids produced on three different growth media (Fig. 3c, Additional file 9). This revealed a close link between the number of isoenzymes and phylogeny, most markedly for the section *Nigri*, in which a series of extra isoenzymes relevant to citrate and gluconic acid production was found, correlating with increased acid production (Additional files 9A and B). This is in accord with this group being prolific producers of these two organic acids and suggests the evolution of specialized enzymes for this purpose. Indeed, transcriptome analysis of *A. niger*, *A. oryzae*, *A. fumigatus*, and *A. nidulans* showed that orthologs of citrate synthase, pyruvate dehydrogenase, isocitrate dehydrogenase, and malate dehydrogenase were highly expressed in *A. niger* relative to the other species (Additional file 10C), consistent with metabolic specialization.

### Plant biomass degradation

#### Genomic comparison

Several aspergilli have a long history of use as producers of plant polysaccharide modifying and degrading enzymes [55, 56]. Their broad ability to degrade these substrates helps to explain their presence in many different habitats, employing what has been considered a generalist lifestyle with respect to carbon utilization. This means that they do not specialize in any particular plant component, but have the ability to degrade all plant biomass polysaccharides [57, 58]. However, this has so far only been investigated for a relatively small number of species. Therefore, in this study we evaluated plant biomass degrading ability across a larger set of *Aspergillus* species and compared this ability to other ascomycete fungi. Comparison of the number of genes per CAZy family involved in plant biomass degradation revealed strong differences among the species investigated (Table 2, Additional file 10), although species from the same section generally contained similar numbers of genes. This comparison was based on the family (and subfamily where possible) designations set by the CAZy database (www.cazy.org) [59]. Exceptions to this were, for example, the lower number of (hemi-) cellulases for *A. carbonarius* compared to other species of the section *Nigri* and that *A. versicolor* is particularly enriched in inulinases compared to other species of the section *Nidulantes* (Table 2). Interestingly, the two most established enzyme producers of this genus, *A. niger* and *A. oryzae*, do not contain the highest number of genes required for degradation of any of the polysaccharides, with the exception of pectin for which the highest number was found in the *A. oryzae* genome (Table 2). The itaconic acid producer *A. terreus* has the highest number of putative cellulases and xyloglucanases, while *A. versicolor* and *A. nidulans* contain the highest numbers of putative xylanases and mannanases, respectively. Gene numbers related to the degradation of the storage polysaccharides starch and inulin are, in general, higher in the sister genera of *Aspergillus* (e.g. *Penicillium*).

**Table 2:**
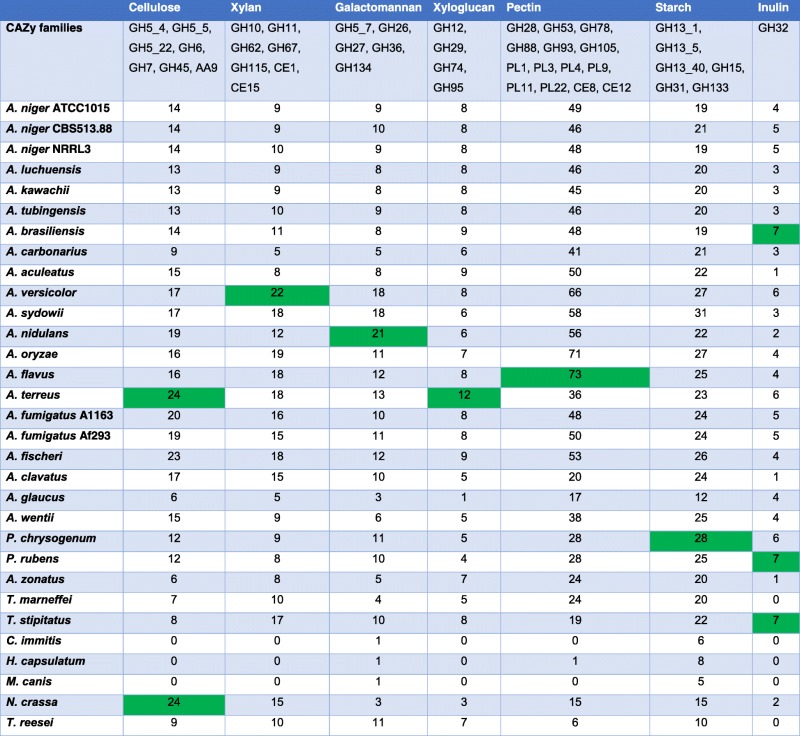
Gene numbers related to degradation of different plant-based polysaccharides detected in genomes of selected ascomycete fungi

Only families that can be assigned to a specific polysaccharide, based on currently available data, are included. Highest numbers for any of the polysaccharides are highlighted in green

When comparing specific enzymes, highly variable gene numbers were observed for GH5_7 and GH26 endomannanases, GH32 endoinulinases, GH28 endorhamnogalacturonases, GH43 α-L-arabinofuranosidases, and GH115 α-glucuronidases (Additional file 11). Only some species contain genes encoding CE15 glucuronoyl esterases, GH45 endoglucanases, and PL11 rhamnogalacturonan lyases. While most of the feruloyl esterase encoding genes are included in the CAZy database, comparison of genes encoding these enzymes also revealed high variability, confirming the considerable diversity in feruloyl esterase activities recently reported for a smaller set of aspergilli [60]. This diversity is in part due to the absence of specific orthologs in some species, resulting in differences in the overall gene set per species (Additional file 11).

High variability was also observed in the number of genes encoding oxidative enzymes in the genomes analyzed (Additional file 10), which can also be involved in plant biomass degradation. Numbers in families AA1 (containing laccase), AA3_1 (cellobiose dehydrogenase), AA7 (containing vanillyl alcohol oxidase), and AA9 (lytic polysaccharide monooxygenases) are in the ranges of 5–18, 1–4, 0–7, and 3–12, respectively, but in general the variation within each section is relatively low.

#### Correlation of gene content to growth and enzyme production

**Fig. 4:**
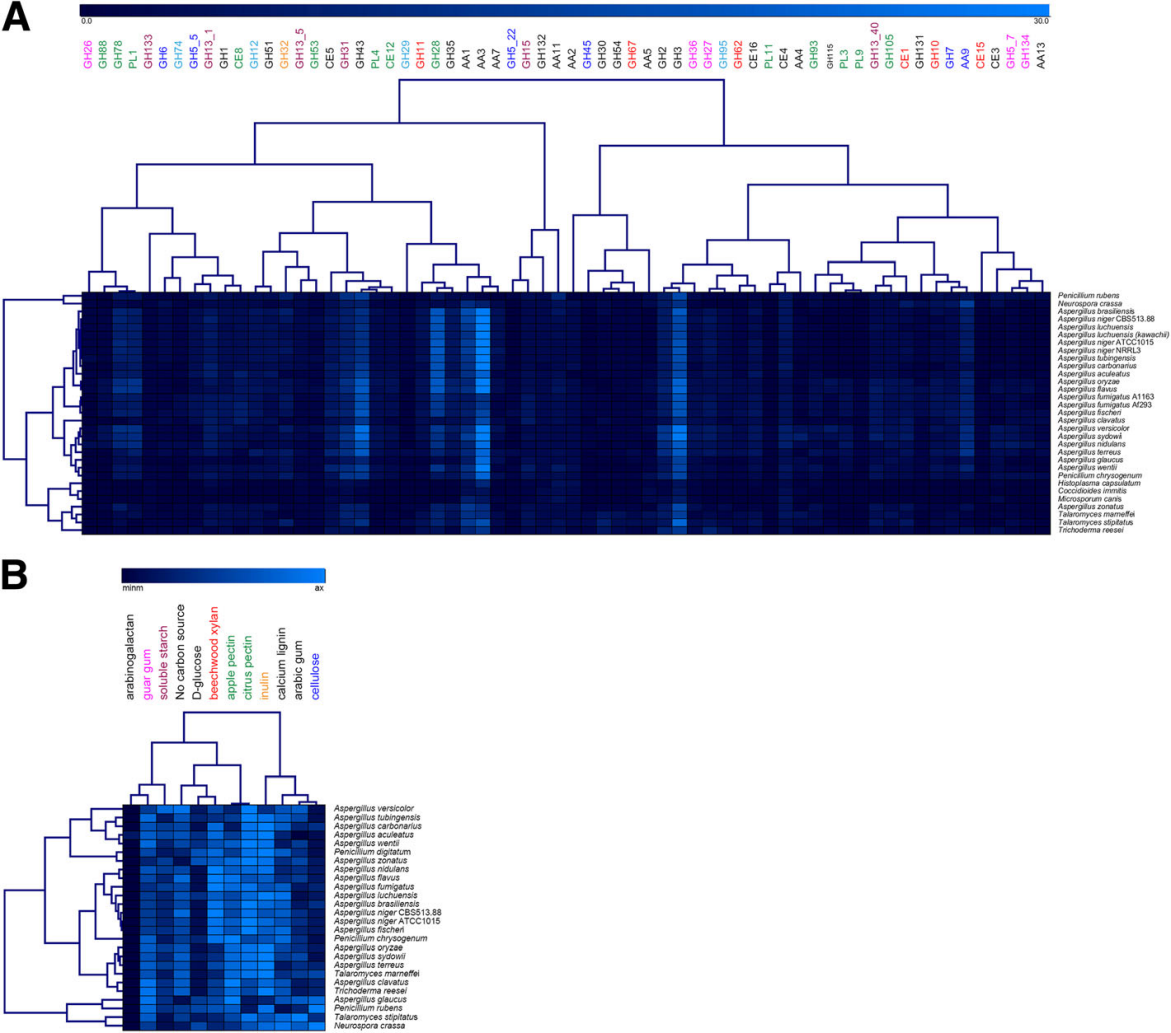
HCL (**a**) the number of genes per CAZy family related to plant biomass degradation in the tested genomes and (**b**) the relative growth of the tested species on polymeric substrates. *Color coding* in (**a**) refers to the polysaccharide these families act on. Black families either contain multiple activities or are active on multiple polysaccharides. For the other colors: *pink* = guar gum (galactomannan), *purple* = starch, *red* = xylan, *green* = pectin, *orange* = inulin, *dark blue* = cellulose, *pale blue* = xyloglucan

To assess any correlation between CAZy gene content and growth, strain profiling was performed by determining growth on 33 carbon sources which contained plant-related monosaccharides, oligosaccharaides, and polysaccharides as well as crude plant-based substrates, and no-carbon source and casein as controls (Additional file 8). Full profiles can be found at [61]. A hierarchical clustering (HCL) correlation analysis was performed on the number of genes per CAZy family for the tested species. Figure 4a shows that overall the evolution of CAZy families is similar to the taxonomic relationship of the species (compare to Fig. 1a). In this analysis, families that become more or less abundant simultaneously group together. No clear overall clustering of CAZy families related to the same substrate could be observed. This is likely due to the presence of multiple polymers in any plant biomass, requiring the production of enzymes acting on different substrates. In addition, commercial substrates used are unlikely to be identical to those encountered in nature and may therefore limit the observed specificity. In some cases, two to three enzyme families related to the same substrate did cluster, such as GH88/GH78/PL1 and PL3/PL9 (pectin), GH7/AA9 (cellulose), and GH27/GH36 and GH5_7/GH134 (galactomannan). In contrast, when the ability of these fungi to grow on polysaccharides (Additional file 8) was quantified and used for HCL, the taxonomic organization was largely lost (Fig. 4b). This suggests that despite conserved CAZome evolution, the ability of an individual species to degrade a specific substrate is dependent on other factors such as the regulation of gene expression or the transport of monomers. Therefore, as observed recently for eight aspergilli [57], no clear correlation between CAZome, taxonomy, and enzyme sets produced on plant biomass substrates could be detected. A possible explanation for this is that under laboratory conditions only complete disappearance of a CAZy family would result in loss of function. However, the persistence of the CAZy genes clearly indicates an evolutionary pressure to maintain them in the genomes, which could be due to the more complex natural environment. A better understanding of fungal biotopes, including the actual components of the available substrates they consume in nature, will likely reveal a better correlation between genome content and carbohydrate preference of each species.

To assess the impact on growth arising from differences in the number of genes encoding oxidative enzymes, 20 aspergilli were grown in minimal medium (MM) with 1% cotton seed hulls over 14 days and were assayed for laccase and cellobiose dehydrogenase activities (Additional file 12). The highest laccase activity was observed for *A. fischeri* and low activities were detected for *A. oryzae*, *A. wentii*, and *A. versicolor*. No laccase activity could be detected for 11 of the aspergilli. The highest cellobiose dehydrogenase levels were measured for *A. fumigatus*, *A. wentii*, *A. tubingensis*, and *A. terreus*. No cellobiose dehydrogenase activity was detected for five aspergilli. Thus, there was no clear correlation between these observed enzyme activities and the number of genes in the respective genomes (Additional file 10).

**Fig. 5:**
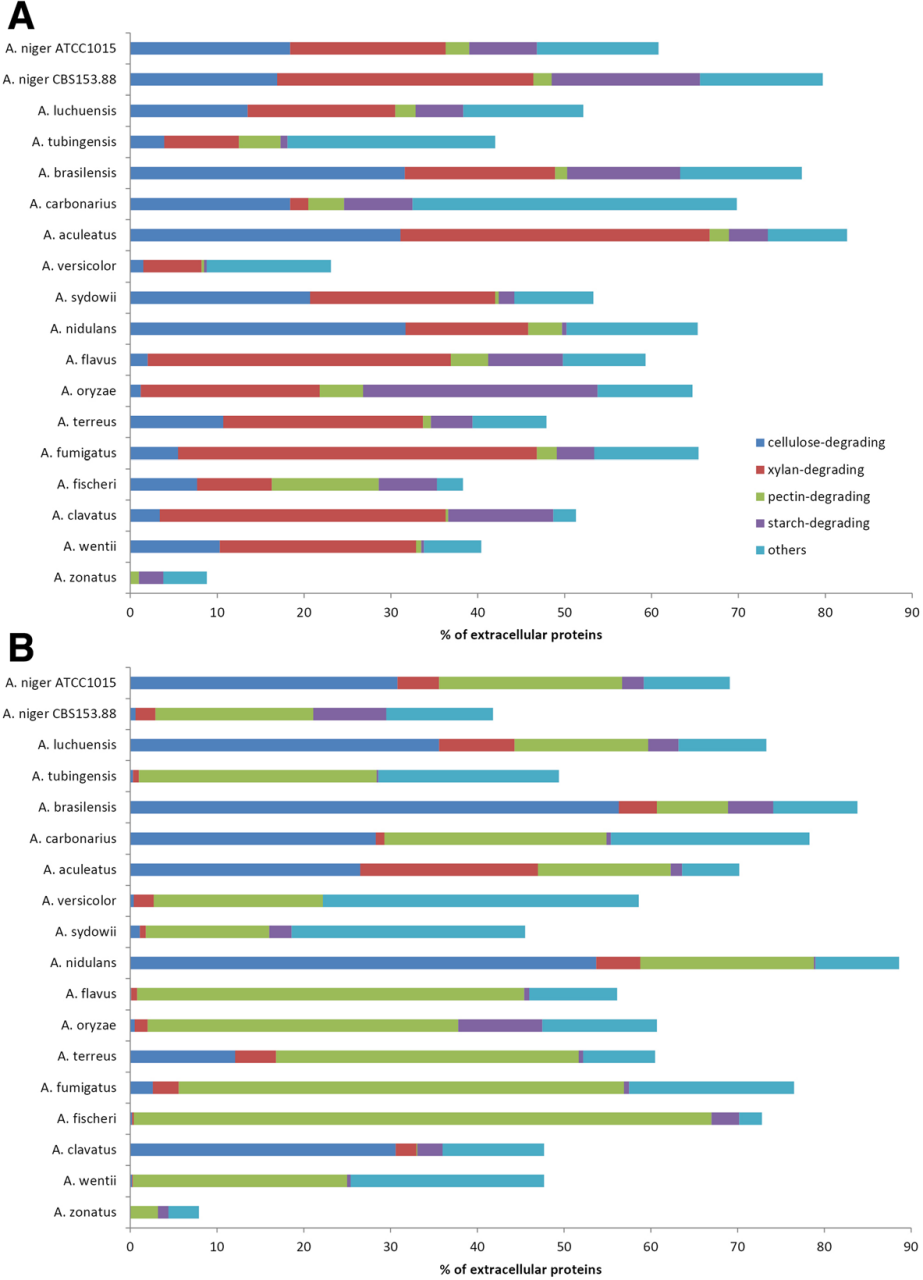
Comparative proteomics of aspergilli during growth on wheat bran (**a**) and sugar beet pulp (**b**). Results are summarized by the polysaccharide that the enzymes act upon. Only CAZy families specific for one polysaccharide are included in the comparison. Cellulose-active enzymes: GH6, GH7, GH12, GH45, GH74, AA9; xylan-active enzymes: CE1, CE15, GH10, GH11, GH62, GH67, GH115; pectin-active enzymes: CE8, CE12, GH28, GH35, GH51, GH53, GH54, GH78, GH88, GH93, GH105, PL1, PL3, PL4, PL9, PL11; starch-active enzymes: GH13, GH15; other (CAZy families with multiple activities or minor activities on the substrates): CE16, GH1, GH2, GH3, GH5, GH26, GH27, GH31, GH32, GH36, GH43, GH95. Extracellular proteins following trypsin digestion were analyzed by LC-MS/MS using a LTQ-Orbitrap Velos mass analyzer (Thermo-Fisher). Quantification was based on MS precursor ion signal. Extracted ion chromatograms were used to determine the peptide area value associated to each identified precursor ion. A protein area value was calculated as the average of the three most intense, distinct peptides assigned to a protein. The amounts of proteins associated with each enzyme activity were expressed as percentage of the amount of total extracellular proteins present in each culture condition

Recently, Benoit et al. compared the production of plant biomass degrading enzymes by eight aspergilli during growth on wheat bran or sugar beet pulp [57]. This work was extended in the present study to include the newly sequenced species, using the same culture conditions and data analysis as in the previous study. These substrates provide useful comparative datasets because both contain cellulose, but differ in that wheat bran is rich in xylan, while sugar beet pulp contains large amounts of pectin and xyloglucan. The results demonstrated a large variation in enzyme production between the aspergilli, even for species within the same section (Fig. 5). Most species produced predominantly cellulose-active and xylan-active enzymes during growth on wheat bran, but the ratio between these two enzyme groups varied strongly in some species (Fig. 5a). Some unusual profiles were also observed, such as the high relative amount of different pectinases produced by *A. fischeri*, the low amount of xylanases for *A. carbonarius*, and the high amount of amylases for *A. oryzae*. On sugar beet pulp, nearly all species produced high amounts of pectinases, appropriate to the composition of the substrate (Fig. 5b). An exception to this was *A. clavatus*, where genome analysis had already revealed a strong reduction in genes encoding pectin-active enzymes [57]. *A. clavatus* compensates by high production of cellulases, whereas cellulases are almost absent in the profiles of several other species (e.g. *A. wentii*, *A. fischeri*, *A. flavus*, *A. tubingensis*). The high production of xylanases by *A. aculeatus* and to a lesser extent by *A. luchuensis* was also unexpected.

The variation in detected enzymes in the proteomic data of the species investigated here far exceeded that which could be explained by genome variation, consistent with previous results from the first eight *Aspergillus* species to be genome sequenced [57]. This suggests that the observed variation is mainly caused by regulatory changes, rather than simply by gene copy number. Since all species contain the same set of conserved regulators for cellulase, xylan, and pectin degradation (data not shown), they presumably have a different fine-tuning of the regulatory system. This could be due to a different range of target genes for their regulators or different functioning of them, as was recently demonstrated for the major (hemi-) cellulolytic regulator XlnR [62]. The exoproteome analysis reported here confirms our recent observation [57] that the enzyme cocktails produced by these fungi in response to the same plant biomass are highly different and will therefore also have different efficiencies in saccharification of plant-derived biomass.

### Secondary metabolism

The ability to produce secondary metabolites (SM) is an important feature of many fungi and certainly of the aspergilli and related fungi. These metabolites are involved in many processes in nature, such as communication between fungi and competition for nutrients and can be pathogenicity factors. Some of these metabolites have become widely used as antibiotics. In this section, we highlight the diversity of SM production in the aspergilli and the role of cytochrome P450 (CYP) genes in secondary metabolism.

#### Secondary metabolite biosynthesis gene clusters in the aspergilli

Polyketide synthases (PKSs), non-ribosomal peptide synthetases (NRPSs), or dimethylallyl tryptophan transferases (DMATs) are involved in the biosynthesis of the vast majority of SM products [63, 64]. The PKS and NRPS genes are frequently clustered together with other pathway genes, such as DMAT genes, involved with processing of the initial product to form the final SM, thereby forming a linked gene cluster. The high degree of conservation of these proteins and their specific domain structure allow reliable in silico prediction of their genes. Thus, the *Aspergillus* genomes were searched to gain insights into their evolution and potential to produce SMs. The number of individual PKS, NRPS, and DMAT genes was found to vary considerably from 21 in *A. glaucus* to 66 in *A. tubingensis* and *A. versicolor* (Table 3). These large variations do not correlate with genome size, suggestsing that the number of SM genes and their associated clusters depends on the lifestyle and the corresponding requirements of each species.

**Table 3:**
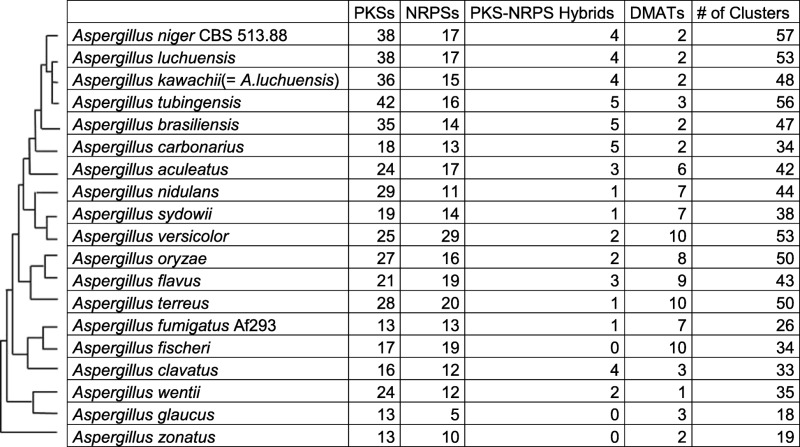
Number of predicted SM biosynthesis genes and number of associated clusters in the genomes

*Aspergillus* species were ordered according to their phylogenetic relationships (see Fig. 1)

To date about 50 non-redundant SM gene clusters have been experimentally characterized in the aspergilli. We scanned the 19 genomes for regions syntenic with these known clusters to examine their distribution and thereby determine the potential of the various species for production of SMs. Thirty-six clusters appeared to be conserved with respect to gene content and order, with 23 clusters conserved in more than one genome (Table 4). By contrast, 12 clusters showed no conservation of gene content and order (Table 4). Interestingly, in total, we defined 44 unique PKS, NRPS, or DMAT genes that have no orthologs across all the analyzed 19 species.

**Table 4:**
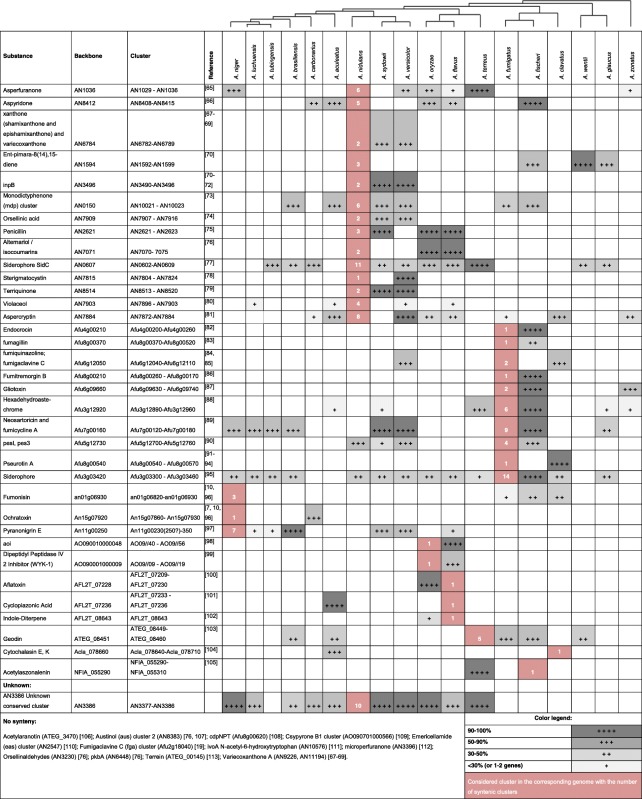
Conservation of experimentally characterized clusters in the 18 *Aspergillus* species

The species are ordered according to their phylogenetic relationships. The level of synteny (percentage of syntenic cluster genes) is reflected by the color legend. Clusters are based on previous studies [65–113]

**Fig. 6:**
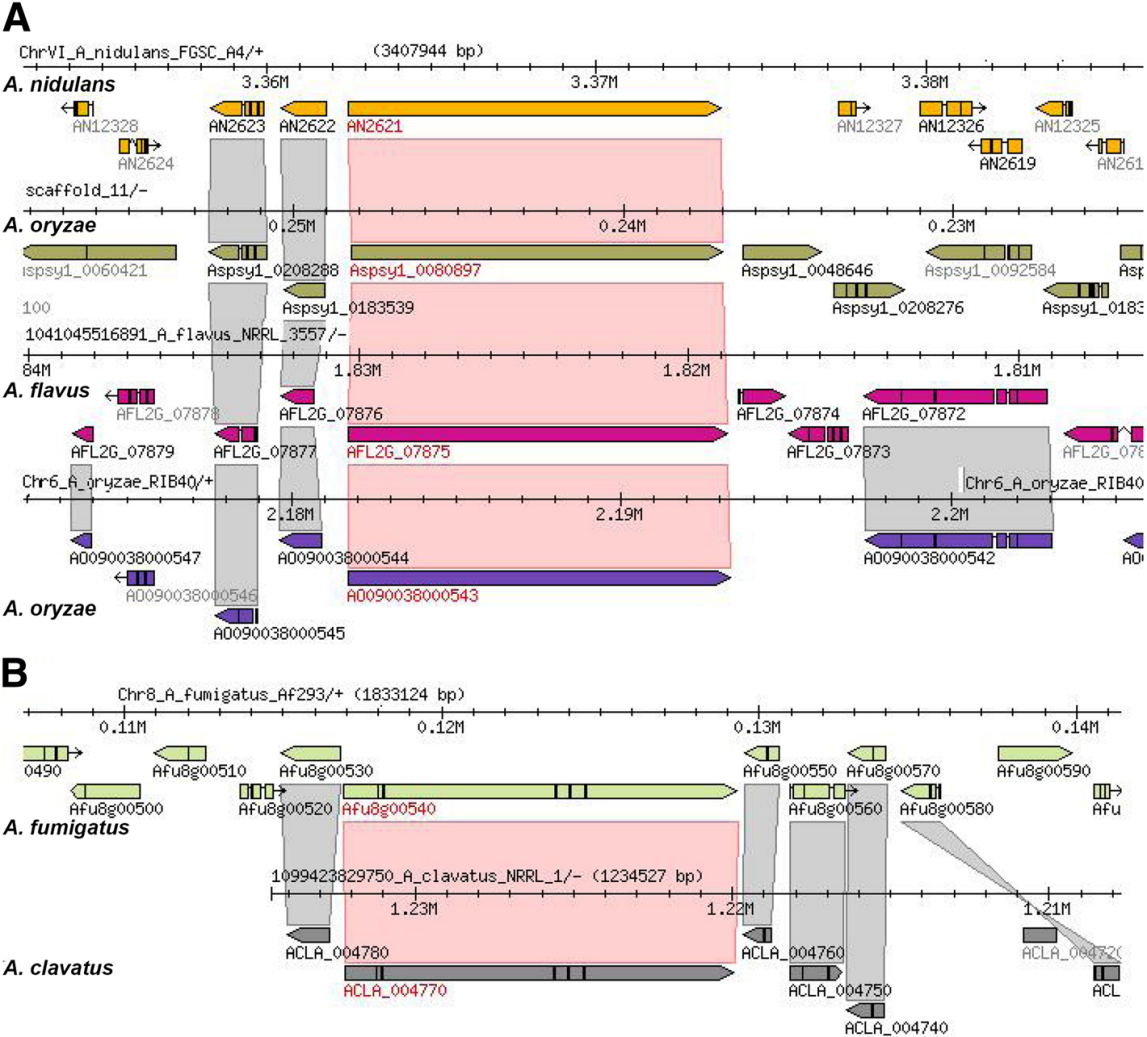
Conservation of penicillin (**a**) and pseurotin (**b**) biosynthesis gene clusters in *Aspergillus* species

The degree of similarity between clusters from different species can provide an indication of potential similarities in the products that they synthesize, with clusters having very high similarity (90–100%) normally coding for the same SM. Genome data from the present study therefore allowed us to predict the production of certain SMs. This can be exemplified for the production of pseurotin and penicillin. Pseurotin was previously described for *A. fumigatus* [64, 91–93], *A. clavatus* [114, 115], *A. oryzae* [116], *A. leporis*, and *A. nomius* [117] but so far not for other *Aspergillus* species. The gene cluster in *A. fumigatus* was already known, but here we identified the conserved pseurotin gene cluster in the *A. clavatus* genome. There was no indication of a pseurotin gene cluster in *A. oryzae*, although its production was reported for this species. However, the particular strain reported to produce pseurotin is not available and may have been misidentified. In another example, analysis of the penicillin gene cluster known to be present in *A. nidulans* [75], *A. oryzae* [118], and *A. flavus* revealed a single additional species, *A. sydowii*, where this cluster is well conserved indicating the potential for penicillin production (Fig. 6).

In addition, we found examples of clusters with partially conserved synteny (50–90%; Table 4). The SM products of such clusters can be expected to be similar but not identical. For instance, fumiquinazolins were observed in *A. fumigatus* [119], whereas in *A. versicolor* and *A. clavatus* there are cottoquinazolins [120] and tryptoquivalins [121], respectively, which is in agreement with the partial conservation and synteny of the corresponding clusters (Table 4). Similarly, geodin produced by *A. terreus* is related to sulochrins produced by *A. fumigatus* (3-O-methylsulochrin) and *A. wentii* (sulochrin and isosulochrin), consistent with moderate conservation of the respective gene clusters (Table 4).

In *A. nidulans*, three gene clusters (AN6784, AN9226, and AN11194) are responsible for the biosynthesis of xanthone (shamixanthone and epishamixanthone) and variecoxanthone. It was observed that the shamixanthone cluster (AN6784) is reasonably conserved, with five of nine *A. nidulans* cluster genes being syntenic in *A. sydowii* and *A. versicolor* (Table 4). However, the genes for the other clusters are unique to *A. nidulans*, which is known to produce a much more varied profile of these compounds than *A. sydowii* and *A. versicolor*. Meanwhile, the lack of production of particular SMs by some strains and/or isolates was often confirmed by the genome data. *Aspergillus versicolor* and *A. nidulans* produce sterigmatocystin [122], whereas the closely related *A. sydowii* CBS 593.65 does not. This can now be explained by the complete lack of the corresponding gene cluster in *A. sydowii*, whereas the sterigmatocystin cluster is perfectly conserved between *A. versicolor* and *A. nidulans* (Table 4)*.* Thus, our bioinformatic analysis overall supports and provides a genetic basis for previous experimental data.

Analysis of the genome data also allowed us to predict gene clusters that might be responsible for the biosynthesis of experimentally detected SMs for which the genetic basis had previously been unknown. For example, the three black *Aspergillus* strains, *A. brasiliensis* CBS101740, *A. luchuensis* CBS106.47, and *A. tubingensis* CBS134.48, all produce naptho-gamma-pyrones (Table 4) [123]. The gene cluster for the related neosartoricin/fumicycline of *A. fumigatus* and *A. fischeri* has previously been characterized and may be analogous to the naptho-gamma-pyrone cluster in species of the section *Nigri*, given that both gene clusters occur in the same conserved syntenic region across all these species.

Finally, the analysis led to the discovery of previously unknown, but well conserved, clusters such as that with an AN3386 backbone (Table 4), providing exciting leads for future cluster analysis.

#### CYPomes and supporting cytochrome P450 reductases

CYP genes are also of interest for secondary metabolism. More than 2300 CYP sequences (PF00067) were identified in the 19 aspergilli examined (data not shown). These numbers are comparable with analysis of the CYPome for *A. nidulans* in the previous CADRE genome annotation [124]. Some of the CYP families were further analyzed in the present study relating to their possible use as antifungals and roles in fungal secondary metabolism, namely: CYP51 (sterol 14α-demethylase) [125], CYP61 (22-sterol desaturase) [126], CYP56 (dityrosine synthesis) [127], CYP53 (benzoate 4-hydroxylase) [128], CYP630 (oleic acid ω-hydroxylase) [129], Ppo (fatty acid oxygenases or oxylipin generating dioxygenases) [130], and a supporting enzyme CYP reductase (CPR) as well as CYP505, CYP-CPR fusion protein (fatty acid hydroxylase) [131].

**Fig. 7:**
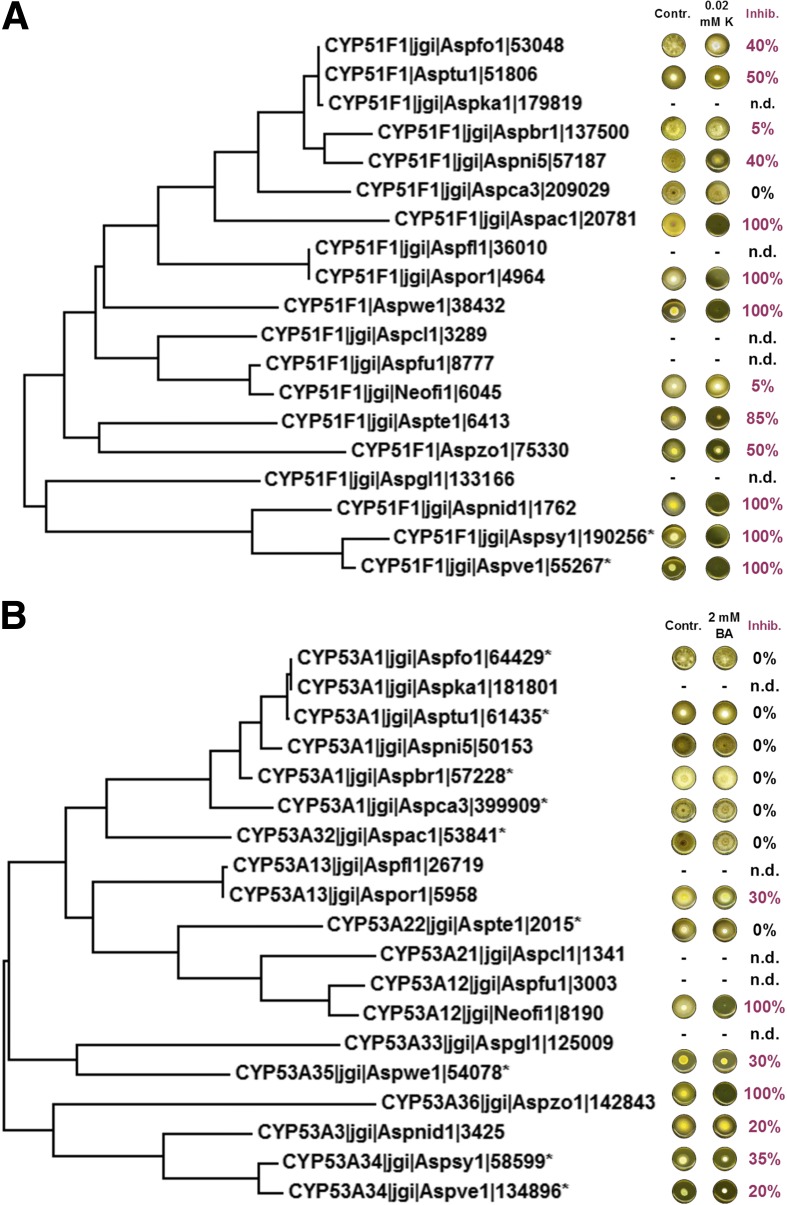
Molecular phylogenetic tree of (**a**) CYP51F1 (CYP51B) family and inhibition of growth on MBFA agar supplemented by 0.02 mM ketoconazole (K) and (**b**) CYP53 family and inhibition of growth on 2 mM benzoic acid (BA). Inhibition of growth (Inhib.) as per cent of the control without supplementation (Contr.) is shown in relation to phylogenetic trees, with growth culture plates shown to the *right-hand side* of the accompanying tree branch. Key to *Aspergillus* species: Aspfo = *A. luchuensis* (formerly *A. foetidus*); Asptu = *A. tubingensis*; Aspka = *A. luchuensis* (*A. kawachii*); Aspbr = *A. brasiliensis*; Aspni = *A. nidulans*; Aspca = *A. carbonarius*; Aspac = *A. aculeatus*; Aspfl = *A. flavus*; Aspor = *A. oryzae*; Aspwe = *A. wentii*; Aspcl; = *A. clavatus*; Aspfu = *A. fumigatus*; Neofi = *A. fischeri*; Aster = *A. terreus*; Aszo = *A. zonatus*; Asgl = *A. glaucus*; Aspsy = *A. sydowii*; Aspve = *A. versicolor*

Most aspergilli were found to contain two copies of CYP51, whereas only a single copy of CYP53 was present in all species. The fact that CYP53 is unique to filamentous fungi makes it an attractive candidate drug target against fungal pathogens [131]. A series of inhibition experiments with ketoconazole (for CYP51) and benzoate (for CYP53) revealed complementary inhibition patterns for these CYPs, suggesting that simultaneous treatment with both drugs may be effective (Fig. 7). CYP61 was always present as a single ortholog, while surprisingly, CYP56 was not found in 11 species (Additional file 13A). As a part of an evolutionarily conserved gene cluster including CYP630, in most species a paralog, CPR2, was identified for the conserved main electron donor CPR1 (Additional file 13B). This cluster may be of importance when the main ß-oxidation pathway is overwhelmed by other substrates such as host terpenoid compounds [129].

One to four copies of CYP-CPR fusion proteins were found in *Aspergillus* species. The presence of multiple copies may allow each particular fusion protein to function in a specific SM pathway. For the second type of fusion protein, haem peroxidase-CYP, orthologs of PpoA (CYP6001A subfamily) and PpoC (CYP6001C) were found in regions of colinearity in all genomes (except for *A. zonatus* and *A. kawachii*, respectively), implying conservation of biological function. Additional copies of haem peroxidase-CYP fusion proteins were present for some genomes in three different subgroups with homology either to PpoB from *A. nidulans*, PpoD from *A. niger* and *A. flavus*, or PpoB from *A. fumigatus* and *A. flavus* (Additional file 14). Specifically, the CYP6002A subfamily consists of PpoB from *A. nidulans* clustered together with orthologs from *A. sydowii*, *A. versicolor*, *A. wentii*, *A. oryzae*, *A. terreus*, *A. clavatus*, and *A. fischeri*. The CYP6003B subfamily consists of PpoD from *A. niger* and *A. flavus* and orthologs from *A. glaucus*, *A. oryzae*, *A. terreus*, *A. tubingensis*, *A. luchuensi*s, *A. kawachii*, and *A. brasilensis*. Finally, the CYP6001C subfamily comprises PpoB from *A. fumigatus* and *A. flavus* together with ortholgs from *A. glaucus*, *A. terreus*, *A. oryzae*, and *A. fischeri*.

### Stress response

Filamentous fungi are exposed to a complex set of abiotic or biotic stresses in natural environments as well as in industrial processes where they are used to produce various biotechnological products. Effective stress perception and signal transduction mechanisms are necessary for adaptation and survival regardless of whether rapid or slow adaptation responses are required.

The complex and robust stress defense systems of the aspergilli [132] were investigated using a wide array of stress physiology experimental and comparative genomics tools.

The stress tolerances of the aspergilli were screened under stress conditions including illumination, oxidative (H_2_O_2_, menadione), hyperosmotic (sorbitol, NaCl), heavy metal (Cd(II)), and cell wall integrity (calcofluor white, Congo Red) stress. The aspergilli were also exposed to selected antimycotics that interfere with cell wall (caspofungin) and cell membrane (voriconazole, amphotericin B) composition and structures. Stress sensitivities were tested in both liquid and surface cultures and the complete stress dataset with photographs is also available in the Fungal Stress Database [133]. A more extensive description of the data can be found in Additional files 3, 15, 16, and 17.

#### Illumination *stress*

**Fig. 8:**
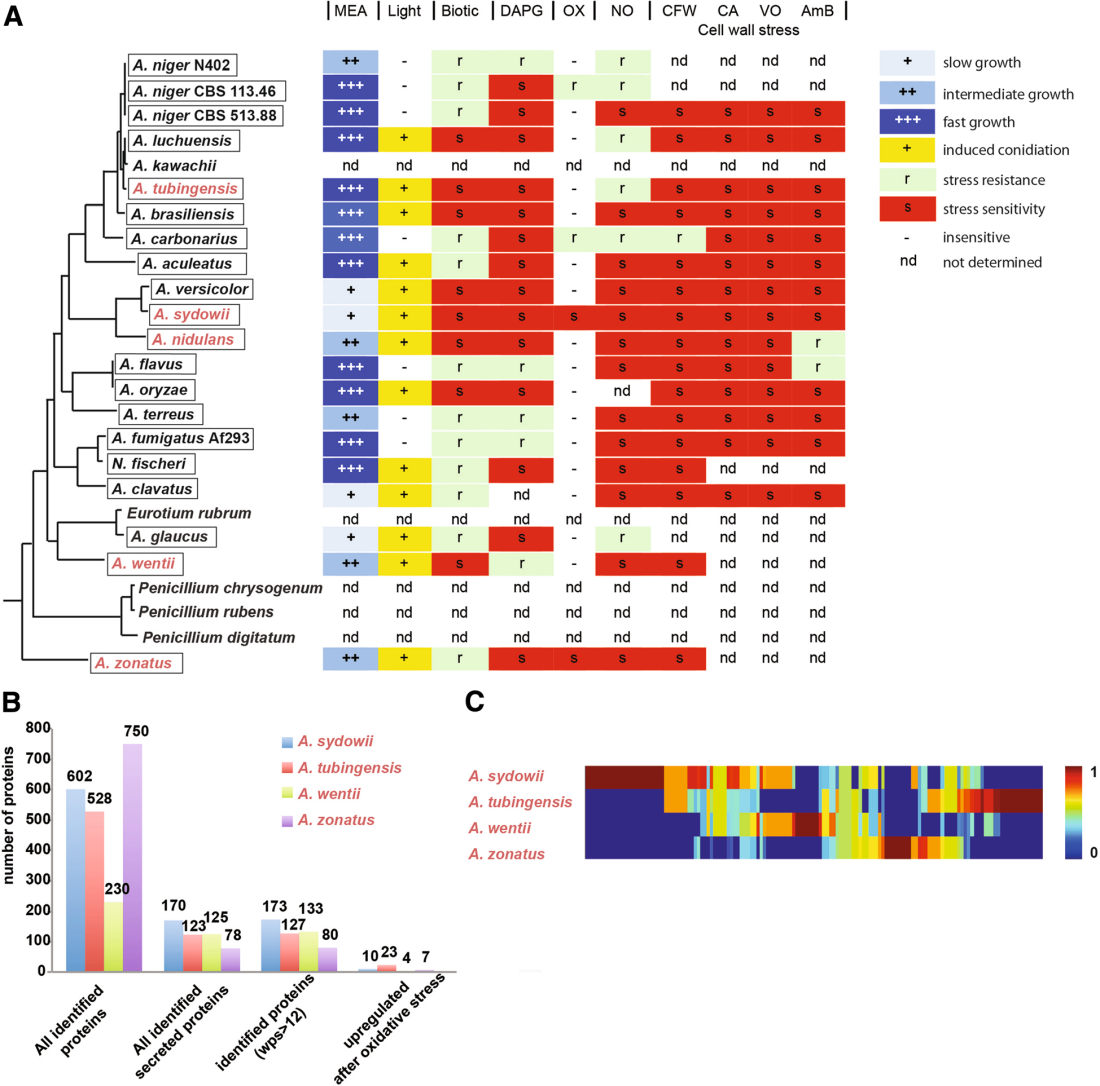
**a** Phylogenetic tree of *Aspergillus* species and summarized phenotypes from this study (modified from [308]). All *Aspergillus* species were grown on malt extract agar (MEA) (*blue*) at 30 °C. *Yellow squares* stand for induced conidiation in white light. Abiotic and biotic stress determinants were colored in *green* for resistance (r), *red* for sensitive (s), *no color* for insensitive (-) and not determined (nd) different abiotic and biotic stressors: 2,4-diacetylphloroglucinol (DAPG), Hydrogen peroxide (OX), nitric oxide (NO), calcofluor white (CFW), caspofungin (CA), voriconazole (VO), amphotericin B (AmB), and *Aspergillus*-*Pseudomonas fluorescens* co-cultivation (Biotic). Species used in this study are framed in *black*. **b** Qualitative descriptive statistics of the exoproteome of four selected *Aspergillus* strains. Exoproteomes were enriched from filtered culture supernatants (*Aspergillus* MM, 30 °C) and analyzed by shotgun proteomics (LC-MS). Proteins were identified using draft genomic databases of the respective strains. For prediction of subcellular localization of the proteins the programs “SignaIP” and “WoLF PSORT” were used. For further analysis, sequences with an extracellular score >12 (WoLF PSORT) were defined as putatively secreted proteins (Additional file 18). Proteins with higher spectral count values after 0.2 mM hydrogen peroxide treatment (“upregulated after oxidative stress”) were selected statistically using MARVIS Filter (s/l > 0.5). **c** Exoproteomic heterogeneity in the genus *Aspergillus*. Comparative MARVIS cluster analysis of PFAM domains predicted in identified exoproteins (WoLF PSORT columns in bar chart B) of four selected *Aspergillus* species (WoLF PSORT: extracellular score >12) (Additional file 19). *Colors* represent the normalized frequency of occurrence of PFAM-domains in the respective exoproteome. Indicated by the color scale on the *right*, the color scale ranges from *blue* (no domain) via *green* (few domains) to *red* (more domains). Each *column* of the cluster image resembles one PFAM-domain. The proteome data is available at http://wwwuser.gwdg.de/~hkusch/GBIO_DeVries/

Light-induced conidiation is common for most of the analyzed aspergilli. While light receptors and regulators were found to be conserved in most species (Additional file 17), rewiring of the response to the receptors and signal transduction pathways appears to have occurred in many species with respect to conidiation at different wavelengths. For example, blue or red light induced conidiation occurs in certain species but not others, while some species (e.g. *A. versicolor*) required specifically white light to induce sporulation. Interestingly, the industrially important black aspergilli and the opportunistic pathogens *A. fumigatus* and *A. flavus* showed little response to illumination (Fig. 8, Additional file 17).

#### Oxidative stress

**Fig. 9:**
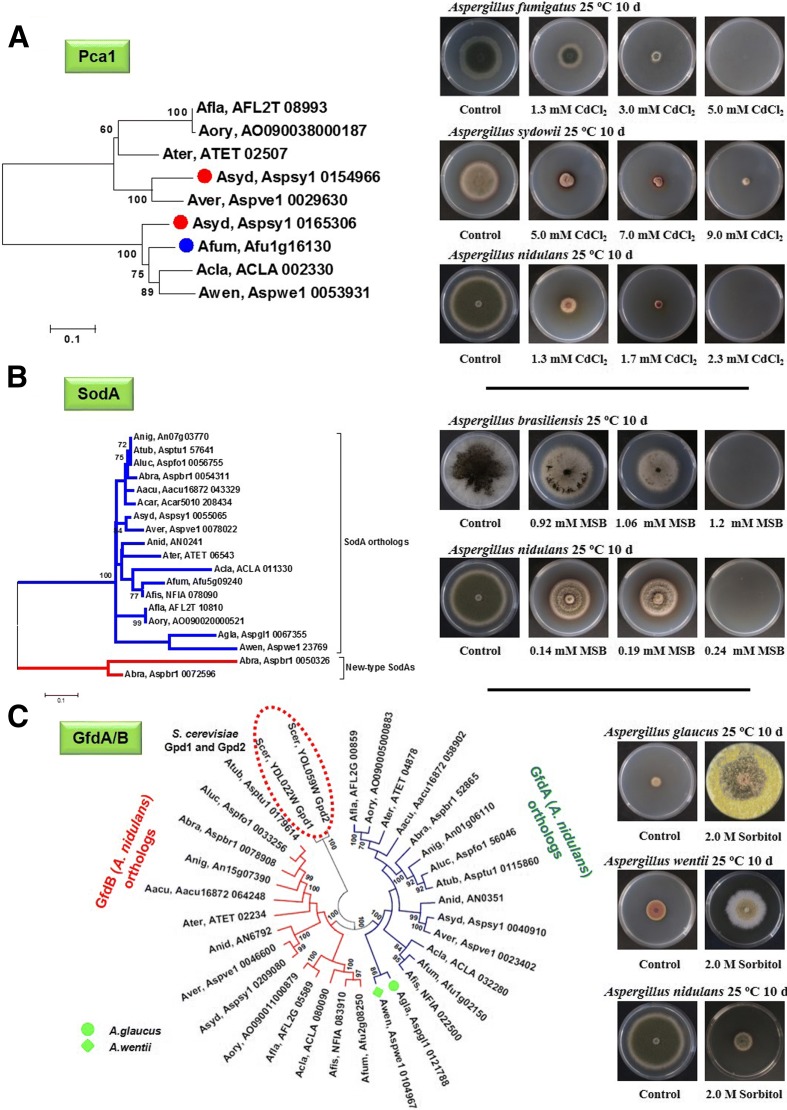
Linking species-level differences in selected *Aspergillus* stress-defense proteins to major stress tolerance phenotypes. The remarkable Cd(II) tolerance of *A. fumigatus* and *A. sydowii* (**a**), the outstanding menadione sodium bisulfite (MSB) tolerance of *A. brasiliensis* (**b**) and the osmophility of *A. glaucus* and *A. wentii* (**c**) were attributed to variations in the cadmium transporting P-type ATPases (**a**), Cu/Zn-superoxide dismutases (**b**), and NAD-dependent glycerol-3-phosphate dehydrogenases (**c**), respectively. In this Figure, the stress sensitivities of *A. nidulans* are also presented for comparison (**a**–**c**), although this species lacks any Pca1 ortholog (Additional file 15). In the dendograms, putative orthologs of baker’s yeast Pca1 cadmium transporting P-type ATPase (**a**), *Aspergillus nidulans* SodA Cu/Zn-superoxide dismutase (**b**), and *A. nidulans* GfdA/B NAD-dependent glycerol-3-phosphate dehydrogenases (**c**) are shown. In (**c**), *S. cerevisiae* Gpd1/2 paralogs are also presented. Putative *A. sydowii* () and *A. fumigatus* () Pca1 orthologs (**a**) and putative *A. glaucus* () and *A. wentii* () GfdA orthologs (**c**) are indicated by *symbols* presented in the *parentheses*. Hypothetical new-type SodA enzymes found in *A. brasiliensis* are indicated by *red lines* in (**b**). Pca1, SodA, and GfdA/B orthologs are indicated by four-letter species name identifiers (listed in Additional file 15) and locus IDs as found in AspGD (http://www.aspergillusgenome.org/) for the aspergilli. The relevant JGI locus IDs are listed in Additional file 16. The Gpd1/2 paralogs of budding yeast are from the *Saccharomyces* Genome Database (http://www.yeastgenome.org/). *Photographs* on the tress tolerance/sensitivity phenotypes were taken from the Fungal Stress Database (http://www.fung-stress.org/). Further details of the phylogenetic and evolutionary calculations and more information on the stress response proteins can be found in Additional file 15

In solid media experiments, *A. brasiliensis* showed outstanding menadione tolerance, which correlated with the emergence of two putative new-type SodA superoxide dismutases (Fig. 9). In liquid cultures, *A. carbonarius* and *A. niger* CBS113.46 were especially resistant to high oxidative stress provided by hydrogen peroxide while *A. zonatus* and *A. sydowii* were the most sensitive species (Fig. 8). In the case of *A. carbonarius*, the remarkable H_2_O_2_ tolerance was coupled to the co-occurrance of two C-type—one group cIII and one group cIV—catalases (Additional file 15). The catalase-encoding *catC* gene (coding for group cIV catalase; Additional file 15) has been lost in some *Aspergillus* genomes including *A. fischeri*, *A. clavatus*, and *A. fumigatus*. In the latter species, the *easC* gene for ergot synthesis (a group cIII catalase gene, Additional file 15) [134] appears to have taken over protective functions against oxidative stress (Additional files 15 and 17).

#### Hyperosmotic stress

One of the most interesting outcomes of the stress studies was that osmophily was more widespread in the genus *Aspergillus* than previously thought. Experimental evidence was obtained for osmophily in *A. glaucus*, *A. wentii*, *A. versicolor*, *A. sydowii*, and *A. oryzae* (Additional file 3). In the case of *A. glaucus* and *A. wentii*, osmophily correlated with the lack of GfdB, a putative NAD^+^-dependent glycerol-3-phosphate dehydrogenase, with a predicted function in glycerol metabolism (Fig. 9; Additional file 15). This is consistent with previous work in *A. nidulans* showing that elimination of *gfdA*, which also encodes a putative glycerol metabolic enzyme, led to an osmoremediable phenotype in the presence of 1 M NaCl (Additional files 3 and 15) [135].

#### Heavy metal stress

Cd(II) tolerance was clearly correlated with the presence of a Pca1-type Cd(II) transporter in the studied genomes (Fig. 9, Additional files 3 and 15). Not surprisingly, *A. sydowii*, *A. fumigatus*, *A. wentii*, *A. versicolor*, and *A. terreus*, all harboring a Pca1 ortholog, were the most tolerant to Cd(II). Interestingly, the important opportunistic human pathogen *A. fumigatus* also possessed high Cd(II) tolerance, but it was remarkably sensitive to oxidative, osmotic, and cell wall integrity stress when compared to other aspergilli (Fig. 9, Additional files 3 and 15).

#### Cell wall integrity stress and sensitivity to antimycotics

The cell walls of the aspergilli consist of chitin, β-1,3-glucan, β-1,6-glucan, α-1,3-glucan, galactomannan, and galactosaminogalactan. Genes required for the synthesis and remodeling of these polymers were found in all sequenced *Aspergillus* genomes, although a high degree of redundancy was observed (Additional file 17). The number of chitin synthases was in the range of 9–15 (noting that *S. cerevisiae* only harbors three chitin synthases). The number of chitin remodeling, chitin-glucan crosslinking, and β-glucan synthesis enzymes also differed considerably (21–31, 10–18, and 15–31, respectively). No such extreme differences were observed with respect to galactomannan and galactosaminogalactan synthesis. However, there was no correlation between the number of cell wall genes and overall susceptibility to the cell wall acting drugs calcofluor white, Congo Red, and caspofungin (Fig. 8, Additional files 15 and 17). In addition, voriconazole and amphotericin B, which affect the composition and structure of cell membranes, also showed species-specific effects (Additional file 17). Therefore, it is likely that numerous strategies have evolved in different *Aspergillus* species to counteract the presence of these antifungals, as has previously been suggested [136].

### Transporters

The complex mixtures of nutrients found in natural growth substrates require transport across cell membranes in order to be assimilated into the fungal cell. To analyze genes that encode transporters, 19 *Aspergillus* genomes were compared to 16 genomes of other fungi, resulting in the detection of up to 120 different transporter-related Pfam motifs in at least one species. The total number of transporters identified in the genomes varied dramatically from 301 transporter-encoding open reading frames (ORFs) in *Schizosaccharomyces pombe* to 1601 in *A. versicolor*. The number of ORFs contributing to the transportome of members of the Eurotiomycetes was variable, the lowest number being 810 in *A. clavatus*, which is still more than twice the number found in the yeasts *S. pombe* or *Saccharomyces cerevisiae*. The majority of transporter types (as denoted by the presence of the Pfam motifs) are present in similar numbers in the genomes of all the fungi included in this study. The Major Facilitator Superfamily, which contributes the largest number of ORFs to the transportome, is also the transporter class showing the highest level of expansion in the Eurotiomyces, with the ABC transporter superfamily also showing a noticeable increase. This expansion of the transporter capacity of the fungi correlates with the ability to use a greater diversity of substrates. The redundancy of most transport systems renders the study of fungal transporters a complex topic where much remains to be discovered. Some subclasses of transporters are discussed in more detail below.

#### The Aspergillus sugar transportome

Sugars are not only important nutrients and structural precursors but are also signaling molecules that trigger changes in gene regulation. Sugar transport and sensing are therefore crucial functions in fungal cells and considerable progress in understanding the molecular bases of these processes has been made in studies on yeast [137]. Most sugar transporters belong to the Major Facilitator Superfamily (MFS) [138, 139] of secondary transporters (secondary active transporters and facilitators), which currently comprises 82 named classes [140, 141]. In order to investigate the sugar transporter complements of diverse filamentous fungi, we focused on those proteins from *Aspergillus* species that were classified as members of the PFAM family of “Sugar and other transporters” (PF00083). Most, but not all, members of this family catalyze sugar transport, with their substrates including hexoses, pentoses, disaccharides, alpha-glucosides, inositol, and quinic acid as well as organic and inorganic ions.

**Fig. 10:**
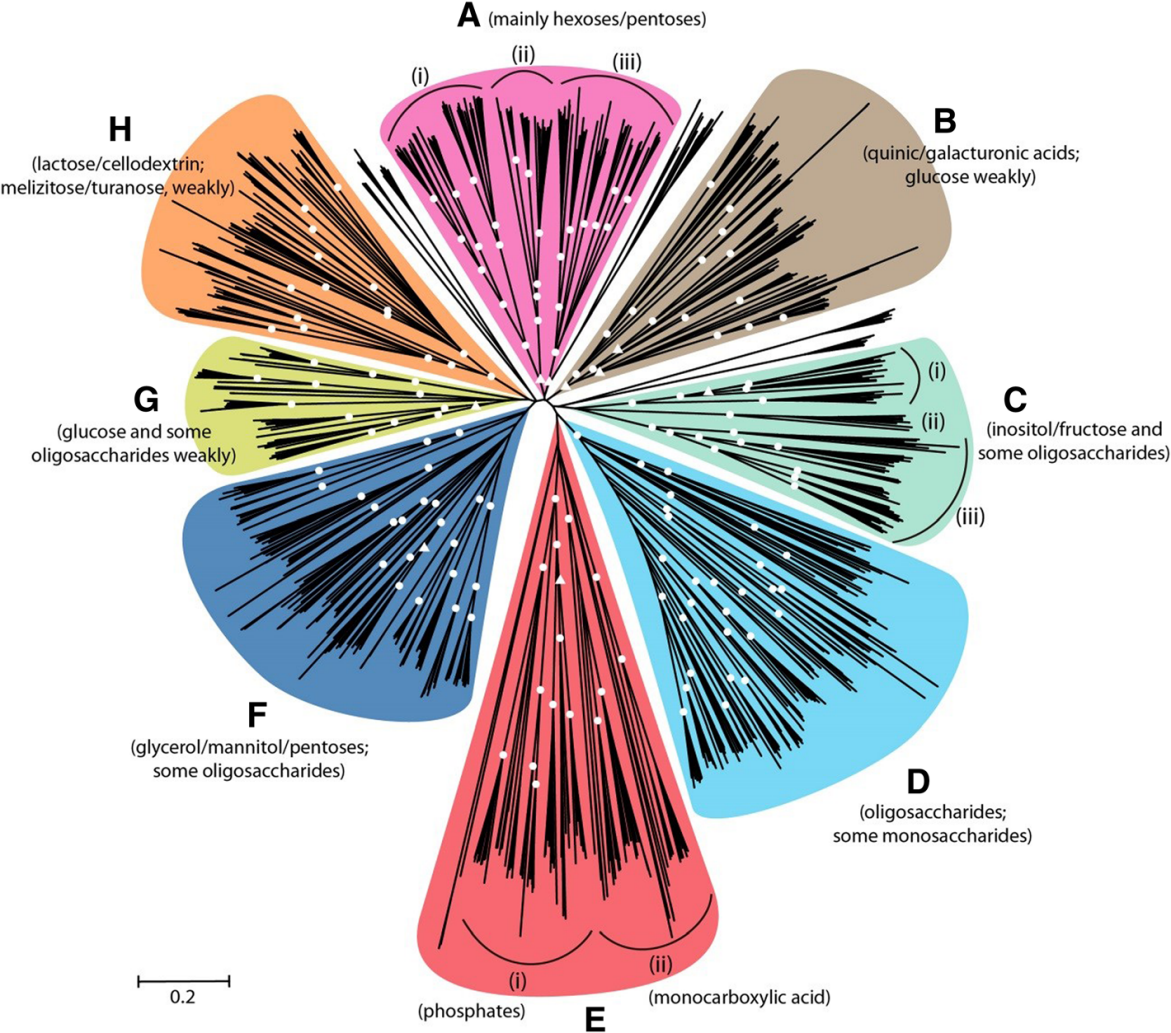
Overview of sugar transporters. The evolutionary history of 940 PF00083 sequences identified in the genomes of *A. clavatus*, *A. flavus*, *A. fumigatus*, *A. nidulans*, *A. niger*, *A. oryzae*, *A. terreus*, and *A. fischeri* was inferred using the neighbor-joining method. The optimal tree is shown. Nodes with 50–69% (∆) or 70–100% (○) bootstrap support (1000 replicates) are indicated. Evolutionary distances were computed using the Poisson correction method and are in the units of the number of amino acid substitutions per site. All ambiguous positions were removed for each sequence pair. Evolutionary analyses were conducted in MEGA6. A small number of sequences could not be assigned to the main clades identified; referring to the smallest genome analyzed (*A. clavatus*), these correspond to *loci* ACLA023780, ACLA026890, ACLA031920, and ACLA041390. Potential substrates for members of the clades are suggested based on experimentally determined transporter functions

Using the annotated genome data from eight previously available *Aspergillus* species, a total of 940 putative PF00083 transporters were identified after manual filtering and reannoation to yield greater consensus to the conserved 12 transmembrane domain topology. From phylogenetic analysis, the vast majority of sequences were found to fall into eight major clades (A to H; Fig. 10). Although support for the very basal branches is generally low, the subclades within them have high bootstrap support.

This initial analysis was extended to include an additional 1422 sequences corresponding to the PF00083 complements of the novel *Aspergillus* genomes, along with those of two fungi for which experimental evidence of function has been obtained for a number of PF00083 members: *N. crassa* (Sordariomycete) and *S. cerevisiae*. For comparison, the complements of *P. chrysogenum*, *T. marneffei*, *T. reesei*, and two animal pathogens (*Histoplasma capsulatum* and *Microsporum canis*) were also included. Additional files 20 and 21 present a summary and details, respectively, of the numbers and distribution of PF00083 members found in each of the clades and the function(s) identified for some members of those clades. Considerable diversity in the numbers of PF00083 members was found, with a threefold difference existing between those species having the largest and smallest complements (*A. sydowii* vs. *A. clavatus*). Even species from the same sections do not have very similar numbers of loci, for example: members of the *Nigri* range from 83 to 113. Of the *Nidulanti*, *A. sydowii* (180) and *A. versicolor* (171) have the highest numbers of loci whereas the number present in *A. nidulans* (113) is similar to that observed in *A. niger* and *P. chrysogenum*. The three genome-sequenced *A. niger* strains (consisting of an enzyme producer, a citric acid producer, and a laboratory strain) all have similar PF00083 complements. Perhaps surprisingly, *T. reesei* (Sordariomycete), a well-known producer of plant cell wall degrading enzymes, only has 64 members. Interestingly, *A. clavatus* has a very similar complement size (63) but there is a notable enrichment of *T. reesei* members in clade D and a reduction in clade G compared to *A. clavatus. A. terreus* (123) and *A. wentii* (120) also have similar numbers of PF00083 members but clade D is threefold enriched in the former, while *A. wentii*, along with *A. sydowii* and *A. versicolor*, has the highest number of members of clade F. To evaluate transporter function in the different clades, we combined data from the literature with results from studies involving the functional complementation of *S. cerevisiae* sugar transporter mutants with 43 putative transporters from *A. niger* (Additional file 22).

Clade A comprises three distinct subclades (bootstrap values >90%) and experimental data demonstrated the transport of certain monosaccharides for a number of its members. Both high-affinity and low-affinity glucose transporters of *A. nidulans* (HxtC, HxtE, MstA, MstC, and MstE), *A. niger* (MstA, MstC), and *N. crassa* (Hgt-1, Glt-1) are found in subclade A(i), as well as two broad-specificity transporters of glucose/pentoses (An29-2, Xyt-1) [138, 139, 142–147]. Paralogous high-affinity glucose transporters (MstA and MstC) differing in their patterns of expression are present in *A. nidulans* [144]. This duplication is also found in the other two *Nidulantes* (in fact *A. sydowii* has three loci, as does *N. crassa*) but is absent in all the other aspergilli analyzed here. *A. glaucus* lacks MstA/MstC homologs. Analysis of the *A. niger* transporters indicated the ability to transport D-glucose, D-galactose, and D-mannose for all tested proteins, while all but one were also able to transport D-fructose (Additional file 22).

An *N. crassa* transporter (Lat-1) of broad sugar substrate specificity (arabinose and other sugars) and a closely related arabinose-specific transporter from *Myceliophthora thermophila* (MtLat-1) map to subclade A(ii) [148], as does the HxtA protein of *A. nidulans* [149]. The tested *A. niger* transporters of this group showed broad specificity, covering the monosaccharaides and oligosaccharides D-glucose, D-mannose, D-fructose, D-galactose, maltose, melizitose, and turanose (Additional file 22).

*A. nidulans* and *N. crassa* xylose transporters [150, 151] occur in subclade A(iii). While one of the tested *A. niger* transporters from this subclade appeared to be specific for D-glucose, the others are more similar in their specificities to the members of subclade A(i) (Additional file 22). One member is also able to transport the disaccharides maltose and trehalose. Clade A might therefore be considered to comprise three diverse groupings of predominantly pentose/hexose transporters.

Clade B contains quinic acid [152, 153] and D-galacturonic acid (GAT-1, GatA) [154, 155] transporters. GatA homologs are present in most aspergilli except section *Fumigati*, *A. clavatus*, and *A. zonatus*. By contrast, no GAT-1 homologs are present in section *Nigri*, *A. glaucus*, or *A. zonatus*. Analysis of *A. niger* members of this clade only revealed the ability to transport D-glucose for some members (Additional file 22).

Clade C comprises three major subclades supported by very high bootstrap values. Subclade C(i) contains homologs of the *S. cerevisiae* inositol transporters Itr1 and Itr2 [156] and the putative *Cryptococcus neoformans* inositol transporters [157]. Subclade C(ii) members show homology to the fructose transporters Fsy1 and Frt1 identified in *S. pastorianus (carlsbergensis)* and *Botrytis cinerea*, respectively [158, 159]. Clade C(iii) comprises homologs corresponding to “group 2” putative inositol transporters [157] that are related to the putative inositol transporter Hgt19 of *Candida albicans* [160]. The *A. niger* members of this clade revealed highly diverse abilities to transport monosaccharides and/or oligosaccharides (Additional file 22).

Clade D incorporates oligosaccharide transporters including the *A. oryzae* maltose permease MalP and its homolog [161], homologs of the melibiose transporter MBT1 of *Colletotrichum graminicola* [162], the maltose transporters of *S. cerevisiae* (Mal11, Mal31, Mal61, and Mph3) [137], and MRT of *Metarhizium robertsii* [163]. Similar to Clade C, highly diverse abilities of the tested *A. niger* members of this clade were observed (Additional file 22).

Clade E can be divided into two main subclades: E(i) contains homologs of phosphate transporters [164–166] and the *S. cerevisiae* transporter (GIT1) of glycerophosphoinositol and glycerophosphocholine [167], while members of E(ii) share homology with the *S. cerevisiae* monocarboxylic acid/selenite transporter Jen1 [168]. No *A. niger* members of this clade have been characterized.

Clade F contains the yeast glycerol transporter Stl1 [169], the *N. crassa* pentose permease XAT-1 [145], and the xylose specific permease An25 [151]. Several of the *A. niger* members of this clade were able to transport oligosaccharides, while others appeared to be specific for glycerol or mannitol (Additional file 22).

No homology to a functionally characterized transporter has been observed for any members of clade G. However, one of the tested *A. niger* members was able to transport D-glucose while another transported maltose, trehalose, and melizitose.

Clade H contains the *A. nidulans* lactose permeases LacpA and LacpB [170, 171] and *N. crassa* cellodextrin transporters [172, 173]. One of the tested *A. niger* members was able to transport melizitose and turanose (Additional file 22).

It is unclear if the widespread occurrence of broad-specificity transporters in *A. niger*, as indicated by the functional complementation studies in *S. cerevisiae*, is specific to this species or common among a wider range of fungi. A transportome containing a diversity of broad-specificity sugar transporters is congruent with a saprobic lifestyle where degradation of natural substrates provides complex mixtures of monosaccharides, disaccharides, and trisaccharides.

#### Amino acid transporters

Phylogenetic analysis (Additional file 23) makes it possible to propose which paralogs are responsible for the uptake of aromatic amino acids (two clades, shown in purple) and those responsible for the uptake of basic amino acids (shown in red). This analysis suggests that, in some cases, specificity arose prior to the divergence of the Saccharomycotina and the Pezizimycotina (e.g. for basic and di-carboxylic amino acids, and for proline) while in other cases the amino acid specificity evolved later. The *S. cerevisiae*-specific subclade shown in light orange is suggested to be a group of broad specificity permeases (excluding proline) based on their similarity to *N. crassa pmg* and its possible ortholog from *Trichoderma harzianum* [174, 175]. The clade clustering with Tat3 of *Saccharomyces pastorianus* (deep purple) [176] may indicate a specificity for aromatic amino acids, while the ability to transport cystine is indicated by the *Candida glabrata* gene *YAT* [177]. A clade, shown in blue contains the well-characterized proline permease of *A. nidulans* ([178] and references therein), clustered with its ortholog, Put4 from *S. cerevisiae*; similarly the *S. cerevisiae* aspartate/glutamate transporter Dip5 is an outgroup of a clade shown in green which contains the isofuctional ortholog of *A. nidulans* [179].

A recent study [180] has allowed us to predict that the smaller clade (shown in red) represents lysine-specific permeases while the larger clade (also shown in red; including AN8279) probably transports arginine and other basic amino acids. Interestingly, AN8279, possessing a Ser residue in TMS10, resembles the T456S mutant of Can1, suggesting that AN8279 is a high affinity and high capacity transporter for both arginine and lysine. A detailed study of the substrate specificity determinants of the *A. nidulans* PrnB transporter suggested that substrate specificity of this family is largely determined by residues in certain key transmembrane domains [178, 181].

The aspergilli screened were found to possess variable numbers of AAAP (Amino Acid/Auxin Permease) family members (TCDB 2.A.18) [140] and some *S. cerevisiae* AAAP proteins have been described as bi-directional vacuolar amino acid transporters, with some shown to be broad specificity permeases accepting aromatic and aliphatic amino acids [182]. All aspergilli were found to possess at least one methionine transporter.

#### Urea transporters

Urea is taken up by fungi and plants through a group of active urea/H^+^ symporters, belonging to the sodium:solute symporter family (SSS), as typified by *A. nidulans* UreA [183, 184] With the exception of *A. zonatus*, which has only one homolog, the *Aspergillus* genomes were found to contain a range of 2–7 UreA homologs, most of which are predicted to have 15 transmembrane segments (TMSs) (Table 5). The phylogenetic relationships of the UreA homologs suggest that two duplication events occurred before the different *Aspergillus* species diverged from a common ancestor, giving rise to two clades (Additional file 24). The first sub-clade, which contains UreA, also contains an ortholog from each of the fungi analyzed, suggesting conservation of urea transport function. The second clade contains members that have not yet been functionally characterized.

**Table 5:** Number of UreA homologs found in the analyzed aspergilli. In *A. niger*, *A. luchuensis*, and *A. fumigatus*, the same number of homologs were found in each of the two sequenced strains

Species	UreA homologs (*n*)	Species	UreA homologs (*n*)
*A. niger*	4	*A. oryzae*	3
*A. luchuensis*	4	*A. flavus*	3
*A. tubingensis*	3	*A. terreus*	4
*A. brasiliensis*	4	*A. fumigatus*	3
*A. carbonarius*	4	*A. fischeri*	3
*A. aculeatus*	2	*A. clavatus*	3
*A. versicolor*	7	*A. glaucus*	4
*A. sydowii*	5	*A. wentii*	5
*A. nidulans*	4	*A. zonatus*	1

Amino acid residues important for the binding, recognition, and/or translocation of urea and for the sorting of UreA to the membrane were recently identified [185]. All of these residues are conserved in all UreA orthologs of the genus, with the exception of *A. aculeatus* where an alanine (Ala163 in UreA) is substituted by a serine. This is remarkable since there is a Ser present at the corresponding position in characterized UreA orthologs of *S. cerevisiae*, *C. albicans*, and *P. involutus* [185].

#### Nitrate, nitrite, and ammonium transporters

All sequenced aspergilli were found to possess at least one copy of the *nrtA* gene encoding a Nitrate Nitrite Porter (NNP, TCDB 2.A.1.8) [140] protein for nitrate/nitrite transport [186–188]. This conserved nitrate transporter-encoding gene is located in a cluster with genes for nitrite reductase and nitrate reductase. *Aspergillus nidulans*, *A. fumigatus*, and *A. fischeri* also have a second nitrate transporter located outside the cluster. In *A. nidulans*, it has been shown that the two transporters, NrtA (in the cluster with nitrate and nitrite reductases) and NrtB, are expressed under identical conditions but have different affinities for nitrate and nitrite, suggesting a kinetic flexibility that may confer an advantage to the species growing in its natural habitat in soil where substrate concentrations can vary rapidly by orders of magnitude [189, 190]. NrtA displays low affinity, high capacity while NrtB is a high affinity, low capacity protein. In species with a single NNP, the protein more closely resembles NrtA rather than NrtB using a Smith-Waterman algorithm for pairwise comparison (Additional file 25).

Nitrite transport can also be performed by proteins of the formate-nitrite transporter (FNT) family (PF01226; TCDB 2.A.44) [140]. Bioinformatic analysis in the present study revealed that the majority of the aspergilli contain one gene encoding an FNT family protein, but a few species (*A. flavus*, *A. oryzae*, *A. sidowii*, *A. fischeri*, and *A. versicolor*) have two such genes. In *A. nidulans*, the FNT protein NitA has been shown to be a high affinity but low capacity nitrite transporter compared to NNP proteins and is expressed under the same conditions as the NNP proteins [191].

The aspergilli also contain one to six members of the proton-dependent oligopeptide transporter (PTR) family (PF00854: TCDB 2.A.17) [140] that includes the *Arabidopsis thaliana* nitrate transporter, Nrt1.1 (CHL1). However, absence of the nitrate binding residues identified in Nrt1.1 (H356 and Y388) [192, 193] implies that nitrate transport by PTR family proteins in the aspergilli is unlikely.

The aspergilli usually possess three or four members of the ammonium transporter/methylammonium permease/Rhesus factor family (AMT/MEP/Rh family) (PF00909; TCDB 1.A.11.2) [140], although the *A. zonatus* genome contains five genes encoding such proteins. In *A. nidulans*, these genes are differentially regulated with MeaA being the principle ammonium transporter in conditions where ammonium is non-limiting. By contrast, MepA is expressed under nitrogen limiting and nitrogen starvation conditions and MepB is only expressed during complete nitrogen starvation [194]. MepA and MepB display higher substrate affinity than MeaA [194].

The inorganic nitrogen transport capability emphasized by the present study most likely reflects the natural habitat of *Aspergillus* species in the soil where nitrate, nitrite, and ammonium form the principle inorganic salts of nitrogen. The diversity of transporters detected is of applied significance as it indicates flexibility in terms of the inorganic nitrogen sources that can be provided for growth of biotechnologically important aspergilli. However, for nitrate utilization (and by extension nitrite utilization), uptake of nitrate has generally been considered a “bottleneck” and so the manipulation of gene copy number in strains could be a goal for strain improvement. On undefined industrial media used for growing fungi, there may be scope for higher biomass yields and faster growth by manipulating inorganic nitrogen transporters.

#### Nucleobase and nucleoside transporters

There are two main fungal families specific for nucleobase and one for nucleoside uptake as described below. In addition, a fourth family, the Equilibrative Nucleoside Transporter (ENT)-like family (2.A.57) [140], with a similarity to nucleoside transporters of protozoa and metazoa, is also present in fungi but has not been characterized [195]. Some of these transporters (e.g. NCS1 and AzgA; see below) are ubiquitous in fungi but absent in mammals, which makes them promising candidates for designing novel targeted pharmacologic agents against pathogenic fungi [196].

##### Nucleobase Ascorbate Transporter family

Homologs of the Nucleobase Ascorbate Transporter (NAT) family (or Nucleobase Cation Symporter NCS2 family, 2.A.40) [140] are present in all domains of life and most fungi [197–199]. Members are thought to consist of 14 transmembrane domains (TMD) and have extended cytoplasmic N-tails and C-tails [200]. Fungal NATs can be further classified intro three distinct subfamilies based on their specificity and primary sequences. First are the “canonical” UapA/C group [201, 202], which are uric acid/xanthine/H+ symporters [203–205]. UapC-like homologs were found to be syntenic and present in all species of aspergilli in the present study (and duplicated in some) [206], whereas UapA paralogues were present only in *A. nidulans* and in *A. zonatus*. UapA-like transporters are present outside the aspergilli (e.g. in *N. crassa* and in members of the Pezizales, a basal group of the Pezizomycotina), so the present data perhaps contradict the suggestion that UapC was the ancestral gene and UapA had arisen by a recent duplication [207]. Second, UapD members were detected in section *Nigri*, but no data supporting their function as nucleobase transporters have yet been reported [205]. Finally, the AzgA group comprises H^+^ symporters specific for adenine, hypoxanthine, and guanine, and this group was present in all of the sequenced aspergilli [204, 208, 209]. This family, despite no evident similarity in primary amino acid sequence, seems ancestrally related to the NAT family [209].

##### Nucleobase Cation Symport 1 family

Nucleobase Cation Symport 1 (NCS1) family (2.A.39) [140] proteins are H^+^ (or Na^+^ in some bacteria) symporters specific for the uptake of purines, pyrimidines, thiamine, hydantoin, pyridoxine, and related metabolites in prokaryotes, fungi, and some plants. All members contain 12 TMDs and extended cytoplasmic tails. NCS1 transporters of the aspergilli and other dikarya can be divided into two distinct subfamilies, Fur and Fcy, both of which are probably the result of independent horizontal transfers from prokaryotes [207]. Fur homologs in the aspergilli and penicillia have presviously been shown to exhibit patterns of gene loss and duplication [195]. Homologs of FurD (uracil) and FurA (allantoin) were the only ones found to be present in all analyzed species in the present study. Convergent evolution has occurred within the Fur family several times, so that prediction of substrate specificities without functional assays is impossible [195]. The phylogenetic relations of these transporters are shown in Additional file 26.

The Fcy family of *A. nidulans* includes an H^+^/cytosine-purine symporter (FcyB) [210, 211] and recently identified cryptic transporters for either adenine or guanine [212]. Orthologs and paralogs of Fcy were found to be present in most of the examined aspergilli and penicillia (Additional file 27).

#### Concentrative Nucleoside Transporters family

The Concentrative Nucleoside Transporters (CNT) family (2.A.41) [140] is exemplified by CntA of *A. nidulans*, which functions as a general H^+^/nucleoside symporter [195]. One Cnt-like homolog is present in most of the genomes investigated, with the homologs being extremely well conserved and fully syntenic, with variations only in the N-tail. However, they are not present in *A. zonatus* and *A. aculeatus*, but some evidence for remnants (pseudogenes) was observed. No homologs were found in *S. pombe*, *S. cerevisiae*, plants, protozoa, and archaea. The presence of an ortholog of CntA in *C. albicans* [213] indicates a possible conservation of function throughout the Ascomycotina.

### Flavohemoglobins and nitric oxide (NO) sensitivity

**Fig. 11:**
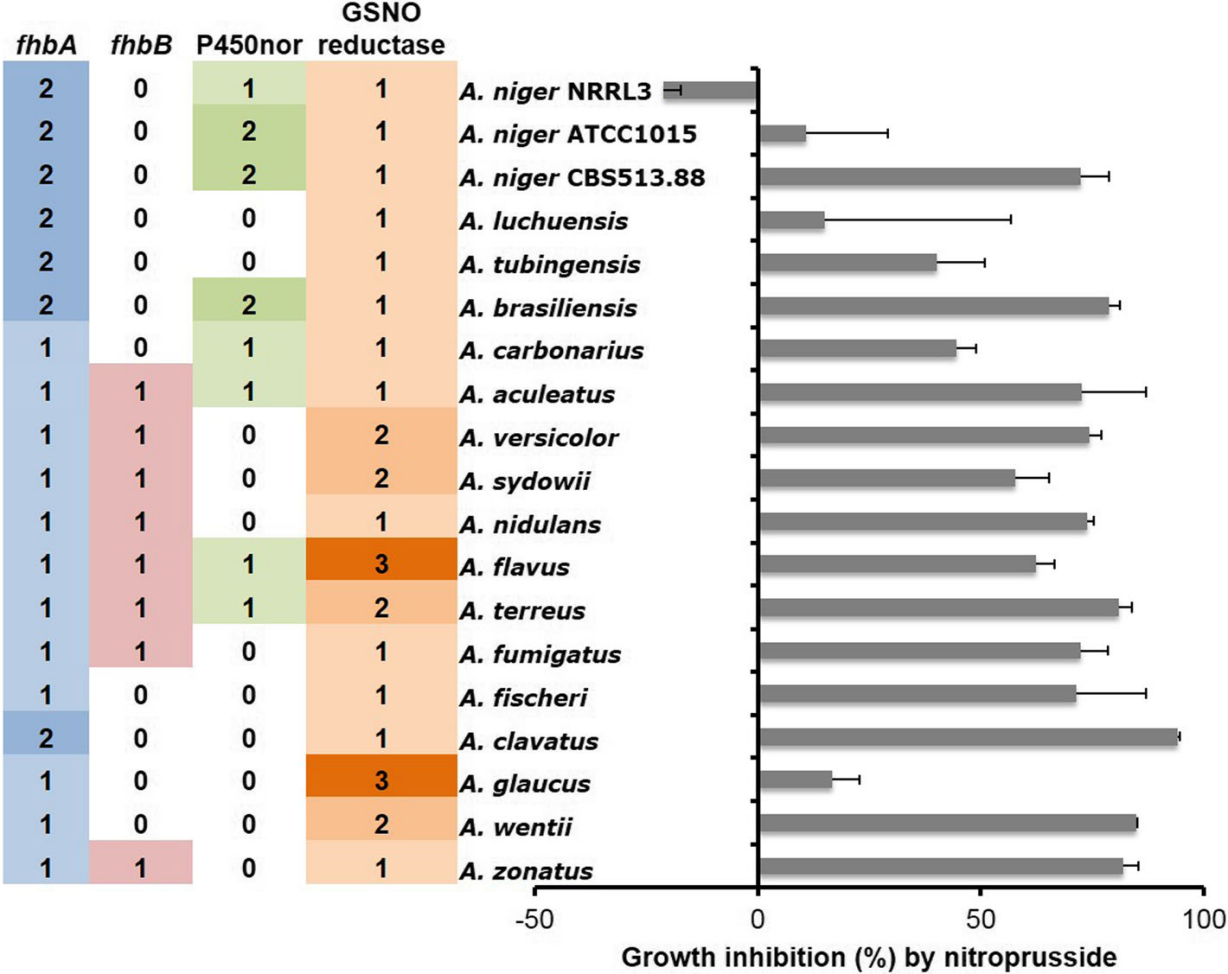
Inhibition of growth by NO. The *table* depicts the number of homologs of the flavohaemoglobin genes (*fhbA* and *fhbB*), the P450 nitric oxide reductase, and the S-nitrosoglutathione (GSNO) reductase in each species. Strains are listed in the same order as in the phylogenetic tree shown in Fig. 1. Strains were grown in the presence or in the absence of the NO-releasing compound nitroprusside. Growth was monitored at different time intervals. The *graph* shows the percentage of growth in the presence of 128 mM nitroprusside compared to the control samples grown in the absence of nitroprusside. The time and temperature of growth was optimized for each strain and it was as follows (species name, strain, temperature, and incubation time for growth inhibition calculation): *A. glaucus:* 22 °C for 72 h; *A. tubingensis*: 30 °C for 96 h; *A. zonatus*: 30 °C for 96 h; *A. brasiliensis*: 30 °C for 72 h; *A. versicolor*: 30 °C for 96 h; *A. sydowii*: 30 °C for 96 h; *A. niger* ATCC1015: 30 °C for 48 h; *A. luchuensis*: 30 °C for 72 h; *A. niger* NRRL3: 30 °C for 72 h; *A. wentii*: 30 °C for 96 h; *A. niger* CBS 513.88: 30 °C for 96 h; *A. fischeri*: 30 °C for 96 h; *A. terreus*: 30 °C for 96 h; *A. flavus*: 30 °C for 96 h; *A. fumigatus*: 30 °C for 96 h; *A. clavatus*: 30 °C for 96 h; *A. nidulans*: 30 °C for 96 h; *A. carbonarius*: 30 °C for 72 h; *A. aculeatus*: 30 °C for 96 h

Nitric oxide (NO) is a gaseous radical with a short half-life and a high reactivity. For these reasons, fungi have evolved a diverse set of strategies to cope with NO [214]. Flavohemoglobins are a family of conserved proteins in bacteria and fungi which have the ability to detoxify NO via an NADPH, FAD, and O_2_-dependent conversion of NO to NO_3_^–^ [215]. Flavohemoglobins have been previously characterized in *A. nidulans* and *A. oryzae*, where two proteins have been identified [216–218]. In *A. nidulans*, it was found that deletion of both flavohemoglobins *fhbA* and *fhb*B resulted in increased sensitivity to NO [216, 219]. In *A. oryzae*, FhbA was localized in the cytosol, while FhbB showed mitochondrial localization [218], although it was also found that FhbA contains a predicted motif for secretion in *A. niger* and some other aspergilli [217]. The cytosolic flavohemoglobin (*fhbA*) is regulated by nitrogen source and the mitochondrial one during development [216, 219]. A cytochrome P450 nor (nitric oxide reductase) that reduces NO to N_2_O was found in *Fusarium oxysporum* [220]. This P450nor is not widespread in aspergilli, being present only in six species (Fig. 11). A S-nitrosoglutathione (GSNO) reductase converts GSNO into ammonia and oxidized glutathione (GSSG) in *Cryptococcus neoformans* and *Magnaporthe oryzae* [221, 222]. GSNO red is present in all *Aspergillus* species sequenced in this work. Some of the strains have up to three copies of this gene (Fig. 11).

#### In silico analysis of flavohemoglobins

A search for homologs of flavohemoglobins in the genomes of the test aspergilli revealed that all these species contain at least one gene encoding flavohemoglobin A (Additional file 28). Five species contain two copies of flavohemoglobin A and eight species contain one *fhbA* and one *fhbB* in their genomes. Phylogenetic analysis confirmed that flavohemoglobins A and B form two well-defined clusters (data not shown). The duplication of flavohemoglobin A could have happened after the separation of the *A. nidulans–A. versicolor–A. sydowii* cluster, which groups separately from the rest of the aspergilli *fhbA* genes. Interestingly, the *fhbB* genes of these three species also cluster together.

#### NO sensitivity

Most of the *Aspergillus* species were sensitive to high concentrations of the NO-releasing compound sodium nitroprusside (Fig. 11, Additional files 3 and 28). However, some were resistant to NO, in particular *A. glaucus*, *A. niger* (NRRL3 and ATCC1015), and *A. luchuensis.* Other strains, such as *A. tubingensis* and *A. carbonarius*, showed an intermediate sensitivity to NO. We could not find any correlation between the sensitivity to NO displayed in our growth tests and the number of copies or the type of genes involved in the detoxification of NO. The presence of one or two types of flavohemoglobins (cytosolic or mitochondrial) and the fact that all species having two copies of *fhbA* do not have the other flavohemoglobin (*fhbB*) is intriguing. Although no association between number and/or type of flavohemoglobin and sensitivity to NO is evident, it may reflect an adaptation to different lifestyles. However, all species contain three to six copies of NO detoxification genes with at least two types of mechanisms (Fig. 11). For example, in *A. fumigatus* detoxification of NO does not seem to be required for full virulence [223, 224] and it contains only three genes for the NO detoxification (two flavohemoglobins and one GSNO reductase) (Fig. 11).

### Signal transduction pathway genes

**Fig. 12:**
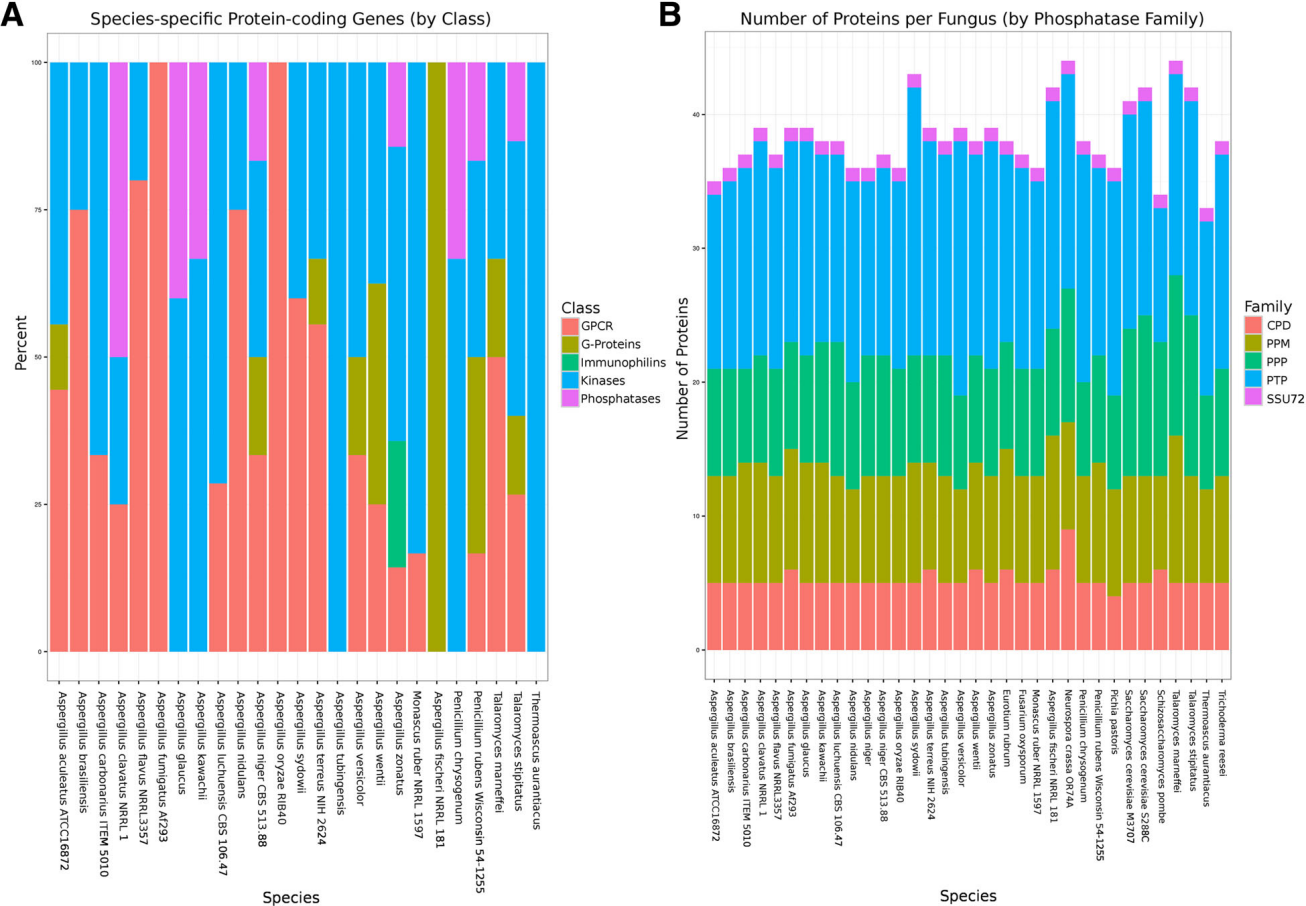
**a** Proportion of species-specific genes of the different signal transduction protein-classes in Eurotiomycetes. **b** Number of protein phosphatases in fungi. Eurotiomycetes appear in *red*

Gene family members for four different protein classes involved in signal transduction in Eurotiomycetes were identified using a protein domain-based approach. In total 11,120 proteins were identified belonging to 29 Eurotiomycetes species, clustered into 765 orthologous groups. The analysis of orthologous groups produced a set of genes that could not be clustered with any other and are regarded here as putative species-specific genes. Of the 29 Eurotiomycetes genomes, 25 contain species-specific genes (Fig. 12a) that may be involved in particular traits of these species. G-protein-coupled receptor (GPCRs) and kinases contribute most to these genomic novelties and thus constitute candidates for further functional studies aiming at understanding the differences among them (Additional file 29).

#### G-protein receptors and subunits

Heterotrimeric G-proteins have universal roles as signaling proteins in eukaryotes. Each G-protein heterotrimer is composed of α, β, and γ subunits that interact with the plasma-membrane, in association with a GPCR. Most filamentous fungi have three conserved Gα subunits (I, II, and III), one Gβ protein, and one Gγ protein. The number of predicted GPCRs varies widely, with a larger number identified in ascomycete than in basidiomycete fungi [225–227]. Results of the domain-based identification of G-proteins and GPCR orthologs in the eurotiomycetes are illustrated in Additional file 28. Gα proteins were found to be highly conserved. The Gα group I (*fadA*) regulates multiple pathways; most filamentous fungi in this study have a single copy of this gene (Additional files 29 and 30). In contrast, Group II Gα proteins (*ganA*) are less conserved than the other Gα proteins and up to three copies were found in one species (*T. stipitatus*) (Additional file 30). The Group II Gα proteins play a positive role in germination of conidia, possibly through cAMP signaling in carbon sensing [228, 229]. The last Gα belongs to group III (*ganB*) and was found as a single copy in all eurotiomycetes where it is thought to act as a negative regulator (Additional files 2, 29, and 30). Additionally, we found three different Gα groups of orthologs (Clusters FungiJGICTBE21135 only present in *A. niger* ATCC 1015, FungiJGICTBE20122 present in *A. flavus* NRRL 3557 and in *A. oryzae* RIB40, and FungiJGICTBE14195 present in *A. flavus* NRRL 3557 (Additional files 29 and 30) that appear to be encode genes that contain the Gα domain. There was also one copy of the Gβ negative regulator of asexual reproduction (*sfaD*) in each genome (Additional files 2, 30, and 31) [230]. Finally, all Eurotiomycetes genomes contained a single highly conserved Gγ gene [231].

#### Serine-arginine protein kinases

Members of the serine-arginine protein kinases (SRPKs) family are known to phosphorylate serine within RS (arginine/serine rich) regions [232]. As previously reported in *A. nidulans*, *N. crassa*, and dermatophytes, there is high genome expansion of SRPKs in filamentous fungi [21]. Interestingly, the *A. nidulans* SRPK types were spread in seven clusters of orthologous genes, whereas only two SRPK types have been found in *N. crassa* (Additional file 32). However, *A. nidulans* is the only eurotiomycete with all seven SRPK types. Most of the SRPK members identified in Eurotiomycetes belong to the types A and F, while the SrpkB and SrpkD types were less conserved in the eurotiomycete genomes. It has been suggested that SRPKs of filamentous fungi are involved in the molecular response to a broad range of environmental stimuli, although these signaling pathways are currently poorly understood [233]. Since the main biological role for SRPKs is the regulation of messenger RNA (mRNA) processing, it is conceivable to speculate that the expansion of SRPK in the eurotiomycetes could reflect the greater importance of splicing in filamentous fungi than in *S. cerevisiae*, whose genes generally do not contain introns. A core set of 48 kinases is present in all eurotiomycetes and in other species of Ascomycota analyzed (*N. crassa*, *F. oxysporum*, *T. reesei*, *S. cerevisiae*, *P. pastoris*, and *S. pombe*), which suggests that these kinases have a critical role in the biology of those organisms. Components of mitogen-activated protein (MAP) kinases are classical examples of conserved kinases [234] and are involved in cell polarity and cell wall maintenance (Kin1, BckA, MkkA, MpkA), mating (SteC, Ste7, Ste 20), and osmotic stress sense (HogA, SskB, PbsA) [233].

In contrast to the above conserved pathways, there is growing evidence that filamentous fungi might have evolved unique kinases. Indeed, a block of kinases that appear to be specific to filamentous fungi have been identfied, named filamentous fungal kinases (Ffk from A to J). These show low sequence identity with kinases of *S. cerevisiae*, *Caenorhabditis elegans*, *Drosophila melanogaster*, and human [233].

Since single deletions of FfkA, FfkB, FfkD, FfkE, FfkF, FfkG, FfkH, and FfkI from *A. nidulans* were not lethal and did not give a different phenotype [233], little is known about the functionality of these kinases [99]. The present study found a similar group of kinases among the Eurotiomycetes that do not appear to have orthologs in the yeast species analyzed (Table 6) [233]. Overall, *P. pastoris*, *S. cerevisiae*, and *S. pombe* encode fewer kinase orthologs than the other fungal species analyzed, with 98, 126, and 101 copies, respectively, which is almost half the number of kinases predicted in the *P. rubens* Wisconsin (205). Conversely, there is a core set of 31 kinases (Additional file 33) that do not appear in any of the eurotiomycetes, but were retained in *S. cerevisiae*, *P. pastoris*, and/or *S pombe*. Twenty-five kinases are essential in *A. nidulans* [233] and all the analyzed eurotiomycetes have at least one copy of these genes, suggesting they are essential across the tested species.

**Table 6:** Filamentous fungal kinases without orthologs in model yeast species

Cluster	*A. nidulans* kinase locus tag	Domain	*A. nidulans* name	Picpa1	Sacce1	SacceM3707_1	Schpo1
FungiJGICTBE4492	AN10485	Pkinase	Stk21	0	0	0	0
FungiJGICTBE1792	AN6339	Pkinase	Pod6	0	0	0	0
FungiJGICTBE4614	AN1560	Pkinase	PlkA	0	0	0	0
FungiJGICTBE4599	AN4717	Pkinase	PkaB	0	0	0	0
FungiJGICTBE3865	AN0822	Pkinase	KfsA	0	0	0	0
FungiJGICTBE1403	AN4936	Pkinase	Prp4	0	0	0	1
FungiJGICTBE4249	AN7537	Pkinase	Ppk33	0	0	0	1
FungiJGICTBE2868	AN3101	HiskA	PhkB	0	0	0	1
FungiJGICTBE1795	AN3102	HisKA	PhkA	0	0	0	2
FungiJGICTBE4245	AN6044	Pkinase	NpkA	0	0	0	1
FungiJGICTBE4202	AN3065	Pkinase	CmkB	0	0	0	1
FungiJGICTBE3486	AN0699	Pkinase	Cak1	0	0	0	1
FungiJGICTBE668	AN4479	HisKA	NikA	2	0	0	0
FungiJGICTBE164	AN11032	Pkinase	SepL	1	0	0	1
FungiJGICTBE3739	AN4385	Pkinase	SepH	1	0	0	1
FungiJGICTBE3573	AN10800	BCDHK_Adom3	pkpA	1	0	0	1
FungiJGICTBE2991	AN11101	Pkinase	Gin4	1	0	0	1
FungiJGICTBE3299	AN5728	Pkinase	Stk22	0	1	1	1
FungiJGICTBE2936	AN10515	Pkinase	PrK1	0	1	1	1
FungiJGICTBE598	AN1665	Pkinase	stk51	1	1	1	0
FungiJGICTBE878	AN2412	Pkinase	CmkA	1	2	2	0
FungiJGICTBE3318	AN7563	Pkinase	ChkC	1	1	1	0

#### Protein phosphatases

Protein phosphatases are classified into the following main groups: (1) classical Ser/Thr phosphatases: phosphoprotein phosphatases (PPP) family and protein phosphatase Mg^2+^ or Mn^2+^ dependent (PPM) family; (2) protein Tyr phosphatase (PTP superfamily); and (3) Asp-based protein phosphatases with DXDXT/V catalytic signature (CPDs) [235]. Ssu72, a different type of protein phosphatases, is regarded as separate, although it shares some similarity with the PTP Superfamily [236]. We found, on average, 8.8 PPPs, 8.37 PPMs, 5.26 CPDs, and 15.3 PTPs in the fungi included in this study (Fig. 12b). The actual numbers varied considerably between species, especially for the PTPs. Ssu72, is an exception, showing a single member in all species. PTP is the largest phosphatase superfamily presented here, including classical PTPs [236], dual-specificity phosphatases (DSPases), that have an important role in intracellular transduction pathways and regulate the mitogen-activated protein kinase (MAPK) pathway and cell cycle progression, Cdc25 type phosphatases, either regulating cell cycle progression [237], and low molecular weight phosphatases [236]. The increased and variable repertoire of protein kinases and phosphatases in *Aspergillus* could have helped in the establishment of several complex morphological traits, as well, as sophisticated responses to stress and the evolution of pathways that can help in the pathogenic process.

### DNA protein organisation and methylation

#### Histones of the aspergilli

Five classes of histones have been identified in eukaryotes, comprising linker histone H1 and core histones H2A, H2B, H3, and H4. Bioinformatic analysis of the newly available *Aspergillus* genome sequences revealed the presence of all of these families of histones, with overall evidence of conservation throughout the aspergilli. However, there were some differences between species, the functional implications of which are unclear, as follows.

All analyzed *Aspergillus* genomes were found to contain an H1 histone gene, following manual curation. The sequences are highly similar at the N-terminus and within the globular domain (residues 18–93 in *A. nidulans*, Additional file 34). They diverge in the C-terminal domain, the maximal divergence being at the boundary of the globular domain with the C-terminal domain, due mainly to sequence insertions.

All *Aspergillus* genomes also contain a highly conserved H2B histone.

As previously reported [238], in the aspergilli, as in other ascomycetes, the extant H2A histone corresponds to the H2AX variant, rather than the canonical H2A form. This was confirmed for all aspergilli in the databases, where the diagnostic motif SQEL is conserved at the carboxy terminus. In all aspergilli, as reported previously for *A. nidulans* and *N. crassa* [239, 240], the H2AX gene is transcribed divergently from the H2B gene. In addition, all analyzed *Aspergillu*s genomes have an H2A.Z ortholog. While nothing is known about the role of this histone in any of the Pezizomycotina, the cognate gene is not essential in *N. crassa*, *S. cerevisiae*, or *S. pombe* [241, 242]*.*

All analyzed genomes contain an H3 gene, with complete amino acid sequence conservation for all species. While we have previously reported the presence of genes with significant amino acid similarity with histone H3 in *A. terreus* and *A. oryzae* [238], no other H3 paralogs were found in the other aspergilli nor in any of the other eurotiomycete genomes. The paralog in *A. terreus* appears real with two typical filamentous fungal introns but we could not find evidence for its expression in publicly available data. In the six sequenced strains of *A. oryzae*, as well as in the genome of the strongly related species *A. sojae* and *A. parasiticus* [243–245], a duplication has occurred of the central part of the functional H3 gene but no legitimate start-codon could be identified. There is no similarity among these sequences downstream of the H3-like sequence [238], which suggests that this “gene” results from a non-functional duplication of part of the canonical H3-encoding gene. This duplication is, however, absent in the ten currently sequenced strains of *A. flavus* [246]. The occurrence of a genetic feature common *to A. sojae*, *A. parasiticus*, and *A. oryzae* but absent from *A. flavus* (and *A. nomius*) is intriguing given the fact that *A. oryzae* are generally considered domesticated variants of *A. flavus* [9].

All species also contain the specific centromeric H3 histone (naming varies in different organisms, here named CenH3), with the motif LPFAR in helix α1 conserved in all species. Alignment of the CenH3 sequences (Additional file 34) shows the typical CenH3 divergence in the N-terminal region, which is a highly variable region in different species of *D. melanogaster* [247], confirming previous observations [238]. Additional file 34 also shows comparative phylogenies of the CenH3 histone and of the other sequence-variable histone, H1. CenH3 histone divergence has been proposed to be instrumental in driving speciation [247].

All genomes have two H4 encoding genes, one transcribed divergently from the H3 encoding gene, denoted H4.I, and a second one, denoted H4.II, unlinked to it. The H3-linked and unlinked H4 paralogs are almost identical at the amino acid sequence but the intron organization of the paralogs differs. Both H4 genes share an intron that splits the Arg4 codon. In H4.II, this is the only intron (in *Aspergillus* and *Penicillium*) and it is always at least twice as big as that in the H4.I paralog, often >200–350 nt. The H4.I gene generally has a second intron interrupting the Arg96 codon. This 3′ intron must have been lost in *A. zonatus*, *A. glaucus*, *A. rubrum*, and also in *Penicillium paxilli*, since it is present in the eight sequenced species that belong to other families of Eurotiales (*Talaromyces*, *Thermoascus*, *Monascus*, *Byssochlamys*, and *Thermomyces*) and in Onygenales. In addition, the amino acid at position 2 is a Ser or Thr in both I and II paralogs. Note that in a given species, paralogs I and II are always identical at this position (Ser for *A. flavus*, *A. glaucus*, *A. nidulans*, *A. oryzae*, and *A. sydowii* and *A. versicolor*, *A. rubrum*, and Thr for the other sequenced aspergilli). These species belong to different taxonomic sections within the aspergilli, suggesting that concerted evolution (possibly by gene conversion) could be responsible for the residue at this position. Similarly, at position 65, there is almost always a Thr in paralog I, which is a Ser in paralog II. At position 69, there is always a Gly in paralog I and a Ser in paralog II.

We have reported previously the presence of a small reading frame with significant similarity to a central part of histone H4 in the genome of *A. nidulans* [238]. We have now found similar reading frames in most *Aspergillus* genomes. This include species where it was previously overlooked or where it was erroneously annotated. The newly recognized “third” histone 4 gene invariably contains seven introns. As a consequence, this gene has considerably more intronic sequences than coding sequences; in *A. nidulans* for instance, the 357 nt long coding sequence (36%) is interrupted by 642 nt of intronic sequences (64%). The additional putative H4 gene is absent from *A. glaucus* and *E. rubrum* as well as from *Penicillium* species, but present in other sequenced Eurotiales species as well as in the Onygenales (Additional file 34).

#### Putative DNA methylases

Two putative DNA methylases have been described in the ascomycetes. They were identified through work relating to the phenomena of MIP (Masc1, *Ascobolus immersus*) and RIP (RID1, *N. crassa*) as inactivation of the *masc1* and *rid‐1* prevent MIP and RIP, respectively [248, 249].

There are, however, serious doubts as to whether *masc1* and *rid-1* encode actual DNA methylases. First, no DNA methylase activity could be detected in vitro with the purified proteins [248, 249]. Second, all DNA methylation in *N. crassa*, even methylation subsequent to RIP, depends on another protein, DIM2 [250]. Third, a homolog of Masc1 and RID1 was reported for *A. nidulans* (DmtA), a species for which DNA methylation has never been detected [251, 252] (Reyes Domínguez and Scazzocchio, unpublished data). Similar results were obtained for *A. flavus* [253]. Interestingly, DmtA is essential for meiosis in *A. nidulans* [251].

All the sequenced aspergilli were found to have Masc1/RID1 homologs at syntenic positions. A phylogentic tree shows DIM2 as outgroup to all other sequences (Additional file 35), while RID1 of *N. crassa* clusters with the two *Talaromyces* species, which, in the absence of other sequences in the tree, may simply mean that the latter have diverged less from an ancestral homolog. The pervasive presence of the DmtA orthologs may indicate that many *Aspergillus* and *Penicillium* “asexual” species are in fact sexual (see earlier “Asexual and sexual reproduction” section).

A DNA methylase with in vitro enzyme activity, Masc2, has been described in *A. immersus* [254] and BLAST analysis revealed a new protein family present in all aspergilli in syntenic positions. The *A. nidulans* protein includes from residue 322 to 586 a putative (incomplete) C‐5 cytosine DNA methylase domain while two (incomplete) SNF_2N domains occur from residues 1305 to 1470 and 1627 to 1818. No function is known for this protein or its homologs, but orthologs are present in all available sequences of *Penicillium* and *Talaromyces*, and in *A. immersus* and *Tuber melanosporum*, members of Pezizales, a basal order of the Pezizomycotina. Interestingly, in *N. crassa*, the fungus where DNA methylation has been more thoroughly studied, no protein with the same architecture is present nor was one found in most other Sordariomycetes.

## Conclusions

Data presented in the present study emphasize the high genomic and functional diversity present within the genus *Aspergillus*. While strong conservation was observed for central biological functions (e.g. sporulation, aspects of cell wall structure, and histones), high diversity was apparent for many other physiological traits, such as carbon utilization, secondary metabolism, and stress response. While most aspergilli can occupy a broad range of habitats, our data suggest that evolution has generated considerable diversity within the genus to allow their exploitation of and persistence in different habitats.

The detailed analyses described in this paper will serve as a reference for variation in other fungal species. The unique position of this genus in the genomics of filamentous fungi, with genome sequences for a large number of species, will become even more evident with the future completion of the currently ongoing *Aspergillus* whole genus genome project in which more than 300 genomes from species of this genus will be sequenced. The data of our study will provide a backbone to rapidly perform detailed genomic comparisons and functional studies in these new genomes.

## Methods

### Genome sequencing, assembly, and annotation

#### Genome sequencing and assembly

The majority of genomes and transcriptomes in this study (*A. luchuensis*, *A. brasiliensis*, *A. glaucus*, *A. sydowii*, *A. tubingensis*, *A. wentii*, *A. versicolor*, and *A. zonatus*,) were sequenced with Illumina and assembled with AllPathsLG [255], except for *A. aculeatus* and *A. carbonarius*, which were sequenced using a combination of 454 and Sanger platforms and assembled with Newbler (Roche). For all standard fragment Illumina libraries, 500 ng–1 μg of genomic DNA was sheared using the Covaris E210 (Covaris) and sized selected for 270 bp using Agencourt Ampure Beads (Beckman Coulter). The DNA fragments were treated with end repair, A-tailing, and adapter ligation using the TruSeq DNA Sample Prep Kit (Illumina) and purified using Agencourt Ampure Beads (Beckma Coulter). These were sequenced on HiSeq in 2 × 150 bp read format and assembled using AllPathsLG with long-mate paired-end libraries, which were built using several approaches.

CLIP (Cre-Lox Inverse PCR) libraries were used for all Illumina sequenced genomes in this study except for *A. zonatus* and *A. glaucus*. For CLIP libraries, 10–15 μg of genomic DNA were sheared using the Hydroshear® and gel size selected for 4–5 kb. The fragments were end-repaired (NEB) and ligated with biotinylated Illumina compatible adapters containing loxP (IDT). The adapter ligated DNA fragments were circularized via recombination by a Cre excision reaction (NEB). The circularized DNA was digested using four base cutter restriction enzymes followed by self-ligation (NEB). The self-ligated products were immobilized on beads and inverse PCR was used to enrich for the final library (NEB). For *A. zonatus* and *A. glaucus*, ligation-free paired-end (LFPE) fragments were used. LFPE fragments were generated using the 5500 SOLiD Mate-Paired Library Construction Kit (SOLiD®). A total of 15 μg of genomic DNA was sheared using the Covaris g-TUBETM (Covaris) and gel size selected for 5 kb. The sheared DNA was end-repaired and ligated with biotinylated internal linkers. The DNA was circularized using intra-molecular hybridization of the internal linkers. The circularized DNA was treated with plasmid safe to remove non-circularized products. The circularized DNA was nick translated and treated with T7 exonuclease and S1 nuclease to generate fragments containing internal linkers with genomic tags on each end. The mate pair fragments were A-tailed and purified using Strepavidin bead selection (Invitrogen). The purified fragments were ligated with Illumina adaptors and amplified using ten cycles of PCR with Illumina primers (Illumina) to generate the final library. Quantitative PCR (qPCR) was used to determine the concentration of the libraries and were sequenced on the Illumina Hiseq in format 2 × 100 bp reads.

All Illumina reads from both types of libraries for each genome were assembled using AllPathsLG.

The genomes of *A. aculeatus* and *A. carbonarius* were sequenced using a combination of 454 standard, 454 paired-end, and Sanger fosmid libraries and assembled with Newbler. For 454 standard libraries, genomic DNA was fragmented by nebulization and purified using Ampure beads (Beckman Coulter). The ends of the fragments were treated with end repair and ligated with biotinylated adapters using either the Titanium (for *A. aculeatus*) or GS FLX (for *A. carbonarius*) Library Prep Kits (454). Adapter ligated products were immobilized on beads and treated with nick repair. Single strands were eluted to generate the final library. For 454 paired-end libraries, genomic DNA was sheared to 10 kb (for *A. aculeatus*) and 3 kb (for *A. carbonarius*) using the Hydroshear®. The ends of the fragments were ligated with biotinylated adapters containing loxP. The adapter ligated DNA fragments were gel-size selected and circularized via recombination by a Cre excision reaction (NEB). The circularized DNA was randomly sheared using the Covaris E210 (Covaris). The sheared fragments were end-repaired (NEB), ligated with 454 adapters (Roche), and immobilized on strepavidin beads followed by PCR using a biotinylated primer (Roche). The PCR product was immobilized and single-strand DNA libraries were isolated using an alkaline treatment. Sanger fosmid libraries for *A. aculeatus* and *A. carbonarius* were prepared from 20 ug of genomic DNA, sheared using the Hydroshear® and size selected for 40 kb using a pulse field gel. The long inserts were cloned into pCC1FOS vector. The hybrid 454-Sanger assemblies were produced using Newbler (Roche).

#### Transcriptome sequencing and assembly

For all transcriptomes in this study, except for *A. aculeatus* and *A. carbonarius*, stranded complementary DNA (cDNA) libraries were generated using the Illumina mRNA sample preparation kit (Illumina). mRNA was purified from 10 μg of total RNA using magnetic beads containing poly-T oligos. mRNA was fragmented using divalent cations and high temperature. The fragmented RNA was reverse transcribed using random hexamers and SSII (Invitrogen) followed by second strand synthesis. The fragmented cDNA was treated with end-pair, A-tailing, adapter ligation, and ten cycles of PCR. qPCR was used to determine the concentration of the libraries. Libraries were sequenced on the Illumina Hiseq in 2 × 100 bp read format. Illumina reads were de novo assembled into consensus sequences using Rnnotator (v. 2.5.2 or later) [256]. For *A. aculeatus*, cDNA libraries were generated using the cDNA Rapid Library Preparation Kit (Roche). mRNA was purified from total RNA using Absolutely mRNA™ purification kit (Stragene) and chemically fragmented using high heat. The fragmented RNA was reversed transcribed using random hexamers and AMV RT followed by second strand synthesis. The cDNA fragments were treated with end repair and ligated with 454 adapters. For *A. carbonarius*, mRNA was purified from total RNA using Absolutely mRNA™ purification kit (Stratagene). mRNA was reverse transcribed with SuperScriptIII using dT15VN2 primer. cDNA was synthesized with *E. coli* DNA Ligase, *E. coli* DNA polymerase I, and *E. coli* RnaseH (Invitrogen). cDNA was nebulized and gel purified to generate fragment sizes between 500–800 bp. The fragments were end-repaired, adaptor ligated, and made into single-strand DNA libraries using the GS FLX Titanium library kit. In both cases, 454 reads were filtered and screened for quality and contamination to include removal of ribosomal RNA, low-quality, and low-complexity reads. The remaining reads were assembled into contigs using Newbler (v2.3-PreRelease-6/30/2009) with default parameters.

#### Genome annotation

Genome assembly scaffolds were masked with RepeatMasker [257], RepBase library repeats [258], and repeats frequently detected (>150 times) by RepeatScout [259]. A combination of gene predictors was run on the masked assemblies: ab initio Fgenesh [260] and GeneMark [261]; homology-based Fgenesh + [260] and Genewise [262] seeded by BLASTx [263] alignments against the NCBI NR database; and transcriptome-based assemblies. In addition to protein-coding genes, tRNAs were predicted using tRNAscan-SE [264]. Predicted proteins were functionally annotated using SignalP [265] to predict signal peptides, TMHMM [266] for transmembrane domains, InterProScan [267] for protein domains, and protein alignments to NCBI NR, SwissProt, KEGG [268] for metabolic pathways, and KOG [269] for eukaryotic clusters of orthologs. Interpro and SwissProt hits were used to map Gene Ontology terms [270]. For each genomic locus, the best representative gene model was selected based on a combination of protein homology and EST support, which resulted in the final set of models used in this work.

#### Protein conservation and phylogeny

To assess protein conservation, we performed MCL clustering [271] with an inflation parameter of 2.0 on the 400,447 protein sequences from 37 predicted full fungal proteomes (25 from the Aspergillaceae, 12 ascomycete outgroups). Some 65,450 protein clusters (or families) resulted, with average size 6.12 including singletons and 19.40 excluding singletons, largest cluster containing 2179 proteins, and 47,245 singletons. Membership in a cluster was used to determine whether a protein was conserved across the comparative organism set. We considered a protein conserved if its homologs were found at least once in all members of an organism subset (e.g. section *Nigri* or Aspergillaceae). A protein was considered specific to an organism subset if it was found in at least one organism of the subset, but not in any organisms outside the subset.

Pfam domains [272] were assigned using v. 22 of the database and a TimeLogic Decypher machine and an e-value of 10^–4^.

The phylogenetic tree was generated from a separate cluster run which differed from the previous one by the addition of non-ascomycete outgroups *Rhizopus oryzae* and *Batrachochytrium dendrobatidis* (JGI portals Rhior3 and Batde5, respectively; omitted from Fig. 1). Protein sequences with single copies in each organism were aligned using MAFFT [273], trimmed to well-aligned regions using Gblocks [274] with default parameters. RAxML [275] was then run using the PROTMIXWAG model and *B. dendrobatidis* set as an outgroup to generate the phylogenetic tree shown in Fig. 1. All branches received maximum support, except for that between *A. niger* strains NRRL2 and ATCC 1015 (73%; Fig. 1b).

#### Identification of clusters of orthologous genes

Clusters of orthologous genes were identified using OrthMCL with an inflation value of 1.5 [276]. All other parameters, e.g., BLAST search, were used in the default settings.

### Identification/annotation of specific gene sets

#### Genes involved in asexual and sexual reproduction

The annotation pipeline of putative genes involved in asexual and sexual sporulation involved a two-step procedure of identification and annotation. Genes previously identified from *A. nidulans* were used as references (Additional files 2 and 5). The identification step of asexual genes was performed by searching the reference protein sequences against Aspergillus overall protein sequences with phmmer of HMMER 3.0 (http://hmmer.org/). Hits that passed MSV, Bias, Vit, and Fwd filters (see HMMER User’s Guide, http://eddylab.org/) were then subject to annotation involving BlastP comparisons against the database of non-redundant protein sequences [277] (Additional file 2). The identification of sexual genes was performed by searching protein databases available at the JGI (http://genome.jgi.doe.gov/) or AspGD (http://www.aspergillusgenome.org/) websites by BLASTP interrogation. Hits that had a minimum E value of 1e^–70^ were considered significant except for analysis of the relatively short PpgA protein where less stringent criteria were applied (Additional file 5). Synteny analysis (SYBIL) was then performed using the Jaccard Orthologous cluster option at AspGD or synteny option at the JGI website. Based on high levels of similarity and/or a large functional homogeneity of the hits, predicted proteins were annotated as corresponding functional proteins.

#### Genes involved in organic acid production

For determination of isoenzymes in production of organic acids, a list of known and predicted isoenzymes from *A. niger* CBS 513.88 was used as the seeding set. Using a BLAST-comparison, all genes above a cutoff value of 80% identity and 80% coverage were recorded as isoenzymes for the individual species.

#### Prediction and synteny analysis of aspergilli SM clusters

Protein and genomic sequences of aspergilli were downloaded from AspGD (http://www.aspgd.org/). Detection of PKS, NRPS, and DMATs and further prediction of respective SM gene clusters were made with SMIPS and CASSIS tools, correspondingly (https://sbi.hki-jena.de/cassis/) Synteny analysis was performed with the help of an *Aspergillus* 21-way comparative database asp2_v9 powered by Sybil. Conservation of clusters was calculated as the percentage of genes in the query cluster conserved in the syntenic counterpart. Confirmation of the identity of different secondary metabolites from the aspergili were checked by ultra high performance liquid chromatography–diode array detection–high resolution mass spectrometric detection (UHPLC-DAD-HRMS) [278].

#### Analysis of CYP proteins

Evolutionary analyses were conducted in MEGA5 [279]. The analysis involved 19 amino acid sequences. If protein size is marked by a star, the sequence was manually curated or sequences are incomplete. Following MUSCLE alignment, the evolutionary history was inferred by using the maximum likelihood method based on the JTT matrix-based model [280]. There was a total of 496 (CYP51F1) or 510 (CYP53) positions in the final dataset. The tree is drawn to scale, with branch lengths measured in the number of substitutions per site. All positions containing gaps and missing data were eliminated. Additional copies of CYP51F2 and CYP51F4 are excluded from the tree.

#### Aspergillus sugar transportome

The assignment of a protein to the “Sugar and other transporters” PFAM family PF00083 is based on its level of similarity to a Hidden Markov Model (HMM). The HMM for PF00083 is derived from a seed alignment of 33 proteins chosen to be the core representatives of that family [281, 282]. The latter includes proteins for which either transport of a specific sugar or related substrate has been experimentally demonstrated, or there is very strong indirect evidence that such a transport function exists, e.g. genetic evidence. Despite their considerable diversity in primary structure, PF00083 members exhibit a common topology of 12 membrane-spanning domains (TMSs) divided into two groups of six. Transmembrane (TM) domain predictions were obtained for all the PF00083-annotated proteins studied using TMHMM v2 [283]. While the majority of protein sequences exhibited 2 × 6 TM topology, we manually reannotated some *loci* to yield greater consensus with the 12 TM topology and a very small number were excluded from further analysis due to the detection of fewer than three TM domains. The resulting collection of amino acid sequences was aligned by MUSCLE and a neighbor-joining (NJ) tree was generated using MEGA6 software [284]. Cluster analysis was also performed using the orthology detection tool “Proteinortho”) [285] and the results obtained found to be concordant with the identifications made in the NJ tree.

#### Amino acid and urea transporters

The high-affinity methionine transporter of *S. cerevisiae* (MUP1) was used to identify several close homologs in all *Aspergillus* species. The methionine transporters of *S. cerevisiae* belong to the L-type amino acid transporter family (LAT; TCDB 2.A.3.8) [140] and exhibit low similarity to YATs [286]. *A. nidulans* UreA [[183, 184] was used to search the fungal genomes analyzed in this study for the presence of urea-like transporters (Table 5).

The *S. cerevisiae* proteins Tat1, Tat2, Tat3, Gap1, Hip1, Gnp, Agp1, Agp2, Agp3, Bap2, Bap3, Sam3, Mmp1, Lyp1, Alp1, Can1, Dip5, Put4, and Ssy1 obtained from the *Saccharomyces* Genome Database [287] were used as in silico probes for BlastP and in several cases, TBlastN searches in the available *Aspergillus* genomes at either the AspGD [14] or the JGI [15] databases. In addition, the homologues obtained for *A. nidulans* were used as in silico probes in a second BlastP search round. We have also identified a number of pseudo genes, either because their coding sequence contains frameshift and/or early chain termination mutations (ANID_4604, ANID_8556, ANID_8659, AFL2G_12189, AO090103000195, AO090005000021, AFL2G_06574, AO090026000738). In three other paralogues (ANID_5024, Aspbr1_139443, and Aspwe1_115075), the coding sequences lack a significant part of a functional YAT transporter [288]. These could be also considered pseudo genes or may have been recruited to a different function. Interestingly, no transcripts at all are detected (Jbrowse, [14]) for ANID_5024 and the adjacent gene (ANID_5023) in complete medium, while the genes on either side are transcribed under these conditions.

Alignment was done with MAFFT, Auto mode, Blosum62. Curation was carried out with BMGE. The tree shown was obtained using Blosum30 as a similarity matrix, but a topologically identical tree was obtained using the more stringent Blosum62 similarity matrix default setting. The maximum likelihood rooted tree was obtained with PhyML. The final tree with collapsed branches was drawn with FigTree.

#### Nucleobase transporters

The alignment of these putative transporters was performed using MAFFT [289]; curation was with BMGE [290] with a Blosum30 substitution matrix. Phylogeny was performed with PhyML [291] and re-drawing of the tree with FigTree [292].

#### Identification of protein class members involved in signal transduction

We employed a domain-based approach for the identification of proteins for each class, i.e. G-proteins, GPCRs, kinases, and phosphatases, following the general approach described by Perez-Rodriguez et al. [293] and using the pipeline available at [294]. The most important step is the identification of a protein domain or block that is characteristic of the protein class/family of interest and that is represented as a HMM, either available in the public database PFAM or created in house. Then specific rules are built accounting for the domain architecture of the different families/classes, in an exclusive way, i.e. a protein can belong to one and only one family/class, which requires the introduction of forbidden domains to resolve ties (Additional file 36). The HMMs used for the identification of protein classes of interest are available as (Additional file 37). Classification results are available in Additional file 31.

##### G-proteins

We studied the three sub-units of G-protein complexes: alpha, beta, and gamma. G-alpha and G-gamma had already well-described, characteristic, and specific domains in the PFAM database [282] with accession numbers PF00503 and PF00631, respectively. The G-beta subunit has several copies of the WD40 repeat (PF00400); however, this domain is not specific for G-beta proteins. We assesed the accuracy of our classification comparing our results against those presented in Li et al. [227]. The G-gamma famly is always found as a single member in each species. However, following our domain-based apporach we could not find the corresponding protein in *A. niger* CBS 513.88, *A. oryzae*, and *S. pombe*. Further inspection revealed that the protein is not annotated in the *Aspergillus* genomes, but we found a genomic region with the information for this gene, using the *A. nidulans* protein sequence as bait in a BLAST search (Additional file 37). In the case of *S. pombe*, the G-gamma subunit appears to be missing from the current version of the genome sequence.

##### GPCRs

For the identification of GPCRs, we took as gold standard the list provided by Li et al. [227] for *A. nidulans*. For some of the families (Stm1-related, GprK-like, PTH11, MG00532-like, material available under request) we built specific HMMs able to retrieve all of the known members of the respective family in *A. nidulans* and then applied them to the other species. Only in the case of PTH11 did we identify more members than the ones listed in the gold standard.

##### Kinases

We used as gold standard the identification and classification of kinases in *A. nidulans* from de Souza et al. [233]. All kinase classes have characteristic and specific domains available in PFAM, except the TOR kinases. We built an HMM specific for TOR kinases as described in Additional files 37 and 38.

##### Classical Ser/Thr phosphatases (PPP and PTP)

All PPPs in *A. nidulans* have the PFAM domain Metallophos (PF00149), but this domain also appears in other proteins. We retrieved all *A. nidulans* proteins that had a hit to the Metallophos domain and inferred the phylogeny, reveling that PPPs form a distinct clade in this evolutionary tree (Additional file 39), thus allowing us to build a PPP-specific HMM, named PP2Ac. In the case of the PPM family there were already specific PFAM domains that could be used to develop classification rules (Additional file 36).

##### PTPs

For most PTP superfamily members in [236] there were already specific domains in PFAM, i.e. Y_phosphatase, Y_phosphatase2, Y_phosphatase3, DSPc, and LMWPc. AN3941, belonging to the CDC25 subclass, was the unique PTP with no specific Pfam domain. Thus, we built a specific HMM (CTBECDC25) for this protein phosphatase. Although SSU72 proteins share sequence similarity with the PTP superfamily, they are classified as a distinct type of protein phosphatases. In that case, we used this PFAM domain Ssu72 Pfam for their identification.

##### Asp-based protein phosphatases

Aspartate-based catalysis phosphatases, which share the DXDXT/V catalytic signature (CPDs), had a specific domain found in the PFAM database, i.e. NIF (PF03031).

#### Histones of the aspergilli

Initial searches for histone H1 by BlastP failed to locate matches in some *Aspergillus* databases, while in other species the gene model was incorrect. Therefore, manual curation was required to establish its presence in *A. oryzae* and correction of the amino acid sequences was required for *A. brasiliensis*, *A. kawachii*, *A. sydowii*, *A. terreus*, *A. versicolor*, and *A. niger* CBS513.88. The NCBI database for *Aspergillus sojae* was interrogated to search for histone proteins in this species. Manual corrections of the H3 histone gene models were required for *A. niger* CBS 513.88, *A. wentii*, and *A. aculeatus*.

### Data access

Genome assembly and annotations are available at at the JGI fungal genome portal MycoCosm [15] and have been deposited at DDBJ/EMBL/GenBank under the following accessions: *A. aculeatus* ATCC16872 - MRCK00000000, *A. brasiliensis* CBS101740 - LJXV00000000, *A. carbonarius* ITEM5010 - AHIG00000000, *A. foetidus* CBS106.47 - MRBP00000000, *A. glaucus* CBS516.65 - LSTL00000000, *A. sydowii* CBS593.65 - MRCH00000000, *A. tubingensis* CBS134.48 - LJXU00000000, *A. versicolor* CBS583.65 - MRBN00000000, *A. wentii* CBS141173 (DTO134E9) - LJSE00000000, and *A. zonatus* CBS506.65 - MRBM00000000. Links to the raw genome and transcriptome data of these species are available in Additional file 1D. This also lists the genbank (if available) and JGI accession numbers of the genomes used for comparison as well as the papers in which they have been published.

Proteomics data of the wheat bran and sugar beet cultures are available via ProteomeXchange with identifier PXD005563. Proteomics data for the stress experiments are available at http://wwwuser.gwdg.de/~hkusch/GBIO_DeVries/.

### Expression of mating-type and pheromone-signaling pathway genes

Representative asexual species (*A. luchuensis* (*A. acidus*, *A. foetidus*), *A. aculeatus*, *A. brasilliensis*, *A. carbonarius*, *A. clavatus*, *A. niger*, *A. sydowii*, *A. versicolor*, *A. wentii*, *A. zonatus*), together with some control sexual species (*A. flavus*, *A. fumigatus*, *A. tubingensis*), were selected for experimental work to assess possible expression of mating-type (*MAT*) and pheromone-signaling pathway (*ppgA*, *preA*, and *preB*) genes. Identified *MAT1-1*, *MAT1-2*, *ppgA*, *preA*, and *preB* sequences were used to design primers for (RT)-PCR investigations (primer sequences available on request). Primers were designed ideally either side of an intron to allow differentiation of genomic or cDNA products by resulting product sizes. Genomic DNA was extracted from all species using a Nucleospin® Plant II kit (Macherey-Nagel). Cultures were grown in *Aspergillus* complete media (ACM) broth [46] at 28 °C with shaking for two days. Cultures were harvested by filtration through a sterile miracloth filter (Millipore) and washed with a pH 7.5 phosphate buffer solution, then freeze-dried and ground under liquid nitrogen into powder by motor and pestle. After quantification, 50 ng was used as a DNA template for subsequent PCR. To evaluate possible transcription of genes, RNA was extracted from cultures of all test species grown on solid media under conditions known to be favourable for sex in the aspergilli. Nylon filter membranes (pore size 0.2 μm) were placed over the surface of 5 cm Petri dishes containing 10 mL of oat meal agar (N.B. This media was used for all species except for *A. versicolor*, which did not grow well on this substrate. ACM agar was instead used for *A. versicolor*). A total of 50 μL of a spore suspension (containing 1 × 10^5^ conidia) was spread over the surface and cultures incubated in the dark at 32 °C. Plates were then sealed with two layer of Parafilm after 15–18 h of incubation [46]. Mycelia were then scraped off the surface of the filters after either four or eight days, dried using sterile paper tissue, and then flash frozen under liquid nitrogen. For most species, RNA extracted was then extracted using a TRizol method according to manufacturer’s instructions (Thermo Fisher). However, a CTAB method (Heather Darbyshir pers comm) was used for RNA extraction from members of the black aspergilli taxonomic grouping. For both methods, a Nucleospin® RNA extraction kit (Macherey-Nagel) was used for clean-up of the extracted RNA. After quantification, the extracted RNA was checked on agarose gel for purity and integrity of RNA. CDNA synthesis from all extracted RNA for all of species was achieved using Superscript^TM^ III; 500 ng of total RNA was used for cDNA synthesis, and then 1 μL of cDNA was used for subsequent amplifications. Final PCR was performed using a Phusion High Fidelity PCR kit according to manufacturer’s instructions (New England Biolabs). One microliter of each genomic DNA and cDNA from four-day-old and eight-day-old cultures were used as a template for amplification of *MAT1-1*, *MAT1-2*, *ppgA*, *preA*, and *preB* genes. In addition, expression of the *actA* (actin) gene was monitored as a control “housekeeping” gene. PCR products were resolved on 2.0–2.5% agarose gels. Successful amplification of all mating-type and pheromone-signaling pathway genes was achieved for for all species except for the *MAT1-2* gene of *A. versicolor*, which could not be amplified despite use of multiple primer sets and this gene was therefore excluded from the analysis.

### Growth profiling on different nutrient sources

For growth profiling, all strains were grown on MM [295] containing monosaccharides/oligosaccharides, polysaccharides, and crude substrates at 25 mM, 1%, and 3% final concentration, respectively. Cultures for proteomics were performed in MM with 1% wheat bran or 1% sugar beet pulp, as previously described [57], and proteomics was performed as described in that paper. For laccase and cellobiose dehydrogenase activity, the 20 aspergilli were grown in duplicate in liquid shaken cultures at 30 °C, 120 rpm. A total of 250 mL baffled erlenmeyers filled with 50 mL of MM [295] and supplemented with 10 g of cotton seed hulls were inoculated with 2 × 10^6^ spores/mL final concentration.

### Stress tolerance studies

In the stress tolerance studies, 18 *Aspergillus* strains with fully sequenced fungal genomes representing 17 species were included (Additional files 3 and 4). For the production of conidia, normally the malt extract—mycological pepton sporulation agar medium (1.5% agar)—recommended by CBS-KNAW (http://www.fung-stress.org/) was used. The sporulation medium for *A. glaucus* was also supplemented with 1.0 M NaCl. Unless otherwise indicated, asexual sporulation was carried out at 25 °C in the dark for six days. *A. fumigatus* and *A. nidulans* were also sporulated on the CBS-KNAW medium at 37 °C and on nitrate minimal medium agar plates (NMM, solidified with 1.8% agar) [296] at 37 °C. Microbial growth and stress sensitivity assays were performed on NMM agar plates (1.8% VWR International Agar powder for bacteriology, ID number 20 767.298), which were incubated at 25 (5 or 10 d) or 37 °C (5 d) in the dark and were supplemented with the stress-initiating agents H_2_O_2_ (oxidative stress), menadione sodium bisulfite (MSB, oxidative stress), NaCl (ionic osmotic stress), sorbitol (non-ionic osmotic stress), Congo Red (cell wall integrity stress), or CdCl_2_ (heavy metal stress) as required (Additional files 3 and 4; [133]). We typically performed two to three sets of experiments (often more) with spores harvested from independent sporulations for each stress condition. Each set contained 4-4 replicates. We took photos of the most representative colonies.

Stress agar plates were inoculated and, after completing the physiological experiments, the fungal colonies were photographed as described before [297]. Fungal growth was characterized either by colony diameters or by scoring them by eye on a 0–10 scale (Additional files 3 and 4). Stress sensitivities were quantified by percentage decreases in the colony diameters in comparison to controls or by decreases in the growth scores. Based on the growth inhibition values, all species tested were ranked according to their stress tolerances (Additional file 4). Physiological data have been stored in the Fungal Stress Database (FSD; http://www.fung-stress.org/) at CBS-KNAW. Stress response proteins were identified and annotated in the newly sequenced *Aspergillus* genomes following the procedure described previously [298] and newly identified orthologs were inserted into the Fungal Stress Response Database (FSRD) - version 2 [298] (FSRDv_2; http://internal.med.unideb.hu/fsrd2/?p=consortium). The phylogeny of selected well-characterized stress response proteins and their orthologs was studied following the procedure described in Additional file 3.

### Enzyme assays

For analysis of oxidative enzymes, the 20 aspergilli were grown in duplicate in liquid shaken cultures at 30 °C, 120 rpm. A total of 250 mL baffled erlenmeyers filled with 50 mL of medium were inoculated with to a final concentration of 2 × 10^6^ spores/mL. MM (for 1000 L: 6 g NaNO_3_, 1.5 g KH_2_PO_4_, 0.5 g KCl, 0.5 g MgSO_4_.7H2O, trace elements, CuSO_4_ 0.02 g, pH 6.0) was supplemented with 10 g of cotton seed hulls as the carbon source. Laccase activity in the culture supernatant was assayed by monitoring the oxidation of 500 μM ABTS at 420 nm to the respective radical (ε420 = 36 mM-1 cm-1) [299], in the presence of 50 mM sodium tartrate pH 4.0 at 30 °C.

The CDH activity was determined at 30 °C, using 0.2 mM 2,6-dichlorophenol indophenol (DCPIP) in 50 mM sodium acetate buffer, pH 5, in the presence of cellobiose 10 mM as the substrate [300]. The decrease in absorption of DCPIP (ε = 68,000 M-1 cm-1) was monitored at 520 nm for 30 s.

### Functionality of P450 proteins and NO sensitivity

Functionality of P450 proteins was tested by growing mycelia of aspergilli on MBFA agar supplemented with 0.02 mM ketoconazole or 2 mM benzoic acid. Inhibition of growth (Inhib.) as a percentage of the control without supplementation (Contr.) was determined. For nitric oxide sensitivity, all 19 species were cultivated in liquid MM containing 1% glucose and 10 mM ammonium tartrate in microtiter plates. Only medium used for *A. niger* CBS 513.88 was supplemented with a vitamin solution (80 mg/L *p*-aminobenzoic acid, 50 mg/L thiamin HCl, 1 mg/L biotin, 400 mg/L inositol, 100 mg/L nicotinic acid, 200 mg/L calcium pantothenate, 100 mg/L riboflavin, 50 mg/L pyridoxine). The liquid medium was supplemented with different concentrations of sodium nitroprusside (SNP): 0 (control samples), 16, 64, or 128 mM. A total of 1 × 10^4^ spores of each organism in a final volume of 150 μL per well was used as a replicate. Four replicates were done per organism per treatment. All fungi were incubated at 30 °C except for *A. glaucus*, which was grown at 22 °C. Absorbance was recorded at 492 nm every 24 h in an Axis expert plus plate reader. Percentage of growth inhibition was calculated comparing absorbance values at 0 versus 128 mM SNP. Growing time for calculations was in the range of 48–96 h depending on each organism (see Fig. 11 legend). Final values shown are the average of two independent experiments with four replicates per experiment.

### Functional analysis of sugar transporters

cDNA clones of the transporter genes were amplified by PCR and cloned into pYEX-BX using DNA ligase according to the manufacturer’s instructions. Sequence analysis was used to verify the correct clones. *S. cerevisiae* tester strains used were: KY73 [301], EB.VW4000 [302], MaDH4 [303], SEY6210 [304], CMY1050 [305]. Plasmids were derived from pYEX-BX (Clontech) [306]. Plasmid transformations of yeast cells were carried out according to the quick and easy TRAFO protocol [307]. Ten milliliters of YNB + 2% maltose supplemented as described above, was inoculated with a single yeast colony and grown overnight (16 h) at 30 °C and 250 rpm in an orbital shaker. OD_500_ measurements were taken for all cultures. The cells were centrifuged at 10,000 rpm for 10 s and, after the supernatant was removed, resuspended in MilliQ water. Cells were washed twice in this manner. A final centrifugation step (10,000 rpm for 10 s) was followed by resuspension in sufficient MilliQ water to result in a cell suspension with an OD of 0.1 (approx. 10^4^ cells/mL). NUNC 96-well optical bottom plates polymer base microtitre plates, containing media as described above, were inoculated with approximately 10^2^ cells to a final volume of 100 uL in a sterile manner. Plates were sealed using breathable seals and incubated at 28 °C. Growth was monitored by OD_590_ measurements taken at 0, 24, 48, 72, 96, and 120 h time points.

## Additional files

Additional file 1: Genome statistics and information. (XLSX 97 kb)
Additional file 2: Summary of putative genes related to asexual sporulation in tested strains. (XLSX 20 kb)
Additional file 3: Stress response in Aspergilli. (XLS 619 kb)
Additional file 4: Summary of putative genes related to mating and pheromone signalling in tested strains. (XLSX 12 kb)
Additional file 5: Expression of mating and pheromone-signalling pathway genes in representative asexual aspergilli. (PDF 488 kb)
Additional file 6: Overview of number of putative orthologs/paralogs in the compared fungal species related to primary carbon metabolism. (PDF 237 kb)
Additional file 7: Comparative growth profiling of the aspergilli and selected other fungi. (PDF 8.65 kb)
Additional file 8: Effect of SHAM and KCN on fungal growth and sporulation. (PDF 252 kb)
Additional file 9: Organic acid production. (XLSX 40 kb)
Additional file 10: CAZy comparison of the species. (XLSX 30 kb)
Additional file 11: Orthology analysis of CAZy genes related to plant biomass degradation. (XLSX 148 kb)
Additional file 12: Laccase and cellobiose dehydrogenase (CDH) activities of the selected aspergilli. (XLSX 11 kb)
Additional file 13: Molecular phylogenetic tree of CYP56 family (A), and CPR2 and CYP630 family (B). (PDF 402 kb)
Additional file 14: Molecular phylogenetic tree of CYP6001 - CYP6003 families (PpoA - PpoD) and colinearity of the genetic surroundings. (PDF 279 kb)
Additional file 15: Extended analysis of stress tolerance of the aspergilli. (PDF 706 kb)
Additional file 16: Orthologs of selected stress response proteins. (XLS 76 kb)
Additional file 17: Extended analysis of stress tolerance of the aspergilli (2). (PDF 1842 kb)
Additional file 18: Identified secreted proteins (*A. sydowii*, *A. tubingensis*, *A. wentii*, *A. zonatus*). (XLSX 34 kb)
Additional file 19: Strain specific PFAM domains predicted for identified secreted proteins (*A. sydowii*, *A. tubingensis*, *A. wentii*, *A. zonatus*). (XLSX 12 kb)
Additional file 20: Distribution of putative sugar transporters. (XLSX 17 kb)
Additional file 21: Detailed overview of putative sugar transporters in the aspergilli. (XLSX 10790 kb)
Additional file 22: Functional analysis of *A. niger* sugar transporters by complementation of *S. cerevisiae* transport mutant strains. (XLSX 12 kb)
Additional file 23: Maximum likelihood tree of YAT transporters in the aspergilli. (PDF 89 kb)
Additional file 24: Phylogenetic relationships of UreA homologs. (PDF 108 kb)
Additional file 25: CLUSTAL O(1.2.3) multiple sequence alignment of NNP nitrate transporters. (PDF 149 kb)
Additional file 26: Maximum likelihood rooted tree of Fur-like transporters of the aspergilli and some other members of the Eurotiales (*Penicillium*, *Talaromyces*). (PDF 537 kb)
Additional file 27: Maximum likelihood rooted phylogeny of the FCY-like transporters of the aspergilli and some penicillia. (PDF 324 kb)
Additional file 28: Number of flavohemoglobins and protein IDs found in the genome sequences of the *Aspergillus* species. (PDF 79 kb)
Additional file 29: Species-specific signal transduction pathway genes in Eurotiomycetes. (XLSX 14 kb)
Additional file 30: Predicted G-proteins and GPCRs proteins in the Eurotiomycetes. (XLSX 16 kb)
Additional file 31: Classification and Clusters of signal transduction related genes. (XLSX 1577 kb)
Additional file 32: Distribution of SRPKs in Eurotiomycetes. (XLSX 11 kb)
Additional file 33: Kinases presented in at least one yeast species and with no orthologous genes in other eurotiomycetes. (PDF 117 kb)
Additional file 34: Histones of the Aspergilli. (PDF 526 kb)
Additional file 35: Maximal likelihood phylogeny of MASC1/RID1 orthologs. (PDF 115 kb)
Additional file 36: Introduction of forbidden domains for the HMM analysis of signal transduction genes. (TXT 806 bytes)
Additional file 37: HMM file used for the identification of protein classes of interest related to signal transduction. (HMMS 4334 kb)
Additional file 38: Unannotated genes related to signal transduction. (XLSX 8 kb)
Additional file 39: Phylogeny of the Methallophos domain in *A. nidulans*. (PDF 96 kb)

## Abbreviations

AA: Auxiliary activity
AAAP: Amino acid/auxid permease
AspGD: Aspergillus Genome Database
CAZy: Carbohydrate Active enzyme database
CE: Carbohydrate esterase
CYP: Cytochrome P450
GH: Glycoside hydrolase
GPCR: G-protein-coupled receptor
H1: Histone 1
H2: Histone 2
H3: Histone 3
H4: Histone 4
JGI: Joint Genome Institute
MFS: Major Facilitator Superfamily
NO: Nitric oxide
PL: Polysaccharide lyase
SM: Secondary metabolite
SSS: Solute symporter family
TMD: Transmembrane domain
