# Supplementary material for: Comparative genomics reveals high biological diversity and specific adaptations in the industrially and medically important fungal genus Aspergillus
Source: Genome Biol. 2017 Feb 14;18:28. doi: 10.1186/s13059-017-1151-0 (PMC5307856; doi:10.1186/s13059-017-1151-0)
Supplement: Additional file 5: — Expression of mating and pheromone-signalling pathway genes in representative asexual aspergilli. (PDF 488 kb) [file 13059_2017_1151_MOESM5_ESM.pdf]

A

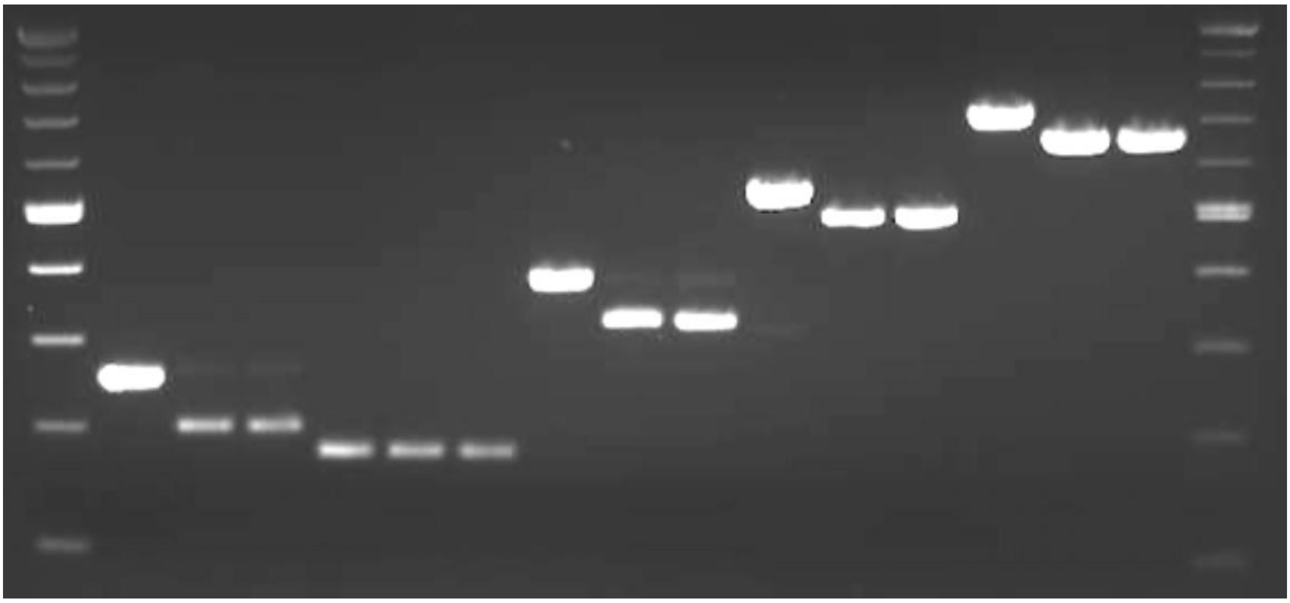

B

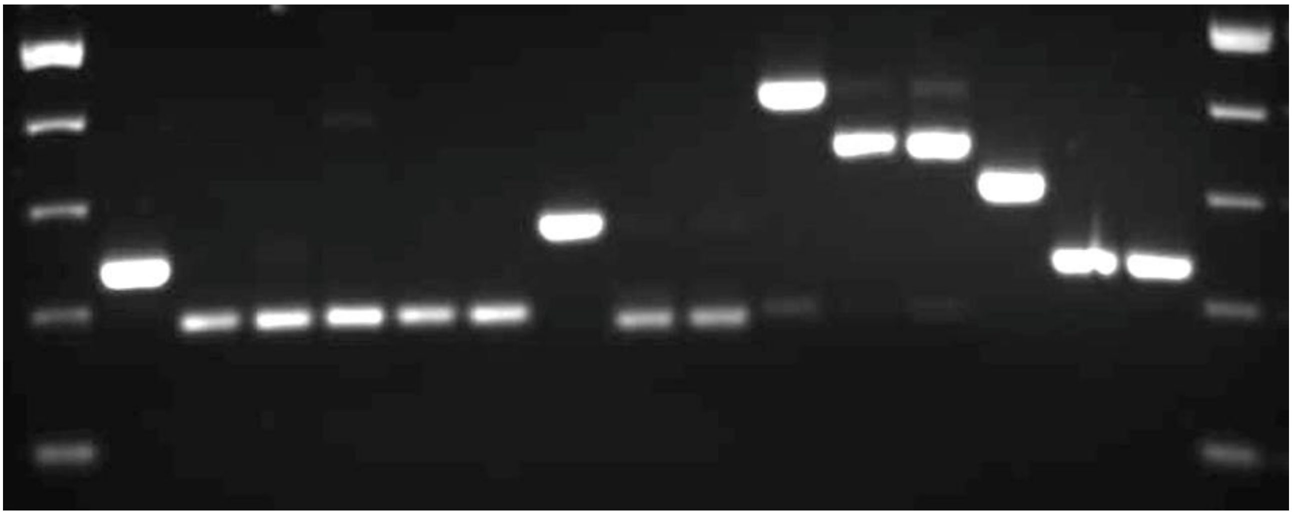

Additional File 5. Expression of mating and pheromone-signalling pathway genes in representative asexual aspergilli. (A) *A. wentii*, (B) *A. zonatus*. Lanes 1 and 17, 100 bp DNA ladder; lane 2, PCR of *MAT1*-2 from genomic DNA; lanes 3 and 4, PCR of *MAT1*-2 from cDNA from 4 and 8 days growth, respectively; lane 5, PCR of *ppgA* from genomic DNA; lanes 6 and 7, PCR of *ppgA* from cDNA from 4 and 8 days growth, respectively; lane 8, PCR of *preA* from genomic DNA; lanes 9 and 10, PCR of *preA* from cDNA from 4 and 8 days growth, respectively; lane 11, PCR of *preB* from genomic DNA; lanes 12 and 13, PCR of *preB* from cDNA from 4 and 8 days growth, respectively; lane 14, PCR of *actA* from genomic DNA; lanes 15 and 16, PCR of *actA* from cDNA from 4 and 8 days growth, respectively. Note that the presence of a smaller size amplicon from the cDNA template indicates the excision of an intron.
