# Supplementary material for: Comparative genomics reveals high biological diversity and specific adaptations in the industrially and medically important fungal genus Aspergillus
Source: Genome Biol. 2017 Feb 14;18:28. doi: 10.1186/s13059-017-1151-0 (PMC5307856; doi:10.1186/s13059-017-1151-0)
Supplement: Additional file 6: — Overview of number of putative orthologs/paralogs in the compared fungal species related to primary carbon metabolism. (PDF 237 kb) [file 13059_2017_1151_MOESM6_ESM.pdf]

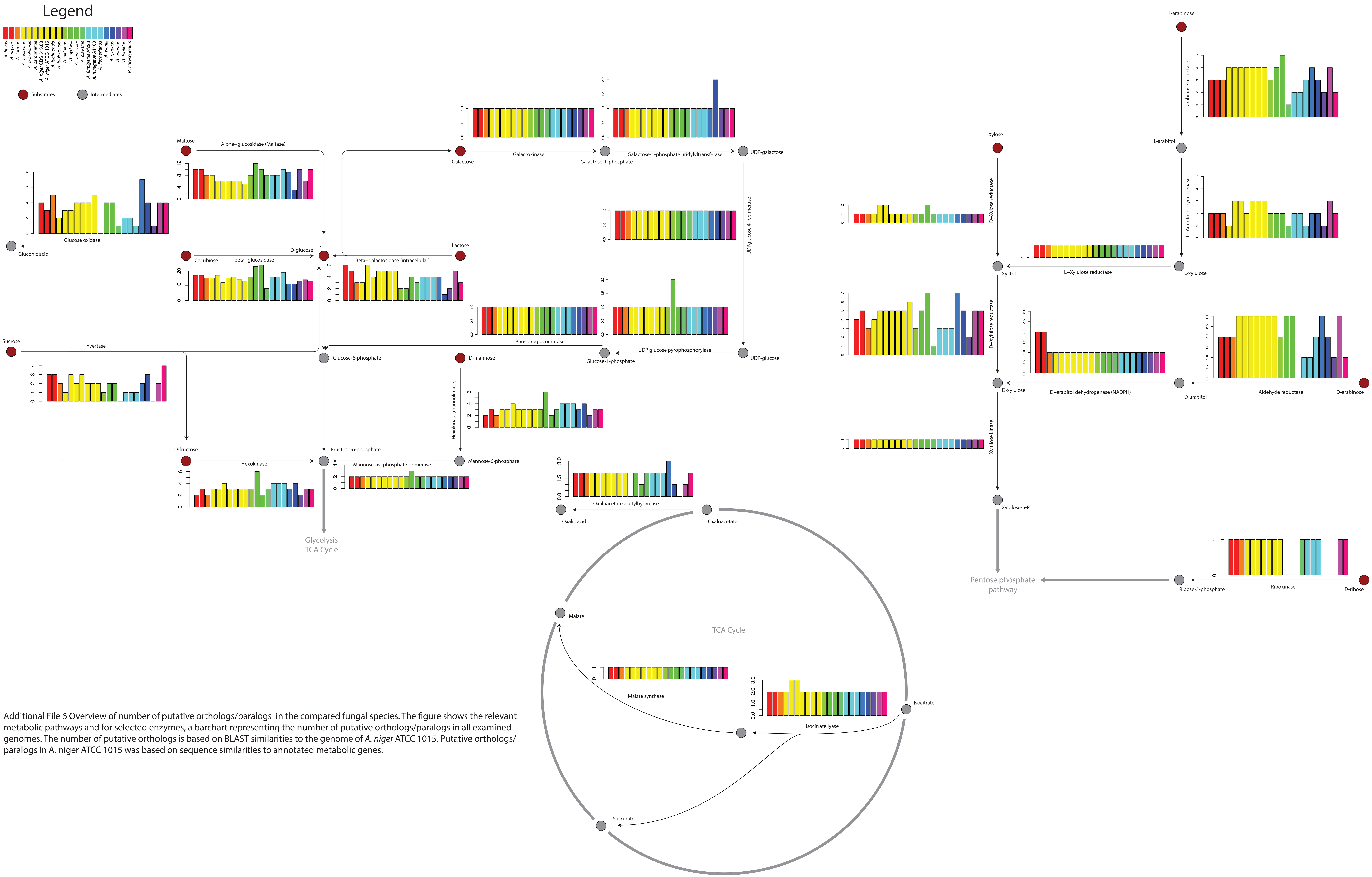

Additional File 6 Overview of number of putative orthologs/paralogs in the compared fungal species. The figure shows the relevant metabolic pathways and for selected enzymes, a barchart representing the number of putative orthologs/paralogs in all examined genomes. The number of putative orthologs is based on BLAST similarities to the genome of *A. niger* ATCC 1015. Putative orthologs/ paralogs in A. niger ATCC 1015 was based on sequence similarities to annotated metabolic genes.
