# Supplementary material for: Comparative genomics reveals high biological diversity and specific adaptations in the industrially and medically important fungal genus Aspergillus
Source: Genome Biol. 2017 Feb 14;18:28. doi: 10.1186/s13059-017-1151-0 (PMC5307856; doi:10.1186/s13059-017-1151-0)
Supplement: Additional file 7: — Comparative growth profiling of the aspergilli and selected other fungi. (PDF 8.65 kb) [file 13059_2017_1151_MOESM7_ESM.pdf]

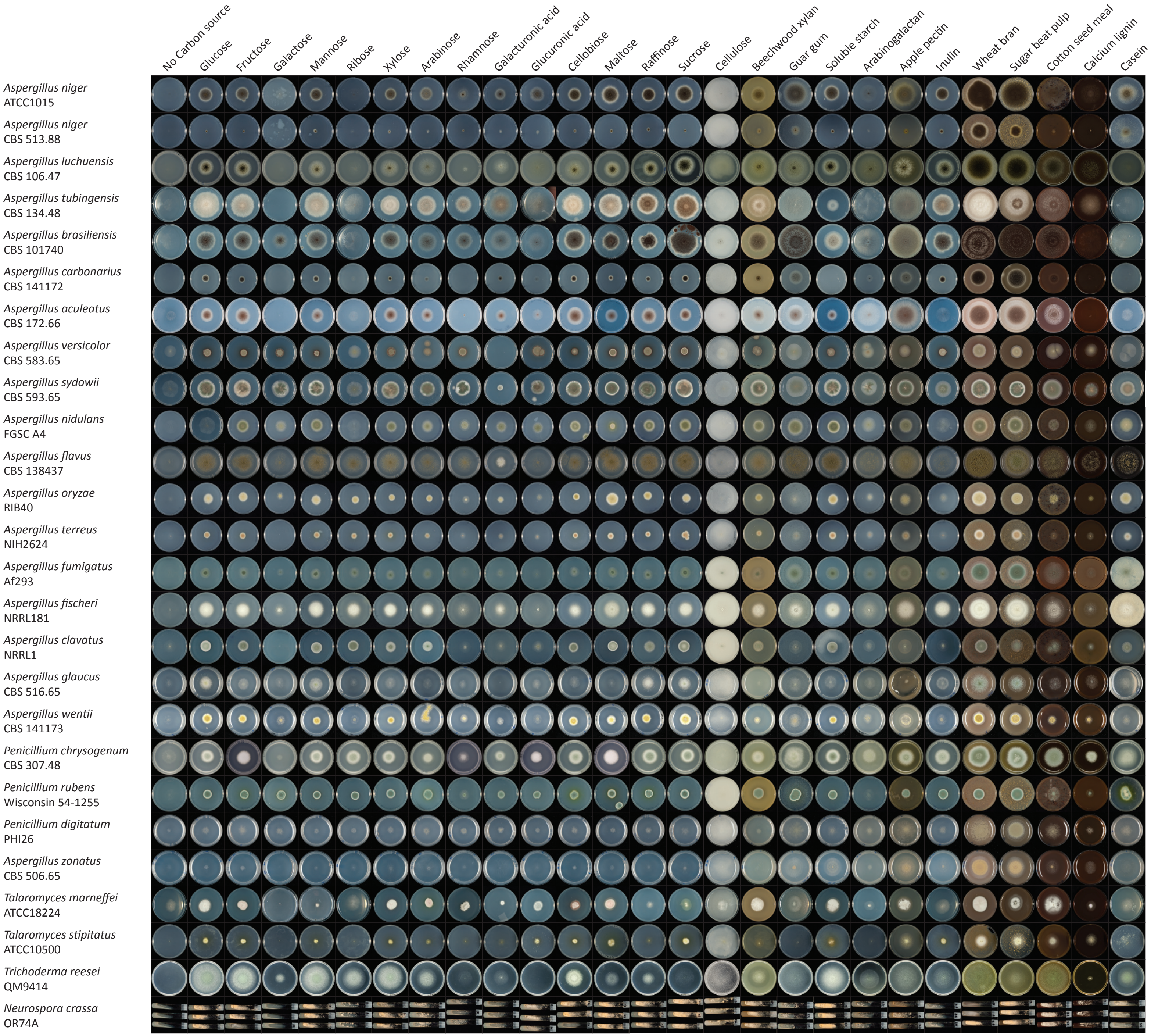

Additional File 7. Comparative growth profiling of the aspergilli and selected other fungi. All monosaccharides and oligosaccharides were added at 25 mM concentrations, all pure polysaccharides at 1% (w/v) and all crude carbon sources at 3% (w/v). No carbon source, casein (1% w/v) and lignin (1% w/v) were included as controls.
