# Supplementary material for: Comparative genomics reveals high biological diversity and specific adaptations in the industrially and medically important fungal genus Aspergillus
Source: Genome Biol. 2017 Feb 14;18:28. doi: 10.1186/s13059-017-1151-0 (PMC5307856; doi:10.1186/s13059-017-1151-0)
Supplement: Additional file 8: — Effect of SHAM and KCN on fungal growth and sporulation. (PDF 252 kb) [file 13059_2017_1151_MOESM8_ESM.pdf]

Additional File 8. Effect of SHAM and KCN on fungal growth and sporulation

Species	Control	SHAM	KCN	SHAM/KCN	Sporulation
A. niger N402	Very good	Very good	Very good	Very good	Sporulation
A. niger CBS 513.88	Good	Good	Good	Good	Sporulation
A. niger CBS 113.46	Very good	Very good	Very good	Very good	Sporulation
A. luchuensis	Very good	Average	Very good	Weak	SHAM delayed sporulation
A. tubingensis	Very good	Average	Very good	Less than average	SHAM and KCN no sporulation
A. brasiliensis	Very good[1]	Good[2]	Very good	Average[3]	SHAM no sporulation
A. carbonarius	Very good	Good	Very good	Average	Sporulation
A. aculeatus	Good	Good	Good	Good	SHAM no sporulation
A. versicolor	Average	Weak	Average	Weak	No sporulation
A. sydowii	Average	Weak	Average	Weak	SHAM and KCN no sporulation
A. nidulans	Very good	Good	Very good	Weak[4]	SHAM no sporulation
A. oryzae	Very good	Weak	Very good	Weak	SHAM no sporulation
A. terreus	Very good	Weak	Good	Weak	SHAM and KCN no sporulation
A. fischeri	Very good	Weak	Very good	Weak	No sporulationl
A. clavatus	Very good	Weak	Very good	Very weak[5]	SHAM and KCN no sporulation
A. glaucus	Average	No growth	Very weak	No growth	No sporulation
A. wentii	Average	Weak	Average	Weak	SHAM and KCN No sporulation
A. zonatus	Very good	Average	Very good	Weak	No sporulation

[1]Very good: growth between 65-85 mm (diameter)
[2]Good: growth between 50-65 mm (diameter)
[3]Average: 35-50 mm (diameter)
[4]Weak: 20-35 mm (diameter)
[5]Very weak: 10-20 mm (diameter)
