# Supplementary material for: Comparative genomics reveals high biological diversity and specific adaptations in the industrially and medically important fungal genus Aspergillus
Source: Genome Biol. 2017 Feb 14;18:28. doi: 10.1186/s13059-017-1151-0 (PMC5307856; doi:10.1186/s13059-017-1151-0)
Supplement: Additional file 13: — Molecular phylogenetic tree of CYP56 family (A), and CPR2 and CYP630 family (B). (PDF 402 kb) [file 13059_2017_1151_MOESM13_ESM.pdf]

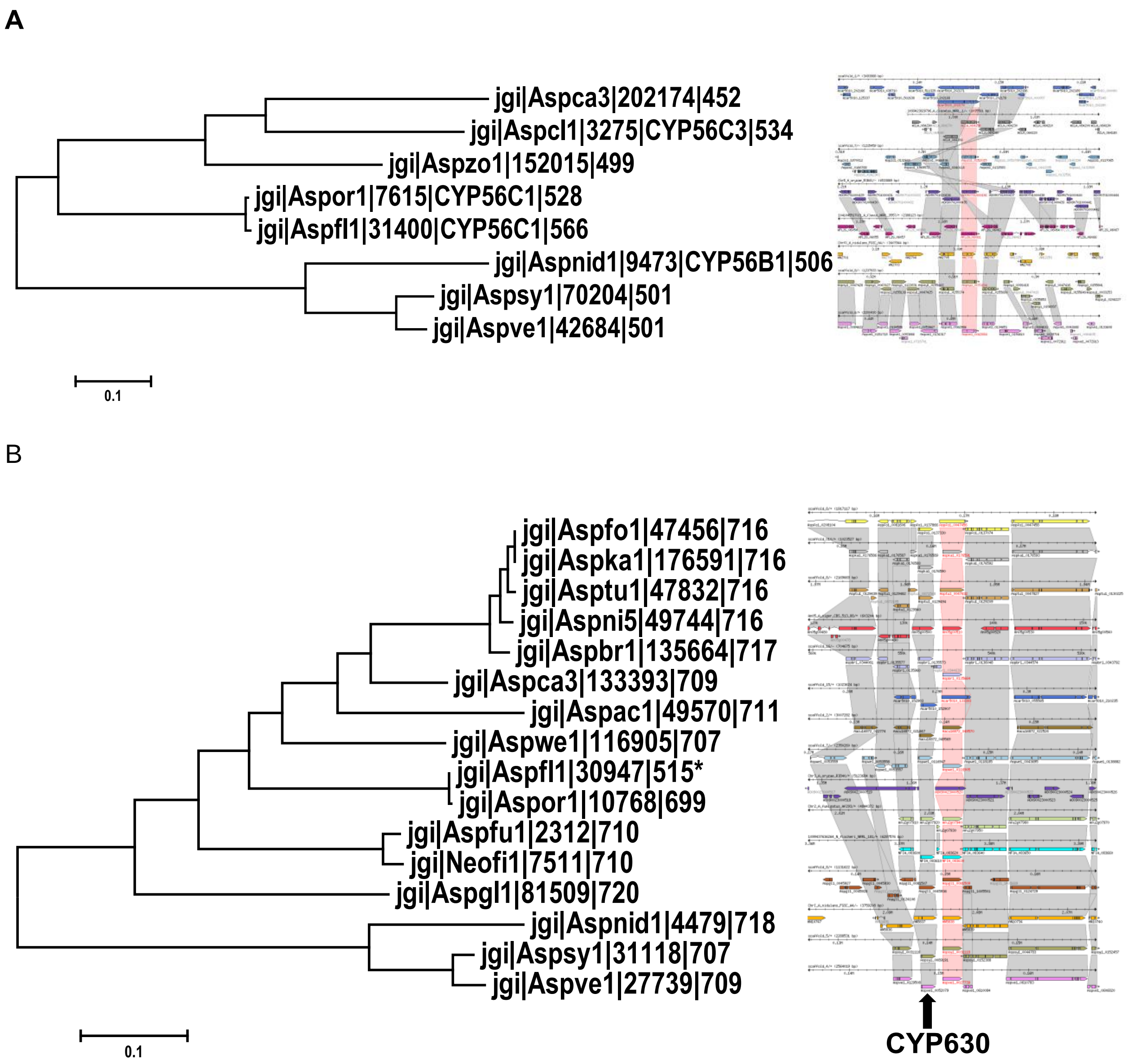

Additional File 13. Molecular phylogenetic tree of CYP56 family (A), and CPR2 and CYP630 family (B)

Evolutionary analyses were conducted in MEGA7 [1]. The analysis involved 8 (A) and 16 (B) amino acid sequences, respectively. Following Muscle alignment the evolutionary history was inferred by using the Maximum Likelihood method based on the JTT matrix-based model [2]. There were a total of 436 (A) and 505 (B) positions in the final datasets, respectively. The tree is drawn to scale, with branch lengths measured in the number of substitutions per site. All positions containing gaps and missing data were eliminated. The last number of the name represents the size of the protein. CYP names were assigned according to the CYP nomenclature (http://drnelson.uthsc.edu/fungal.genomes.html). Regions of collinearity in the genomes are represented by AspGD Sybil tool [3], proteins are ordered following the phylogenetic tree. In (A), CYP56 orthologues are marked in red lines and other collinear proteins

in grey lines. In (B), CPR2 orthologues are marked in red lines, the collinear proteins in grey lines, and an arrow is pointing CYP630 orthologues. If protein size is marked by a star is the sequence incomplete.

References

1. Kumar S, Stecher G, Tamura K: **MEGA7: Molecular Evolutionary Genetics Analysis version 7.0 for bigger datasets.** *Mol Biol Evol* 2015, **33:**1870–1874.
2. Jones DT, Taylor WR, Thornton JM: **The rapid generation of mutation data matrices from protein sequences.** *Comput Appl Biosci* 1992, **8:**275-282.
3. Arnaud MB, Cerqueira GC, Inglis DO, Skrzypek MS, Binkley J, Chibucos MC, Crabtree J, Howarth C, Orvis J, Shah P, et al: **The Aspergillus Genome Database (AspGD): recent developments in comprehensive multispecies curation, comparative genomics and community resources.** *Nucleic Acids Res* 2012, **40:**D653-659.
