# Supplementary material for: Comparative genomics reveals high biological diversity and specific adaptations in the industrially and medically important fungal genus Aspergillus
Source: Genome Biol. 2017 Feb 14;18:28. doi: 10.1186/s13059-017-1151-0 (PMC5307856; doi:10.1186/s13059-017-1151-0)
Supplement: Additional file 14: — Molecular phylogenetic tree of CYP6001 - CYP6003 families (PpoA - PpoD) and colinearity of the genetic surroundings. (PDF 279 kb) [file 13059_2017_1151_MOESM14_ESM.pdf]

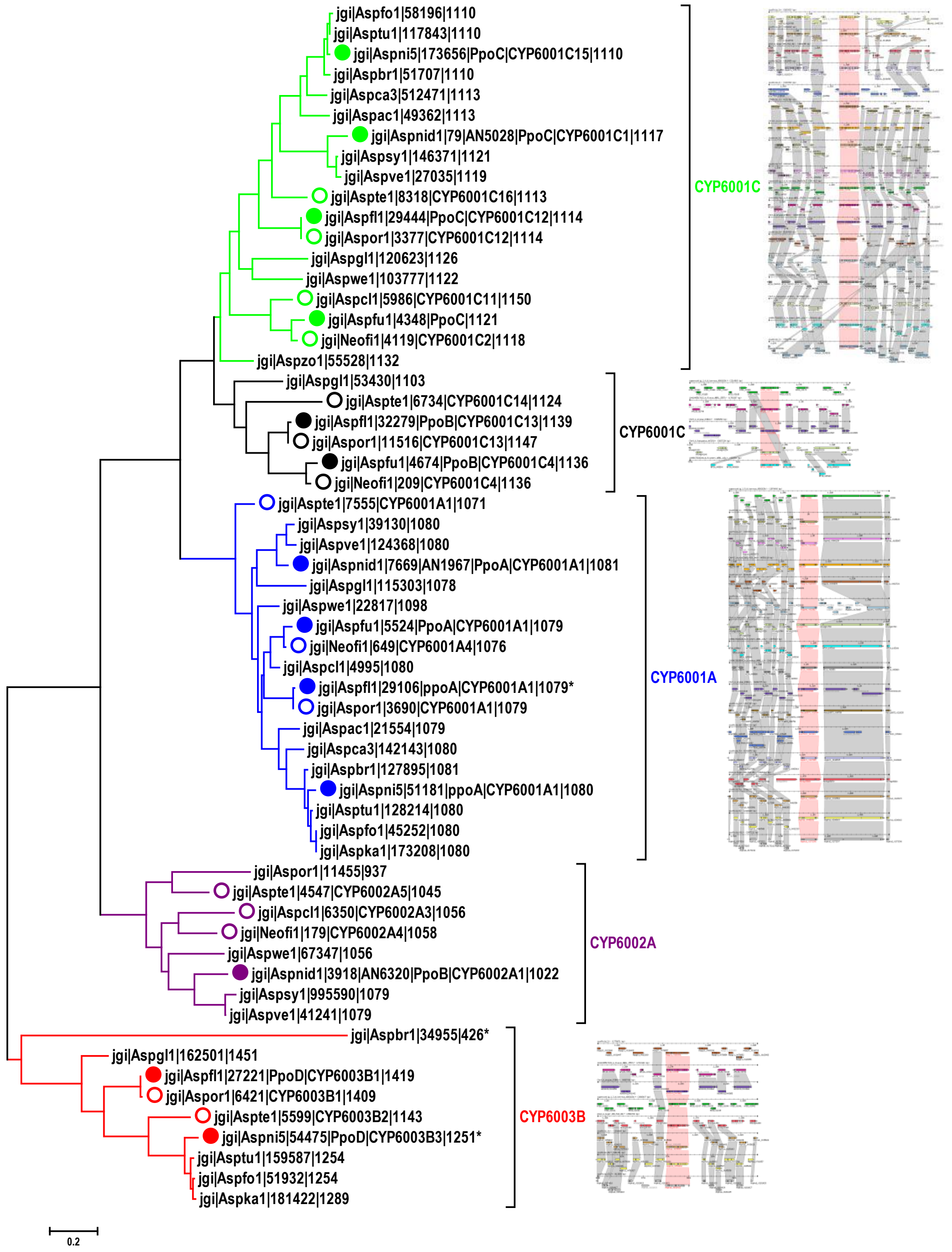


**Additional File 14. Molecular phylogenetic tree of CYP6001 - CYP6003 families (PpoA - PpoD) and colinearity of the genetic surroundings.**

Evolutionary analyses were conducted in MEGA6 [1]. The analysis involved 59 amino acid sequences. If protein size is marked by a star was the sequence manually curated or sequence is incomplete. Following Muscle alignment the evolutionary history was inferred by using the Maximum Likelihood method based on the JTT matrix-based model. There were a total of 183 positions in the final dataset. The tree is drawn to scale, with branch lengths measured in the number of substitutions per site. All positions containing gaps and missing data were eliminated. The last number of the name represents the size of the protein. For open circle marked sequences CYP names were assigned according to the CYP nomenclature (http://drnelson.uthsc.edu/fungal.genomes.html), for full circle marked sequences function were ascribed in the

literature. Regions of colinearity in all genomes are represented by AspGD Sybil tool [2], proteins are ordered following the phylogenetic tree, orthologues are marked in red lines, other collinear proteins in grey lines. For CYP6002A subfamily no AspGD ortholog cluster is available.

References


1. Tamura K, Stecher G, Peterson D, Filipski A, Kumar S: **MEGA6: Molecular Evolutionary Genetics Analysis version 6.0.** *Mol Biol Evol* 2013, **30:**2725-2729.
2. Arnaud MB, Cerqueira GC, Inglis DO, Skrzypek MS, Binkley J, Chibucos MC, Crabtree J, Howarth C, Orvis J, Shah P, et al: **The Aspergillus Genome Database (AspGD): recent developments in comprehensive multispecies curation, comparative genomics and community resources.** *Nucleic Acids Res* 2012, **40:**D653-659.
