# Supplementary material for: Comparative genomics reveals high biological diversity and specific adaptations in the industrially and medically important fungal genus Aspergillus
Source: Genome Biol. 2017 Feb 14;18:28. doi: 10.1186/s13059-017-1151-0 (PMC5307856; doi:10.1186/s13059-017-1151-0)
Supplement: Additional file 15: — Extended analysis of stress tolerance of the aspergilli. (PDF 706 kb) [file 13059_2017_1151_MOESM15_ESM.pdf]

Additional file 15. Extended analysis of stress tolerance of the aspergilli (1)

Authors in alphabetic order: Ronald P. de Vries[1,2], Tamás Emri[3], Zsolt Karányi[4], Márton Miskei[3,5], Erzsébet Orosz[1,3], István Pócsi[3], Vincent Robert[1], Nathalie van de Wiele[1], Miaomiao Zhou[1,2]

Affiliations:
[1]CBS-KNAW Fungal Biodiversity Centre, Uppsalalaan 8, 3584 CT Utrecht, The Netherlands; [2]Fungal Molecular Physiology, Utrecht University, Uppsalalaan 8, 3584 CT Utrecht, The Netherlands; [3]Department of Biotechnology and Microbiology, Faculty of Science and Technology, University of Debrecen, Egyetem tér 1., H-4032 Debrecen, Hungary; [4]Department of Medicine, Faculty of Medicine, University of Debrecen, Nagyerdei krt. 98., H-4032 Debrecen, Hungary; [5]MTA-DE Momentum, Laboratory of Protein Dynamics, Department of Biochemistry and Molecular Biology, University of Debrecen, Nagyerdei krt.98., H-4032 Debrecen, Hungary

The aspergilli represent a versatile group of fungi with enormous environmental, industrial and biomedical significance. Similar to other fungi, the aspergilli have to cope with various types of environmental stress meanwhile they occupy their highly divergent habitats [1, 2]. A deeper understanding of the evolution and organization of their complex and robust stress defense systems may give us suitable tools to control their growths and metabolite productions, which may subsequently lead to the development of new, stress-tolerant industrial *Aspergillus* strains with increased yields or to the development of novel antifungal drugs and strategies [1].

For the construction of stress databases, 18 *Aspergillus* strains with fully sequenced fungal genomes representing 17 species were investigated. *A. niger* was represented by two strains. For the production of conidia, normally the malt extract – mycological pepton sporulation agar medium (1.5 % agar) recommended by CBS-KNAW (for more detailed description of culture conditions visit the website http://www.fung-stress.org/) was used. The sporulation medium for *A. glaucus* was also supplemented with 1.0 M NaCl. Unless otherwise indicated, asexual sporulation was carried out at 25°C in the dark for 6 days. *A. fumigatus* and *A. nidulans* also sporulated on the CBS-KNAW medium (http://www.fung-stress.org/) at 37°C and on nitrate minimal medium agar plates (NMM, solidified with 1.8 % agar, [3]) at 37°C. As recommended by Barratt [3], we always added one ml Hutner's trace element solution (http://www.fgsc.net/methods/anidmed.html) to one liter culture medium. Microbial growth and stress sensitivity assays were performed on NMM agar plates (1.8 % VWR International Agar powder for bacteriology, ID number 20 767.298), which were incubated at 25 (5 or 10 d) or 37°C (5 d) in the dark and were supplemented with stress-initiating agents like H_2O_2 (oxidative stress), menadione sodium bisulfite (MSB, oxidative stress), NaCl (ionic osmotic stress), sorbitol (non-ionic osmotic stress), Congo Red (cell wall integrity stress) or $CdCl_2$ (heavy metal stress) as required. Stress agar plates were always point-inoculated by 10^5 freshly grown conidia washed and suspended in 5 µl 0.9 % NaCl, 0.01 % Tween 80 as described elsewhere [4]. After completing the physiological experiments, colonies were photographed, and fungal growth was characterized by colony diameters [4] or by scoring them by eye on a 0-10 scale (Additional File 3). Stress sensitivities were quantified by % decreases in the colony diameters in comparison to controls or by decreases in the growth scores presented on a 0-1 scale (Additional File 3). Physiological data were collected in the Fungal Stress Database (FSD) at

CBS-KNAW (http://www.fung-stress.org/). Because *A. niger* CBS 513.88 did not grow well on NMM agar and *A. zonatus* CBS 506.65 was not able to use either glucose or oat bran effectively as a carbon source these strains were not included in the construction of stress databases.

Stress response proteins were identified and annotated in the newly sequenced *Aspergillus* species following the procedure described previously by Karányi et al. [5], and newly identified orthologs were inserted into the Fungal Stress Response Database version 2 (FSRDv_2), which is available at http://internal.med.unideb.hu/fsrd2/?p=consortium. The phylogeny of selected stress response proteins and their orthologs was also studied. Orthologs of selected stress response proteins were collected after blastp search carried out in AspGD (*Aspergillus* Genome Database; http://www.aspergillusgenome.org/) and in JGI (Joint Genome Institute) MycoCosm (http://genome.jgi.doe.gov/pages/blast-query.jsf?db=eurotiomycetes) and protein domain analysis performed using SMART (Simple Modular Architecture Research Tool; http://smart.embl-heidelberg.de/). Following that, ClustalW (http://www.ebi.ac.uk/clustalw/index.html) and BioEdit Sequence Alignment Editor [6] programs were employed to compare the translated protein sequences. Phylogenetic and molecular evolutionary calculations were conducted using MEGA version 5.0 using Maximum likelihood method in the WAG or Jones-Taylor-Thornton (JTT) substitution model [7]. Bootstrap tests were performed with 500 replications.

Considering the outcomes of the stress physiology experiments (Additional File 3), the growth patterns of the 17 *Aspergillus* species recorded at 25 and 37°C incubation times were quite different; meanwhile *A. fischeri* (*Neosartorya fischeri*), *A. luchuensis* (*A. acidus*) and *A. nidulans* were the fastest growing spp. at 37 ^{o}C (5 d incubation time) *A. brasiliensis*, *A. fischeri* and *A. tubingensis* grew the best at 25°C (10 d incubation time) under unstressed conditions. Interestingly, the addition of 2.0 M sorbitol to NMM stimulated the growth of *A. wentii*, *A. sydowii* and *A. versicolor* at 37°C where they normally hardly grow. It is noteworthy that the most osmophilic *A. glaucus* did not grow at 37°C even in the presence of this sugar alcohol. Sorbitol also increased the growth rates of *A. glaucus*, *A. wentii* and *A. versicolor* at 25 ^{o}C and that of *A. oryzae* at 37°C. The supplementation of NMM agar with 1.0 M NaCl increased the growths of *A. sydowii*, *A. wentii* and *A. versicolor* at 37°C and also those of *A. glaucus*, *A. wentii* and *A. versicolor* at 25 ^{o}C. It is remarkable that the addition of NaCl to *A. glaucus* cultures grown on NMM at 25 ^{o}C also provided the osmophilic fungus with Congo Red resistance (Additional File 3).

The aspergilli studied showed quite versatile stress tolerance fingerprints with some outstanding features. It is noteworthy that incubation temperature (25 *vs.* 37°C), incubation time (5 *vs*. 10 d at 25°C) and sporulation conditions (malt medium *vs.* NMM and 25 *vs.* 37°C) all influenced the observed stress tolerances, and the inherent stress tolerances of the species also varied within quite wide ranges. It is reasonable to assume that incubation at 37°C itself is quite stressful for some species (*A. glaucus*, *A. sydowii*, *A. versicolor*, *A. wentii*) meanwhile other species like *A. fischeri*, *A. luchuensis* and *A. nidulans* can adapt more easily to this temperature. Stress tolerance rankings (Additional File 3) changed significantly when the incubation time was increased from 5 to 10 d at 25°C, which is indicative of the onset of stress adaptation processes (*e.g.* in the case of *A. versicolor* when exposed to MSB). The composition of the sporulation medium and the sporulation temperature had a significant impact on the oxidative stress tolerances of *A. fumigatus* and *A. nidulans* because the malt

extract-mycological peptone sporulation medium and the elevation of the sporulation temperature from 25 to 37°C increased the MSB tolerances of these fungi. Furthermore, sporulation at 37°C was beneficial for *A. nidulans* when it was exposed to H_2O_2 (Additional File 3).

The construction of the FSD (currently contains 1497 colony pictures) and FSRDv_2 {incorporates 43725 stress response protein orthologs found in 39 fungal species characterized with 41 fully sequenced genomes (17 *Aspergillus* species with 3 genomes available for *A. niger*)} gave us a unique wealth of information to map the differences and possible connections between the physiological properties and the stress response systems of selected aspergilli. The large-scale comparison of physiological and proteome data revealed novel and important species-specific stress tolerance/sensitivity phenotypes and shed light on the corresponding proteome-level differences in the stress response systems of the fungi.

One of the most outstanding observations was that *A. fumigatus*, the effective opportunistic parasite of humans, was remarkably sensitive to oxidative, osmotic and call wall integrity stress meanwhile it possessed an exceptionally high Cd(II) tolerance (FSD; Additional File 3). The latter phenotype was explained by the Pca1-type cadmium transporter of the fungus (Fig. S1) and, maybe in part, by its novel group cIII CatC-type catalase (Fig. S2). Pca1 encodes a cadmium transporting P-type ATPase, which exports toxic Cd(II) in *S. cerevisiae* [8]. It is possible that Pca1 first appeared in the common ancestor of Pezizomycotina. As shown in Figure 1, Pca1 is absent in some Pezizomycotina species (*A. fischeri*, *A. carbonarius*, *A. tubingensis, A. aculeatus*, *A. luchuensis*, *A. brasiliensis*, *A. niger*, *A. nidulans*, *A. glaucus*, *Coccidiodies posadasii*, *Sclerotinia sclerotiorum*). The presence of this transporter is consistent with the occurrence of the Cd(II) tolerance in the aspergilli because *A. sydowii*, *A. fumigatus*, *A. wentii*, *A. terreus* and *A. versicolor*, all of which harbor Pca1 orthologue(s), are among the most Cd(II) tolerant *Aspergillus* spp. Meanwhile *A. carbonarius, A. glaucus* and *A. aculeatus*, none of which has a Pca1 ortholog, are among the most Cd(II) sensitive aspergilli. Importantly, *A. sydowii* with highest Cd(II) tolerance recorded after 10 d incubation at 25°C uniquely possesses two Pca1 orthologs. Nevertheless, the outstanding Cd(II) tolerance of *A. fumigatus* may be attributed not only to the presence of Pca1 but also to its group cIII CatC catalase (Fig. S2). Interestingly, the appearance of the Pca1-type pump in *S. cerevisiae* and in *Saccharomyces kudriavzevii* can only be explained by a horizontal gene transfer event (Fig. S1).

The presence of the Pca1-type transporter was consistent with the occurrence of the Cd(II) tolerance in the aspergilli in general because *A. sydowii*, *A. fumigatus*, *A. wentii*, *A. versicolor* and *A. terreus*, all of which harbor Pca1 orthologue(s) (Fig. S1), are among the most Cd(II) tolerant *Aspergillus* spp. (FSD; Additional File 3). Meanwhile, *A. carbonarius, A. glaucus* and *A. aculeatus*, none of which has a Pca1 ortholog (Fig. S1), are among the most Cd(II) sensitive aspergilli (FSD; Additional File 3). Further research is needed to estimate the biomedical significance of these findings.

A

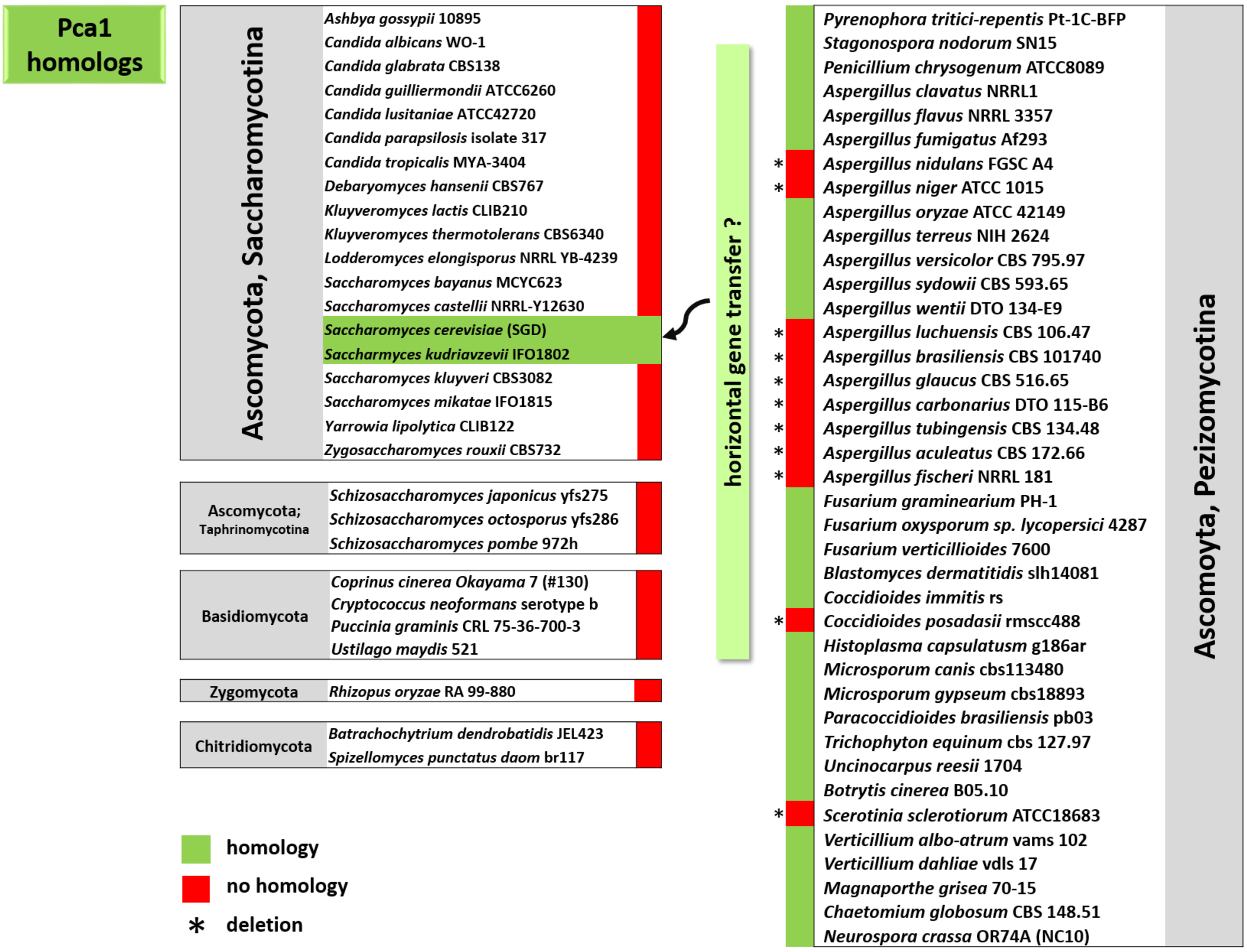

B

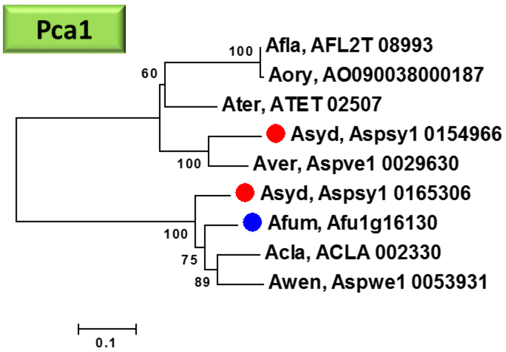

Figure S1. Distribution of Pca1 homologs in the Fungal Kingdom including the studied *Aspergillus* species. Pca1 encodes a transporter, which exports toxic Cd(II) in *S. cerevisiae*. In Part A, red color and black stars indicate the absence of Pca1 homologs. In Part B, the dendrogram of Pca1 orthologs in the aspergilli is presented. Pca1 orthologs found in various *Aspergillus* species are indicated by four letters species name identifiers (Table S1) and locus IDs found in AspGD. (The relevant JGI locus IDs are listed in Additional File 16.) Please note that *A. sydowii* harbors two Pca1 orthologs (red circles). Blue circle marks the Pca1 ortholog in *A. fumigatus*.

Table S1. List of four letters species name identifiers used in this study

Aacu – *Aspergillus aculeatus* (ATCC 18872)	Anid – *Aspergillus nidulans* (FGSCA4)
Abra – *Aspergillus brasiliensis* (CBS 101740)	Anig – *Aspergillus niger* (CBS 513.88)
Acar – *Aspergillus carbonarius* (ITEM 5010)	Aory – *Aspergillus oryzae* (Rib40)
Acla – *Aspergillus clavatus* (CBS 513.65 = NRRL1)	Asyd – *Aspergillus sydowii*
Afis – *Aspergillus fischeri* (NRRL 181)	Ater – *Aspergillus terreus* (NIH2624)
Afla – *Aspergillus flavus* (CBS 128202 = NRRL 3357)	Atub – *Aspergillus tubingensis* (CBS 134.48)
Afum – *Aspergillus fumigatus* (Af293)	Aver – *Aspergillus versicolor*
Agla – *Aspergillus glaucus* (CBS 516.65)	Awen – *Aspergillus wentii* (DTO 134-E9)
Aluc – *Aspergillus luchuensis* (CBS 106.47)	Scer – *Saccharomyces cerevisiae* (S288C)

In this table, strain designations shown in AspGD are presented.

CatC is a catalase with a putative peroxisomal localization [9]. The *catC* gene displayed a relatively constant level of expression, not being induced by oxidative or other types of stress in *A. nidulans* [9]. However, higher concentrations of Cd(II) caused a small increase in total catalase activity but there was a major increase in only one (most likely CatC) isoenzymic form in this fungus [10]. In contrast, Cd(II) treatment eliminated completely the catalase activity in *A. niger* [11]. Therefore, the inducibility/resistance of the available catalases by/to Cd(II) is likely an important factor in combating Cd(II) stress.

We have managed to find six (cI-cVI) separated subgroups of catalase C in the fungal species studied. The well-known CatC candidates are present in group cIV. There are no such candidates in *A. fumigatus*, *A. fischeri* and *A. clavatus*, which species should have a common ancestor where the deletion of the catalase C (group cIV) happened. Group cIII contains one-one candidate from the species *A. fumigatus* (Afu2g18030) and *A. carbonarius*.
In our opinion, group cIII reflects an ancient duplication event, and the proteins we see today in this group likely gained a function which may be different from those of catalase Cs. More recently, the involvement of the group cIII catalase of *A. fumigatus* (named as EasC) in the synthesis of the ergot alkaloid chanoclavine-I and also its ability to decompose H_2O_2 have been demonstrated by Goetz et al. [12]. Further studies are needed to estimate the H_2O_2 decomposing capability as well as the inducibility by and the sensitivity to Cd(II) of group cIII and group cIV catalase C isoenzymes in the aspergilli. Considering that *A. fumigatus* lacks a group cIV catalase C (meanwhile *A. carbonarius* has got one) but it has one group cIII enzyme, these observations may explain, at least in part, the relatively weak H_2O_2 tolerance and the exceptionally high Cd(II) tolerance observable in the important opportunistic human pathogen *A. fumigatus*.

Catalases

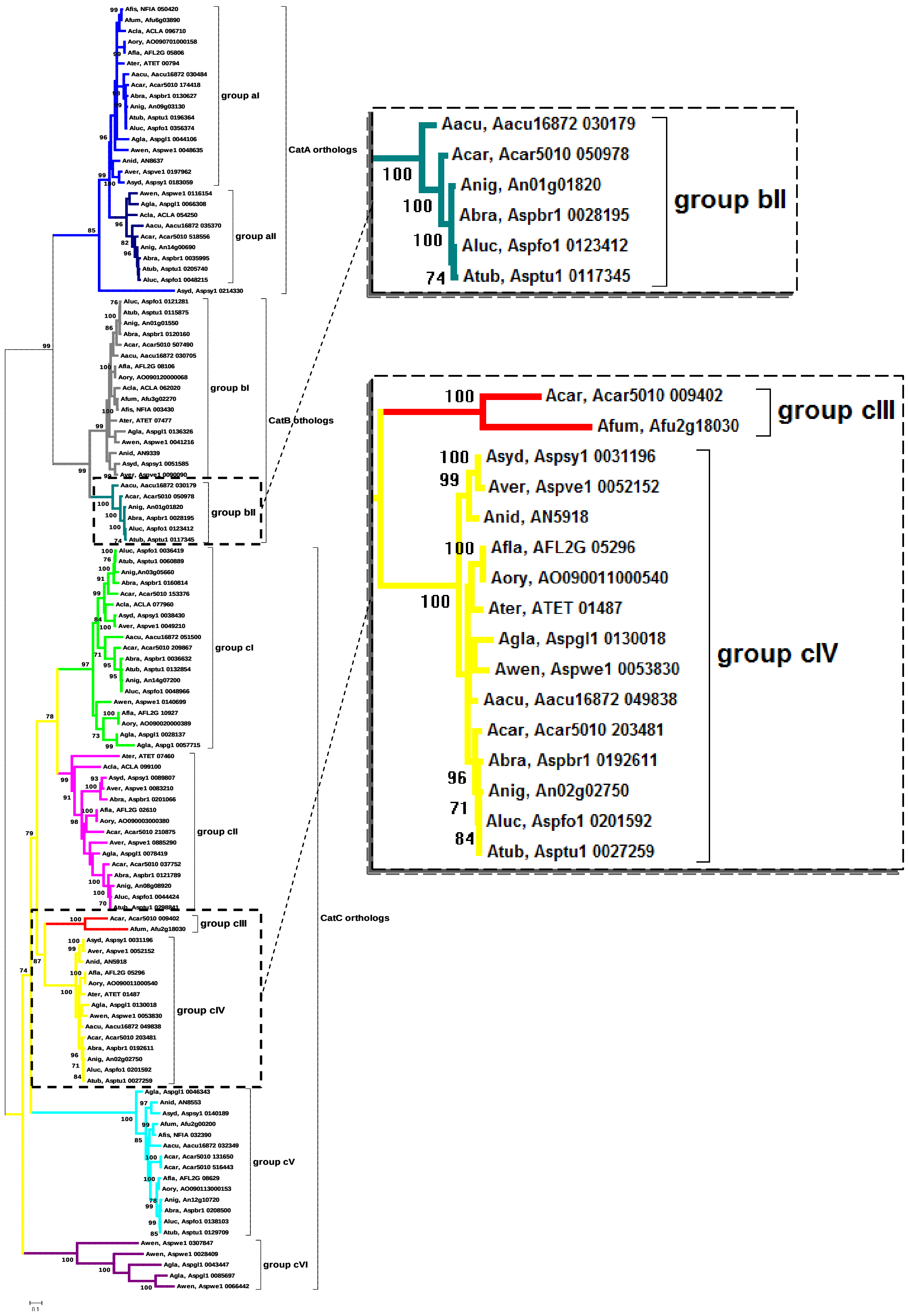

Figure S2. Dendrogram of catalases in *Aspergillus* species. Catalase orthologs are indicated by four letters species name identifiers (Table S1) and locus IDs found in AspGD. (The relevant JGI locus IDs are listed in Additional File 16.)

In addition, a new-type catalase was identified which has phylogenetic relation to catalase Bs and was therefore designated as group bII in this study. There are candidates in this cluster from six species (*A. carbonarius*, *A. aculeatus*, *A. niger*, *A. brasiliensis*, *A. luchuensis*, *A. tubingensis*). Nevertheless, the existence of this putative new-type of catalase does not seem to correlate the H_2O_2 stress tolerance of the aspergilli studied.

Orthologs of well-characterized catalases, *e.g.* CatA and CatB from *A. nidulans*, were collected after blastp search and protein domain analysis. CatA is a developmentally regulated catalase in *A. nidulans*, which is crucial in the oxidative stress defense of conidia [13] meanwhile CatB is the predominant catalase in growing hyphae [14].

Another remarkable outcome of the comparative physiology studies was that osmophility among the aspergilli seems to be much more wide-spread than we thought before. Osmophilic aspergilli now include *A. glaucus*, *A. wentii*, *A. versicolor*, *A. sydowii* and *A. oryzae* (FSD; Additional File 3). In the case of *A. glaucus* and *A. wentii*, we managed to couple the osmophility of these species to the lack of their GfdB NAD^+-dependent glycerol-3-phosphate dehydrogenase ortholog (Fig. S3). Further studies should aim at the insertion of a functional *gfdB* gene into the *A. glaucus* and *A. wentii* genomes and/or the deletion of this type of NAD^+-dependent glycerol-3-phosphate dehydrogenase from other aspergilli to screen the changes in their osmophility/osmosensitivity. It is important to note that the elimination of *gfdA* in *A. nidulans* has resulted in an osmoremediable phenotype on various carbon sources but not on glycerol as shown by Fillinger et al. [15].

There are two Gpd1/2 orthologs in the majority of the species placed in the Eurotiomycetes taxon, which are designated as “GfdA” and “GfdB” [15, 16]. Interestingly one of them (“GfdB”) is absent in two species, *A. glaucus* and *A. wentii*. It is possible that there was a *gpd* gene deletion/degradation in the ancestor of these species. Importantly, the two species not harboring any GfdB (osmotic stress responsive) ortholog are osmophilic. This finding is not surprising because the elimination of GfdA in *A. nidulans* also resulted in an osmoremediable (in the presence of 1 M NaCl) phenotype when the mutant was grown on glucose, fructose or ethanol but not on glycerol [15]. The abnormal morphology of the mutant hyphae was reminiscent of mutants defected in cell wall biosynthesis [15]. Although no literature data is available for the phenotypic characterization of and *gfdB* gene deletion mutant the gene is under HogA control in osmotic stress exposed *A. nidulans* [16]. Therefore, a physiological function similar to that of GfdA is foreseeable for GfdB but this hypothesis needs experimental verification.

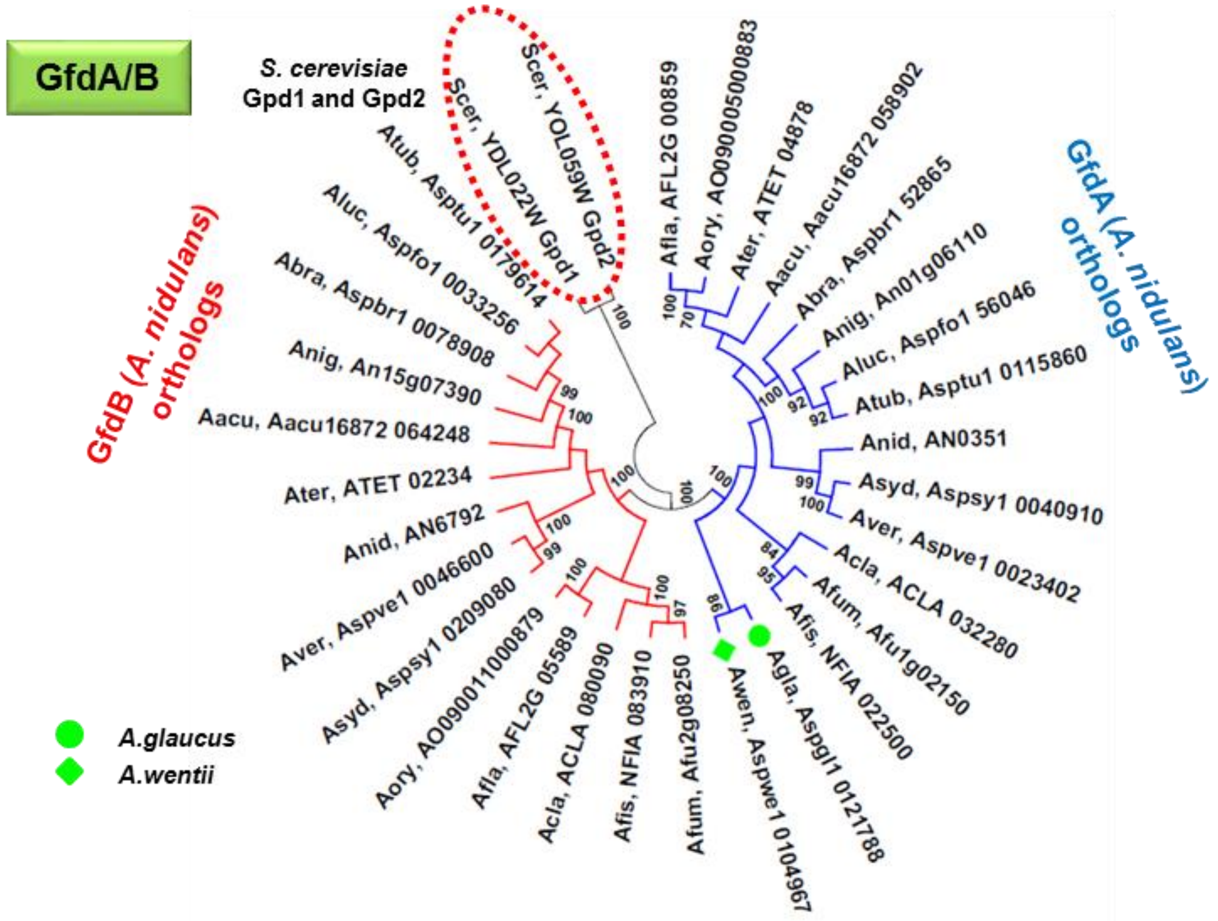

Figure S3. Dendrogram of the *S. cerevisiae* Gpd1/2 paralogs and the *A. nidulans* GfdA/B (NAD$^+$-dependent glycerol-3-phosphate dehydrogenases) orthologs in the studied *Aspergillus* species. GfdA/B orthologs are indicated by four letters species name identifiers (Table S1) and locus IDs as found in AspGD for the aspergilli. The relevant JGI locus IDs are listed in Additional File 16. The Gpd1/2 paralogs of budding yeast were taken from the *Saccharomyces* Genome Database (http://www.yeastgenome.org/).

Interestingly, likely the lack of a group cIV type CatC (Fig. S2) resulted in a H_2O_2 sensitive phenotype in *A. fumigatus* (FSD; Additional File 3) meanwhile the duplication of Pca1 cadmium transporter (Fig. S1) gave a highly Cd(II) tolerant phenotype to *A. sydowii* (FSD; Additional File 3). There are some data indicating that the species specific appearance of new elements in the antioxidative defense system of the aspergilli might either enhance the oxidative stress tolerance of the fungi, *e.g.* two new SodAs (Fig. S4) gave *A. brasiliensis* an exceptional tolerance to MSB (FSD; Additional File 3), or be neutral (FSD; Additional File 3) in terms of antioxidative potential, *e.g.* the appearance of a new-type catalase with a phylogenetic relation to CatB in several aspergilli (designated as group bII; Fig. S2). Of course, further genetic and physiological studies are needed to verify the physiological functions of these putative new-type antioxidant enzymes.

SodA is the Cu/Zn-superoxide dismutase of *A. nidulans*, which is induced under iron starvation and is repressed under copper starvation [17]. In addition, SodB and SodM are manganese-superoxide dismutases in the aspergilli [18, 19]. The outstanding menadione tolerance of *A. brasiliensis* is likely related to the appearance of two new *sod* genes in the genome of the fungus. The two new *sod* genes have a phylogenetic relation to SodA.

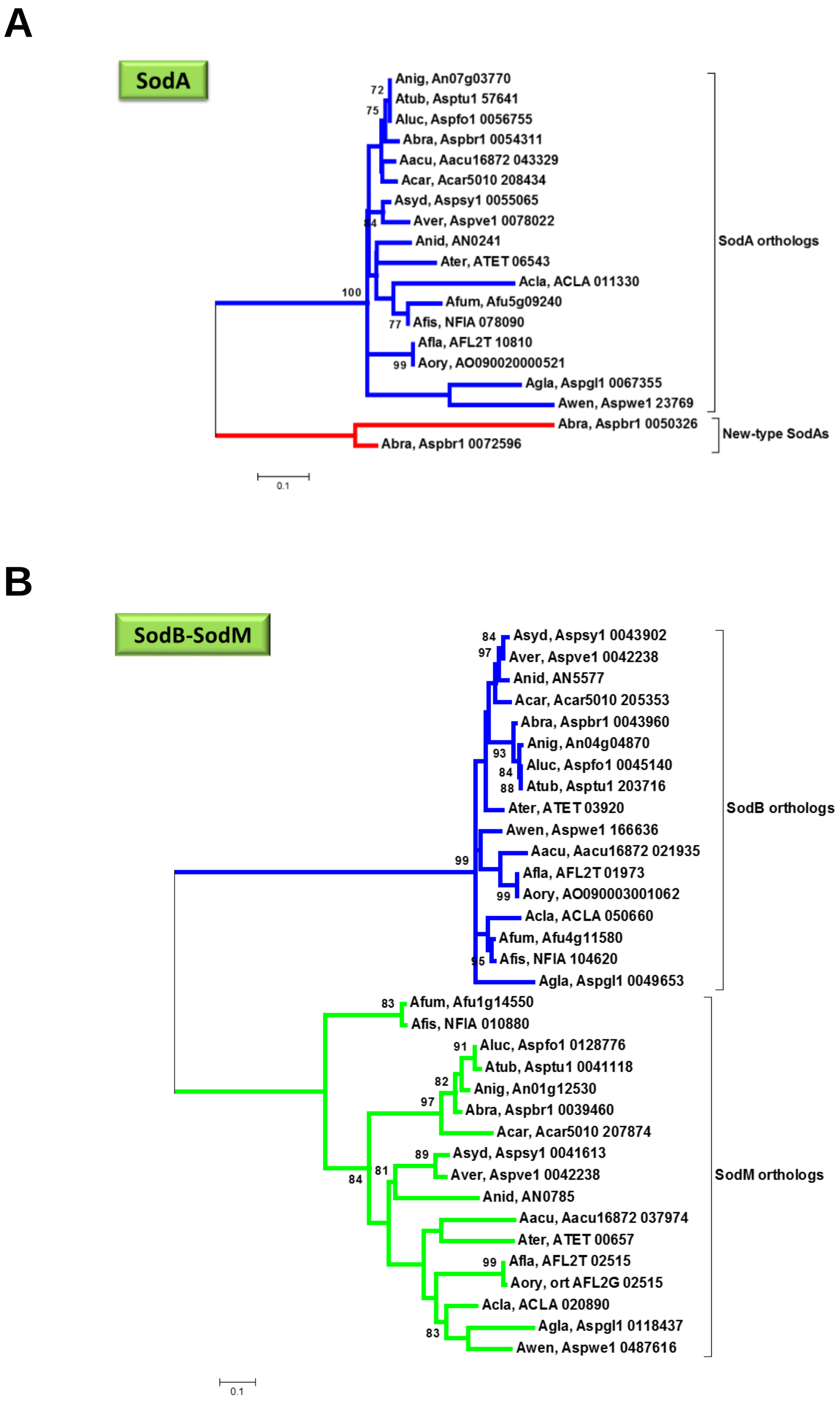

Figure S4. Dendrogram of the *Aspergillus* superoxide dismutases. SodA as well as SodB and SodM orthologs are presented in Parts A and B, respectively. Superoxide dismutase orthologs are indicated by four letters species name identifiers (Table S1) and locus IDs found in AspGD. (The relevant JGI locus IDs are listed in Additional File 16.)

All these observations indicate that the stress defense systems of the aspergilli are evolving rapidly which helps this important group of fungi to occupy effectively all the ecological niches available for them [2]

References

1. Miskei M, Karanyi Z, Pocsi I: **Annotation of stress-response proteins in the aspergilli.** *Fungal Genet Biol* 2009, **46 Suppl 1:**S105-120.
2. Nikolaou E, Agrafioti I, Stumpf M, Quinn J, Stansfield I, Brown AJ: **Phylogenetic diversity of stress signalling pathways in fungi.** *BMC Evol Biol* 2009, **9:**44.
3. Barratt RW, Johnson GB, Ogata WN: **Wild-type and mutant stocks of *Aspergillus nidulans*.** *Genetics* 1965, **52:**233-246.
4. Yin WB, Reinke AW, Szilagyi M, Emri T, Chiang YM, Keating AE, Pocsi I, Wang CC, Keller NP: **bZIP transcription factors affecting secondary metabolism, sexual development and stress responses in *Aspergillus nidulans*.** *Microbiology* 2013, **159:**77-88.
5. Karányi Z, Holb I, Hornok L, Pócsi I, Miskei M: **FSRD: fungal stress response database.** *Database (Oxford)* 2013, **2013:**bat037.
6. Hall TA: **BioEdit: a user-friendly biological sequence alignment editor and analysis program for Windows 95/98/NT.** *Nucl Acids Symp Ser* 1999, **41:**95-98.
7. Tamura K, Peterson D, Peterson N, Stecher G, Nei M, Kumar S: **MEGA5: Molecular evolutionary genetics analysis using maximum likelihood, evolutionary distance, and maximum parsimony methods.** *Mol Biol Evol* 2011, **28:**2731-2739.
8. Shiraishi E, Inouhe M, Joho M, Tohoyama H: **The cadmium-resistant gene, *CAD2*, which is a mutated putative copper-transporter gene (*PCA1*), controls the intracellular cadmium-level in the yeast *S. cerevisiae*.** *Curr Genet* 2000, **37:**79-86.
9. Kawasaki L, Aguirre J: **Multiple catalase genes are differentially regulated in Aspergillus nidulans.** *J Bacteriol* 2001, **183:**1434-1440.
10. Guelfi A, Azevedo RA, Lea PJ, Molina SM: **Growth inhibition of the filamentous fungus *Aspergillus nidulans* by cadmium: an antioxidant enzyme approach.** *J Gen Appl Microbiol* 2003, **49:**63-73.
11. Todorova D, Nedeva D, Abrashev R, Tsekova K: **Cd (II) stress response during the growth of *Aspergillus niger* B 77.** *J Appl Microbiol* 2008, **104:**178-184.
12. Goetz KE, Coyle CM, Cheng JZ, O'Connor SE, Panaccione DG: **Ergot cluster-encoded catalase is required for synthesis of chanoclavine-I in *Aspergillus fumigatus*.** *Curr Genet* 2011, **57:**201-211.
13. Navarro RE, Stringer MA, Hansberg W, Timberlake WE, Aguirre J: ***catA*, a new *Aspergillus nidulans* gene encoding a developmentally regulated catalase.** *Curr Genet* 1996, **29:**352-359.
14. Kawasaki L, Wysong D, Diamond R, Aguirre J: **Two divergent catalase genes are differentially regulated during *Aspergillus nidulans* development and oxidative stress.** *J Bacteriol* 1997, **179:**3284-3292.
15. Fillinger S, Ruijter G, Tamas MJ, Visser J, Thevelein JM, d'Enfert C: **Molecular and physiological characterization of the NAD- dependent glycerol 3-phosphate dehydrogenase in the filamentous fungus Aspergillus nidulans.** *Mol Microbiol* 2001, **39:**145-157.
16. Furukawa K, Yoshimi A, Furukawa T, Hoshi Y, Hagiwara D, Sato N, Fujioka T, Mizutani O, Mizuno T, Kobayashi T, Abe K: **Novel reporter gene expression systems for monitoring activation of the *Aspergillus nidulans* HOG pathway.** *Biosci Biotechnol Biochem* 2007, **71:**1724-1730.
17. Oberegger H, Zadra I, Schoeser M, Haas H: **Iron starvation leads to increased expression of Cu/Zn-superoxide dismutase in *Aspergillus*.** *FEBS Lett* 2000, **485:**113-116.
18. Hisada H, Sano M, Ishida H, Hata Y, Abe Y, Machida M: **Deletion analysis of the superoxide dismutase (*sodM*) promoter from *Aspergillus oryzae*.** *Appl Microbiol Biotechnol* 2006, **72:**1048-1053.
19. Oberegger H, Schoeser M, Zadra I, Abt B, Haas H: **SREA is involved in regulation of siderophore biosynthesis, utilization and uptake in Aspergillus nidulans.** *Mol Microbiol* 2001, **41:**1077-1089.
