# Supplementary material for: Comparative genomics reveals high biological diversity and specific adaptations in the industrially and medically important fungal genus Aspergillus
Source: Genome Biol. 2017 Feb 14;18:28. doi: 10.1186/s13059-017-1151-0 (PMC5307856; doi:10.1186/s13059-017-1151-0)
Supplement: Additional file 17: — Extended analysis of stress tolerance of the aspergilli (2). (PDF 1842 kb) [file 13059_2017_1151_MOESM17_ESM.pdf]

Additional File 17: Extended analysis of stress tolerance of the aspergilli (2)

Harald Kusch[1], Susanne Freyberg[1], Susanna A. Braus-Stromeyer[1], Özgür Bayram[1], István Pócsi[2], Arthur F.J. Ram[3], Ellen L. Lagendijk[3], Jean Paul Ouedraogo[3], Vera Meyer[4], Natalia Mielnichuk[5], David Cánovas[5], Stefan Rauscher[6], Julian Röhrig[6], Reinhard Fischer[6], Gerhard H. Braus[1]

[1]Abteilung für Molekulare Mikrobiologie und Genetik, Institute of Microbiology and Genetics, Georg-August-Universität Göttingen, Grisebachstr. 8, D-37077 Göttingen, Germany; [2]Department of Microbial Biotechnology and Cell Biology, Faculty of Science and Technology, University of Debrecen, Debrecen, Hungary and MTA-DE Vascular Biology, Thrombosis and Hemostasis Research Group, Hungarian Academy of Sciences, Debrecen, Hungary; [3]Leiden University, Institute of Biology Leiden, Molecular Microbiology and Biotechnology, Sylviusweg 72, 2333 BE Leiden, The Netherlands
[4]Berlin University of Technology, Institute of Biotechnology, Department Applied and Molecular Microbiology, Gustav-Meyer-Allee 25, D-13355 Berlin, Germany; [5]Departamento de Genética, Facultad de Biología, Universidad de Sevilla, Spain; [6]Karlsruhe Institute of Technology, Institute for Applied Biosciences, Department of Microbiology, Hertzstr. 16, D-76187 Karlsruhe

- **8 Supplementary Figures: Figure S17.1, Figure S17.2, Figure S17.3, Figure S17.4, Figure S17.5, Figure S17.6, Figure S17.7. Figure S17.8**
- **1 Supplementary Tables: Table S17.1**
- **Materials and Methods**
- **References**

Filamentous fungi are exposed to a complex set of abiotic or biotic stresses in natural environments. Effective stress perception and signal transduction mechanisms are necessary for adaptation and survival when rapid or slow adaptation responses are required. 18 species representing the ubiquitous fungal genus *Aspergillus* were examined covering all phylogenetic branches. Genomic sequences of all analyzed strains are available and include novel genomes of 8 species which have been sequenced in the course of this study. The potential spectrum of fungal reactions was compared during illumination or oxidative stress as abiotic cues or the growth response when a common Gram-negative soil bacterium is competing for nutrients as an example for a biotic stress. All strains were analyzed under the same physiological culture conditions in a complex complete medium supporting significant growth with a rate ranging from slow to fast.

Stress conditions were applied under the same physiological requirements using malt extract agar (MEA) as major carbon source which can be used for growth by all analyzed strains. Most species (11) could grow with a similar fast growth rate in this medium (Fig. S17.1), whereas only a small portion of the applied species (four) exhibited a slow growth phenotype. Four species showed an intermediate growth pattern. Two out of three strains representing the species *A. niger* were in the fast and one in the intermediate growth category. Fast as well as slow growth on malt extract is also reflected in the phylogenetic tree derived from the genome sequences (Fig. 8).

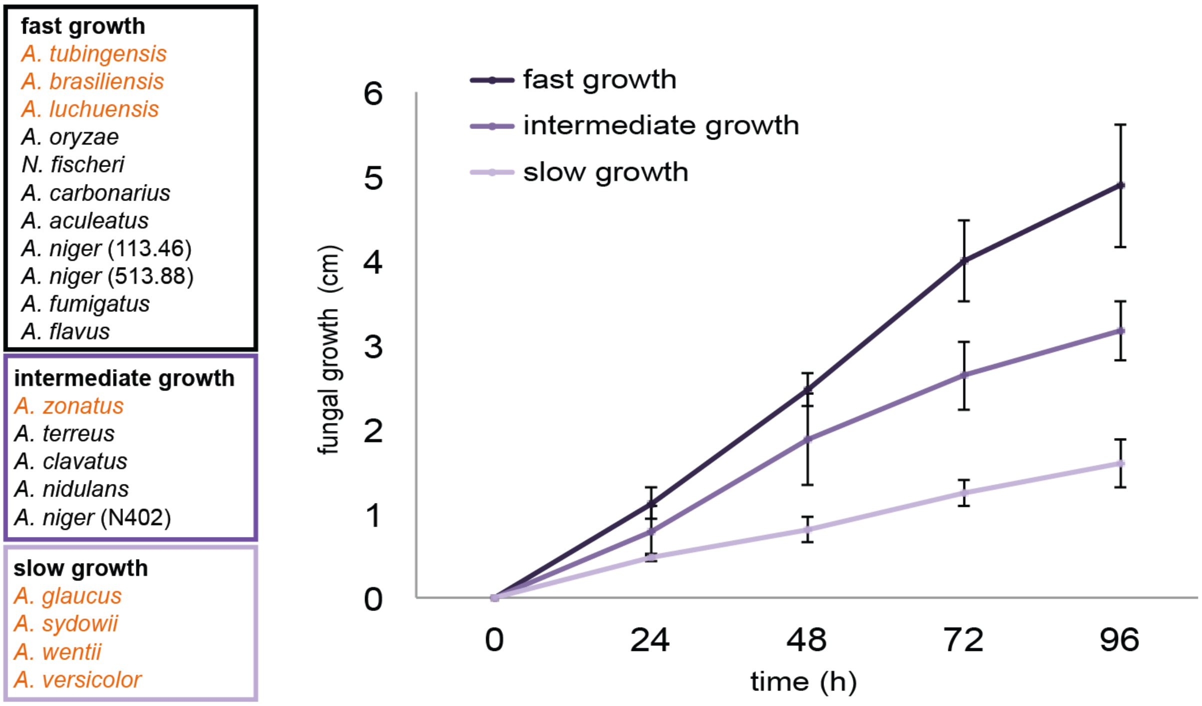

Figure S17.1: Comparison of colony growth of three growth categories of together 18 *Aspergillus* species (represented by 20 strains) on agar plates. *Aspergillus* strains were cultured on solid complete medium (MEA) at 30˚C. The colony size was measured in a time period of four days. Growth of each strain was measured in triplicates. All *Aspergillus* strains were able to grow on this medium with growth rates which correspond to three categories which were defined as: slow-, intermediate- and fast growth. Lines correspond to the average values of each category. Error bars represent the standard deviation for each group. Strains in orange correspond to the newly sequenced species.

Light induced conidiation is common for most *Aspergilli* except examples of industrial, pathogenic or toxic species

Light is known as an important triggering signal to influence *Aspergillus* development. In the model mold *Aspergillus nidulans* light controls the degree of asexual or sexual development by reducing sexual differentiation and reproduction and simultaneously promoting conidiation [1]. An amino acid sequence comparison revealed that genes for the light receptors for different wave length perception as well as for the velvet domain family are present in the genomes of all 18 species.

Light control depends on various photoreceptors and components of light depending signaling pathways like members of the velvet domain family [2]. *Aspergilli* perceive light signals in a wide wavelength range through the UV receptor CryA, the red light receptor FphA or the white collar blue light receptors LreA and LreB. Sequence identity ranges varied for the different genes when identities relative to the corresponding *A. nidulans* counterparts were compared. The CryA UV receptors (ranging from 67% to 78%) and the FphA red light receptors (from 57% to 78%) are most conserved among *Aspergilli* (Fig. S17.2A). In contrast both blue light receptors are less conserved with identities ranging from 38% to 61% for LreB and 49% to 64% for LreA, respectively.

The trimeric velvet complex consisting of the velvet domain protein heterodimer VelB-VeA and the putative methyltransferase LaeA is coordinating fungal development and secondary metabolism in *A. nidulans.* VeA bridges physically the two other proteins [3]. VelB identities relative to the corresponding *A. nidulans* gene vary in a narrow window of 67% to 70%; the window for LaeA amino acid identities is similar but broader (65% to 86%). The highest variability is shown for the bridging protein VeA ranging from 28% (in the *A. carbonarius* genome) to 73%. When the differences in the VeA amino acid sequence is used as single criterion to build a phylogenetic tree, this results in a different classification of species like *A. carbonarius* within the genus tree of *Aspergilli* in comparison to VelB or LaeA derived trees (Fig. S17.2B).

The presence of genes of numerous light receptors and regulators within the *Aspergillus* genus suggests similarities in the impact of light on development. We examined the effect of white light versus darkness on conidiation during malt extract driven cultivation. 13 of 18 *Aspergillus* species including all newly sequenced strains showed significant stimulation of conidiation when illumination and darkness conditions were compared. The only exceptions were three different strains of the industrially used black *A. niger*, toxin producers which contaminate food such as the black *A. carbonarius* (with lowest LreA and VeA similarity of all strains relative to *A. nidulans*) or *A. flavus*, and the human pathogens *A. fumigatus* and *A. terreus* (Fig. 8, Fig. S17.3). This suggests specific adaptations of *Aspergilli* to selective pressures provided in biotechnology, agriculture or by host-pathogen interactions.

A

	FphA Identities [%]	LreA Identities [%]	LreB Identities [%]	CryA Identities [%]	VeA Identities [%]	VeB Identities [%]	LaeA Identities [%]
A. glaucus	62	50	51	72	47	67	72
A. tubingensis	59	50	57	75	56	69	78
A. brasiliensis	60.2	50.9	57	75.5	56.7	68.1	73.7
A. sydowii	77.5	63.9	54.7	77.1	72.4	79.9	86.1
A. wentii	63	50	54	71	51	68	72
A. luchuensis	60	51.6	57	76	56.7	69.8	80.4
A. zonatus	61	54	57	67	55	64	74
A. versicolor	75.2	63.3	51.1	77.9	72.5	79.1	85.5
A. niger (N402)	60.2	51.6	57	75.6	56.4	63.6	73.9
A. oryzae	61.6	62.8	61	74.5	54	69.9	76.6
A. nidulans	100	100	100	100	100	100	100
N. fischeri	63	51.6	44	70	55.2	69.9	74.5
A. carbonarius	60	49	57	74	28	70	77
A. aculeatus	60	50	38	74	47	69	69
A. niger (113.46)	60.2	51.6	57	75.6	56.4	63.6	73.9
A. niger (513.88)	60.2	51.6	57	75.6	56.4	63.6	73.9
A. fumigatus	62.7	50.1	57	70.6	55.1	69.7	74
A. terreus	57.5	51	43	72.2	51.3	68.3	66
A. clavatus	62.7	51.2	47	72.9	68.9	70.3	75.1
A. flavus	61.6	52.1	61	73.6	51.8	69.7	65

< 50%   > 50%   > 70%   100%

B

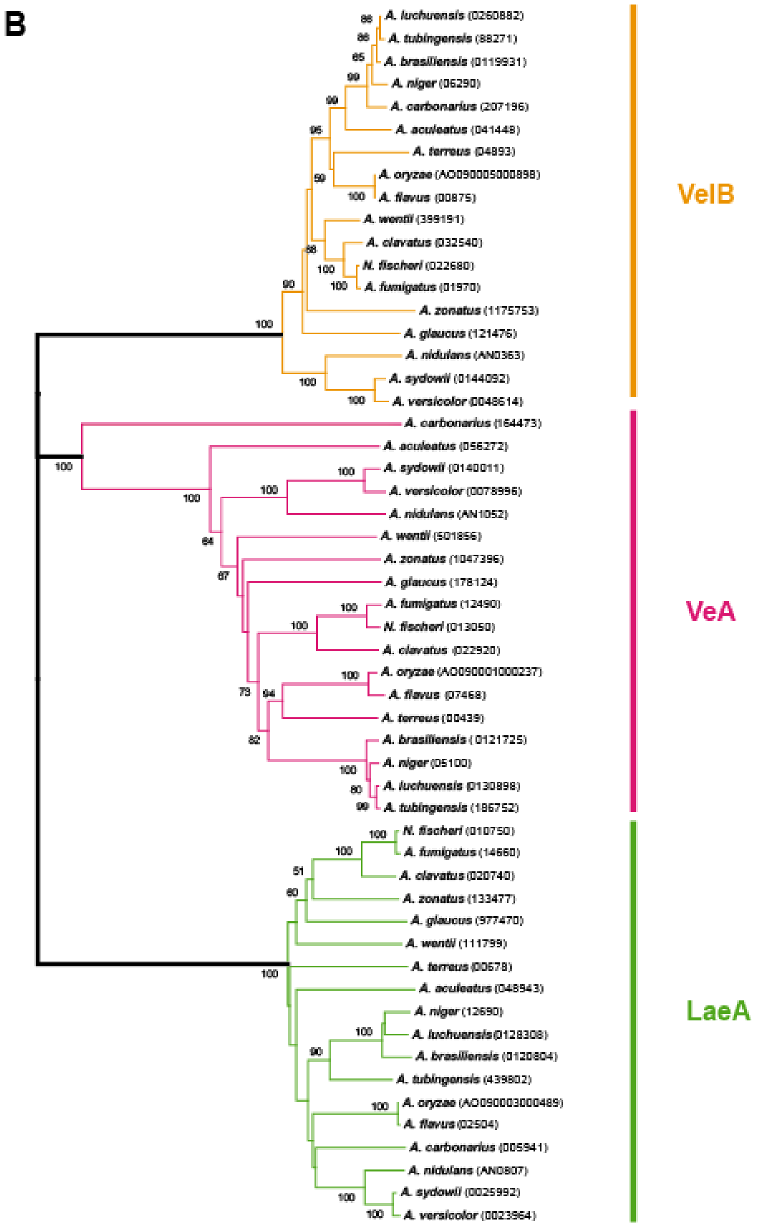

Figure S17.2: Comparison of deduced protein sequences of light control proteins of *Aspergillus* species. A. Blast based alignments of photoreceptors and regulatory genes involved in light regulation. *Aspergillus* genomes were analyzed with the Basic Local Alignment Search Tool (BLAST) using *Aspergillus nidulans* protein sequences as reference. The photosensor FphA for red-light sensing is highlighted in red, blue-light sensing proteins via LreA-

LreB or blue-UVA sensing via CryA are highlighted in blue. Velvet proteins veA, velvet-like proteins velB and LaeA are highlighted in pink, yellow or green. Values represent identical amino acids in comparison to the *A. nidulans* reference sequences (accession numbers AN9008 (FphA), AN3435 (LreA), AN3607 (LreB), AN0387 (CryA), AN1052 (VeA), AN0363 (VeB) and AN0807 (LaeA)). Novel genomes of this studiy include *A. tubingensis, A. wentii, A. versicolor, A. sydowii, A. glaucus, A. luchuensis, A. brasiliensis* and *A. zonatus.* **B.** Tree-based comparison of protein sequences of *Aspergillus* species (VelA, VelB and LaeA). The presented tree was generated by MEGA 5.2.2 (neighbor joining, 1,000 bootstrap replications). Branch lengths indicate relative relatedness and numbers above branches are bootstrap values. Only values above 50% are indicated. The content within bracket indicates the AspGD or JGI database for each strain.

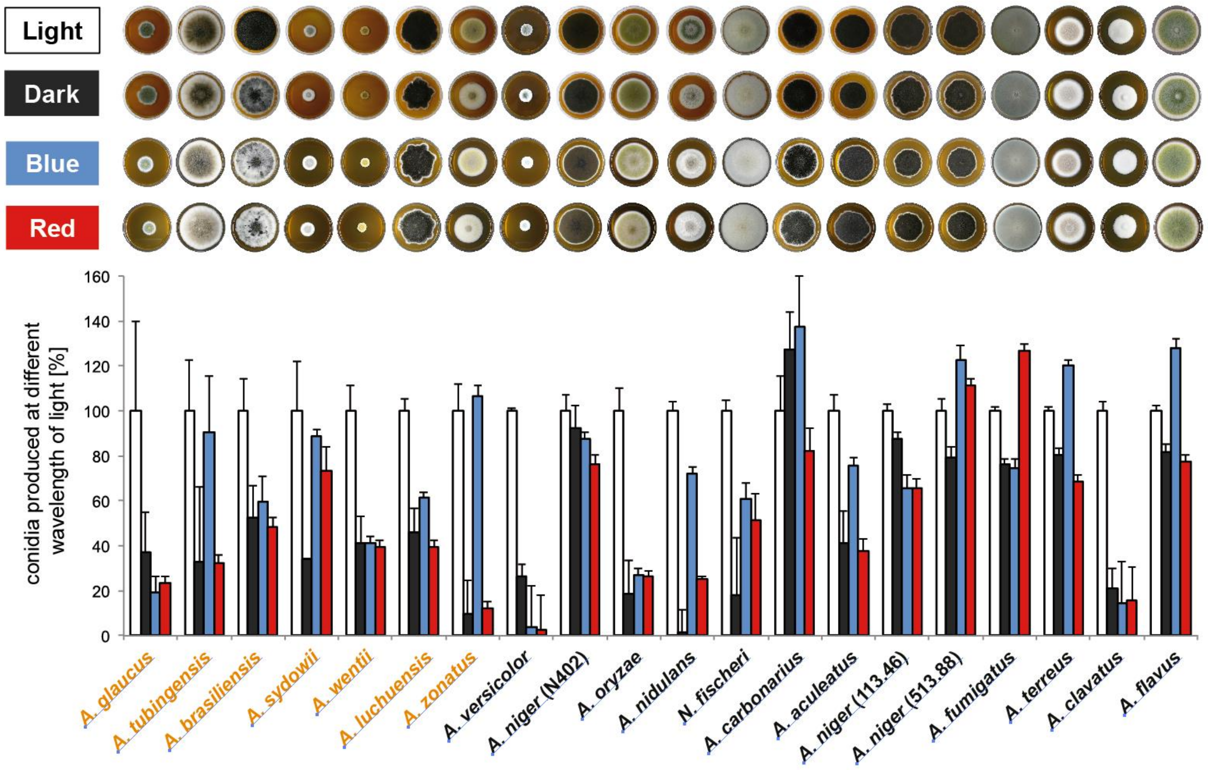

Figure S17.3 Light induction influences asexual development. Asexual development was induced on solid complete medium (MEA) by incubation in different light illuminations, at 30˚C for 6 days. For quantification of conidiospores formation, conidia are counted in an area of 50 mm^2. Error bars represent the standard deviation (n=3). As reference, conidiation in white light of the respective strain was set to 100%. Strains in orange correspond to the newly sequenced species.

Stimulation of conidiation of different species of the genus *Aspergillus* is specific for different wave lengths

The 13 *Aspergillus* species which promote conidiation during illumination were further dissected by replacing white light for selected illumination with different wave lengths (Fig. S17.3). This allowed a further distinction between the different species of *Aspergillus*. Only three species (*A. tubingensis*, *A. zonatus*, *A. aculeatus*) which are dispersed on the phylogenetic tree of *Aspergillus* are significantly induced in conidiation by blue but not by red light. Conidiation of three species which are *A. sydowii*, *A. fischeri* and *A. nidulans* is significantly promoted by blue as well as red light. In contrast, there was no species with an exclusive induction of asexual development by red light. Conidiation of six species

which are distributed on the phylogenetic tree (*A. glaucus, A. brasiliensis, A. wentii, A. luchuensis, A. oryzae, A. clavatus*) was promoted by white light but neither by red nor by blue light suggesting that receptors like CryA or yet unidentified receptors might be here more important, whereas red or blue receptors might not play a significant role in these species for asexual development. The CryA amino acid sequence of *A. versicolor* has the highest similarity to *A. nidulans* where a CryA dependent UV response on development had been demonstrated [4]. *A. versicolor* represents a special case of white light induced stimulation of conidiation. Only this species is in addition significantly inhibited in asexual development by blue or red light suggesting that light receptors might induce different responses in the *Aspergilli*. The data demonstrate a significant range of light dependent responses on conidia formation within the genus of *Aspergillus* which might reflect the different ecological niches which are colonized in nature.

Oxidative stress tolerance ranges between resistant and highly sensitive among *Aspergillus* species

Fungal cells are exposed to stress by reactive oxygen species (ROS) in nature by abiotic, biotic or internal sources. ROS can cause oxidative damage to DNA, lipids and proteins in higher concentrations [5,6]. Fungi possess a variety of defense mechanisms such as antioxidant enzymatic systems to cope with oxidative stress [7,8]. These enzymes are of essential importance in oxidative stress defense in filamentous fungi. Among those superoxide dismutases (SodA, SodB in *A. nidulans;* [9]) convert superoxide anions into oxygen and hydrogen peroxide. Hydrogen peroxide can be detoxified by catalases (CatA, CatB, CatC in *A. nidulans*; [10–12]). The antioxidant thioredoxin system is involved in balancing the redox state of the cell. It includes thioredoxins and thioredoxin reductases (TrxA, TrxR in *A. nidulans*; [13]). In the 20 *Aspergillus* genomes the highest peptide sequence similarities in comparison to *A. nidulans* were detected for SodA, SodB, CatC and TrxR (76-94 % identity) followed by CatA and CatB (73-84 % identity) and TrxA (51-78 % identity) (Fig. S17.4A). There are some remarkable exceptions in conservation. CatB (45%) and TrxR (41%) showed only reduced sequence identities in comparison to the others in *A. zonatus.* Multiple sequence alignments of protein sequences for CatA, CatB and CatC resulted in similar groupings of *Aspergilli* (Fig. S17.4B) in comparison to the phylogenetic tree (Fig. 8).

A

	SodA Identities [%]	SodB Identities [%]	CatA Identities [%]	CatB Identities [%]	CatC Identities [%]	TrxA Identities [%]	TrxR Identities [%]
A. glaucus	81	76	75	73	86	64	86
A. tubingensis	88	83	75	75	85	73	86
A. brasiliensis	88	85	75	76	88	66	87
A. sydowii	92	94	82	81	93	64	90
A. wentii	79	81	73	76	87	53	86
A. luchuensis	88	83	75	75	86	72	86
A. zonatus	84	86	74	45	85	61	41
A. versicolor	88	95	83	84	92	65	90
A. niger (N402)	88	84	75	75	86	71	87
A. oryzae	88	82	79	77	85	51	87
A. nidulans	100	100	100	100	100	100	100
N. fischeri	90	84	79	77		62	91
A. carbonarius	88	85	75	76	86	74	87
A. aculeatus	88	82	76	77	85	69	88
A. niger (113.46)	88	84	75	75	86	71	87
A. niger (513.88)	88	84	75	75	86	71	87
A. fumigatus	89	86	79	77	45	62	90
A. terreus	92	86	77	76	87	78	89
A. clavatus	89	80	78	75			85
A. flavus	88	82	79	77	85	64	87

< 50% > 50% > 70% 100%

B

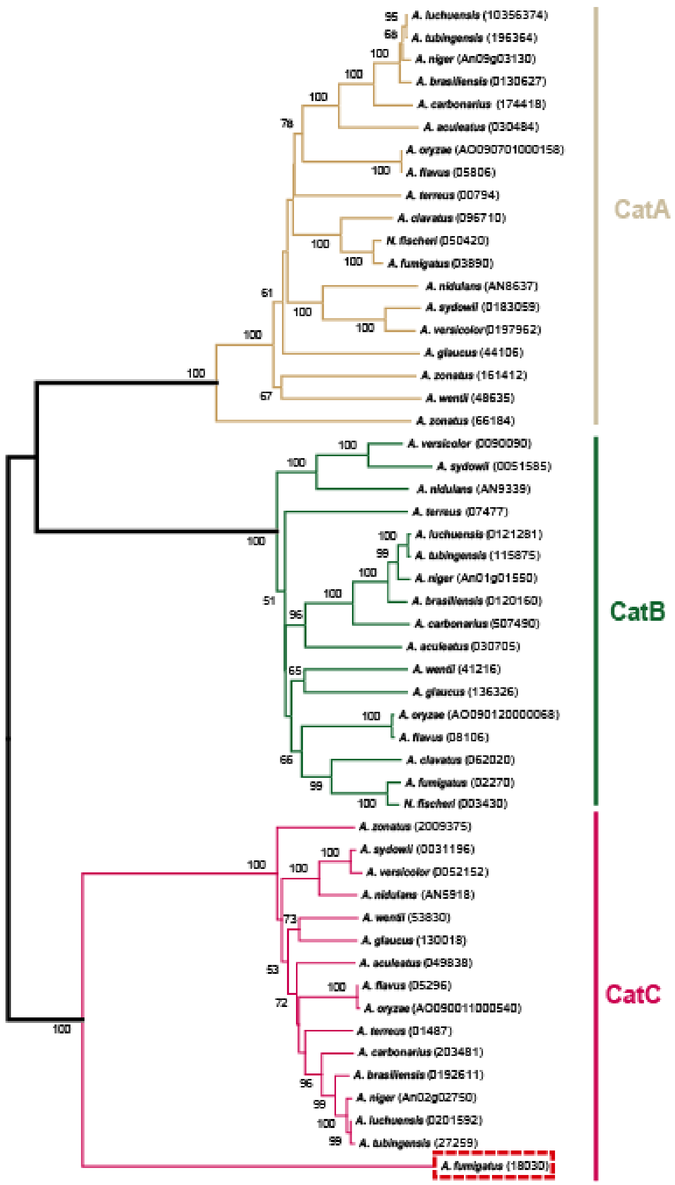

Figure S17.4: Comparison of deduced protein sequences of proteins involved in oxidative stress protection of *Aspergillus* species. A. Blast based alignments of enzymes involved in detoxification of ROS. All 20 *Aspergillus*

genomes were analyzed with Blast using *Aspergillus nidulans* protein sequences as reference. SOD = Superoxide dismutase. Cat = Catalase, Trx = Thioredoxin reductase. Values represent identical amino acids in comparison to the *A. nidulans* reference sequences (accession numbers AN0241 (SodA), AN5577 (SodB), AN8637 (catA), AN9339 (catB), AN5918 (catC), AN0170 (trxA) and AN3581 (trxR)). Newly sequenced species are highlighted in orange. **B.** Tree-based comparison of protein sequences (CatA, CatB and CatC) of *Aspergillus* species. The presented tree was generated by MEGA 5.2.2 (neighbor joining, 1,000 bootstrap replications). Branch lengths indicate relative relatedness and numbers above branches are bootstrap values. Only values above 50% are indicated. Terms in brackets represent the AspGD or JGI database accession numbers for protein sequences. The red frame highlights a new-type catalase C protein of *A. fumigatus.*

No significant blast hits were detected for CatC in *N. fischeri* and CatC and TrxA in *A. clavatus* suggesting that this gene has been lost in these species. CatC of *A. fumigatus* (45%) is an exception and distinct from the other CatC representatives. It builds a single tree branch which is uncorrelated to the phylogenetic tree position of *A. fumigatus* suggesting a horizontal gene transfer. This catalase was described as the EasC component of an ergot alkaloid synthesis cluster and is required for synthesis of chanoclavine-I [14–16]. We examined the function of EasC in oxidative stress by comparing wildtype *A. fumigatus* to the described *easC* disruption mutant strain [15]. Growth of the deletion mutant strain was reduced by 50% in the presence of 0.8 mM hydrogen peroxide which suggests that EasC is also involved in oxidative stress response (Fig. S17.5).

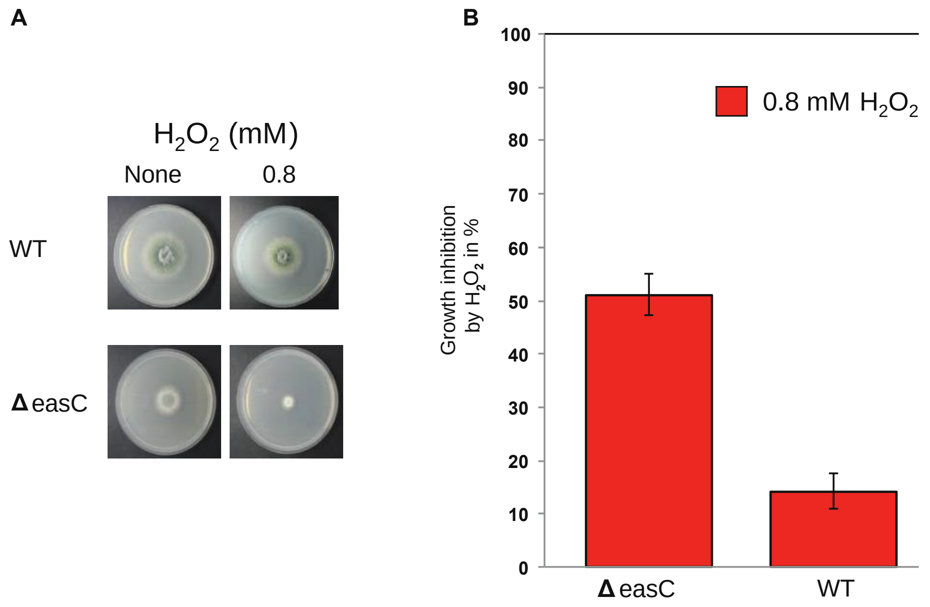

Figure S17.5: The *A. fumigatus easC* (18030) disruption mutant [15] **displays increased sensitivity towards hydrogen peroxide treatment. A.** Growth phenotype of the hydrogen peroxide stressed *easC* disruption mutant. Conidia of *A. fumigatus* wild type and *easC* disruption mutant were inoculated on *Aspergillus* minimal medium agar plates without or with addition of 0.8 mM hydrogen peroxide. **B.** Quantified relative growth inhibition in comparison to non-stressed control plates. Error bars represent the standard deviation (n=3).

High sequence similarities between antioxidant proteins suggested similar resistance patterns towards ROS induced stress. Therefore we performed growth (solid agar medium) and proliferation (liquid medium with AlamarBlue) tests in response to treatment with 0.8 and 10 mM hydrogen peroxide with

all 20 *Aspergillus* strains (Fig. S17.6). We observed heterogeneous sensitivity profiles dependent on fungal strain, growth condition and hydrogen peroxide concentration. All strains were inhibited in growth (> 20 % inhibition) with 10 mM hydrogen peroxide on solid medium but at this concentration two strains (*A. carbonarius* and *A. niger* 113.46) were not inhibited in liquid medium. Among others these two strains also showed less sensitivity on agar plates in comparison to the mean sensitivity of all strains. In contrast, at the lower concentration (0.8 mM) of hydrogen peroxide only three strains (*A. glaucus, A. sydowii, A. zonatus*) were already inhibited by 30, 50 and 100 %, respectively. In liquid medium sensitivity was generally higher. Here, nine strains were already inhibited (30-90% inhibition) with *A. zonatus, N. fischeri and A. clavatus* being most sensitive (> 80% inhibition). Only *A. zonatus* and *A. sydowii* were inhibited in liquid and on solid medium already at 0.8 mM hydrogen peroxide.

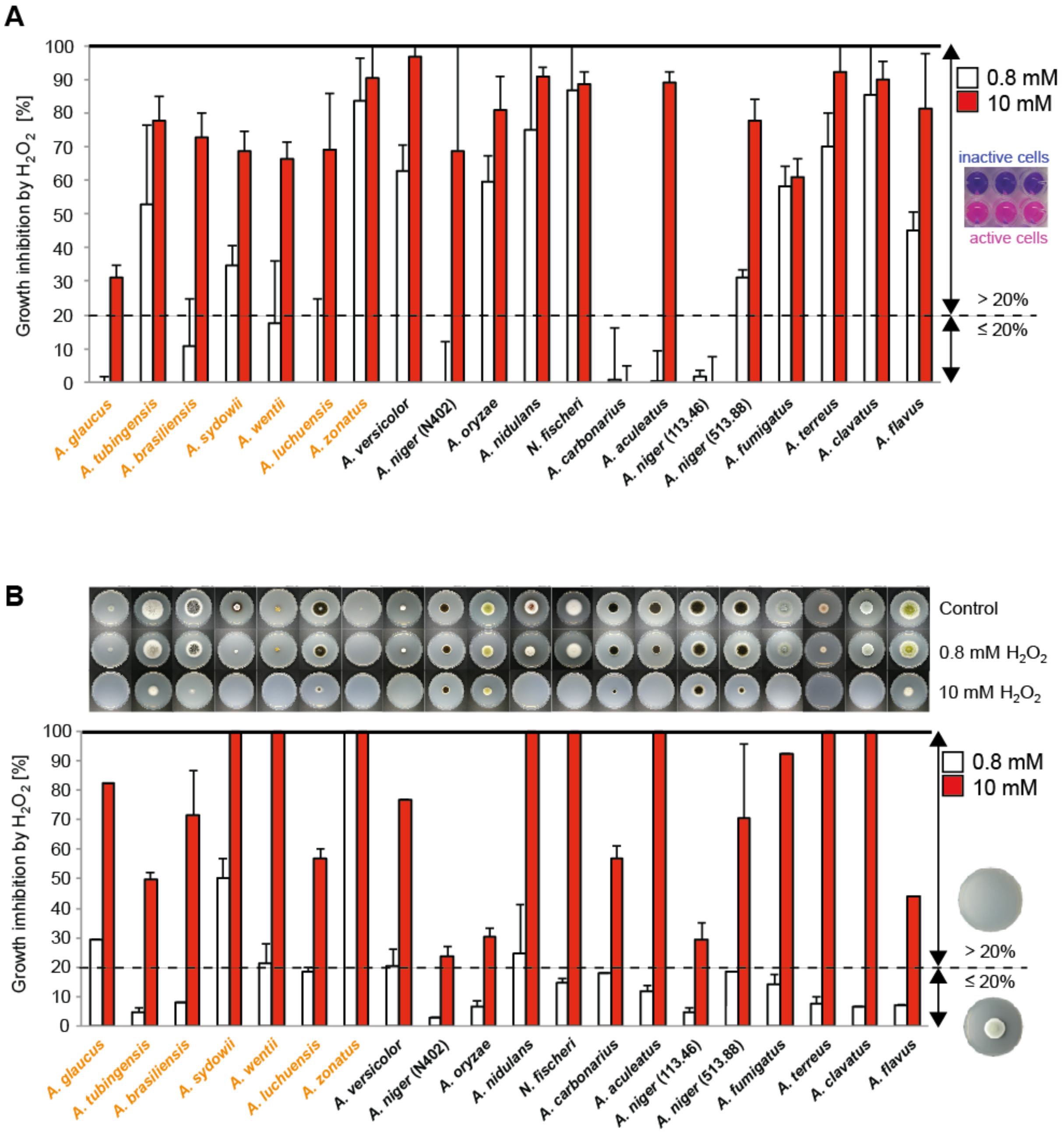

Figure S17.6 A: Hydrogen peroxide treatment induces inhibition in proliferation. The sensitivity of *Aspergillus* strains against two different hydrogen peroxide concentrations (0.8 mM, 10mM) was

determined in an AlamarBlue proliferation assay in liquid *Aspergillus* minimal medium at 30°C. Samples without hydrogen peroxide treatment were used as reference. Values in percent [%] represent the relative rate of growth inhibition compared to the reference. Error bars indicate the standard deviation (n=6). **B: Hydrogen peroxide treatment induces inhibition of fungal growth.** Conidia of the fungal species were inoculated on solid *Aspergillus* minimal medium (AMM) agar plates containing two different concentrations of hydrogen peroxide (0.8 mM and 10 mM). Colony size was measured after 4 days of incubation at 30 °C in the dark. Plates without hydrogen peroxide treatment were used as a reference. Growth inhibition is expressed in percent [%] of colony diameter of treated cells in relation to the un-stressed reference plates. Error bars represent the standard deviation (n=3). The newly sequenced strains are indicated in orange.

In summary, especially the black *Aspergilli A. carbonarius* and *A. niger* 113.46 are resistant to high hydrogen peroxide suggesting a protective role of melanin which is more pronounced in liquid medium. Particularly the strains under human influence (*A. niger* 113.46*, A. niger* N402*, A. oryzae and A. flavus*) are resistant on solid medium. *A. zonatus* and less pronounced also *A. sydowii* are most sensitive against hydrogen peroxide. The *catC* gene has been lost in some *Aspergillus* genomes including *N. fischeri, A. clavatus* and presumably also *A. fumigatus* where the *easC* gene for ergot synthesis has taken over protective functions against oxidative stress.

Co-cultivation of the soil bacterium *Pseudomonas fluorescens* and *Aspergillus spp.* reveal a gradient of resistant to sensitive fungal strains

Aspergilli are competing for nutrients with other microorganisms in their ecological niches. Among those are Gram-negative bacteria of the genus *Pseudomonas* which are ubiquitous in nature. A co-culture assay was performed to test if *Pseudomonas fluorescens* affects fungal growth of the 20 *Aspergillus* strains. We observed growth inhibition (inhibition zone > 2 mm) in eight *Aspergillus* strains, namely *A. oryzae, A. luchuensis, A. tubingensis, A. brasiliensis, A. versicolor, A. nidulans* and most sensitive (inhibition zone > 5 mm) *A. sydowii* and *A. wentii* (Fig. S17.7A).

Resistance patterns of *Aspergillus spp.* towards the *P. fluorescens* antimycotic 2,4-DAPG vary in a considerable range between resistance and high sensitivity

Pseudomonas species produce a variety of secondary metabolites with antifungal activity like 2,4-diacetylphloroglucinol (2,4-DAPG). Resistance mechanisms against 2,4-DAPG have been described for *Fusarium oxysporum* [17,18] and might also exist in *A. glaucus, A. zonatus, A. niger* or *A. fumigatus* which were not influenced in growth by the bacterium. The influence of the bacterial secondary metabolites on *Aspergilli* was analyzed by growth (solid agar medium) and proliferation (liquid medium with AlamarBlue) using two concentrations (80 µM and 0.3 mM) of purified 2,4-DAPG (Fig. S17.7B-C). All strains were sensitive to high concentrations of 2,4-DAPG (0.3 mM) on solid medium. The sensitivity profile was heterogeneous when all strains are compared. In liquid medium two strains (*A. wentii* and *A. flavus*) were not inhibited (inhibition ≤ 20 %) (Fig. S17.7B) and were also among the strains which owed less sensitivity on agar plates in comparison to the mean sensitivity of all strains. At the low concentration of 80 µM 2,4-DAPG nine strains were inhibited (inhibition > 20 %) in liquid and on solid medium. Six of these (*A. glaucus*, *A. tubingensis*, *A. versicolor*, *A. carbonarius*, *A. niger* 513.88 and *A. fumigatus*) were inhibited in both conditions. In liquid or on solid medium seven (*A. sydowii*, *A.*

nidulans, *A. aculeatus*, *A. niger* 113.46, *A. terreus*, *A. clavatus* and *A. flavus*) and four strains (*A. brasiliensis*, *A. sydowii*, *A. zonatus*, *A. niger* N402), respectively, were not inhibited (inhibition < 10 %). Only *A. sydowii* was resistant to 80 µM 2,4-DAPG at both conditions.

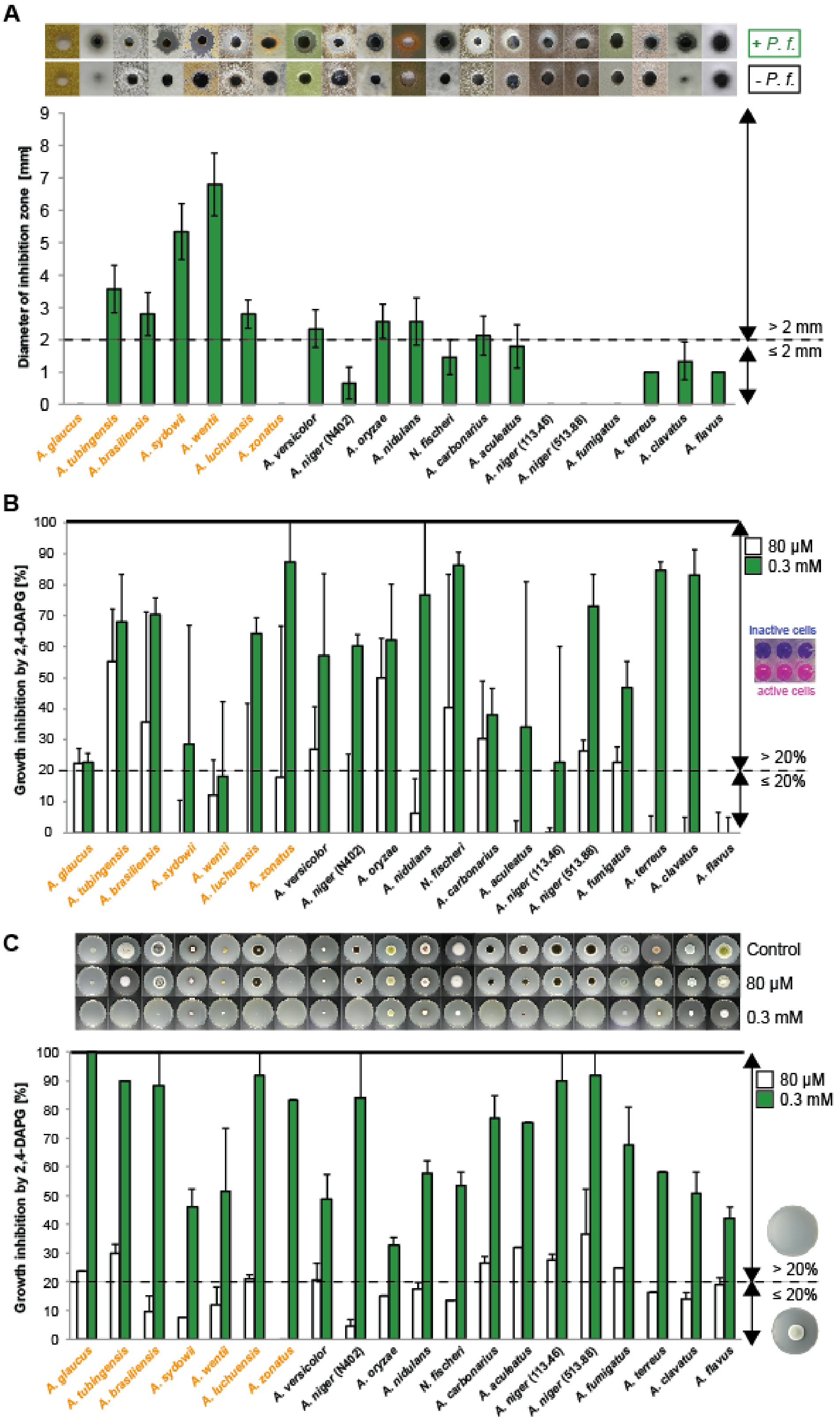

Figure S17.7 A: *Pseudomonas fluorescens* impact on growth of *Aspergillus* spp.. Conidia of the fungal species were inoculated by spreading on solid *Aspergillus* minimal medium (AMM). In the middle of the agar plate a well was filled with an overnight culture of Pseudomonas fluorescens suspension and incubated at 30˚C. Inhibition zones were measured after 4 days. Error bars represent the standard deviation in two independent experiments (n=3). The newly sequenced strains are indicated in orange. **B: Sensitivity of 20 *Aspergillus* strains towards 2,4 DAPG.** The sensitivity of *Aspergillus* strains against two 2,4-DAPG concentrations (80µM and 10mM 2,4-DAPG) was determined in an AlamarBlue proliferation assay. Samples without 2,4-DAPG treatment were used as reference. Error bars represent the standard deviation in two independent experiments (n=6). The newly sequenced species are indicated in orange. **C: Sensitivity of 20 *Aspergillus* strains to 2,4 DAPG.** Conidia of the fungal species were inoculated on *Aspergillus* minimal medium (AMM) agar plates containing two concentrations of 2,4-DAPG (80 µM and 0.3 mM 2,4-DAPG). Colony size was measured after 4 days of incubation at 30 °C in the dark. Strains without 2,4-DAPG treatment were used as reference. Error bars represent the standard deviation (n=3). The newly sequenced species are indicated in orange.

Summarized, we observed a broad spectrum of sensitive and resistant *Aspergillus* strains towards bacterial or 2,4-DAPG-treatment (80 µM). This might reflect variations in the adaptation to different ecological niches. Only *A. zonatus* and *A. niger* N402 were resistant towards bacterial and 2,4-DAPG treatment at 80 µM which supports earlier findings [19] that 2,4-DAPG is produced in lower amounts in the *Aspergillus-Pseudomonas* co-cultivation.

Exoproteins vary under the same cultivation conditions in four representative *Aspergillus spp.*

Different ecological niches of Aspergilli might be reflected by different exoproteomes. Four representatives (*A. tubingensis, A. wentii, A. sydowii, A. zonatus*) of the 20 strains were chosen for secretome analysis according to the following criteria: (i) covering the phylogenetic tree (Fig. 8), (ii) differences in the response of co-cultivation *Aspergillus-Pseudomonas* (Fig. S17.7A), and (iii) sensitivity or insensitivity against hydrogen peroxide (Fig. S17.6). *A. zonatus* is unsensitive (resistant) against the bacterium, but sensitive against hydrogen peroxide. *A. tubingensis* shows an intermediate bacterial response. *A. wentii* and *A. sydowii* are highly sensitive against the bacterium, but only *A. sydowii* is also sensitive against hydrogen peroxide.

Aspergilli were grown for 3 days either non-stressed (without hydrogen peroxide addition) or stressed with sub-lethal 0.2 mM hydrogen peroxide in *Aspergillus* minimal medium. Exoproteomes were enriched from the filtered culture supernatants and analyzed by shotgun proteomics (LC-MS). The total number of all identified proteins differs between the four different *Aspergillus* strains (*A. sydowii* 602, *A. tubingensis* 528, *A. wentii* 230 and *A. zonatus* 750) (Fig. 8B). In contrast, the total number of the subfraction of putatively secreted proteins is differently distributed: *A. zonatus* secretes fewer exoproteins (80) than either *A. sydowii*, *A. tubingensis* and *A. wentii* (173, 127 and 133, respectively) (Additional File 18).

Functional classification by detected PFAM-domains [20] enabled an inter-species comparison of the four exoproteomes. Although many PFAM domains were detectable in all four strains this analysis revealed a high heterogeneity of the exoproteomes of the different species (Fig. 8C). Most identified PFAM-domains were related to carbohydrate-active enzymes (CAZYs, 47), followed by peptidases (12), ROS defense-associated (11) and lipases (8). PFAM-domains that were specifically identified for each strain (Additional File 19) also contained members of these categories like glycoside hydrolases (e.g.

Aspsy1-342693 of *A. sydowii* or Aspzo1-137377 of *A. zonatus*) or peptidases (e. g. Aspsy1-80480 or Aspsy1-42088 of *A. sydowii*). Additionally, putative virulence factors of other functional classes were identified among the strain specific exoproteins (e.g. the LysM-domain containing Aspsy1-131653 of *A. sydowii*). Several proteins of the four exoproteomes were assigned as putative ROS detoxifying enzymes (thioredoxin-like proteins, catalase, peroxidase). Analysis of PFAM- and Superfamily-domains revealed that these proteins are as well present in unstressed cells as in stressed cells with hydrogen peroxide (Fig. 8B).

In summary, a comparison of four selected exoproteomes of *Aspergillus* ssp. reflects a spectrum of different secreted proteins which might correspond to different niche adaptations of the respective strains. All exoproteomes contain enzymes for nutrient acquisition like CAZYs and peptidases but each strain has a specific composition of this subfraction of proteins under the same growth conditions. The presence of ROS-defense related exoproteins reveals the importance of an efficient antioxidative machinery on the surface and outside of the fungal cell. *A. zonatus* which is unsensitive against co-cultivation with Pseudomana but also sensitive against hydrogen peroxide secretes fewer exoproteins than the other analysed strains.

Variations in cell wall gene composition are not indicative for susceptibility of *Aspergillus spp*. against cell wall drugs

The first and ultimate barriers which have to counteract adverse conditions and to ensure cell survival of *Aspergilli* are defined by the cell wall and cell membrane. Both are intimately bound and represent a dynamically forming exoskeleton harbouring most of the fungal antigens. Cell wall integrity is thus of vital importance for the survival of fungi. Besides, it coordinates cell wall biogenesis and cell polarisation thereby being key for the orchestration of growth and morphogenesis in fungi.

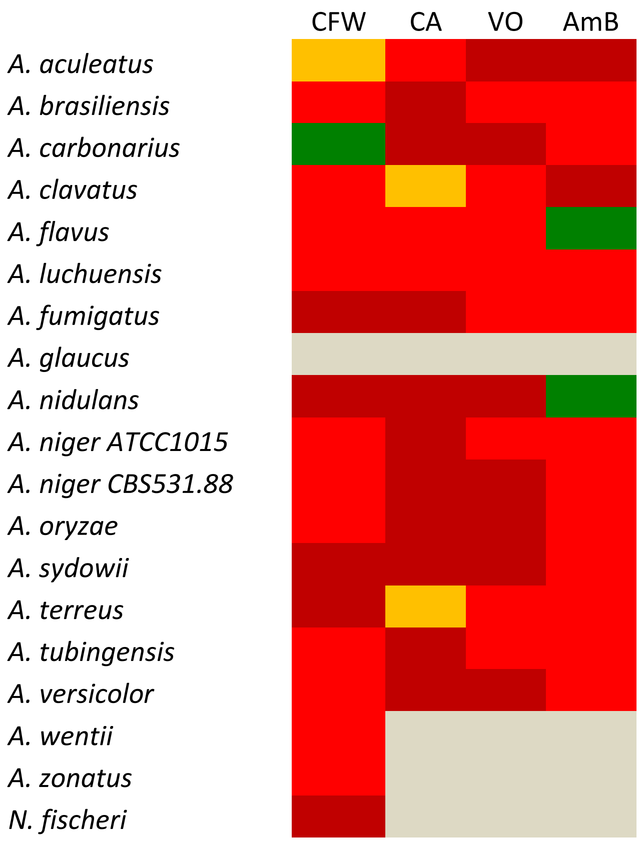

Figure S17.8: Susceptibilities of *Aspergilli* against calcofluor white (CFW), caspofungin (CA), voriconazole (VO) and amphotericin B (AmB). Color code: dark red, highly sensitive; red, sensitive; orange, moderate-sensitive; green: resistant; grey, not determined.

To inventory all cell wall genes in *Aspergilli*, the genome sequence of *A. niger* CBS513.88 was taken as reference as its cell wall genes were systematically annotated recently [21]. The cell wall of *A. niger* consists of chitin, β-1,3-glucan, β-1,6-glucan, α-1,3-glucan, galactomannan and galactosaminogalactan. Genes required for the synthesis and remodelling of theses polymers were found in all sequenced *Aspergillus* genomes (Table S17.1). Notably, a high degree of redundancies were observed. The number of chitin synthases ranged from 9 to 15, which is significantly higher when compared to *S. cerevisiae*, which only harbours 3 chitin synthases. The number of chitin remodelling and chitin-glucan crosslinking enzymes differed also considerably among the sequenced *Aspergillus* genomes (21-31 and 10-18, respectively), and the same was found for the genes involved in β-glucan synthesis and processing (15-31). No such extreme differences were observed with respect to galactomannan and galactosaminogalactan synthesis. *A. zonatus, A. clavatus* and *A. glaucus* comprise the lowest (69, 70, 70) and *A. kawachii, A. flavus* and *N. fischeri* the highest (96, 96, 99) amount of cell wall synthesis and remodelling genes in their genomes.

Can these differences be linked to the susceptibilities of *Aspergilli* towards cell surface acting antifungal compounds? To answer this question, all strains were stressed with the following antifungals: calcofluor white (inhibitor of chitin fibril formation), caspofungin (inhibitor of β-1,6-glucan synthesis), voriconazole (inhibitor of ergosterol synthesis) and amphotericin B (forms transmembrane channels). The latter two were chosen as many cell wall biosynthetic enzymes are thought to be embedded in

the cell membrane. Surprisingly, the strain with the highest number of chitin synthases is calcofluor white sensitive (*A. flavus*), whereas the strain with the lowest amount of chitin synthase genes turned out to be calcofluor white resistant (*A. carbonarius*, Fig. 8, Fig. S17.8 and Table S17.1). Also unexpectedly, the strains with overall very high number of cell wall genes, e.g. *A. niger* CBS513.88 and *A. niger* ATCC1015 displayed high sensitivities against all four antifungals. Thus, the comparative genome analysis of cell wall genes of different *Aspergilli* does not necessarily reflect a correlation between the number of cell wall genes and overall susceptibility against cell wall acting drugs. It is more likely that the different strains have evolved different strategies to counteract the presence of these antifungals. As previously suggested by [22], the outcome of an antifungal attack is not only dependent on the innate susceptibility of the microorganism, but also by the damage potential of the antifungal which is defined by its concentration and target-(non)specific molecular interactions as well as by the microbial survival response, which can be too weak, appropriate, or even too strong and thus detrimental to the host.

Table S17.1: Fungal strains used in this study.

Strain nr	Species	(Source)/Country of origin/Reference
CBS 172.66	*A. aculeatus*	(CBS)
CBS 101740	*A. brasiliensis*	(CBS) Brazil
DTO 115-B6	*A. carbonarius*	(CBS)
CBS 544.65	*N. fischeri*	(CBS)
CBS 106.47	*A. luchuensis*	(CBS) Switzerland
FGSC A4	*A. nidulans*	(CBS)
N402	*A. niger*	(CBS
Rib40	*A. oryzae*	(CBS)
CBS 593.65	*A. sydowii*	(CBS)
NIH 2624	*A. terreus*	(CBS)
CBS 134.48	*A. tubingensis*	(CBS)
DTO 134-E9	*A. wentii*	(CBS)
CBS 506.65	*A. zonatus*	(CBS) Costa Rica
CBS 516.65	*A. glaucus*	(CBS) USA
NRRL 1	*A. clavatus*	(CBS)
CBS 113.46	*A. niger*	(CBS) USA
CBS 513.88	*A. niger*	(CBS)
CBS 795.97	*A. versicolor*	(CBS) Netherlands
NRRL 3357	*A. flavus*	(CBS) USA
Af293	*A. fumigatus*	(CBS)
Af *easC* disruption	*A. fumigatus*	[15]

Materials and methods

Strains and media

Aspergillus strains used in this work (Tab. S16.3) were obtained from Prof. Dr. Ronald de Vries (CBS, Utrecht, Netherlands). For cultivation 5 µl of conidia suspensions (1×10^6 conidia per ml) were inoculated at 30 °C on Malt-Extract-Agar (MEA, [23]) or *Aspergillus* minimal medium (AMM, [24]) for phenotype testing. For *Aspergillus glaucus* 1 M NaCl has been added to the media. Wild type *Pseudomonas fluorescens* CHA0 [25] was grown at 30 °C in LURIA-BERTANI medium (LB, [26]).

Determination of fungal growth rates

To determine fungal growth rates, *Aspergillus* strains were point inoculated onto the centers of MEA plates and incubated in the light. Radial growth was measured every day for four days.

Quantification of conidiospore formation

For induction of asexual development, *Aspergillus* spores were inoculated on MEA plates in the light or dark for 6 days. For blue (470 nm wavelength) and red (630 nm wavelength) light treatments, plates were incubated in a portable self-made illumination chamber. Conidia were harvested by scraping gently with a sterile cotton bud and transferred into 15 ml centrifuge tubes containing 5 ml of sterile physiological solution. Spore suspensions were enumerated with a hemocytometer and kept at 4 °C until used.

Cell proliferation AlamarBlue® Assay

Cell proliferation in liquid medium was carried out by a colorimetric Alamar Blue® (AB, AbD Serotec, Oxford, United Kingdom) based bioassay in flat-bottom 96-well plates (Sarstedt, Nümbrecht, Germany) with a final volume of 250 µl per well. For each *Aspergillus* strain and each well, 5 µl a 1×10^6 ml^{-1} conidia suspension were seeded into AMM medium with a final concentration of 10 % [v/v] of AB and incubated at 30°C in the dark for 24 h. Hydrogen peroxide or 2,4-diacetylphloroglucinol (DAPG, Toronto Research Chemicals Inc., Toronto, Canada, diluted in AMM) were added to a final concentration of 0, 0.8 or 10 mM or 0, 80 or 300 µM, respectively, followed by incubation for 2 days in the dark. Growth controls consisted of AMM medium, AB and conidia suspension. A background control consisted of AMM medium and AB. The background control corrected ratio of the absorbance was measured at 540 and 630 nm with a plate reader (Tecan, infinite M 200, Männedorf, Switzerland) after 0 h, 24 h, 48 h and 72 h.

Solid plate assay

Conidia suspensions were point inoculated onto the center of solid AMM agar plates containing varying end concentration of hydrogen peroxide or 2,4-DAPG at 0 (control), 0,8 mM and 10 mM or 0, 80 or 300 µM, respectively, and incubated in the dark. After 6 days, the colony size was measured.

Aspergillus-Pseudomonas co-cultivation

For co-cultivation with *P. fluorescens*, 1×10^6 conidia of each *Aspergillus* strain were spread with sterile glass beads (Ø 2,85 - 3,45 mm, Carl-Roth, Germany) on solid AMM plates. A central agar whole (Ø 1 cm) was filled with 70 µl of *P. fluorescens* CHA0 overnight culture. After 4 days of incubation in the dark, inhibition zones were measured as the distance between the margin of the CHA0 spot and the limit of *Aspergillus* growth area.

Enrichment and isolation of extracellular proteins

For sample preparation, 1 ml conidia suspension (1×10^6 spores/ml) of *A. zonatus*, *A. sydowii*, *A. wentii* and *A. tubingensis* were inoculated in 100 ml liquid Malt-Extract (ME) medium and grown at 30 °C in the light on a rotary shaker (Infors AG, Bottmingen, Switzerland) at 110 rpm for 18 h. Germinated spores were rinsed with sterile deionized water to remove residual ME medium and transferred into 100 ml fresh liquid *Aspergillus* minimal medium (AMM). For stress samples hydrogen peroxide was added to the flasks after 1 h at a final concentration of 2 mM and the incubation was continued for another 2 days in the light. Negative control samples were treated identically without the addition of hydrogen peroxide. Supernatants were separated from mycelium by filtering liquid cultures through Miracloth-filter paper and centrifuged at 4 °C and 4000 x g for 10 min. Subsequently, extracellular proteins were precipitated from the supernatant with 10 % trichloroacetic acid (TCA) in acetone over night at 4 °C. After centrifugation at 4 °C and 4000 x g for 1 h pellets were washed 4 times with 80 % acetone as well as once with 100 % acetone, in order to remove residual water and dried at room temperature (RT). The dry pellets were resolved in 50 µl 8 M urea/ 2 M thiourea and protein concentration determined by Roti-Nanoquant (Roth) according to manufacturer's instructions. Protein samples were loaded onto a 12 % SDS-polyacrylamide gel [27] and stained with Coomassie Brilliant Blue G250 (Sigma). Gels were fixed for 1 h in 40 % ethanol and 10 % acetic acid and washed two times for 20 min with deionized water and subsequently stained for 2 h with the Coomassie staining solution. Residual staining solution was removed by washing the gels three times for 15 min with deionized water. Before mass spectrometrical analyses, SDS-gels were destained with fixing solution until bands were barely visible.

Tryptic digestion, mass spectrometry analysis and protein identification

Lanes of SDS-gels were each cut into 5 pieces of equal size. Gel fragments were digested with trypsin according to [28]. Resulting tryptic peptide mixtures were separated by a reversed-phase liquid chromatographic column (Acclaim PepMap RSLC column, 75 µm x 15 cm, C18, 3 µm, 100 Å, P/N164534, Thermo Fisher Scientific, Waltham, Massachusetts) to further reduce sample complexity prior to mass analyses with an LTQ Velos Pro mass spectrometer (Thermo Fisher Scientific, Waltham, Massachusetts) were used to search for matches against draft genome-wide protein sequence databases (JGI MycoCosm, [29]). The search was performed by a Thermo Proteome Discoverer (version 1.3) workflow that integrates Sequest and Mascot search engines with an initial precursor mass tolerance of 10 ppm and fragment mass tolerance of 0.8 Da. Carbamidomethylcysteine was used as a fixed modification, oxidized methionine was included as variable modification and two miscleavages were allowed for each peptide. For peptide and protein validation, a 0.5 % false discovery rate was set and determined by using peptide validator with a reverse decoy database. Resulting lists of identified proteins were semi-quantitatively processed by a statistical workflow using Marvis Suite [30]. Functional annotation of predicted proteins was carried out by an in-house bioinformatics pipeline

including Interpro [31], NCBI nr standalone blast (http://blast.ncbi.nlm.nih.gov/), SignalP [32]and WoLF PSORT [33].

Susceptibility assay against cell wall stress:

Epsilometer tests (Etest) were performed to determine the susceptibilities of all *Aspergilli* against caspofungin, voriconazole and amphotericin B. A defined number of spores (1 ×106) were spread on complete medium agar plates, the Etest strips (Biomeriuex) laid on top and the plates incubated at 30°C. After 3 days of incubation, the minimal inhibitory concentrations (MIC) were determined. Strains were classified into susceptibility groups according to the MIC: highly sensitive, 0.01 - 0.1 µg/ml; sensitive, > 0.1 - 1 µg/ml; moderate sensitive, > 1 - 10 µg/ml; resistant, > 10 µg/ml. In the case of calcofluor white, complete medium agar was supplemented with 0 or 200 µg/ml calcofluor white and spot inoculated with 1 ×105 spores. After incubation at 30°C for 3 days, the diameters of the colonies were measured. Strains were classified into susceptibility groups according to the calcofluor white-induced growth inhibition: highly sensitive, > 60 – 100 %; sensitive, > 30 – 60 %; moderate sensitive, > 10 - 30 %; resistant, < 10 %. Note that 4 strains were (partially) excluded from the analysis (*A. glaucus, A. wentii, A. zonatus, N. fischeri*) as it was impossible to generate sufficient amount of spores for the susceptibility assays.

References

1. Bayram Ö, Braus GH, Fischer R, Rodriguez-Romero J. Spotlight on Aspergillus nidulans photosensory systems. Fungal Genet. Biol. 2010;47:900–8.

2. Bayram Ö, Braus GH. Coordination of secondarymetabolism and development in fungi: the velvet familyof regulatory proteins. FEMS Microbiol. Rev. 2012;36:1–24.

3. Bayram Ö, Krappmann S, Ni M, Bok JW, Helmstaedt K, Valerius O, et al. VelB/VeA/LaeA Complex Coordinates Light Signal with Fungal Development and Secondary Metabolism. Science. 2008;320:1504–6.

4. Bayram Ö, Biesemann C, Krappmann S, Galland P, Braus GH. More Than a Repair Enzyme: Aspergillus nidulans Photolyase-like CryA Is a Regulator of Sexual Development. Mol. Biol. Cell. 2008;19:3254–62.

5. Halliwell B. Reactive Species and Antioxidants. Redox Biology Is a Fundamental Theme of Aerobic Life. Plant Physiol. 2006;141:312–22.

6. Aguirre J, Hansberg W, Navarro R. Fungal responses to reactive oxygen species. Med. Mycol. 2006;44:101–7.

7. Li Q, Harvey LM, McNeil B. Oxidative stress in industrial fungi. Crit. Rev. Biotechnol. 2009;29:199–213.

8. Morano KA, Grant CM, Moye-Rowley WS. The Response to Heat Shock and Oxidative Stress in Saccharomyces cerevisiae. Genetics. 2012;190:1157–95.

9. Oberegger H, Schoeser M, Zadra I, Abt B, Haas H. SREA is involved in regulation of siderophore biosynthesis, utilization and uptake in Aspergillus nidulans. Mol. Microbiol. 2001;41:1077–89.

10. Navarro RE, Stringer MA, Hansberg W, Timberlake WE, Aguirre J. catA, a newAspergillus nidulans gene encoding a developmentally regulated catalane. Curr. Genet. 1996;29:352–9.

11. Kawasaki L, Wysong D, Diamond R, Aguirre J. Two divergent catalase genes are differentially regulated during Aspergillus nidulans development and oxidative stress. J. Bacteriol. 1997;179:3284–92.

12. Kawasaki L, Aguirre J. Multiple Catalase Genes Are Differentially Regulated in Aspergillus nidulans. J. Bacteriol. 2001;183:1434–40.

13. Thön M, Al-Abdallah Q, Hortschansky P, Brakhage AA. The Thioredoxin System of the Filamentous Fungus Aspergillus nidulans IMPACT ON DEVELOPMENT AND OXIDATIVE STRESS RESPONSE. J. Biol. Chem. 2007;282:27259–69.

14. Coyle CM, Panaccione DG. An Ergot Alkaloid Biosynthesis Gene and Clustered Hypothetical Genes from Aspergillus fumigatus. Appl. Environ. Microbiol. 2005;71:3112–8.

15. Goetz KE, Coyle CM, Cheng JZ, O'Connor SE, Panaccione DG. Ergot cluster-encoded catalase is required for synthesis of chanoclavine-I in Aspergillus fumigatus. Curr. Genet. 2011;57:201–11.

16. Ryan KL, Moore CT, Panaccione DG. Partial Reconstruction of the Ergot Alkaloid Pathway by Heterologous Gene Expression in Aspergillus nidulans. Toxins. 2013;5:445–55.

17. Raaijmakers JM, Mazzola M. Diversity and Natural Functions of Antibiotics Produced by Beneficial and Plant Pathogenic Bacteria. Annu. Rev. Phytopathol. 2012;50:403–24.

18. Kwak Y-S, Han S, Thomashow LS, Rice JT, Paulitz TC, Kim D, et al. Saccharomyces cerevisiae Genome-Wide Mutant Screen for Sensitivity to 2,4-Diacetylphloroglucinol, an Antibiotic Produced by Pseudomonas fluorescens. Appl. Environ. Microbiol. 2011;77:1770–6.

19. Yang F, Cao Y. Biosynthesis of phloroglucinol compounds in microorganisms—review. Appl. Microbiol. Biotechnol. 2011;93:487–95.

20. Finn RD, Mistry J, Tate J, Coggill P, Heger A, Pollington JE, et al. The Pfam protein families database. Nucleic Acids Res. 2010;38:D211–22.

21. Pel HJ, de Winde JH, Archer DB, Dyer PS, Hofmann G, Schaap PJ, et al. Genome sequencing and analysis of the versatile cell factory Aspergillus niger CBS 513.88. Nat. Biotechnol. 2007;25:221–31.

22. Ouedraogo JP, Hagen S, Spielvogel A, Engelhardt S, Meyer V. Survival Strategies of Yeast and Filamentous Fungi against the Antifungal Protein AFP. J. Biol. Chem. 2011;286:13859–68.

23. Galloway LD, Burgess R. Applied mycology and bacteriology. 3rd ed. London: Hill; 1952.

24. Barratt RW, Johnson GB, Ogata WN. Wild-Type and Mutant Stocks of Aspergillus Nidulans. Genetics. 1965;52:233–46.

25. Voisard C, Bull CT, Keel C, Laville J, Maurhofer M, Schnider U, et al. Biocontrol of Root Diseases by Pseudomonas fluorescens CHA0: Current Concepts and Experimental Approaches. Mol. Ecol. Rhizosphere Microorg. Biotechnol. Release GMOs. Wiley-VCH Verlag GmbH; 2007. p. 67–89.

26. Bertani G. Sudies on lysogenesis I. The Mode of Phage Liberation by Lysogenic Escherichia coli. J. Bacteriol. 1951;62:293–300.

27. Laemmli UK. Cleavage of structural proteins during the assembly of the head of bacteriophage T4. Nature. 1970;227:680–5.

28. Shevchenko A, Wilm M, Vorm O, Mann M. Mass spectrometric sequencing of proteins silver-stained polyacrylamide gels. Anal. Chem. 1996;68:850–8.

29. Grigoriev IV, Nikitin R, Haridas S, Kuo A, Ohm R, Otillar R, et al. MycoCosm portal: gearing up for 1000 fungal genomes. Nucleic Acids Res. 2014;42:D699–704.

30. Kaever A, Landesfeind M, Possienke M, Feussner K, Feussner I, Meinicke P, et al. MarVis-Filter: Ranking, Filtering, Adduct and Isotope Correction of Mass Spectrometry Data, MarVis-Filter: Ranking, Filtering, Adduct and Isotope Correction of Mass Spectrometry Data. BioMed Res. Int. BioMed Res. Int. 2012;2012, 2012:e263910.

31. Hunter S, Jones P, Mitchell A, Apweiler R, Attwood TK, Bateman A, et al. InterPro in 2011: new developments in the family and domain prediction database. Nucleic Acids Res. 2012;40:D306–12.

32. Emanuelsson O, Brunak S, von Heijne G, Nielsen H. Locating proteins in the cell using TargetP, SignalP and related tools. Nat. Protoc. 2007;2:953–71.

33. Horton P, Park K-J, Obayashi T, Fujita N, Harada H, Adams-Collier CJ, et al. WoLF PSORT: protein localization predictor. Nucleic Acids Res. 2007;35:W585–7.
