# Supplementary material for: Comparative genomics reveals high biological diversity and specific adaptations in the industrially and medically important fungal genus Aspergillus
Source: Genome Biol. 2017 Feb 14;18:28. doi: 10.1186/s13059-017-1151-0 (PMC5307856; doi:10.1186/s13059-017-1151-0)
Supplement: Additional file 24: — Phylogenetic relationships of UreA homologs. (PDF 108 kb) [file 13059_2017_1151_MOESM24_ESM.pdf]

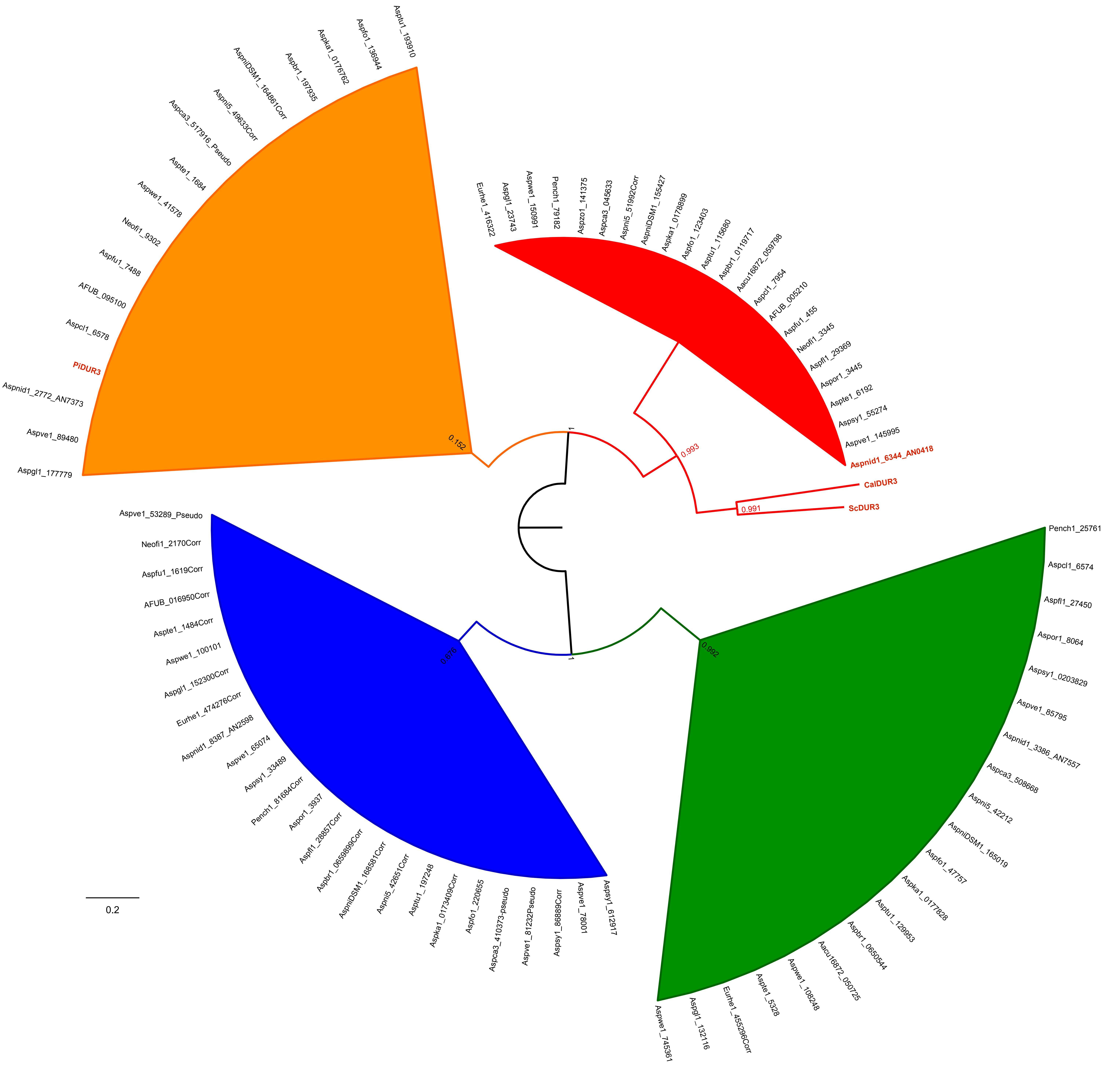

Additional File 24. Phylogenetic relationships of UreA homologs. Two duplication events may have occurred before the different Aspergillus species diverged from a common ancestor, giving rise to two clades, divided in two subclades each. The first sub-clade of clade 1 (shown in red), contains UreA, *S.cerevisiae* DUR3 and *C. albicans* DUR3, as well as an ortholog from each of the fungi analyzed suggesting conservation of urea transport function. The functionally characterized urea/H+ symporter from *P. involutus* is found in the second sub-clade of clade 1 (shown in orange). Clade 2 (subclades shown in blue and green) contains members that have not been characterized functionally. Cartoon drawn with Figtree. Alignments were done with MAFFT Version 7, G-INS-I, curation with MBGE, phylogenies with PhyML. Characterized urea transporters UreA (Aspnid1_6344; AN0418), PiDUR3, ScDUR3 and CaDUR3 are shown in red lettering. Digits at nodes are aLTRs (approximate likelihood ratio values).
