# Supplementary material for: Comparative genomics reveals high biological diversity and specific adaptations in the industrially and medically important fungal genus Aspergillus
Source: Genome Biol. 2017 Feb 14;18:28. doi: 10.1186/s13059-017-1151-0 (PMC5307856; doi:10.1186/s13059-017-1151-0)
Supplement: Additional file 25: — CLUSTAL O(1.2.3) multiple sequence alignment of NNP nitrate transporters. (PDF 149 kb) [file 13059_2017_1151_MOESM25_ESM.pdf]

Additional File 25. CLUSTAL O(1.2.3) multiple sequence alignment of NNP nitrate transporters. Species identification are based on JGI Mycocosm.Numbers after the species identifier indicate the gene id. Aspnid1 = *Aspergillus nidulans*, Aspfu1 = *Aspergillus fumigatus*, Neofi1 = *Aspergillus fischeri*, Aspzo1 = *Aspergillus zonatus*, Aspsy1 = *Aspergillus sydowii*, Aspve1 = *Aspergillus versicolor*, Aspgl1 = *Aspergillus glaucus*, Aspwe1 = *Aspergillus wentii*, Aspcl1 = *Aspergillus clavatus*, Aspac1 = *Aspergillus aculeatus*, Aspca3 = *Aspergillus carbonarius*, Aspbr1 = *Aspergillus brasiliensis*, Aspni DSM 1 = *Aspergillus niger* CBS513.88, Asptu1 = *Aspergillus tubingensis*, Aspfo1 = *Aspergillus luchuensis*, Aspka1 = *Aspergillus luchuensis*, Aspte1 = *Aspergillus terreus*, Aspor1 = *Aspergillus oryzae*, Aspfl1 = *Aspergillus flavus*.

```
jgi|Aspnid1|6363|AN0399 NrtB                            MKPTQVLRLAVAAPDVNPQTRKARSIPVLNPFDLYGRVFFFSWIGFLVAFLSWYAFPPLL
jgi|Aspfu1|1610|Afu1g17470_mRNA                         ---MEILKLAFASPEINPVNHKARSIPVLNPVDRYGRVFFFSWFGFMVAFLSWYAFPPLL
jgi|Neofi1|2173|700000156971211                         ---MEMLKLVFASPEINPVNHKARSIPLLNPVDRYGRVFFFSWLGFMVAFLSWYAFPPLL
jgi|Aspzo1|58567|e_gw1.2.2008.1                         ---MEVLRLAFVSPEINPNNRKAKSIPFFNPFDKYGRVYFFSWLGFMVAFLSWYAFPPLL
jgi|Aspnid1|5705|AN1008 NrtA                            ---MDFAKLLVASPEVNPNNRKALTIPVLNPFNTYGRVFFFSWFGFMLAFLSWYAFPPLL
jgi|Aspsy1|53362|fgenesh1_pm.1_#_405                    ---MGFFKSMVASPEVNPNNRKALSIPILNPLDTYGRVFFFSWFGFMLAFLSWYAFPPLL
jgi|Aspve1|49262|fgenesh1_pm.1_#_1170                   ---MGFFKLLVASPEVNPSNRKALSIPILNPLDAYGRVFFFSWLGFMLAFLSWYAFPPLL
jgi|Aspgl1|28588|fgenesh1_pg.21_#_105                   ---MDFVKLLFISPEVNPSNRKALSIPVFNPFDRYGRVFTFSWLGFMVAFLSWYAFPPLL
jgi|Aspwe1|40392|fgenesh1_kg.4_#_684_#_Locus1608v1rpkm116.27 -MGMEFFKLFVASPDVDPNTRKAKSIPILNPFDRYGRVFFFSWLGFMVAFLSWYAFPPLL
jgi|Aspcl1|7255|700000156880179                         ---MGFIKSLFVSSEINPSNRKARTIPVLNPFDQYGRVFFFSWLGFMVAFLSWYAFPPLL
jgi|Aspfu1|1150|Afu1g12850_mRNA                         --MMNSLKLLVVSPEINPSNRKARSIPVLNPFDRYGRVFFFSWLGFMVAFLSWYAFPPLL
jgi|Neofi1|2646|700000156973616                         ---MNSLKSLVVSPDVNPSNRKARSIPVLNPFDRYGRVFFFSWLGFMVAFLSWYAFPPLL
jgi|Aspac1|38369|Genemark1.545_g                        ---MDFVKLLFVSPEINPSNRKAQSIPVLNPFDRYGRVFFFSWLGFMVAFLSWYAFPPLL
jgi|Aspca3|159116|estExt_Genewise1Plus.C_11082          ---MDYVKLLFVSPEINPSNRKAKSIPIFNPFDKYGRVFFFSWLGFMVAFLSWYAFPPLL
jgi|Aspbr1|41323|fgenesh1_kg.4_#_627_#_Locus1876v1rpkm111.71 ---MDYVKLLFVSPEINPSNRKAQSIPILNPFDKYGRVFFFSWLGFMVAFLSWYAFPPLL
jgi|Aspni_DSM_1|161829|An08g05670m.01                   ---MDRVKLLFASPEINPSNRKAQSIPVLNPFDKYGRVFFFSWLGFMVAFLSWYAFPPLL
jgi|Asptu1|112586|e_gw1.1.743.1                         ---MDHIKLLFVSPEINPSNRKAQSIPILNPFDKYGRVFFFSWLGFMVAFLSWYAFPPLL
jgi|Aspfo1|130828|e_gw1.4.716.1                         ---MDHIKLLFVSPEINPSNRKAQSIPILNPFDKYGRVFFFSWLGFMVAFLSWYAFPPLL
jgi|Aspka1_1|18106|AKAW_06633                           ---MDHIKLLFVSPEVNPSNRKAQSIPILNPFDKYGRVFFFSWLGFMVAFLSWYAFPPLL
jgi|Aspte1|476|ATET_00476                               ---MESIKLLFLSPEINPSNRKAKSIPVLNPFDQYGRVFFFSWLGFLVAFLSWYAFPPLL
jgi|Aspor1|6220|AO090012000623_mRNA                     ---MDSVKLLFLSPEVNPSNRKAMSIPILNPFDKYGRVYFFSWLGFMVAFLSWYAFPPLL
jgi|Aspfl1|27006|7000001155834032                       ---MDSVKLLFLSPEVNPSNRKARSIPILNPFDKYGRVYFFSWLGFMVAFLSWYAFPPLL
                                                               :  . : :::* .:** :**.:**.: ****: ***:**::************

jgi|Aspnid1|6363|AN0399 NrtB                            SVTIKKDLHMSQDDVANSNIVALLGTFVMRFIAGPLCDRFGPRLVFVGLLICGAVPTAMA
jgi|Aspfu1|1610|Afu1g17470_mRNA                         SVTIKKDLHMTQDQVANSNIVALLGTLLMRFIAGPLCDRFGPRYVFVGCLLGGAIPTAMA
jgi|Neofi1|2173|700000156971211                         SVTIKKDLHMTQDQVANSNIVALLGTLLMRFIAGPLCDRFGPRYVFVGCLLCGAVPTAMA
jgi|Aspzo1|58567|e_gw1.2.2008.1                         TDTIKQDLKMTQVDIANSNIVALLATFLVRFVAGPLCDRFGPRLVFVGLLIGGSIPTAMA
jgi|Aspnid1|5705|AN1008 NrtA                            TVTIRDDLDMSQTQIANSNIIALLATLLVRLICGPLCDRFGPRLVFIGLLLVGSIPTAMA
jgi|Aspsy1|53362|fgenesh1_pm.1_#_405                    TVTIRDDLEMSQTQIANSNIIALLATLLVRFVCGPLCDRFGPRLVFIGLLLVGSIPTAMA
jgi|Aspve1|49262|fgenesh1_pm.1_#_1170                   TVTIRDDLHMSQTQIANSNIIALLATLLVRFVCGPMCDRFGPRLVFVGLLLVGSIPTAMA
jgi|Aspgl1|28588|fgenesh1_pg.21_#_105                   TATIKDDLGMTQQQVANSNIVALLATLIVRLIAGPLCDRFGPRLVFVGLLLCGSIPTAMA
jgi|Aspwe1|40392|fgenesh1_kg.4_#_684_#_Locus1608v1rpkm116.27 TVTIRQDLHMTQQDVANSNIIALLATFLVRFIAGPLCDRFGPRLVFVGLLLCGSIPTAMA
jgi|Aspcl1|7255|700000156880179                         TVTIRNDLHMTQQQVANSNIVALLATFLVRFIAGPLCDRFGPRLVFIGLLLCGSIPTAMA
jgi|Aspfu1|1150|Afu1g12850_mRNA                         TVTIRKDLHMTQQQVANSNIVALLATFLVRFIAGPLCDRFGPRLVFIGLLLCGSIPTAMA
jgi|Neofi1|2646|700000156973616                         TVTIRKDLHMTQQQVANSNIVALLATFLVRFIAGPLCDRFGPRLVFIGLLLCGSIPTAMA
jgi|Aspac1|38369|Genemark1.545_g                        TVTIRKDLHMTQQDVANSNIVALLATLLVRFVAGPLCDRFGPRLVFIGLLLSGSIPTAMA
jgi|Aspca3|159116|estExt_Genewise1Plus.C_11082          TDTIRQDLHMTQTQVANSNIVALLATLLVRFVAGPLCDRFGPRLVFIGLLLCGSIPTAMA
jgi|Aspbr1|41323|fgenesh1_kg.4_#_627_#_Locus1876v1rpkm111.71 TDTIRQDLHMTQTQVANSNIVALLATLLVRFIAGPLCDKFGPRLVFIGLLLCGSIPTAMA
jgi|Aspni_DSM_1|161829|An08g05670m.01                   TDTIRQDLNMTETQVANSNIVALLATLLVRFVAGPLCDKFGPRLVFIGLLLCGSIPTAMA
jgi|Asptu1|112586|e_gw1.1.743.1                         TDTIRQDLKMTETQVANSNIVALLATLLVRFVAGPLCDKFGPRLVFIGLLLCGSIPTAMA
jgi|Aspfo1|130828|e_gw1.4.716.1                         TDTIRQDLNMTETQVANSNIVALLATLLVRFVAGPLCDKFGPRLVFIGLLLCGSIPTAMA
jgi|Aspka1_1|18106|AKAW_06633                           TDTIRQDLNMTETQVANSNIVALLATLLVRFVAGPLCDKFGPRLVFIGLLLCGSIPTAMA
jgi|Aspte1|476|ATET_00476                               TVTISKDLHMTQNDIANSNIVALLATFLVRFVAGPLCDRFGPRYVFIGLLLAGSIPTAMA
jgi|Aspor1|6220|AO090012000623_mRNA                     TVTIRKDLKMTQPEVANSNIVALLATLLVRFVAGPLCDRFGPRLVFIGLLLCGSIPTAMA
jgi|Aspfl1|27006|7000001155834032                       TVTIRKDLKMTQPEVANSNIVALLATLLVRFVAGPLCDRFGPRLVFIGLLLCGSIPTAMA
                                                        : ** .** *:: ::*****:***.*:::*::.**:**:**** **:* *: *::*****
```

```
jgi|Aspnid1|6363|AN0399 NrtB                                   GLVTTPQGLIALRFFVGILGATFVPCQVWCTGFFDKNIVGTANSLAGGFGNAGGGITYFV
jgi|Aspfu1|1610|Afu1g17470_mRNA                                GLVTSPQGLIALRFFIGILGATFVPCQVWCTGFFDKNVVGTANALAGGFGNGGGGITYFV
jgi|Neofi1|2173|7000001156971211                               GLVTSPQGLIALRFFIGILGATFVPCQVWCTGFFDKNVVGTANALAGGFGNAGGGITYFV
jgi|Aspzo1|58567|e_gw1.2.2008.1                                GLVTTPSGLIALRFFVGILGATFVPCQVWCTGFFDKNIVGTANALAAGWGNAGGGITYFV
jgi|Aspnid1|5705|AN1008 NrtA                                   GLVTSPQGLIALRFFIGILGGTFVPCQVWCTGFFDKSIVGTANSLAAGLGNAGGGITYFV
jgi|Aspsy1|53362|fgenesh1_pm.1_#_405                           GLVTTPQGLIALRFFIGILGGTFVPCQVWCTGFFDKKVVGTANSLAAGLGNAGGGITYFV
jgi|Aspve1|49262|fgenesh1_pm.1_#_1170                          GLVTTPEGLIALRFFIGILGGTFVPCQVWCTGFFDKKVVGTANSLAAGLGNAGGGITYFV
jgi|Aspgl1|28588|fgenesh1_pg.21_#_105                          GLVTNPQGLIALRFFVGILGGTFVPCQVWCTGFFDKKIVGTANSLAGGWGNSGGGITYFI
jgi|Aspwe1|40392|fgenesh1_kg.4_#_684_#_Locus1608v1rpkm116.27   GLVTTPKGLIALRFFVGILGGTFVPCQVWCTGFFDKKIVGTANSLAAGWGNSGGGITYFV
jgi|Aspcl1|7255|7000001156880179                               GLVTNPNGLIALRFFIGILGGTFVPCQVWCTGFFDKNIIGTANSLAAGWGNAGGGITYFL
jgi|Aspfu1|1150|Afu1g12850_mRNA                                GLVTTPNGLIALRFFVGILGGTFVPCQVWCTGFFDKNIVGTANSLAAGWGNAGGGITYFV
jgi|Neofi1|2646|7000001156973616                               GLVTTPNGLIALRFFVGILGGTFVPCQVWCTGFFDKNIVGTANSLAAGWGNAGGGITYFV
jgi|Aspac1|38369|Genemark1.545_g                               GLVTTPKGLIALRFFIGILGGTFVPCQVWCTGFFDKKIVGMANALAGGWGNAGGGITYFV
jgi|Aspca3|159116|estExt_Genewise1Plus.C_11082                 GLVTTPEGLIALRFFVGILGGTFVPCQVWCTGFFDKKIVGAANALAGGWGNSGGGITFFV
jgi|Aspbr1|41323|fgenesh1_kg.4_#_627_#_Locus1876v1rpkm111.71   GLVTTPNGLIALRFFVGILGGTFVPCQVWCTGFFDKKIVGAANALAGGWGNAGGGITFFV
jgi|Aspni_DSM_1|161829|An08g05670m.01                          GLVTTPNGLIALRFFVGILGGTFVPCQVWCTGFFDKKIVGTANALAGGWGNAGGGITFFV
jgi|Asptu1|112586|e_gw1.1.743.1                                GLVTTPNGLIALRFFVGILGGTFVPCQVWCTGFFDKKIVGTANALAGGWGNAGGGITFFV
jgi|Aspfo1|130828|e_gw1.4.716.1                                GLVTTPNGLIALRFFVGILGGTFVPCQVWCTGFFDKKIVGTANALAGGWGNAGGGITFFV
jgi|Aspka1_1|18106|AKAW_06633                                  GLVTTPNGLIALRFFVGILGGTFVPCQVWCTGFFDKKIVGTANALAGGWGNAGGGITFFV
jgi|Aspte1|476|ATET_00476                                      GLVTNSRGLIALRFFVGVLGGTFVPCQVWCTGFFDKKIVGTANSLAAGWGNAGGGITYFV
jgi|Aspor1|6220|AO090012000623_mRNA                            GLVTNAQGLIALRFFVGILGGTFVPCQVWCTGFFDKKIVGTANSLAAGWGNAGGGITYFV
jgi|Aspfl1|27006|7000001155834032                              GLVTNAQGLIALRFFVGILGGTFVPCQVWCTGFFDKKIVGTANSLAAGWGNAGGGITYFV
                                                               ****.  ********:*:**.***************.::* **:**.* **.*****:*:

jgi|Aspnid1|6363|AN0399 NrtB                                   MPAIYDSFVHDRGLTPHKAWRVSYIVPFIIIVSIALAMLFTCPDTPTGKWADREKT----
jgi|Aspfu1|1610|Afu1g17470_mRNA                                MPAIYDSLVHSQGLTPHRAWRVAYVAPFIIIVTLALAMLFLCEDTPTGKWSQRNDN----
jgi|Neofi1|2173|7000001156971211                               MPAIYDSLVHSQGLTPHRAWRVAYVVPFIIIVTLALSMLFLCEDTPTGKWSQRNDN----
jgi|Aspzo1|58567|e_gw1.2.2008.1                                MPAIFDSLVHSQGLSAHKAWRVAYIVPFVLINVIALAMLFTCEDTPTGKWSERQIWMKEE
jgi|Aspnid1|5705|AN1008 NrtA                                   MPAIFDSLIRDQGLPAHKAWRVAYIVPFILIVAAALGMLFTCDDTPTGKWSERHIWMKED
jgi|Aspsy1|53362|fgenesh1_pm.1_#_405                           MPAIFDSLVHNQGLPAHKAWRVAYIVPFILIVAAALGMLFTCDDTPTGPWSERHIWTKEE
jgi|Aspve1|49262|fgenesh1_pm.1_#_1170                          MPAIFDSLVHSQGLAAHKAWRVAYIVPFILIVATALGMLFTCDDTPTGPWSQRHIWTKEE
jgi|Aspgl1|28588|fgenesh1_pg.21_#_105                          MPTVYDSLVHSQGLAPHKAWRVAYVIPFIIIVALAVAMLFTCDDTPMGKWSERHIWMKEN
jgi|Aspwe1|40392|fgenesh1_kg.4_#_684_#_Locus1608v1rpkm116.27   MPAIYDSLVHTSGLPPHKAWRVSYVVPFIIIVSLALCMLFTCEDTPTGKWSERHIWMKDQ
jgi|Aspcl1|7255|7000001156880179                               MPAIFDSLVHSRGLAPHKAWRVAYVVPFCIIVVLALCMLLTCEDTPTGKWSERHIWMKES
jgi|Aspfu1|1150|Afu1g12850_mRNA                                MPAIYDSLVHSQGLAPHKAWRVAYIVPFIIIVVLALCMLLICEDTPTGKWSERHIWMKEN
jgi|Neofi1|2646|7000001156973616                               MPAIYDSLVHSQGLAPHKAWRVAYIVPFIIIVVLALCMLLICEDTPTGKWSERHIWMKEN
jgi|Aspac1|38369|Genemark1.545_g                               MPAIYDSLVYSQGLTPHKAWRVAFIVPFIIIVVIAVAMLFLCEDTPTGKWSERHLWAREM
jgi|Aspca3|159116|estExt_Genewise1Plus.C_11082                 MPAVYDSLVNVQGLTPHKAWRVSYIVPFIIIVAVALTMLFVCEDTPTGKWSERHLWVKEA
jgi|Aspbr1|41323|fgenesh1_kg.4_#_627_#_Locus1876v1rpkm111.71   MPAVYDSLVKAQGLTPHKAWRVAYIVPFIIIVGVALTMLFVCEDTPTGKWSERHLWVKEA
jgi|Aspni_DSM_1|161829|An08g05670m.01                          MPAVYDSLVKVQGLTPHKAWRVAYIVPFIIIVGVALTMLFVCEDTPTGKWSERHLWVKEA
jgi|Asptu1|112586|e_gw1.1.743.1                                MPAVYDSLVKVQGLPPHKAWRVAYIVPFIIIVGVALTMLFVCEDTPTGKWSERHLWVKEA
jgi|Aspfo1|130828|e_gw1.4.716.1                                MPAVYDSLVKVQGLPPHKAWRVAYIVPFIIIVGVALTMLFVCEDTPTGKWSERHLWVKEA
jgi|Aspka1_1|18106|AKAW_06633                                  MPAVYDSLVKVQGLPPHKAWRVAYIVPFIIIVGVALTMLFVCEDTPTGKWSERHLWVKEA
jgi|Aspte1|476|ATET_00476                                      MPAIFDSLVHAQGLPAHKAWRVAYVVPFIIIVALALGMLFTCEDTPTGKWSERHLWASEN
jgi|Aspor1|6220|AO090012000623_mRNA                            MPAIFDSLVHNQSLPAHKAWRVAYIVPFIIIVVIAVAMFFTCEDTPTGKWSERHLWAEET
jgi|Aspfl1|27006|7000001155834032                              MPAIFDSLVHNQGLPAHKAWRVAYIVPFIIIVVIAVAMFFTCEDTPTGKWSERHLWAEET
                                                               **:::**::   .*  *:****::: ** :*   *: *:: * *** * *::*.
```

```
jgi|Aspnid1|6363|AN0399_NrtB                                  ----SGQSIVDLSSTPNAS----SANSINI---SSDEKKAVHPE--VTDSEAQVHVRAGQ
jgi|Aspfu1|1610|Afu1g17470_mRNA                               -NPVMQSNIVDLHRPSA----SGTPSSINVST--VPEKKGVATPQ-IVDTEAQPVID-IS
jgi|Neofi1|2173|7000001156971211                              -NPIIQSNIVDLHRSSG----SGAPSSTNVST--VPEKKGVSTPQ-IVDTEAQPVID-IS
jgi|Aspzo1|58567|e_gw1.2.2008.1                               HTTGS-ENVIDLDTQSP-DGPSGSPSVMNVPT--DIEKKGAQTPV-TIDRE-AQTGQ-VD
jgi|Aspnid1|5705|AN1008_NrtA                                  TQTASKGNIVDLSSGAQSSRPSGPPSIIAYAIPDVE-KKGTETPL-EPQ--SQAIGQ-FD
jgi|Aspsy1|53362|fgenesh1_pm.1_#_405                          T--RSQGNIVDLNSGNQSTQPSGSPSIITHVAVDVE-KKGVQSPI-ESDGTTQVMGQ-LD
jgi|Aspve1|49262|fgenesh1_pm.1_#_1170                         T--HSQGNIVDLNSGSQSAQPSGSPSIITNIAVDVE-KKGIQSPI-ESDGT-QVMGQ-LD
jgi|Aspgl1|28588|fgenesh1_pg.21_#_105                         SPQAANANIVDITSDNPSSRTSGAPSISQVVAVSDVEKKGAETPNNNIDTEAQTTGQ-VG
jgi|Aspwe1|40392|fgenesh1_kg.4_#_684_#_Locus1608v1rpkm116.27  E--VPEGNIVNINSVNPS----SPPSVNNFMT-PDLEKKGAQSPE-VVDQ-GQAVGQ-VD
jgi|Aspcl1|7255|7000001156880179                              A--ESDGNIISLDSGSPSVRPSGPPS-INLPT-PPTEKKGVQTPQ-VVDPEAQAVGE-VD
jgi|Aspfu1|1150|Afu1g12850_mRNA                               N--ESQANIVNLMSGSPSTRPSGPPS-INIST-PQSEKKGAQTPQ-MVDPEAQAVGQ-VD
jgi|Neofi1|2646|7000001156973616                              N--ESQANIVNLMSGSPSTRPSGPPS-INIST-PQSEKKGAQTPQ-MVDPEAQAVGQ-VD
jgi|Aspac1|38369|Genemark1.545_g                              AG-ESDGNIVDLKADMRSAQPSSTPSMTNVMA-DVEKKG-AQTPV-SLGSNSQTIAE-FD
jgi|Aspca3|159116|estExt_Genewise1Plus.C_11082                T--GSDGNIIDINSGTLSS-PSKSPSMSDVMG-DVEKKGGTQTPV-SLEPESQRIGQ-FD
jgi|Aspbr1|41323|fgenesh1_kg.4_#_627_#_Locus1876v1rpkm111.71  T--ESEGNIIDINSGIPSTRESKAPSVSDMMA-DVEKKGGAQTPM-SLGAESQQIGQ-FD
jgi|Aspni_DSM_1|161829|An08g05670m.01                         T--ESEGKIINVNSGIPS----KAPSVSDMMA-DVEKKGGAQTPI-SLGAESQQIGQ-FD
jgi|Asptu1|112586|e_gw1.1.743.1                               T--ESEGNIIDINSGIPS----KAPSVSDMMA-DVEKKGGAQTPM-SLGAESQQIGQ-FD
jgi|Aspfo1|130828|e_gw1.4.716.1                               T--ESEGNIIDINSGIPS----KAPSVSDMMA-DVEKKGGAQTPM-SLGAESQQIGQ-FD
jgi|Aspka1_1|18106|AKAW_06633                                 T--ESEGNIIDINSGIPS----KAPSVSDMMA-DVEKKGGAQTPM-SLGAESQQIGQ-FD
jgi|Aspte1_476|ATET_00476                                     D--GSDPNIVDLTSGRTSVRPSVAPSLTNIAV-DVEKKGTDTPVSTALDREPQMLGQ-MD
jgi|Aspor1|6220|AO090012000623_mRNA                           S--RFEGNIVNINSGISSSHPSSPPSTTNIVA-DLEKKGNPSPPES----IAPMPGQ-LE
jgi|Aspfl1|27006|7000001155834032                             S--RFEGNIVNINSGISSSHPSSPPSTTNIVA-DLEKKGNPSPPES----IAPMPGQ-LE
                                                                     .::.:             .           *

jgi|Aspnid1|6363|AN0399_NrtB                                  IESSDAVIEAPTIKRYLSIALDPSALAVAVPYACSFGAELAINSILGAYYLLNFPLLGQT
jgi|Aspfu1|1610|Afu1g17470_mRNA                               IANNET-IKNPTLREALRVAFSLPTLALALPYACSFGAELAINSMLGAYYLKNFPSLGQT
jgi|Neofi1|2173|7000001156971211                              LANNEM-IQKPTLREALRVIFSLPTLALALPYACSFGAELAINSVLGAYYLKNFPSLGQT
jgi|Aspzo1|58567|e_gw1.2.2008.1                               FYNREV-VVAPTFKEALGVIFSLSTMAVALPYACSFGTELALDSILGSYYTDNFPNLGQT
jgi|Aspnid1|5705|AN1008_NrtA                                  AFRANA-VASPSRKEAFNVIFSLATMAVAVPYACSFGSELAINSILGDYYDKNFPYMGQT
jgi|Aspsy1|53362|fgenesh1_pm.1_#_405                          ALRTDA-VIAPSRKEAMSVVLSLASLAVAIPYACSFGSELAINSILGDYYDKNFPYMGQT
jgi|Aspve1|49262|fgenesh1_pm.1_#_1170                         AIQTDA-VVSPSRKEAMKVVFSLASLAVAIPYACSFGSELAINSILGDYYDKNFPYMGQT
jgi|Aspgl1|28588|fgenesh1_pg.21_#_105                         ILDTTTTPPTPSHKETLSIMFSLSTLAVAVPYACSFGSELSINSILGDYYAQNFPYMSQT
jgi|Aspwe1|40392|fgenesh1_kg.4_#_684_#_Locus1608v1rpkm116.27  IFKKDT-VVAPSRKEAMNVILSLSTFAVAIPYACSFGAELSVNSILGDYYSENFPHMGQT
jgi|Aspcl1|7255|7000001156880179                              VLRTDA-VVSPSRREILNVLLSLSTAAVAIPYACSFGSELAINSILGEFYSDNFPHLGQT
jgi|Aspfu1|1150|Afu1g12850_mRNA                               ILRADT-VVAPSRKEVMNVLLSLSTAAVAIPYACSFGSELAINSILGEFYSKNFPDMSQT
jgi|Neofi1|2646|7000001156973616                              ILRTDT-VVAPSRKEVMNVLLSLSTAAVAIPYACSFGSELAINSILGEFYSKNFPDMGQT
jgi|Aspac1|38369|Genemark1.545_g                              AIRTDT-VVAPTRKEAMGVLFSLSTAAVAIPYACSFGAELAINSNLGEYYSANFPQMGQT
jgi|Aspca3|159116|estExt_Genewise1Plus.C_11082                ALRTDM-VVAPTRKEALNVLFSLSTAAVAIPYACSFGAELAIESVLANYYLDNFPSMGQT
jgi|Aspbr1|41323|fgenesh1_kg.4_#_627_#_Locus1876v1rpkm111.71  ALRTDT-VVAPTRKEALNVLFSMFTAAVAIPYACTFGAELAIESVLANYYVANFPSMGQT
jgi|Aspni_DSM_1|161829|An08g05670m.01                         ALRTDT-VVAPTRKEALNVLFSMFTAAVAIPYACTFGAELAIESVLANYYVANFPSMGQT
jgi|Asptu1|112586|e_gw1.1.743.1                               ALRTDT-VVAPTRKEALNVLFSMFTAAVAIPYACTFGAELAIESVLANYYVANFPSMGQT
jgi|Aspfo1|130828|e_gw1.4.716.1                               ALRTDT-VVAPTRKEALNVLFSMFTAAVAIPYACTFGAELAIESVLANYYVANFPSMGQT
jgi|Aspka1_1|18106|AKAW_06633                                 ALRTDT-VVAPTRKEALNVLFSMFTAAVAIPYACTFGAELAIESVLANYYVANFPSMGQT
jgi|Aspte1_476|ATET_00476                                     ALRTET-VVAPSFKEALRVIFNLHTAAVALPYACSFGAELSINSILGKYYADNFPHMGQT
jgi|Aspor1|6220|AO090012000623_mRNA                           SLRTDT-VVAPTFKEAMNVLLSLSTAAVAIPYACSFGAELAINSILGDFYAENFPYMGQT
jgi|Aspfl1|27006|7000001155834032                             SLRTDT-VVAPTFKEAMNVLLSLSTAAVAIPYACSFGAELAINSILGDFYAENFPYMGQT
                                                                        *: :. : : :.  : *:*:****:**:**:::* *. :*  *** :.**
```

```
jgi|Aspnid1|6363|AN0399_NrtB                                        QSGRWASMFGLVNVVFRPMGGFIADLIYARTNSVWAKKMWLVVLGLAMSGMAILIGFLDP
jgi|Aspfu1|1610|Afu1g17470_mRNA                                     KSGRWAAMFGLVNVVFRPAGGFMADVIYRYTRSVWGKKVWLVCLGVCMGAAALAVGLLDP
jgi|Neofi1|2173|7000001156971211                                    KSGRWAAMFGLVNVVFRPAGGFMADLIYRYTQSVWGKKVWLVCLGVCMGGSALAVGLLDP
jgi|Aspzo1|58567|e_gw1.2.2008.1                                     RSGQWAAMFGLLNVVFRPAGGLVADFLYKRTNSIWAKKIWLVSLSIIMGAFLLAIGLTDP
jgi|Aspnid1|5705|AN1008_NrtA                                        QTGKWAAMFGFLNIVCRPAGGFLADFLYRKTNTPWAKKLLLSFLGVVMGAFMIAMGFSDP
jgi|Aspsy1|53362|fgenesh1_pm.1_#_405                                QTGKWAAMFGFLNIVCRPAGGFLADILYNYSETLWSKKLLLSFLGVVMGAFMLAMGLSDP
jgi|Aspve1|49262|fgenesh1_pm.1_#_1170                               QTGKWAAMFGFLNVICRPAGGFLADILYNYSETLWSKKLLLSFLGVIMGAFMLAMGLSDP
jgi|Aspgl1|28588|fgenesh1_pg.21_#_105                               QTGQWAAMFGLLNIVCRPAGGFVADLVYRYTETVWSKKILLVFLGVIMGAFQLAMGLSDP
jgi|Aspwe1|40392|fgenesh1_kg.4_#_684_#_Locus1608v1rpkm116.27        RTGQWAAMFGLLNVVFRPAGGFLADVLYQYTDSVWSKKLLLSFLGVVMGAFLLALGLADP
jgi|Aspcl1|7255|7000001156880179                                    ESGRWAAMFGLLNVVFRPAGGFIADMLYNHTQAVWSKKLFMSFLGIVMGAFLLALGLTDP
jgi|Aspfu1|1150|Afu1g12850_mRNA                                     ESGRWAAMFGLLNVVFRPAGGFIADMLYKYTGAVWSKKVLLSFLGVVMGAFQLAIGLADP
jgi|Neofi1|2646|7000001156973616                                    ESGRWAAMFGLLNVVFRPAGGFIADMLYKYTGAVWSKKLLLSFLGVVMGAFQLAIGLTDP
jgi|Aspac1|38369|Genemark1.545_g                                    KTGQWAAMFGLLNVVFRPAGGFLADLLLRRTQNLWSKKILLVFLGVIMGAFELAIGLTNP
jgi|Aspca3|159116|estExt_Genewise1Plus.C_11082                      ESGRWAAMFGLLNIVCRPAGGFLADYLYRSTQSLWSKKILLVFLGVVMGAFQLAIGFTNP
jgi|Aspbr1|41323|fgenesh1_kg.4_#_627_#_Locus1876v1rpkm111.71        ESGRWAAMFGLLNIICRPAGGFLADYLYRVTQSLWSKKVLLVFLGLVTGAFQLAIGLTNP
jgi|Aspni_DSM_1|161829|An08g05670m.01                               ESGRWAAMFGLLNIICRPAGGFLADFLYRATQSLWSKKILLVCLGLVTGAFQLAIGLTNP
jgi|Asptu1|112586|e_gw1.1.743.1                                     ESGRWAAMFGLLNIICRPAGGFVADFLYRTTQSLWSKKILLVSLGLITGAFQLAIGLTNP
jgi|Aspfo1|130828|e_gw1.4.716.1                                     ESGRWAAMFGLLNIICRPAGGFVADFLYRTTQSLWSKKILLVSLGLITGAFQLAIGLTNP
jgi|Aspka1_1|18106|AKAW_06633                                       ESGRWAAMFGLLNIICRPAGGFVADFLYRTTQSLWSKKILLVSLGLITGAFQLAIGLTNP
jgi|Aspte1_476|ATET_00476                                           KSGQWAAMFGLLNVIFRPAGGFIADLLYRHTDSVWSKKIFLSFLGVVMGAFQVAMGLTNP
jgi|Aspor1|6220|AO090012000623_mRNA                                 KTGQWAAMFGLLNVICRPAGGFIADLLYRHTQSVWSKKILLSFLGVGMGAFQLALGFSNP
jgi|Aspfl1|27006|7000001155834032                                   KTGQWAAMFGLLNVICRPAGGFIADLLYRHTQSVWSKKILLSFLGVGMGAFQLALGFSNP
                                                                    .:*:**:***::*:: ** **::** :   :   *.**: :  *.:  ..  : :*: :*

jgi|Aspnid1|6363|AN0399_NrtB                                        HRESVMFGLVVLMAFFIAASNGANFAIVPHVHPSANGIVSGIVGGMGNFGGIIFAIVFRY
jgi|Aspfu1|1610|Afu1g17470_mRNA                                     KREGLMFGLVVLMAFFIAASNGANFAIVPHVHPTANGIVSGFVGGMGNFGGIAFAIIFRY
jgi|Neofi1|2173|7000001156971211                                    KSEGLMFGLVVLMAFFIAASNGANFAIVPHVHPTANGIVSGIVGGMGNFGGIVFAIIFRY
jgi|Aspzo1|58567|e_gw1.2.2008.1                                     KSQSTMFGLAAGLAFFTEAANGANFAVVPHVHPFANGIVSGAVGGMGNFGGIIFAIVFRY
jgi|Aspnid1|5705|AN1008_NrtA                                        KSEATMFGLTAGLAFFLESCNGAIFSLVPHVHPYANGIVSGMVGGFGNLGGIIFAIIFRY
jgi|Aspsy1|53362|fgenesh1_pm.1_#_405                                TSEATMFGLTAGLAFFLEACNGAIFALVPHVHPFANGIVSGIVGGFGNLGGIIFAIIFRY
jgi|Aspve1|49262|fgenesh1_pm.1_#_1170                               TSEATMFGLTAGLAFFLEACNGANFALVPHVHPFANGIVSGMVGGFGNLGGIIFAIIFRY
jgi|Aspgl1|28588|fgenesh1_pg.21_#_105                               KDEATMFGLTAGLAFFLEACNGANFALVPHVHSFANGIVSGTVGGMGNLGGIIFAIIFRY
jgi|Aspwe1|40392|fgenesh1_kg.4_#_684_#_Locus1608v1rpkm116.27        KTEAAMFGLSAGLAFFLESCNGVIFAVVPHVHPFANGIVSGVVGGMGNLGGIIFAIIFRY
jgi|Aspcl1|7255|7000001156880179                                    KSEATMFGLTAGLAFFLEACNGANFAVVPHVHPFANGVVSGAVGGMGNFGGIIFAIIFRY
jgi|Aspfu1|1150|Afu1g12850_mRNA                                     RSEATMFGLTAGLAFFLEACNGTNFAVVPHVHPFANGVVSGAVGGMGNLGGIIFAIIFRY
jgi|Neofi1|2646|7000001156973616                                    RSEATMFGLTAGLAFFLEACNGANFAVVPHVHPFANGVVSGAVGGMGNLGGIIFAIIFRY
jgi|Aspac1|38369|Genemark1.545_g                                    KSESTMFGLIAGLAFFLESCNGANFALVPHVYPFANGVVSGAVGGMGNLGGIIFAIIFRY
jgi|Aspca3|159116|estExt_Genewise1Plus.C_11082                      KSEATMFGLMAGLAFFLEACNGAIFSLVPHVYPFANGVVSGAVGGMGNLGGIIFAIIFRY
jgi|Aspbr1|41323|fgenesh1_kg.4_#_627_#_Locus1876v1rpkm111.71        KSESTMFGLMAGLAFFLEACNGAIFSLVPHVYPFANGVVSGAVGGMGNFGGIIFAIIFRY
jgi|Aspni_DSM_1|161829|An08g05670m.01                               KSEATMFGLMAGLAFFLEACNGAIFSLVPHVYPFANGVVSGAVGGMGNFGGIIFAIIFRY
jgi|Asptu1|112586|e_gw1.1.743.1                                     KTEATMFGLLAGLAFFLEACNGAIFSLVPHVYPFANGVVSGAVGGMGNFGGIIFAIIFRY
jgi|Aspfo1|130828|e_gw1.4.716.1                                     KTEATMFGLMAGLAFFLEACNGAIFSLVPHVYPFANGVVSGAVGGMGNFGGIIFAIIFRY
jgi|Aspka1_1|18106|AKAW_06633                                       KTEATMFGLMAGLAFFLEACNGAIFSLVPHVYPFANGVVSGAVGGMGNFGGIIFAIIFRY
jgi|Aspte1_476|ATET_00476                                           KTEATMFGLTAGMAFFLEAANGANFAVVPHVYPFANGIVSGAVGGMGNFGGIIGAIIFRY
jgi|Aspor1|6220|AO090012000623_mRNA                                 KSEATMFGLTAGLAFFLEACNGANFAVVPHVHPFANGIVSGAVGGMGNLGGIIFAIIFRY
jgi|Aspfl1|27006|7000001155834032                                   KSEATMFGLTAGLAFFLEACNGANFAVVPHVHPFANGIVSGAVGGMGNLGGIIFAIIFRY
                                                                      :. **** . :***  :.**. *::****:  ***:*** ***:**:***  **:***
```

```
jgi|Aspnid1|6363|AN0399 NrtB                                     NGTQYHRSLWIIGFIILGCTLFFSWVRPVPKQNH*---
jgi|Aspfu1|1610|Afu1g17470_mRNA                                  NGTHYDRALWIIGAIVAGVNICVSWIRPVPRT*-----
jgi|Neofi1|2173|7000001156971211                                 NGTHYDRALWIIGAIAAGVNICVSWIRPVPRT*-----
jgi|Aspzo1|58567|e_gw1.2.2008.1                                  NGTNYARGMWIIGVISLGVNLSMSWVRPMPRSEMP*--
jgi|Aspnid1|5705|AN1008 NrtA                                     SHHDYARGIWILGVISMAVFISVSWVRPVPKSQMRE*-
jgi|Aspsy1|53362|fgenesh1_pm.1_#_405                             SHHDYARGIWILGVISMAANIAVAWIRPVPKSQMRE*-
jgi|Aspve1|49262|fgenesh1_pm.1_#_1170                            SHHDYARGIWILGVISIAANIAVAWIRPVPKSQMQD*-
jgi|Aspgl1|28588|fgenesh1_pg.21_#_105                            NASHYARSLWIIGVICIAGNMLLSWIRPVPRGQTTRS*
jgi|Aspwe1|40392|fgenesh1_kg.4_#_684_#_Locus1608v1rpkm116.27     NGGHYPRSLWIIGVIAIAGNLVVSWINPVPRSQRVRS*
jgi|Aspcl1|7255|7000001156880179                                 NGTHYARSLWIIGVISIAANLAVAWIRPVPKSQMQRQ*
jgi|Aspfu1|1150|Afu1g12850_mRNA                                  NETHYSRSLWIIGVISIAANLAVSWIRPVPKSQMMRQ*
jgi|Neofi1|2646|7000001156973616                                 NETHYSQSLWIIGVISIAANLAVSWIRPVPKSQMMRQ*
jgi|Aspac1|38369|Genemark1.545_g                                 NGSDYSRSLWIIGAISIGANVLVSWIRPVSKKQVVQR*
jgi|Aspca3|159116|estExt_Genewise1Plus.C_11082                   NGSHYARSLWIIGVISLAANVVTSWIRPVAKNPVVSQ*
jgi|Aspbr1|41323|fgenesh1_kg.4_#_627_#_Locus1876v1rpkm111.71     NGSHYAQSLWIIGAISLAANVGISWIRPVAKNPVVKQ*
jgi|Aspni_DSM_1|161829|An08g05670m.01                            NGSHYARSLWIIGVISLAANVCISWIRPVAKNPVVRQ*
jgi|Asptu1|112586|e_gw1.1.743.1                                  NGSHYAQSLWIIGAISLAANVGISWIRPVAKNPVVRQ*
jgi|Aspfo1|130828|e_gw1.4.716.1                                  NGSHYAQSLWIIGAISLAANVGISWIRPVAKNPVVRQ*
jgi|Aspka1_1|18106|AKAW_06633                                    NGSHYAQSLWIIGAISLAANVGISWIRPVAKNPVVRQ*
jgi|Aspte1|476|ATET_00476                                        NGHDYARSLWIIGVLCIATNLLVAWIRPVPREQMVR*-
jgi|Aspor1|6220|AO090012000623_mRNA                              NGSHYARSLWIIGIIAIAANLAVSWIRPVPRRQMV*--
jgi|Aspfl1|27006|7000001155834032                                NGSHYARSLWIIGIIAIAANLAVSWIRPVPRPQMV*--
                                                                 .  .* :.:**:* :  .  :  :*:.*: :
```

Pairwise Similarity % using EMBOSS Water (Smith-Waterman algorithm)

	NrtA	NrtB
jgi|Aspnid1|6363|AN0399 NrtB	75.0	100
jgi|Aspfu1|1610|Afu1g17470_mRNA	75.8	82.5
jgi|Neofi1|2173|7000001156971211	76.6	83.6
jgi|Aspzo1|58567|e_gw1.2.2008.1	83.0	80.1
jgi|Aspnid1|5705|AN1008 NrtA	100	75.0
jgi|Aspsy1|53362|fgenesh1_pm.1_#_405	92.5	77.6
jgi|Aspve1|49262|fgenesh1_pm.1_#_1170	91.4	77.0
jgi|Aspgl1|28588|fgenesh1_pg.21_#_105	83.9	74.8
jgi|Aspwe1|40392|fgenesh1_kg.4_#_684_#_Locus1608v1rpkm116.27	86.1	79.7
jgi|Aspcl1|7255|7000001156880179	85.0	79.8
jgi|Aspfu1|1150|Afu1g12850_mRNA	86.2	79.1
jgi|Neofi1|2646|7000001156973616	86.2	79.3
jgi|Aspac1|38369|Genemark1.545_g	83.2	76.8
jgi|Aspca3|159116|estExt_Genewise1Plus.C_11082	86.0	78.3
jgi|Aspbr1|41323|fgenesh1_kg.4_#_627_#_Locus1876v1rpkm111.71	85.0	75.3
jgi|Aspni_DSM_1|161829|An08g05670m.01	84.6	77.8
jgi|Asptu1|112586|e_gw1.1.743.1	84.4	78.0
jgi|Aspfo1|130828|e_gw1.4.716.1	84.6	78.2
jgi|Aspka1_1|18106|AKAW_06633	84.6	78.2
jgi|Aspte1|476|ATET_00476	83.9	78.6
jgi|Aspor1|6220|AO090012000623_mRNA	85.8	79.2
jgi|Aspfl1|27006|7000001155834032	85.8	79.6
