# Supplementary material for: Comparative genomics reveals high biological diversity and specific adaptations in the industrially and medically important fungal genus Aspergillus
Source: Genome Biol. 2017 Feb 14;18:28. doi: 10.1186/s13059-017-1151-0 (PMC5307856; doi:10.1186/s13059-017-1151-0)
Supplement: Additional file 26: — Maximum likelihood rooted tree of Fur-like transporters of the aspergilli and some other members of the Eurotiales (Penicillium, Talaromyces). (PDF 537 kb) [file 13059_2017_1151_MOESM26_ESM.pdf]

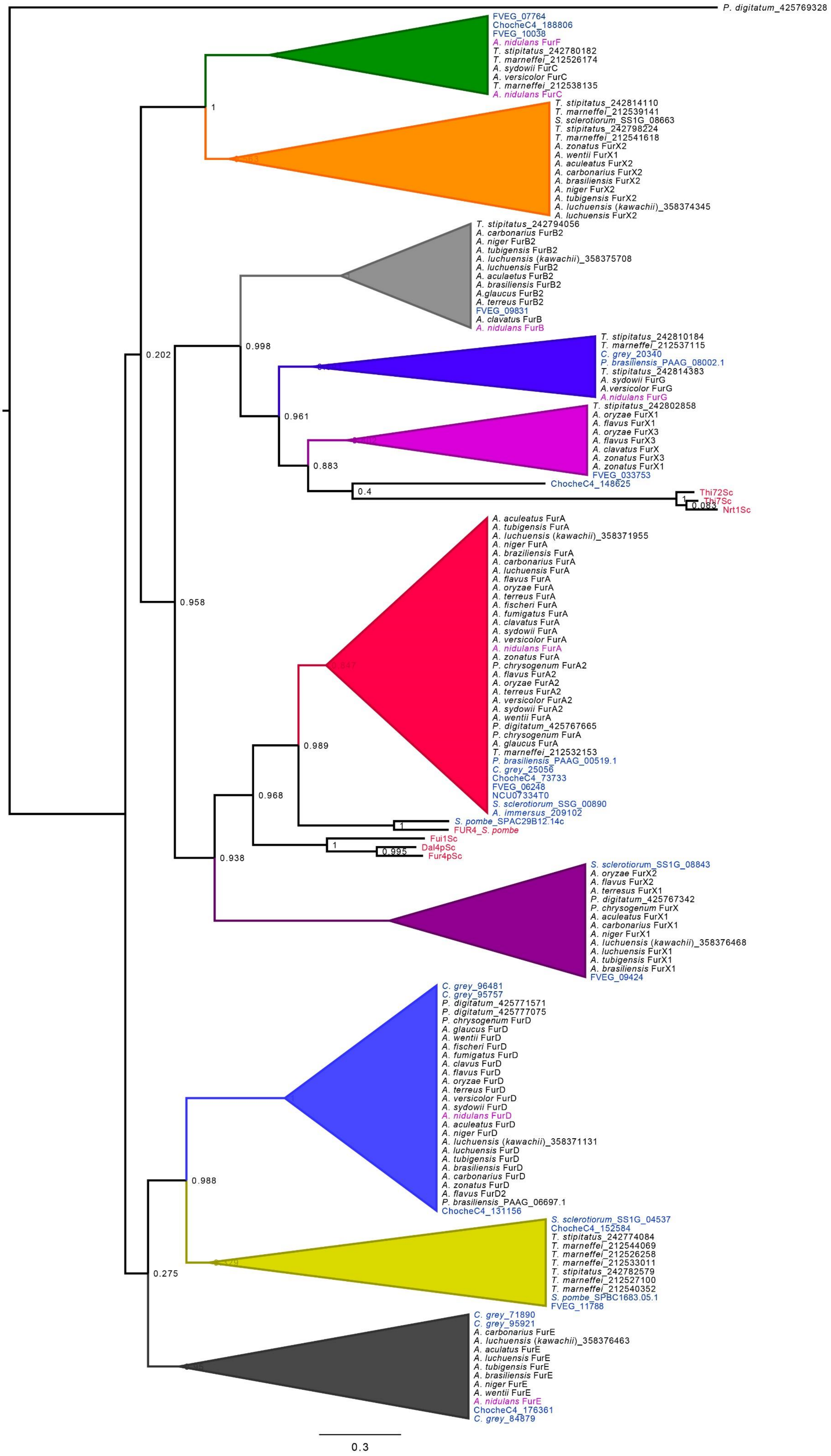

P. digitatum_425769328
FVEG_07764
ChocheC4_188806
FVEG_10038
A. nidulans FurF
T. stipitatus_242780182
T. marneffei_212526174
A. sydowii FurC
A. versicolor FurC
T. marneffei_212538135
A. nidulans FurC
1
T. stipitatus_242814110
T. marneffei_212539141
S. sclerotiorum_SS1G_08663
T. stipitatus_242798224
T. marneffei_212541618
A. zonatus FurX2
A. wentii FurX1
A. aculeatus FurX2
A. carbonarius FurX2
A. brasiliensis FurX2
A. niger FurX2
A. tubigensis FurX2
A. luchuensis (kawachii)_358374345
A. luchuensis FurX2
T. stipitatus_242794056
A. carbonarius FurB2
A. niger FurB2
A. tubigensis FurB2
A. luchuensis (kawachii)_358375708
A. luchuensis FurB2
A. aculaetus FurB2
A. brasiliensis FurB2
A.glaucus FurB2
A. terreus FurB2
FVEG_09831
A. clavatus FurB
A. nidulans FurB
0.202
0.998
T. stipitatus_242810184
T. marneffei_212537115
C. grey_20340
P. brasiliensis_PAAG_08002.1
T. stipitatus_242814383
A. sydowii FurG
A.versicolor FurG
A.nidulans FurG
0.961
T. stipitatus_242802858
A. oryzae FurX1
A. flavus FurX1
A. oryzae FurX3
A. flavus FurX3
A. clavatus FurX
A. zonatus FurX3
A. zonatus FurX1
FVEG_033753
0.883
ChocheC4_148625
0.4
Thi72Sc
Thi7Sc
0.083
Nrt1Sc
A. aculeatus FurA
A. tubigensis FurA
A. luchuensis (kawachii)_358371955
A. niger FurA
A. braziliensis FurA
A. carbonarius FurA
A. luchuensis FurA
A. flavus FurA
A. oryzae FurA
A. terreus FurA
A. fischeri FurA
A. fumigatus FurA
A. clavatus FurA
A. sydowii FurA
A. versicolor FurA
A. nidulans FurA
A. zonatus FurA
P. chrysogenum FurA2
A. flavus FurA2
A. oryzae FurA2
A. terreus FurA2
A. versicolor FurA2
A. sydowii FurA2
A. wentii FurA
P. digitatum_425767665
P. chrysogenum FurA
A. glaucus FurA
T. marneffei_212532153
P. brasiliensis_PAAG_00519.1
C. grey_25056
ChocheC4_73733
FVEG_06248
NCU07334T0
S. sclerotiorum_SSG_00890
A. immersus_209102
0.958
0.989
0.968
S. pombe_SPAC29B12.14c
1
FUR4_S. pombe
Fui1Sc
1
Dal4pSc
0.995
Fur4pSc
0.938
S. sclerotiorum_SS1G_08843
A. oryzae FurX2
A. flavus FurX2
A. terresus FurX1
P. digitatum_425767342
P. chrysogenum FurX
A. aculeatus FurX1
A. carbonarius FurX1
A. niger FurX1
A. luchuensis (kawachii)_358376468
A. luchuensis FurX1
A. tubigensis FurX1
A. brasiliensis FurX1
FVEG_09424
C. grey_96481
C. grey_95757
P. digitatum_425771571
P. digitatum_425777075
P. chrysogenum FurD
A. glaucus FurD
A. wentii FurD
A. fischeri FurD
A. fumigatus FurD
A. clavus FurD
A. flavus FurD
A. oryzae FurD
A. terreus FurD
A. versicolor FurD
A. sydowii FurD
A. nidulans FurD
A. aculeatus FurD
A. niger FurD
A. luchuensis (kawachii)_358371131
A. luchuensis FurD
A. tubigensis FurD
A. brasiliensis FurD
A. carbonarius FurD
A. zonatus FurD
A. flavus FurD2
P. brasiliensis_PAAG_06697.1
ChocheC4_131156
0.988
S. sclerotiorum_SS1G_04537
ChocheC4_152584
T. stipitatus_242774084
T. marneffei_212544069
T. marneffei_212526258
T. marneffei_212533011
T. stipitatus_242782579
T. marneffei_212527100
T. marneffei_212540352
S. pombe_SPBC1683.05.1
FVEG_11788
0.275
C. grey_71890
C. grey_95921
A. carbonarius FurE
A. luchuensis (kawachii)_358376463
A. aculatus FurE
A. luchuensis FurE
A. tubigensis FurE
A. brasiliensis FurE
A. niger FurE
A. wentii FurE
A. nidulans FurE
ChocheC4_176361
C. grey_84879
0.3

Additional File 26. Maximum likelihood rooted tree of Fur-like transporters of the aspergilli and some other members of the *Eurotiales* (*Penicillium*, *Talaromyces*). Some sequences from outside these groups included are in blue color lettering. The tree is shown in a cartoon form, built around the characterized paralogues of *A. nidulans* [1, 2]. Orthologs of each of the *A. nidulans* paralogs (in purple) are characterized by identity ≥ 60% and synteny. These are called with the same letter as the paralogs of *A. nidulans*, followed by a number in the few cases where identity is lower (e.g. FurB2 are proteins present in several aspergilli showing 55-58% identity with the FurB of *A. nidulans* or *A. clavatus*). Homologs with lower identities (in the range 25-45%) are named with an X followed when necessary with a number. Characterized transporters of *S. cerevisiae* and *S. pombe* are shown by their gene names in red lettering. Other proteins are indicated by the accession number in JGI Mycosm following the species names. These are: *Eurotiomycetes*, *Onigenales*, *Paracoccidioides brasiliensis*. *Sordariomycetes*, *Fusarium verticilloides* (*Hypocreomycetidae*, FGVE) and the only homolog present in *N. crassa* (*Sordariales*, NCU). *Leotiomycetes*, *Sclerotina sclerotium*. *Dothideomycetes*, *Cochliobolus heterostrophus*. *Lecanoomycetes*, *Xanthoria parietina*. *Pezizomycetes*, *Ascobolus immersus*. *P. digitatum*_425769328 is in fact a FCY transporter and constitutes a suitable outgroup. It is also part of Fig. 13 as a putative FycA ortholog. Values at the nodes are aLTRs (approximate likelihood ratio test) [3].

1. Hamari Z, Amillis S, Drevet C, Apostolaki A, Vagvolgyi C, Diallinas G, Scazzocchio C: **Convergent evolution and orphan genes in the Fur4p-like family and characterization of a general nucleoside transporter in *Aspergillus nidulans*.** *Mol Microbiol* 2009, **73:**43-57.
2. Krypotou E, Evangelidis T, Bobonis J, Pittis AA, Gabaldon T, Scazzocchio C, Mikros E, Diallinas G: **Origin, diversification and substrate specificity in the family of NCS1/FUR transporters.** *Mol Microbiol* 2015, **96:**927-950.
3. Anisimova M, Gascuel O: **Approximate likelihood-ratio test for branches: A fast, accurate, and powerful alternative.** *Syst Biol* 2006, **55:**539-552.
