# Supplementary material for: Comparative genomics reveals high biological diversity and specific adaptations in the industrially and medically important fungal genus Aspergillus
Source: Genome Biol. 2017 Feb 14;18:28. doi: 10.1186/s13059-017-1151-0 (PMC5307856; doi:10.1186/s13059-017-1151-0)
Supplement: Additional file 27: — Maximum likelihood rooted phylogeny of the FCY-like transporters of the aspergilli and some penicillia. (PDF 324 kb) [file 13059_2017_1151_MOESM27_ESM.pdf]

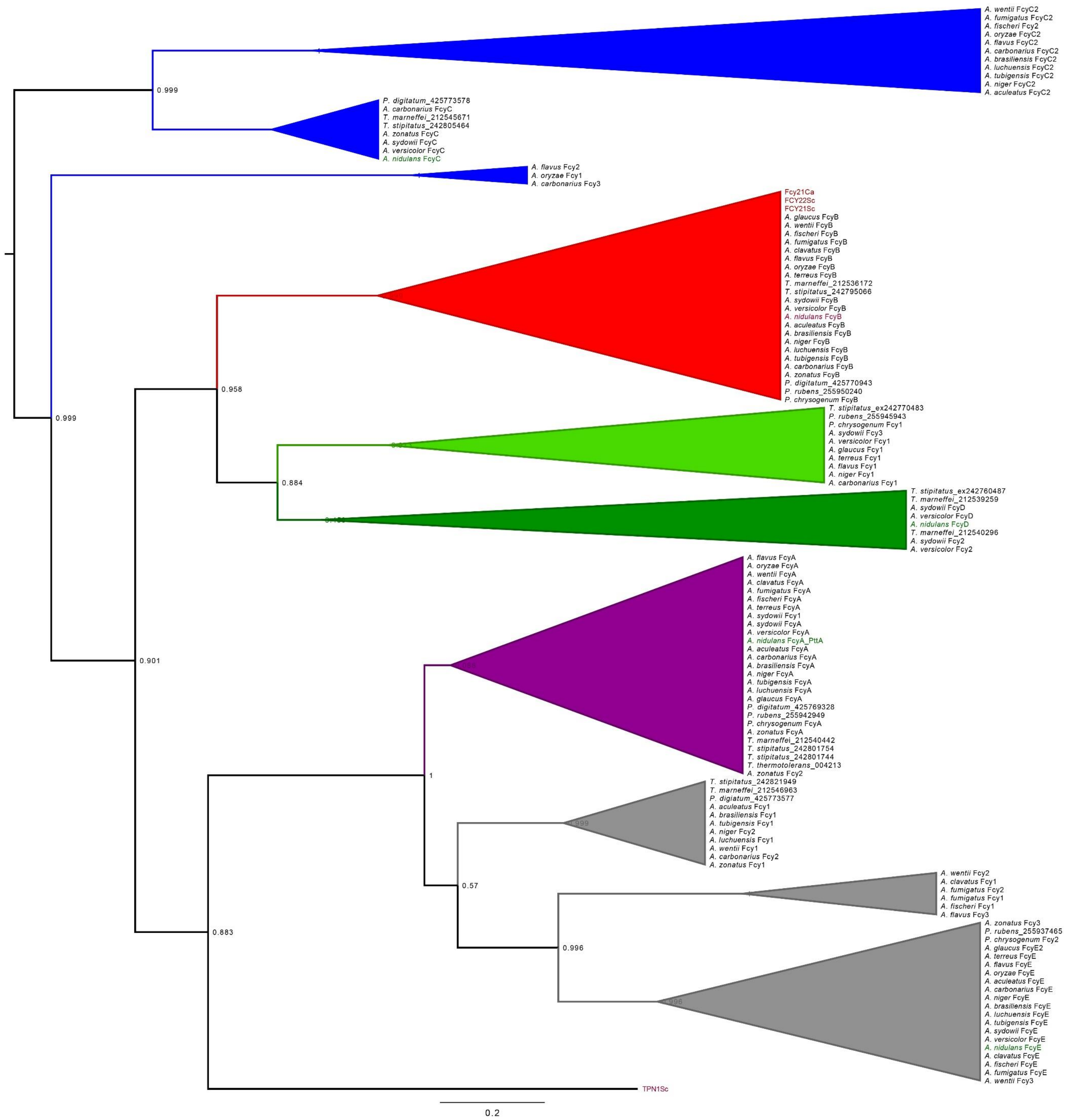

Additional File 27. Maximum likelihood rooted phylogeny of the FCY-like transporters of the aspergilli and some penicillia. The tree, shown in cartoon form, shows the distribution of orthologs of the different *A. nidulans* FCY paralogues [1]. The orthologs within the aspergilli, showing an amino acid identity >60% and synteny, are called accordingly with the degree of identity with the different *A. nidulans* paralogs shown in green lettering (e.g. all aspergilli have FcyB transporters). Homologs sharing ~50-60% identity with an *A. nidulans* protein are named with the same letter followed by a number (e.g. FcyE2 are proteins sharing 55-58% identity with FcyE of *A. nidulans*). Homologs with lower identities (in the range 25-45%) are named with a number (e.g. Fcy1, Fcy2, Fcy3 in *A. wentii*). Sequences of uncharacterized proteins from other organisms (*Penicillium* and *Talaromyces*) are shown with the name of the organism plus the accession number in the JGI MycoCosm database. The paralogues of *S. cerevisiae* are included, denoted with the specific gene name. Characterized transporters (including *A. nidulans* FcyB) [2] are shown in red lettering. Values at nodes are aLTRs (approximate likelihood ratio test) [3].

1. Krypotou E, Scazzocchio C, Diallinas G: **Functional characterization of NAT/NCS2 proteins of *Aspergillus brasiliensis* reveals a genuine xanthine-uric acid transporter and an intrinsically misfolded polypeptide.** *Fungal Genet Biol* 2015, **75:**56-63.
2. Vlanti A, Diallinas G: **The *Aspergillus nidulans* FcyB cytosine-purine scavenger is highly expressed during germination and in reproductive compartments and is downregulated by endocytosis.** *Mol Microbiol* 2008, **68:**959-977.
3. Anisimova M, Gascuel O: **Approximate likelihood-ratio test for branches: A fast, accurate, and powerful alternative.** *Syst Biol* 2006, **55:**539-552.
