# Supplementary material for: Comparative genomics reveals high biological diversity and specific adaptations in the industrially and medically important fungal genus Aspergillus
Source: Genome Biol. 2017 Feb 14;18:28. doi: 10.1186/s13059-017-1151-0 (PMC5307856; doi:10.1186/s13059-017-1151-0)
Supplement: Additional file 28: — Number of flavohemoglobins and protein IDs found in the genome sequences of the Aspergillus species. (PDF 79 kb) [file 13059_2017_1151_MOESM28_ESM.pdf]

Additional File 28. Number of flavohemoglobins and protein IDs found in the genome sequences of the *Aspergillus* species.

| Name | Strain | # of genes | % Growth Inhibition | Locus tag[1] | Flavo type | Length | Predicted localization |
|---|---|---|---|---|---|---|---|
| *A. glaucus* | CBS 516.65 | 1 | 17 | Aspgl1_0031833 | *fhbA* | 415 aa | cytosol; nucleus |
| *A. tubingensis* | CBS 134.48 | 2 | 40 | Asptu1_0052006 | *fhbA* | 456 aa | cytosol; nucleus |
| | | | | Asptu1_0195204 | *fhbA* | 417 aa | cytosol; nucleus |
| *A. zonatus* | CBS 506.65 | 2 | 82 | Aspzo1_0148118 | *fhbA* | 411 aa | cytosol; nucleus |
| | | | | Aspzo1_0056900 | *fhbB* | 429 aa | cytosol; nucleus |
| *A. brasiliensis* | CBS 101740 | 2 | 79 | Aspbr1_0049986 | *fhbA* | 417 aa | cytosol; nucleus |
| | | | | Aspbr1_0047169 | *fhbA* | 455 aa | cytosol; nucleus |
| *A. versicolor* | CBS 795.97 | 2 | 74 | Aspve1_0140313 | *fhbA* | 412 aa | cytosol; nucleus |
| | | | | Aspve1_0056118 | *fhbB* | 424 aa | cytosol; nucleus |
| *A. sydowii* | CBS 593.65 | 2 | 58 | Aspsy1_0092771 | *fhbA* | 413 aa | cytosol; nucleus |
| | | | | Aspsy1_0158001 | *fhbB* | 426 aa | cytosol; nucleus |
| *A. oryzae* | Rib40 | 2 | | AO090011000202 | *fhbA* | 415 aa | cytosol; mitochondrial; nucleus |
| | | | | AO090012000171 | *fhbB* | 436 aa | mitochondrion |
| *A. niger* | ATCC1015 | 2 | 11 | 201503 | *fhbA* | 417 aa | cytosol |
| | | | | 54097 | *fhbA* | 456 aa | cytosol; nucleus |
| *A. luchuensis* | CBS 106.47 | 2 | 15 | e_gw1.9.1180.1<br>estExt_Genewise1.C_10_t10326 | *fhbA*<br>*fhbA* | 417 aa<br>456 aa | cytosol; nucleus<br>extracellular, cytosol;mitochondrion |
| *A. niger* | NRRL3 | 2 | -21 | NRRL3_00799<br>NRRL3_10307 | *fhbA*<br>*fhbA* | 417 aa<br>456 aa | cytosol; nucleus<br>extracellular, cytosol;mitochondrion |
| *A. wentii* | DTO 134-E9 | 1 | 85 | Aspwe1_0174689 | *fhbA* | 415 aa | cytosol; nucleus |
| *A. niger* | CBS 513.88 | 2 | 72 | An14g02460 | *fhbA* | 417 aa | cytosol |
| | | | | An18g01780 | *fhbA* | 336 aa | cytosol; nucleus |

| | | | | | | | |
|---|---|---|---|---|---|---|---|
| *A. fischeri* | CBS 544.65 | 1 | 72 | NFIA_030070 | *fhbA* | 413 aa | cytosol; nucleus |
| *A. terreus* | NCCB IH2624 | 2 | 81 | ATEG_02147 | *fhbA* | 415 aa | cytosol; nucleus |
| | | | | ATEG_07899 | *fhbB* | 427 aa | cytosol; nucleus |
| *A. flavus* | NRRL 3357 | 2 | 62 | AFL2G_04980 | *fhbA* | 416 aa | cytosol; nucleus |
| | (CBS 128202) | | | AFL2G_03098 | *fhbB* | 431 aa | cytosol; nucleus |
| *A. fumigatus* | Af 293 | 2 | 73 | Afu4g03410 | *fhbA* | 412 aa | cytosol |
| | | | | Afu8g06080 | *fhbB* | 432 aa | cytosol; mitochondrial; nucleus |
| *A. clavatus* | NRRL1 | 2 | 94 | ACLA_055100 | *fhbA* | 409 aa | cytosol; nucleus |
| | (CBS 513.65) | | | ACLA_018040 | *fhbA* | 458 aa | cytosol; nucleus |
| *A. nidulans* | FGCS A4 | 2 | 74 | AN7169 | *fhbA* | 410 aa | cytosol; nucleus |
| | | | | AN3522 | *fhbB* | 426 aa | cytosol; mitochondrial; nucleus |
| *A. carbonarius* | DTO 115-B6 | 1 | 45 | Acar5010_400189 | *fhbA* | 417 aa | cytosol; nucleus |
| *A. aculeatus* | CBS 172.66 | 2 | 73 | Aacu16872_050856 | *fhbA* | 418 aa | cytosol; nucleus |
| | | | | Aacu16872_063347 | *fhbB* | 430 aa | cytosol; nucleus |

[1] Proteins IDs correspond to: Broad Institute - *Aspergillus* Comparative Database (*A. fischeri, A. oryzae, A. fumigatus, A. terreus, A. clavatus, A. nidulans, A. flavus*) and JGI (*A. aculeatus, A. carbonarius, A. brasiliensis, A. glaucus, A. tubingensis, A. wentii, A. zonatus, A. versicolor, A. sydowii*).
