# Supplementary material for: Comparative genomics reveals high biological diversity and specific adaptations in the industrially and medically important fungal genus Aspergillus
Source: Genome Biol. 2017 Feb 14;18:28. doi: 10.1186/s13059-017-1151-0 (PMC5307856; doi:10.1186/s13059-017-1151-0)
Supplement: Additional file 33: — Kinases presented in at least one yeast species and with no orthologous genes in other eurotiomycetes. (PDF 117 kb) [file 13059_2017_1151_MOESM33_ESM.pdf]

Additional File 33. Kinases presented in at least one yeast species and with no orthologous genes in other eurotiomycetes. Yeast species analyzed: *P. pastoris* (Picpa1), *S. cerevisiae* (Sacce1, SacceM3707_1) and/or *S. pombe* (Schpo1). Systematic name and description of kinase function refer to *S. cerevisiae* Sacce1.

Cluster	Picpa1	Sacce1	Sacce M3707_1	Schpo1	Systematic Name	Description
FungiJGICTBE22669	0	1	1	0	YPL026C	Suppressor Kinase of SNF3
FungiJGICTBE23938	0	1	1	0	YDR466W	Pkb-activating Kinase Homolog
FungiJGICTBE23984	0	1	1	0	YIL095W	p53 Regulatory Kinase
FungiJGICTBE23997	0	1	1	0	YIL035C	Casein Kinase Alpha subunit
FungiJGICTBE24081	0	1	1	0	YER123W	Yeast Casein Kinase
FungiJGICTBE24161	0	1	1	0	YGL158W	Radiation sensitivity Complementing Kinase
FungiJGICTBE24216	0	1	1	0	YGR052W	Found in Mitochondrial Proteome
FungiJGICTBE24312	0	1	1	0	YHR082C	Kinase Suppressing Prp20-10
FungiJGICTBE24315	0	1	1	0	YBR059C	Ark family Kinase-Like protein
FungiJGICTBE24364	0	1	1	0	YJL165C	HALotolerance
FungiJGICTBE24442	0	1	1	0	YKL168C	
FungiJGICTBE24445	0	1	1	0	YKL161C	Kinase Dead X-talker
FungiJGICTBE24459	0	1	1	0	YKL101W	Histone Synthetic Lethal
FungiJGICTBE24480	0	1	1	0	YKL048C	ELongated Morphology
FungiJGICTBE24796	0	1	1	0	YMR291W	Topoisomerase I Damage Affected
FungiJGICTBE24919	0	1	1	0	YOL128C	Yeast homolog of Glycogen synthase Kinase
FungiJGICTBE24928	0	1	1	0	YOL100W	Pkb-activating Kinase Homolog
FungiJGICTBE25222	0	1	1	0	YDL214C	Pheromone Response Regulator

FungiJGICTBE25245	0	1	1	0	YAR019C	Cell Division Cycle
FungiJGICTBE25246	0	1	1	0	YDL079C	Mds1p Related Kinase
FungiJGICTBE12570	1	2	2	0	YOR233W, YPL141C	KINase, Fatty acyl-CoA synthetase and RNA processing-associated Kinase
FungiJGICTBE12571	1	2	2	0	YDR507C, YCL024W	Growth Inhibitory
FungiJGICTBE13605	0	2	2	0	YJR059W, YKL198C	Putative serine/Threonine protein Kinase
FungiJGICTBE14077	1	1	1	1	YMR001C	Cell Division Cycle
FungiJGICTBE15775	1	1	1	0	YPR106W	Inhibition of Staurosporine Resistance
FungiJGICTBE16796	1	1	1	0	YDL025C	Ribosome biogenesis and TRNA synthetase-associated Kinase
FungiJGICTBE17172	1	1	1	0	YFL029C	Cdk-Activating Kinase
FungiJGICTBE17365	1	1	1	0	YKL171W	Nitrogen Network Kinase
FungiJGICTBE17408	1	1	1	0	YPR054W	
FungiJGICTBE17449	1	1	1	0	YHR102W	Kinase that Interacts with Cdc31p
FungiJGICTBE17505	1	1	1	0	YNL307C	Meiosis and Centromere regulatory Kinase
