# Supplementary material for: Comparative genomics reveals high biological diversity and specific adaptations in the industrially and medically important fungal genus Aspergillus
Source: Genome Biol. 2017 Feb 14;18:28. doi: 10.1186/s13059-017-1151-0 (PMC5307856; doi:10.1186/s13059-017-1151-0)
Supplement: Additional file 23: — Maximum likelihood tree of YAT transporters in the aspergilli. (PDF 89 kb) [file 13059_2017_1151_MOESM23_ESM.pdf]

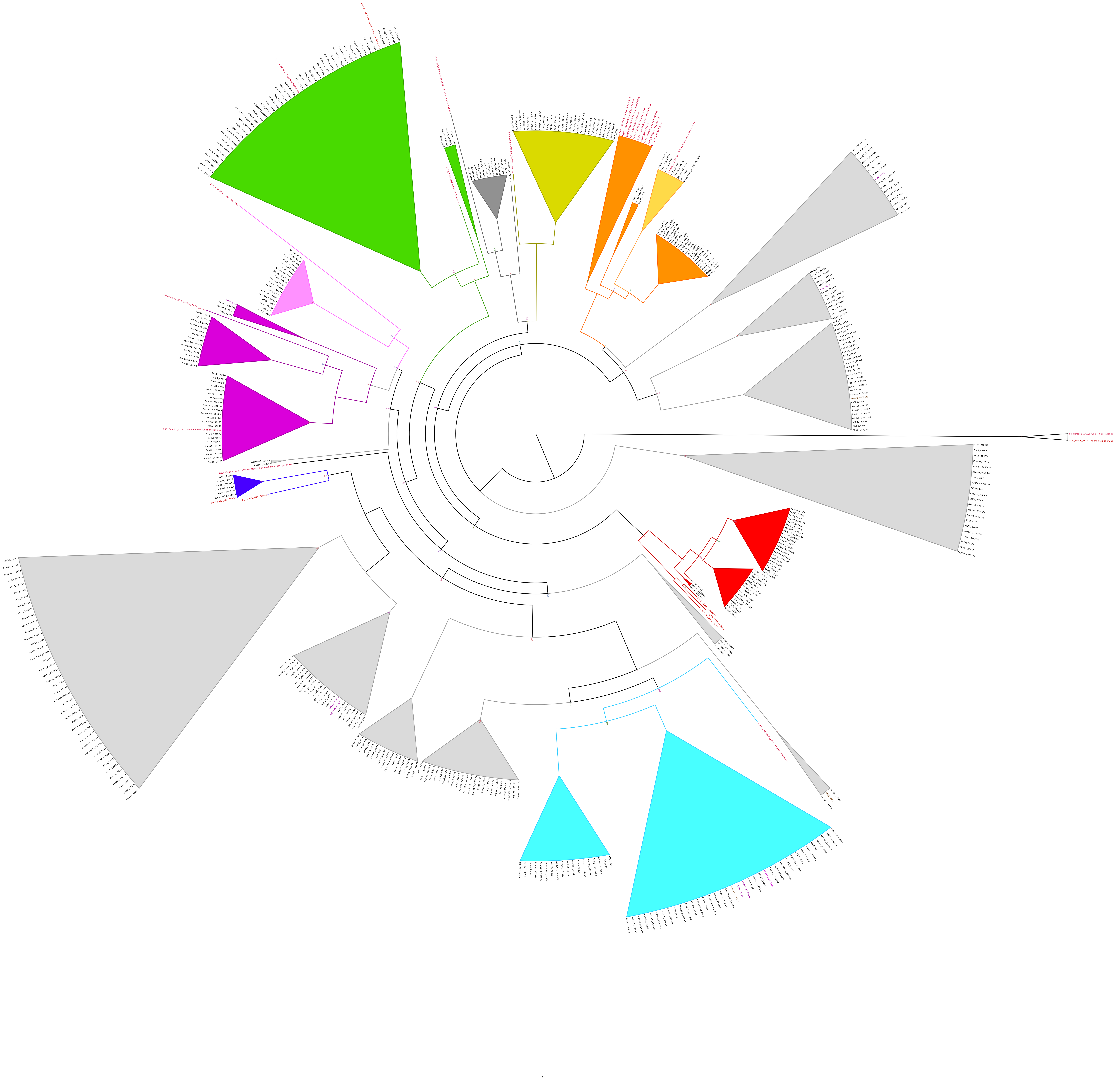

Additional File 23. Maximum likelihood tree of YAT transporters in the aspergilli.

All transporters of the YAT family of the available *Aspergillus* genomes are included. When manual annotation was carried out the corrected sequences were used for this phylogeny. The tree is presented in a simplified cartoon form with collapsed branches. Different colours distinguish putative functional groups, where a functionally characterised transporter of an organism not belonging to the Eurotiales (shown in red, usually a YAT from *S. cerevisiae*) defines the group, with the collapsed branch denoted by the same colour as the out group reference sequence.

Physiologically characterised transporters are shown in red lettering. Proteins reconstructed from pseudogenes, featuring chain termination codons and/or frameshifts, are indicated in purple lettering while genes coding for putative membrane proteins lacking consensus YAT sequences (180, 183) within the open reading frame are shown in brown lettering. Characterised *S. cerevisiae* transporters are indicated by the gene abbreviation followed by its systematic locus identifier starting with "Y". Homologs in the Trichocomaceae are indicated as follows: *A. luchuensis* (Aspfo1), A*. aculeatus* (Aacu16872), *A. brasiliensis* (Aspbr1), *A. carbonarius* (Acar5010), *A. clavatus* (ACLA), *A. flavus* (AFL2G), *A. fumigatus* A1163 (AFUB), *A. fumigatus* Af293 (Afu), *A. glaucus* (Aspgl1), *A. nidulans* (ANID), *A. niger* (An), *A. oryzae* (AO), *A. sydowii* (Aspsy1), *A. terreus* (ATEG), *A. tubingensis* (Asptu1), *A. versicolor* (Aspve1), *A. wentii* (Aspwe1), *A. zonatus* (Aspzo1), *A. fischeri* (NFIA), *P. chrysogenum* (Pench1), and *E. rubrum* (Eurhe1).

Color codes: light grey = clades which do not relate to any physiologically characterized transporters, red = basic amino acid transporters, green = dicarboxylic amino acid transporters, olive green = putative cystine transporters, dark grey = putative branched amino acid transporters, dark purple = putative aromatic amino acid transporters, light purple = a group of membrane proteins similar to the *S. cerevisiæ* amino acid sensor SSY1, dark blue = proline transporters, cyan = related to the *S. cerevisiae* regulator of polyamine transport (AGP1), orange = transporters related to a functionally diverse group of structurally related *S. cerevisiae* transporters. Within this latter group: light orange = proteins related to a characterized general amino acid transporter of *N. crassa*. Empty triangle: two characterised AAAP transporters that appear as an outgroup to the YATs. Values at the nodes indicate aLTRs (approximate likelihood test ratios) values. Colours at nodes indicate the significance of the grouping, from red to purple to blue to green to grey, in order of decreasing significance.
