# Supplementary material for: Comparative genomics reveals high biological diversity and specific adaptations in the industrially and medically important fungal genus Aspergillus
Source: Genome Biol. 2017 Feb 14;18:28. doi: 10.1186/s13059-017-1151-0 (PMC5307856; doi:10.1186/s13059-017-1151-0)
Supplement: Additional file 35: — Maximal likelihood phylogeny of MASC1/RID1 orthologs. (PDF 115 kb) [file 13059_2017_1151_MOESM35_ESM.pdf]

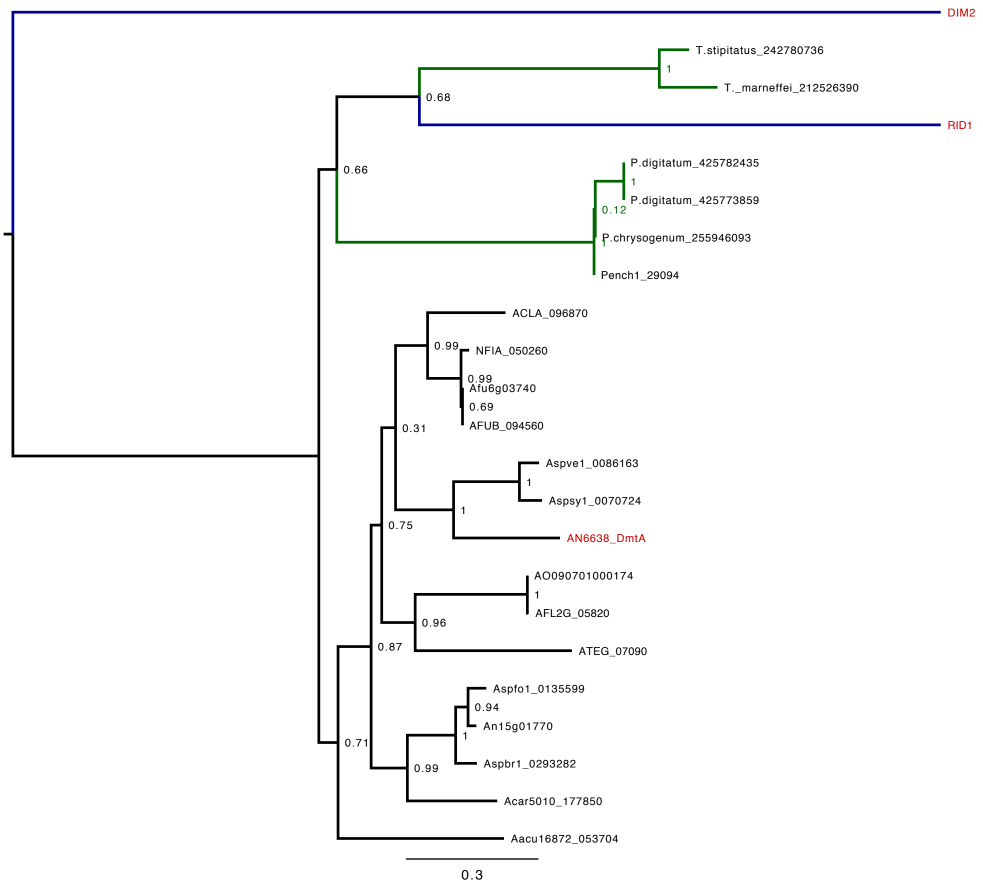

Additional.File.35.Maximum likelihood phylogeny of MASC1/RID1 orthologs.

Alignment carried out with MAFFT (G-ins-i), curation with BMGE with default settings, version-1, phylogeny with PhyML. Digits at the nodes are aLTR (approximate likelihood ratio test values). Names in red, proteins encoded by experimentally characterised genes. Blue branches, *N. crassa*, green branches *Penicillium* and *Talaromyces*, black branches *Aspergillus*. Using different curation algorithms gave essentially the same topology.
