# Supplementary material for: Comparative genomics reveals high biological diversity and specific adaptations in the industrially and medically important fungal genus Aspergillus
Source: Genome Biol. 2017 Feb 14;18:28. doi: 10.1186/s13059-017-1151-0 (PMC5307856; doi:10.1186/s13059-017-1151-0)
Supplement: Additional file 39: — Phylogeny of the Methallophos domain in A. nidulans. (PDF 96 kb) [file 13059_2017_1151_MOESM39_ESM.pdf]

Additional File 39.Phylogeny of the Methallophos domain in *A. nidulans*

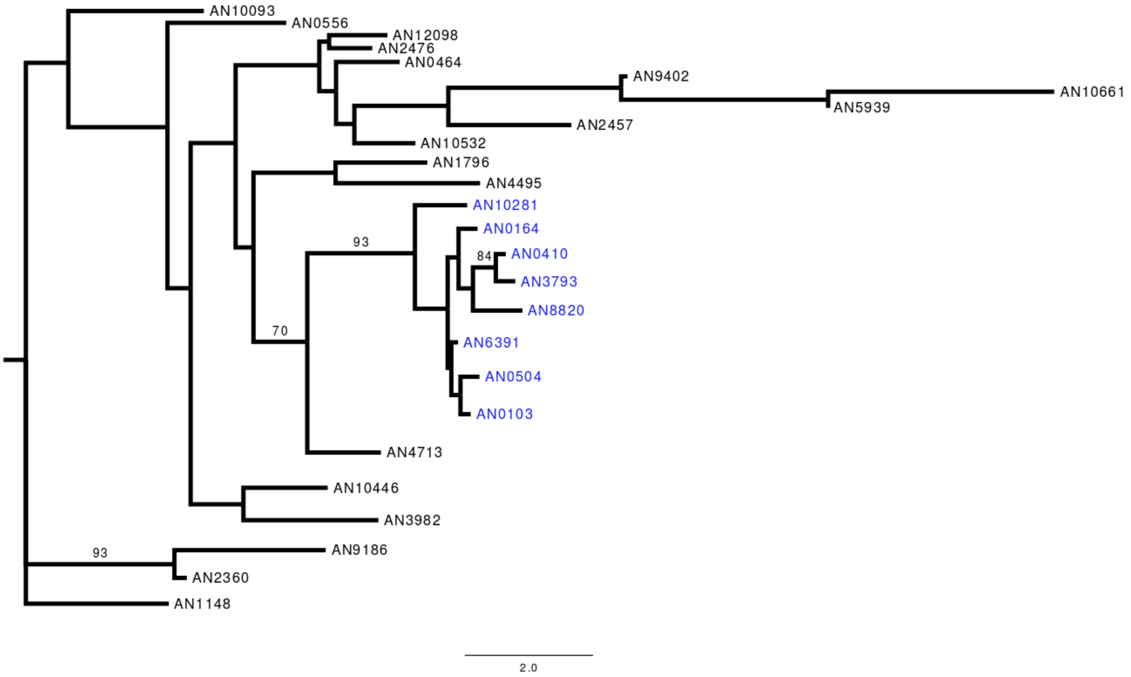

The evolutionary model was estimated using ProtTest (http://darwin.uvigo.es/software/prottest3/). Phylogeny was inferred using RAxML (http://www.exelixis-lab.org/) under the model BLOSUM62 + Γ + I + F and 500 bootstrap replicates. Sequences in blue are PPPs from Son and Osmani [1]. The PPPs form a clear clade in the tree with a bootstrap support of 93% (values of 50% or greater are shown). This group was used to create a PPP-specific HMM.

1. Son S, Osmani SA: **Analysis of all protein phosphatase genes in *Aspergillus nidulans* identifies a new mitotic regulator, *fcp1*.** *Eukaryot Cell* 2009, **8:**573-585.
