# Supplementary material for: Comparative genomics reveals high biological diversity and specific adaptations in the industrially and medically important fungal genus Aspergillus
Source: Genome Biol. 2017 Feb 14;18:28. doi: 10.1186/s13059-017-1151-0 (PMC5307856; doi:10.1186/s13059-017-1151-0)
Supplement: Additional file 34: — Histones of the Aspergilli. (PDF 526 kb) [file 13059_2017_1151_MOESM34_ESM.pdf]

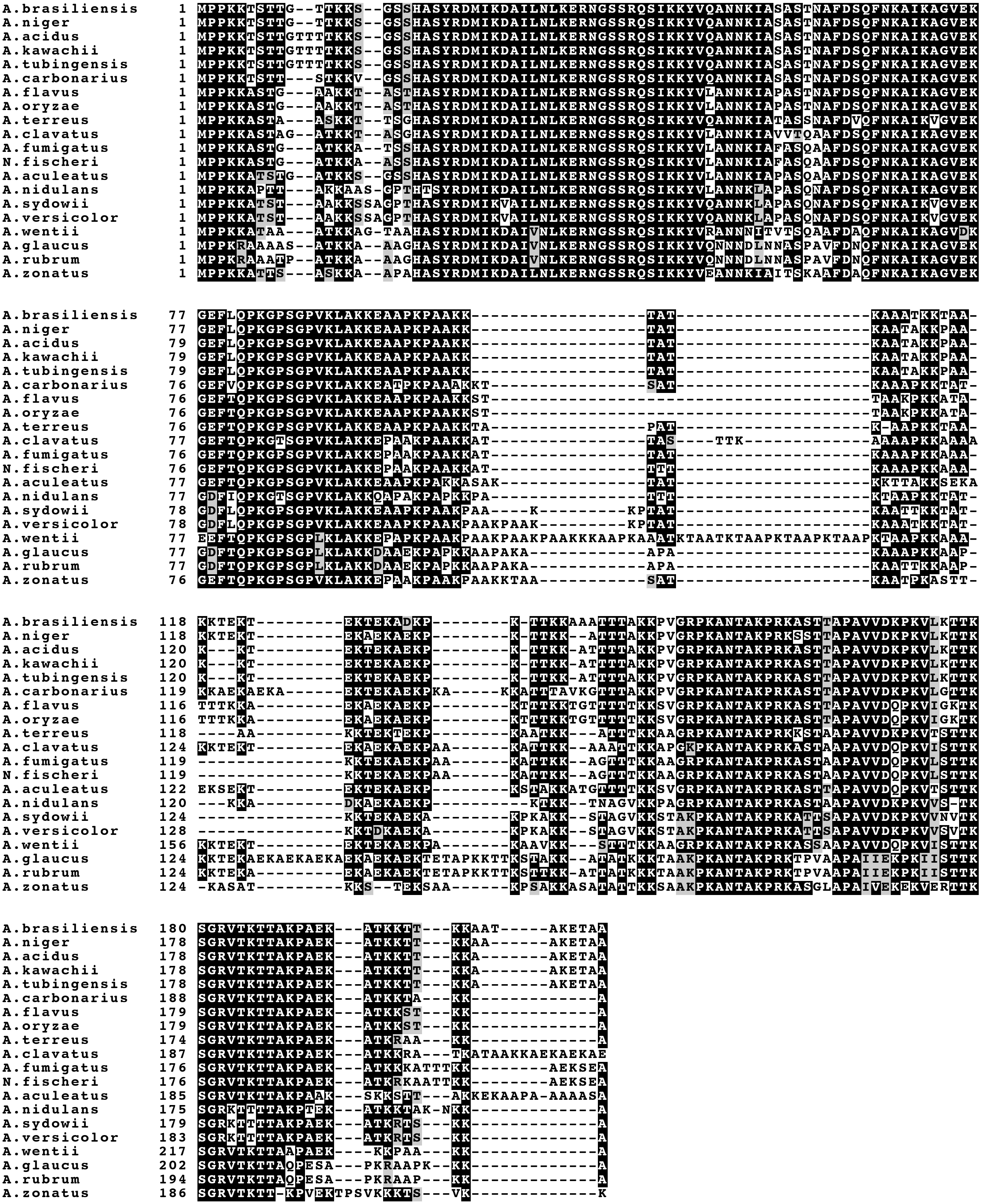

Histone H1 Alignment

```
A.brasiliensis     1 MPPKTG--GRGRKSIVAA-PRQSTAGDRAAASSSKA-----------------AATTSPAAKKV-RK--STGPARGGKRP
A.niger            1 MPPKTG--GRGRKSIVAA-PRQSTAGDRAAASSSKA-----------------AATTSPAAKKV-RK--STGPARGGKRP
A.acidus           1 MPPKTGGRGRGRKSVTAA-PRQSTAGDRAAASSSTA-----------------ATTASPAAKKV-RK--STGATRGGKRP
A.tubingensis      1 MPPKTGGRGRGRKSVTAA-PRQSTAGDRAAASSSTA-----------------ATTASPAAKKV-RK--STGATRGGKRP
A.kawachii         1 MPPKTGGRGRGRKSVTAA-PRQSTAGDRAAASSSTA-----------------AITASPAAKKV-RK--STGATRGGKRP
A.carbonarius      1 MPPKT---GRGRKA-AST-PRKSTTGDRAAASASKA-----------------AATTSPSASKV-HK--TAGSSRGGKRP
A.terreus          1 MPPKT---GRGHKI-TAA-PRTEASSSRTTAAAA-G-----------------PSTASPSAHRV-RK--STGPSRGGKRP
A.clavatus         1 MPPKT---GRGRKA-VSA-GRPRKD----ESGPSDA-----------------PATASPSTRGQ-RK--SSGATRGGKRP
A.flavus           1 MPPKT---GGGRKV-IAA-SRAKAD----GAGKESA-----------------AAKGSPSTRG--RK--SAGGARAGKRP
A.oryzae           1 MPPKT---GGGRKV-IAA-SRAKAD----GAGKESA-----------------AAKGSPSTRG--RK--SAGGARAGKRP
A.fumigatus        1 MPPKT---GRGRKA-IGA-PRTQPR-DDGGAGPSNA-----------------AATASPSTRGQPPK--FRGGTRGGKRP
N.fischeri         1 MPPKT---GRGRKA-IGA-PRTQPR-DDGGAGPSNA-----------------AATASPSTRGRPPK--SRGGTRGAKRP
A.wentii           1 MPPKT---GRGNKI-IAA-PRAKAAKEEAEAGPSRA----------------SPATASPATRGR-RK--SAGASRGGKKP
A.aculeatus        1 MPPKT---GRGRKS-IAG-PRKSTAAAAAAAATTAAATDDGAAAAAGPSTAPATTTTTPAARG--RK--PAGVTR-GKRP
A.zonatus          1 MPPKT---GRGRKM-LAAQARPRPATGD-GGADGDG------AAAAGPS-SRNDGATKPG-RG--GR--KSGGTRGGKRP
A.glaucus          1 MPPKT---GKGRKV-VSE-PRSRPSTGDGEAGPSSK----------------PQQTASPANKRK-PKASTSGEAR-DKRP
A.rubrum           1 MPPKT---GKGRKV-VSE-PRSRPSASDGEAGLSSD----------------LQQTVSPANKKN-PKASTSGEAREGKRP
A.nidulans         1 MPPK------GRKP-SSA-ATAGTGSSKATSAKTTST----TTSGASP--KVTKSSRTLGSKALASA--SVSKSKGSKRP
A.sydowii          1 MPPK------GRKP-ASA-----------TAGPSTTTT---TTTGAGP-------AKSAGVKKG-AK--GVQGTTGGKRP
A.versicolor       1 MPPK------GRKP-ASA-----------TAGPS-------KTPAAGP-------SKSAGVKKG-PK--------GGKRP
A.nidulans_H3      1 MARTK---QTARKS------------------------------------------------------------TGGKAP

A.brasiliensis    58 AG--------KAPRQSDVQ---PGDPTPQG--RVRRYKPGTVALKEIRKYQRSYDLLIQKLPFARLVREVALDLLPSEVG
A.niger           58 AG--------KAPRESDVQILAAGDPTPQG--RVRRYKPGTVALKEIRKYQRSYDLLIQKLPFARLVREVALDLLPSEVG
A.acidus          60 AG--------KAPRESDVQ---PGDPTPQG--RVRRYKPGTVALKEIRKYQRSYDLLIQKLPFARLVREVALDLLPSEVG
A.tubingensis     60 AG--------KAPRESDVQ---PGDPTPQG--RVRRYKPGTVALKEIRKYQRSYDLLIQKLPFARLVREVALDLLPSEVG
A.kawachii        60 AG--------KAPRESDVQ---PGDPTPQG--RVRRYKPGTVALKEIRKYQRSYDLLIQKLPFARLVREVALDLLPSEVG
A.carbonarius     56 AG--------KKPRVSDVQ---PGDPTPQG--RSRRYKPGTVALKEIRKYQRSYDLLISKLPFARLVREVALDLLPSDVG
A.terreus         55 AG---------APRDSDVQ---PGDPTPTG--RRRRYRPGTVALKEIRKYQRSYDLLIQKLPFARLVREVALDILPAERG
A.clavatus        52 AG---------APRKSDVQ---PGDPTPQG--RPRRYRPGTVALKEIRKYQRSYDLLIQKLPFARLVREVALDLLPTEVG
A.flavus          51 AG---------ASRKSDVQ---PGDPTPQG--RHRRYRPGTVALKEIRKYQRSYDLLIRKLPFARLVREVALDLLPADVG
A.oryzae          51 AG---------ASRKSDVQ---PGDPTPQG--RHRRYRPGTVALKEIRKYQRSYDLLIRKLPFARLVREVALDLLPADVG
A.fumigatus       56 AH---------APRPSDVQ---PGDPTPQG--RRHRYKPGTVALKEIRRYQRSYDLLIQKLPFARLVREVALELLPAEVG
N.fischeri        56 AR---------APRPSDVQ---PGDPTPQG--RRHRYKPGTVALKEIRKYQRSYDLLIQKLPFARLVREVALELLPAEVG
A.wentii          57 AGVSK-PK-AKPSKQTDVQ---PGDPTPQG--RRHRYKPGTVALKEIRKYQRSYDLLIQKLPFARLVREVALELLPADVG
A.aculeatus       71 AK---------APRTSNVE---PGDPTPTG--RRRRYKPGTVALKEIRHYQRSFDLLIRKLPFARLVREVALDLLPAEVG
A.zonatus         64 PG---------TSGQSNIQ---PGDPTPKG--RRHRYKPGTVALKEIRKYQRSYDLLLRKLPFARLVREVALELLPANVG
A.glaucus         58 SGVTKTPKAAKTAITSDVQ---PGDPTPQG--RSRRYKPGTVALKEIRKYQRSFDLLIQKLPFARLVREVALELLPADVG
A.rubrum          59 SGVTKPPK---TAKTLDIQ---PGDPTPQG--RPRRYKPGTVALKEIRKYQRSFDLLIQKLPFARLVREVALELLPADVG
A.nidulans        65 PT-------APSDDQSSIQ---PGDPTPKG--RRHRYKPGTVALKEIRKYQRSYDLLLRKLPFARLVREVALDILPADVG
A.sydowii         50 PR--------TSDAQSDVQ---PGDPTPKG--RRHRYKPGTVALKEIRKYQRSYDLLLRKLPFARLVREVALDLLPAEVG
A.versicolor      40 RT---------SDVQPDVQ---PGDPTPKG--RRHRYKPGTVALKEIRKYQRSYDLLLRKLPFARLVREVALDLLPAEVG
A.nidulans_H3     18 RK---------QLASKAAR---KAAPSTGGVKKPHRYKPGTVALREIRRYQKSTELLIRKLPFQRLVREIA-----QDFK

A.brasiliensis   125 AELRWQSHAIQALQEAAEAFLVHLFEDTNLCAIHAKRVTIMQKDIQLARRIRGVWGGLG
A.niger          128 AELRWQSHAIQALQEAAEAFLVHLFEDTNLCAIHAKRVTIMQKDIQLARRIRGVWGGLG
A.acidus         127 AELRWQSHAIQALQEAAEAFLVHLFEDTNLCAIHAKRVTIMQKDIQLARRIRGVWGGLG
A.tubingensis    127 AELRWQSHAIQALQEAAEAFLVHLFEDTNLCAIHAKRVTIMQKDIQLARRIRGVWGGLG
A.kawachii       127 AELRWQSHAIQALQEAAEAFLVHLFEDTNLCAIHAKRVTIMQKDIQLARRIRGVWGGLG
A.carbonarius    123 SELRWQSHAIQALQEAAEAFLVHLFEDTNLCAIHAKRVTIMQKDIQLARRIRGVWGGLG
A.terreus        121 AELRWQSHAIQALQEAAEAFLVHLFEDTNLCALHAKRVTIMQKDIQLARRIRGVWGGLG
A.clavatus       118 AQLRWQSHAIQALQEAAEAFLVHLFEDTNLCAIHAKRVTIMQKDIQLARRIRGAWGGLG
A.flavus         117 SELRWQSQAIMALQEAAEAFLVHLFEDTNLCALHAKRVTIMQKDIQLARRIRGAWGGLG
A.oryzae         117 SELRWQSQAIMALQEAAEAFLVHLFEDTNLCALHAKRVTIMQKDIQLARRIRGAWGGLG
A.fumigatus      122 AELRWQSHAIQALQEAAEAFLVHLFEDTNLCALHAKRVTIMQKDIQLARRIRGIWGGLG
N.fischeri       122 AELRWQSHAIQALQEAAEAFLVHLFEDTNLCALHAKRVTIMQKDIQLARRIRGVWGGLG
A.wentii         130 AELRWQSHAIQALQEAAEAFLVHLFEDTNLCALHAKRVTIMQKDIQLARRIRGAWGGLG
A.aculeatus      137 SELRWQSHAILALQEAAEAFLVHLFEDTNLCAIHAKRVTIMQKDIQLARRIRGVWGGLG
A.zonatus        130 AELRWQSHAIQALQEAAEAFLVHLFEDTNLCALHAKRVTIMQKDIQLARRIRGVWGGLG
A.glaucus        133 EQLRWQSHAIQALQEAAEAFLVHLFEDTNLCALHAKRVTIMQKDIQLARRIRGAWGGLG
A.rubrum         131 EQLRWQSHAIQALQEAAEAFLVHLFEDTNLCALHAKRVTIMQKDIQLARRIRGAWGGLG
A.nidulans       133 SELRWQSHAIQALQEAAEAFLVHLFEDTNLCAIHAKRVTIMQKDIQLARRIRGAWGGLG
A.sydowii        117 AELRWQSHAILALQEAAEAFLVHLFEDTNLCAIHAKRVTIMQKDIQLARRIRGAWGGLG
A.versicolor     106 AELRWQSHAILALQEAAEAFLVHLFEDTNLCAIHAKRVTIMQKDIQLARRIRGAWGGLG
A.nidulans_H3     81 SDLRFQSSAIGALQESVEAYLVSLFEDTNLCAIHAKRVTIQSKDIQLARRLRGE---RS
```

Centromeric Histone H3
Alignment

```
A.acidus            1  MD--PRLVTDRKSKRF-LPKKR-FRKILRNNIDGITRPSIRRLARRGGVIRISADVYPEV
A.kawachii          1  MD--PRLVTDRKSKRF-LPKKR-FRKILRNNIDGITRPSIRRLARRGGVIRISADVYPEV
A.tubingensis       1  MD--PRLVTDRKSKRF-LPKKR-FRKILRNNIDGITRPSIRRLARRGGVIRISADVYPEV
A.niger             1  MD--PRLVTDRRSKRF-LPKKR-LRKLLRNNIDGITRPSIRRLARRGGVIRISADVYPEV
A.niger             1  MD--PRLVTDRRSKRF-LPKKR-LRKLLRNNIDGITRPSIRRLARRGGVIRISADVYPEV
A.carbonarius       1  MD--PKLVTDKRSRRF-LPKKR-YRKVLRNNIDGITRPTIRRLARRGGVIRISAGIYAEV
A.aculeatus         1  MD--LNLLKESGKHRAFMPRRR-FRKLARDNIQGVTRPAIRRLARRGGVYRIGNEIYEEA
A.nidulans          1  MPSLISVPQSTGLHGLATLQSR-RHKKLRESIRGVTRPAIRRLARRGGVYRIKNEIYDEI
A.wentii            1  MA-----PRLQ------NIFPK-RHRKLRDNIAGITRPAIRRLARRGGVMRIKKEIYDEI
A.versicolor        1  MAR-------VAVHILPLAGYR-RHKVLRDNILGVTRPAIRRLARRGGVYRIKKEIYDEI
A.sydowii           1  MAR-------VATCVLPMSRYK-RHRILRDNILGITRPAIRRLARRGGVYRIKKEIYEEI
A.fumigatus_A11     1  MDV-AHLAANPNTQGL-LLKGHRRRRLLRDNVNGITKPAIRRLARRGGVVRMKTDIYAQI
A.fumigatus_Af2     1  MDV-AHLAANPNTQGL-LLKGHRRRRLLRDNVNGITKPAIRRLARRGGVVRMKTDIYAQI
N.fischeri          1  MDV-THLAANPNTRGL-LLKGH-RRRFLRDNIMGITKPAIRRLARRAGVVRIKTDIYAEI
A.clavatus          1  MD--PNSPQNRSTRGL-LLKSH-RRRHLRDNLLGITRPAIRRLARRGGVMRIKSDIYNEA
A.flavus            1  MDT-ANLPRN-----LIIGQSR-RRKKIRDNIMGITRPAIRKLARRGGIIRIKNDIYDEA
A.oryzae            1  MDT-ANLPRN-----LIIGQSR-RRKKIRDIIMGITRPAIRKLARRGGIIRIKSDIYDEA
A.terreus           1  MDP-TRLPRTW------RPKAK-RHKKLRDNLMGITRPAIRRLARRGGVIRIKADIYDEI
A.zonatus           1  MIT-PPPTGNPLTGGFPALPRR-GRKPIRDNILGVSRQSIRRLARRGGIARINIDIYQGV
A.nidulans_H4       1  MS-----GRGKGGKGLGKGGAKRHRKILRDNIQGITKPAIRRLARRGGVKRISAMIYEET

A.acidus           57  RKTVKNRLTEIIRQILLVMESSTTPGHERKVVRTEDVVFALNRMGHTLYGFG--------
A.kawachii         57  RKTVKNRLTEIIRQILLVMESSTTPGHERKVVRTEDVVFALNRMGHTLYGFG--------
A.tubingensis      57  RKTVKNRLTEIIRQILLVMESSTTPGHERKVVRTTDVVFALNRMGHTLYGFG--------
A.niger            57  RKTVKNRLTEIIRQIILVMESSTTPGHERKLVRTQDVVFALNRMGHTLYGFG--------
A.niger            57  RKTVKNRLTEIIRQIILVMESSTTPGHERKLVRTQDVVFAFNRMGHTLYGFG--------
A.carbonarius      57  RVALKDRLTEILRQVVHIMDSSTTPRRERKVVTTRDVVFALNRMGHTLYGFNT------T
A.aculeatus        58  RRALKNRLTEIIRRIVHVMDSGTTPGHERKVVTTRDVTFVLNQMGNPIYGFQ--------
A.nidulans         60  RIVLKERLAETLKQVCLVMESGTIPSSERKLVTTRDVVYALKRMGQTIYGFDRVSSERRI
A.wentii           49  RGVLKDRLREILRHVVYILESARTPSHERKVVTTRDVVYALQKIGHTVYGFAE------I
A.versicolor       53  RLALRDRLSEILRHIMLILESSTVPSHQRKLVTTRDVVFALSRMGHTLYGFD--------
A.sydowii          53  RIVLKERLSDIIRNIVLVLESSTSPSHQRKLVTTRDVVYALNRMGHTLYGFQ--------
A.fumigatus_A11    59  RSVIRGRLREILFQVVQVLESSKTHRHDRKVVTTRDVVYALQRMGQTMYGF---------
A.fumigatus_Af2    59  RSVIRGRLREILFQVVQVLESSKTHRHDRKVVTTRDVVYALQRMGQTMYGF---------
N.fischeri         58  RSVIRGRLREILFQVVQVLESSKTHRHDRKVVTTRDVVYALQRMGQTMYGF---------
A.clavatus         57  RVAIRQRLREVLFQVVQVLDSASTHRHQRKVVTTRDVVYALQRIGQTLYGF---------
A.flavus           54  RRAIRDRLTEILRQLALILESSDIHSRQRKVVTTRDVIYALNRLGNTLYGF---------
A.oryzae           54  RRAIRDRLTEILRQVALILESSNVHSRQRKVVTTRDVIYALNRLGNTLYGF---------
A.terreus          53  RRVVKNRIEDVLRQVVDIMDSASTNRTDRKVVTTRDIVYALNRMGNTLYGFD--------
A.zonatus          59  RQVIKDRLELLISQLVELLESGNTPDHERTIVDSRDVVYVLSRLGTPMYGF---------
A.nidulans_H4      56  RGVLKTFLEGVIRDAVTYTEHA-----KRKTVTSLDVVYALKRQGRTLYGFGG-------
```

Putative H4 Homologue
Alignment

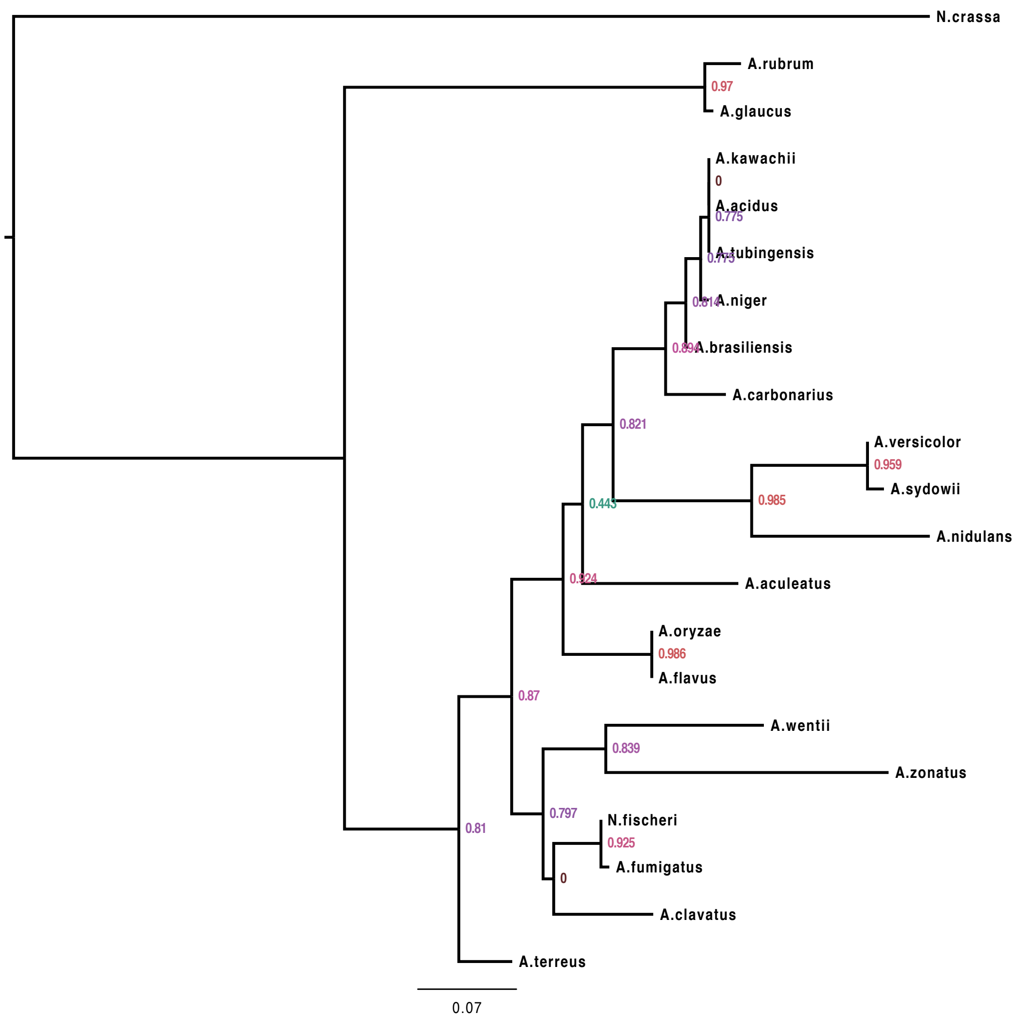

H1

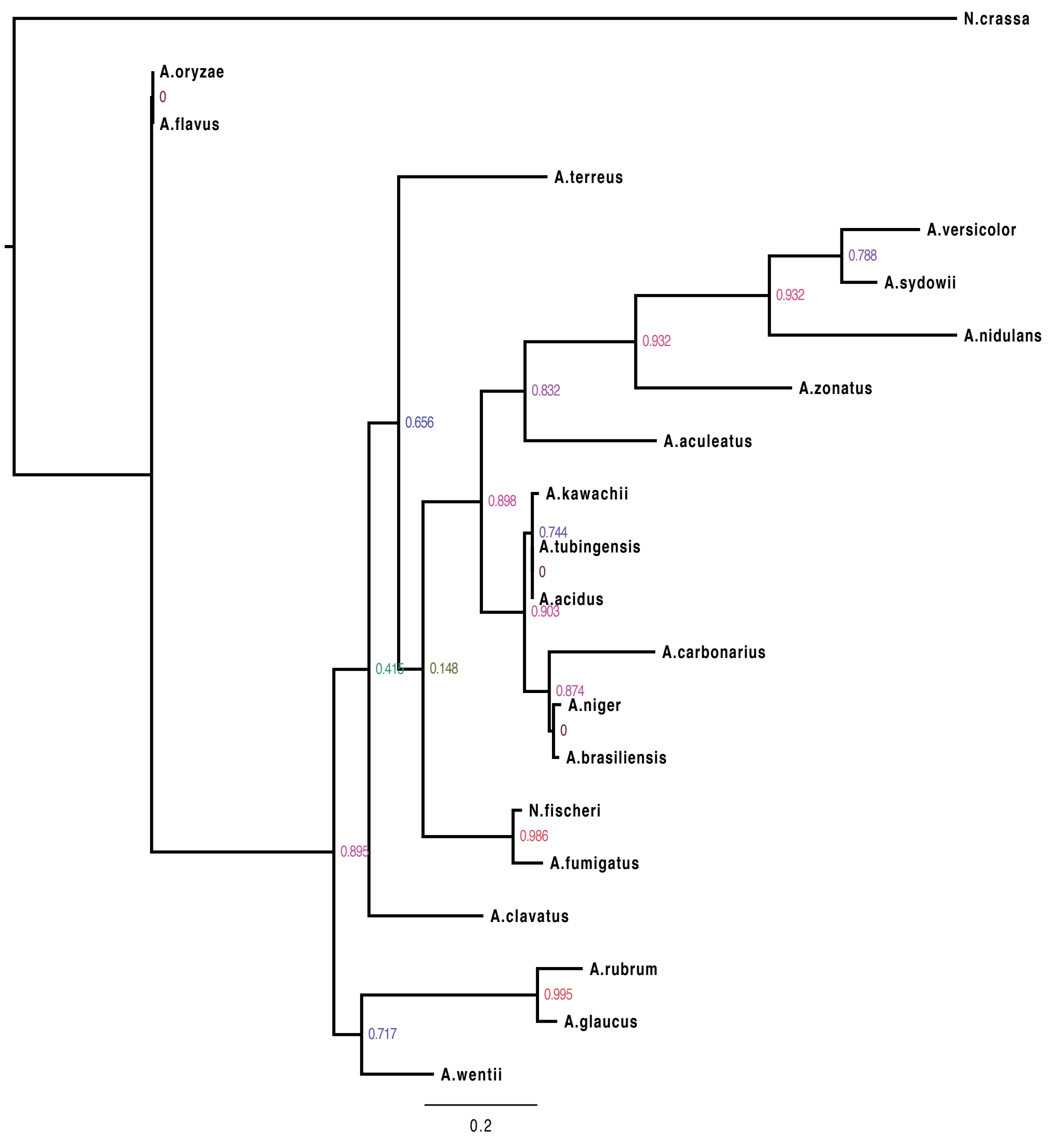

CenH3

Maximum likelihood phylogenetic trees

Additional File 34. Histones of the Aspergilli

This file shows alignments of the histone H1, the centromeric histone H3 and the putative histone showing similarity with histone H4 for different species of *Aspergillus*. Alignments carried out with MAFFT (G-ins-i) (http://mafft.cbrc.jp/alignment/server/) and visualised with BoxShade . (http://www.ch.embnet.org/software/BOX_form.html). The last page of this file compares the phylogenetic trees of the two most variables histones, H1 and CenH3. Trees are maximum likelihood trees derived from a MAFFT (G-ins-i) alignment curated with BMGE (http://mobyle.pasteur.fr/cgi-bin/portal.py#forms::BMGE) with default settings. Maximum likelihood trees with PhyML (http://phylogeny.lirmm.fr/phylo_cgi/alacarte.cgi). Digits at the nodes are aLTRs (approximate likelihood ratio test values).
